# Supplementary material for: Self-assembly of polycyclic supramolecules using linear metal-organic ligands
Source: Nat Commun. 2018 Nov 1;9:4575. doi: 10.1038/s41467-018-07045-9 (PMC6212524; doi:10.1038/s41467-018-07045-9)
Supplement: Supplementary file 1 — Supplementary Information [file 41467_2018_7045_MOESM1_ESM.pdf]

# Self-Assembly of Polycyclic Supramolecules Using Linear Metal-Organic Ligands

Bo Song,[1,+] , Sneha Kandapal[2,+] Jiali Gu,[3] Keren Zhang ,[2] Alex Reese,[2] Yuanfang Ying,[4] Lei Wang,[1] Heng Wang,[1] Yiming Li,[1] Ming Wang,[5] Shuai Lu,[6] Xin-Qi Hao,[6,*] Xiaohong Li,[3,*] Bingqian Xu,[2,*] Xiaopeng Li[1,*]

[1]*Department of Chemistry, University of South Florida, Tampa, Florida 33620, United States*

[2]*Single Molecule Study Laboratory, College of Engineering and Nanoscale Science and Engineering Center, University of Georgia, Athens, Georgia 30602, United States*

[3]*College of Chemistry, Chemical Engineering and Materials Science, Soochow University, Suzhou 215123, China*

[4]*Department of Chemistry and Biochemistry, Texas State University, San Marcos, Texas 78666, United States*

[5]*State Key Laboratory of Supramolecular Structure and Materials, College of Chemistry, Jilin University, Changchun, Jilin 130012, China*

[6]*College of Chemistry and Molecular Engineering, Zhengzhou University, Zhengzhou, Henan 450001, China*

[+] These authors contributed equally to this work.

*Correspondence and requests for materials should be addressed to

E-mail: xiaopengli1@usf.edu (X. L.); bxu@engr.uga.edu (B. X.); lxh83@suda.edu.cn (X-H. L.); xqhao@zzu.edu.cn (X-Q. H.)

# Table of Content

**Supplementary Methods** ........ 3
**Three approaches to terpyridine-Ru(II) metal-organic ligands** ........ 6
**Synthesis of intermediates and ligands L1-L5** ........ 7
**Synthesis of supramolecules C1-C5** ........ 44
**ESI-MS for ligands L1-L5** ........ 54
**ESI-MS for supramolecules C1-C5** ........ 57
**AFM, TEM, and STM images** ........ 64
**Experimental and theoretical collision cross sections** ........ 66
**NMR spectra for ligands L1-L5** ........ 68
**NMR spectra for supramolecules C1-C5** ........ 94
**NMR spectra of isomers for compound 15, compound 21** ........ 124
**$^1$H NMR comparison of ligand L1-L4 with corresponding supramolecules** ........ 126
**DOSY NMR comparison between C3 and C3'** ........ 130
**Synthesis of L5' using end-cap strategy and possible products** ........ 131
**Self-assembly of cis L2 and L5 for C2-like and C5-like supramolecules** ........ 132
**Fitting procedures to derive size parameters from log D** ........ 133
**Supplementary References** ........ 137

## Supplementary Methods

**General Methods**. All reagents were purchased from Sigma-Aldrich, Matrix Scientific, Alfa Aesar or Ark Pharm and were used without further purification. Column chromatography was conducted using neutral $Al_2O_3$ (Brockman II, activity, 58 Å) or $SiO_2$ (VWR, 40-60 µm, 60 Å) and the separated products were identified by UV light or detected by ESI-TOF-MS. NMR spectra data were recorded on Varian inova 400 MHz, Varian inova 500 MHz, Varian inova 600 MHz, Varian dd 600 MHz, Bruker Avance 400 MHz as well as Bruker Avance 500 MHz NMR spectrometers in $CDCl_3$, $CD_3CN$, $CD_3OD$ or DMSO-$d_6$ with TMS as reference. Some of the Ru(II) complexes were run using a mixed solvent of $CDCl_3$ with 10% $CD_3OD$. $F^{19}$ NMR spectra were recorded on a Varian inova-400 MHz spectrometer using trifluoromethanesulfonic acid (δ -78.50 ppm) or $PF_6^-$ (δ -71.11 ppm)[1] as the standard. All chemical shifts were given in ppm. ESI-TOF-MS and TWIM-MS were recorded on a Waters Synapt G2 mass spectrometer, using solutions of 0.01 mg sample in 1 mL of $CHCl_3/CH_3OH$ (1:3, v/v) for ligands or 0.5 mg sample in 1 mL of MeCN/MeOH (3:1, v/v) for supramolecules.

**TWIM-MS**. The TWIM-MS experiments were performed under the following conditions: ESI capillary voltage, 3 kV; sample cone voltage, 30 V; extraction cone voltage, 3.5 V; source temperature 100 ºC; desolvation temperature, 100 ºC; cone gas flow, 10 L per hour; desolvation gas flow, 700 L per hour ($N_2$); source gas control, 0 mL per minute; trap gas control, 2 mL per minute; helium cell gas control, 100 mL per minute; ion mobility (IM) cell gas control, 30 mL per minute; sample flow rate, 5 µL per minute; IM traveling wave height, 25 V; and IM traveling wave velocity, 1000 m per second. Q was set in rf-only mode to transmit all ions produced by ESI into the triwave region for the acquisition of TWIM-MS data.

**Collision cross-section calibration**. The calibration procedure of Scrivens et. al[2] was used to convert the drift time scale of the TWIM-MS experiments to a collision cross-section (CCS) scale. The calibration curve was constructed by plotting the corrected CCSs of the molecular ions of myoglobin against the corrected drift times of the corresponding molecular ions measured in TWIM-MS experiments at the same traveling wave velocity, traveling wave height and ion mobility gas flow settings viz., 1000 m/s, 25 V, and 30 mL/min, respectively.

**Molecular modeling**. Energy minimization of the macrocycles was conducted with Materials Studio version 4.2, using the Anneal and Geometry Optimization tasks in the Forcite module (Accelrys Software, Inc.). All counterions were omitted. An initially energy-minimized structure was subjected to 80 - 100 annealing cycles with initial and mid-cycle temperatures of 300 and 1500 K, respectively, twenty heating ramps per cycle, one thousand dynamic steps per ramp, and one femtosecond per dynamic step. A constant volume/constant energy (NVE) ensemble was used and the geometry was optimized after each cycle. Geometry optimization used a universal force field with atom-based summation and cubic spline truncation for both the electrostatic and Van der Waals parameters. 75-80 energy-minimized structures were used for the calculation of theoretical collision cross-sections using MOBCAL program[3].

**TEM**. The sample was drop-casted on to a lacey carbon coated Cu grid (300 mesh, purchased from Ted Pella Inc.) or carbon-coated Cu grid (400 mesh, purchased from SPI supplies), and the extra solution was absorbed by filter paper to avoid aggregation. The TEM images of the drop casted samples were taken with a FEI Morgagni transmission electron microscope,

**AFM.** AFM imaging was carried out with a Digital Instrument Nanoscope Dimension 3000 system. The sample was diluted to a concentration of $10^{-6}$ M using acetonitrile, then dropped on freshly

cleaved mica surface and dried in the air. Silicon cantilevers tip with spring constant of around 0.1N/m was used for the experiments.

**STM**. The sample was dissolved in DMF at a concentration of 5.0 mg/mL. Sample Solution (5 uL) was dropped on HOPG surface. After 30 seconds, surface was washed slightly with water for three times and acetone for once, then dried at room temperature in air. The STM images were taken with a PicoPlus SPM system with a PicoScan 3000 Controller. The 3D STM images were processed using a free Scanning Probe Microscope (SPM) image analysis software (Gwyddion 2.4).

### Three approaches to terpyridine-Ru(II) metal-organic ligands

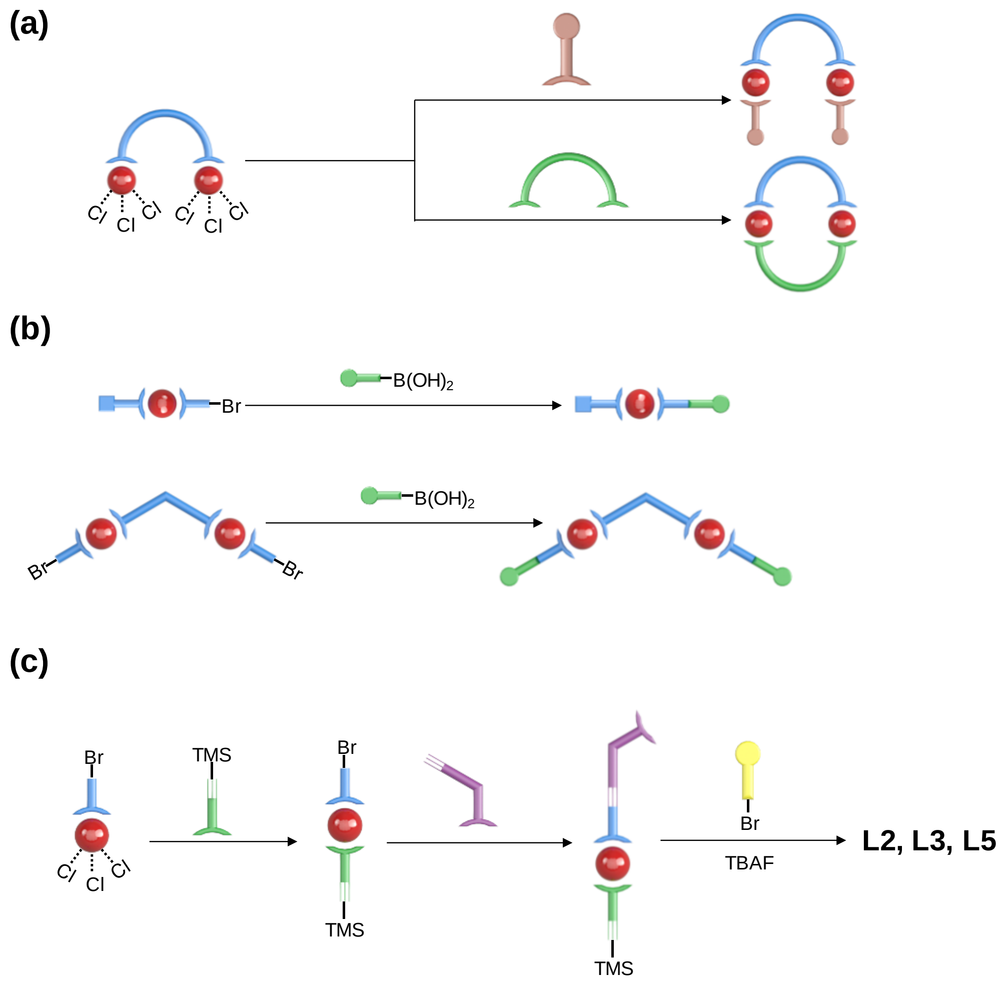


**Supplementary Figure 1**. Three approaches to terpyridine-Ru(II) metal-organic ligands. End-capping approach based on the coordination with Ru(III) complex followed by reduction (a)[4-11]; Suzuki coupling reaction on terpyridine Ru(II) complex (b)[12-17]; Sonogashira coupling reaction on terpyridine Ru(II) complex (This work) (c). Cardinal ball = Ru.

**Synthesis of intermediates and ligands L1-L5.**

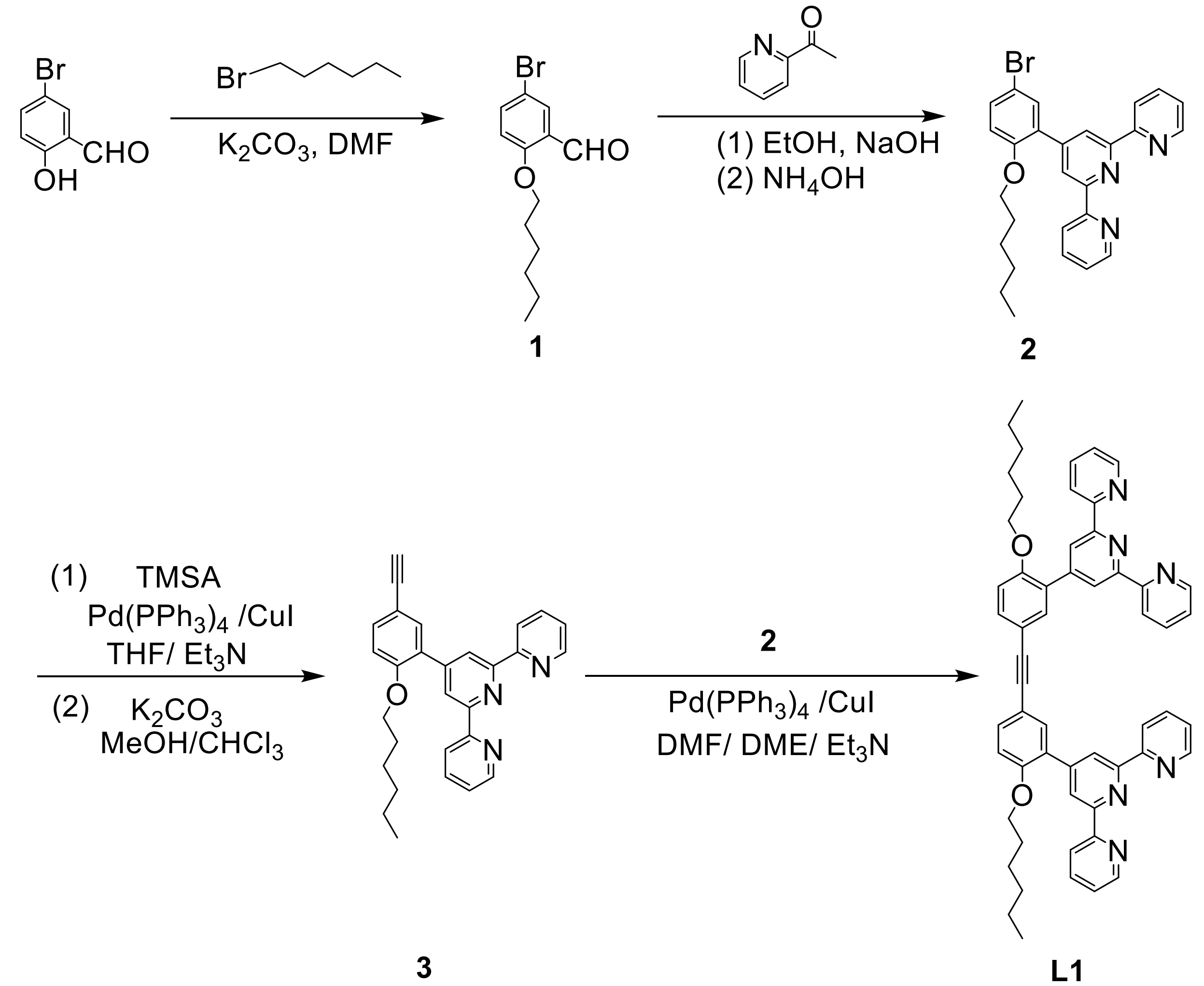


**Supplementary Figure 2**. Synthesis of Ligand **L1**

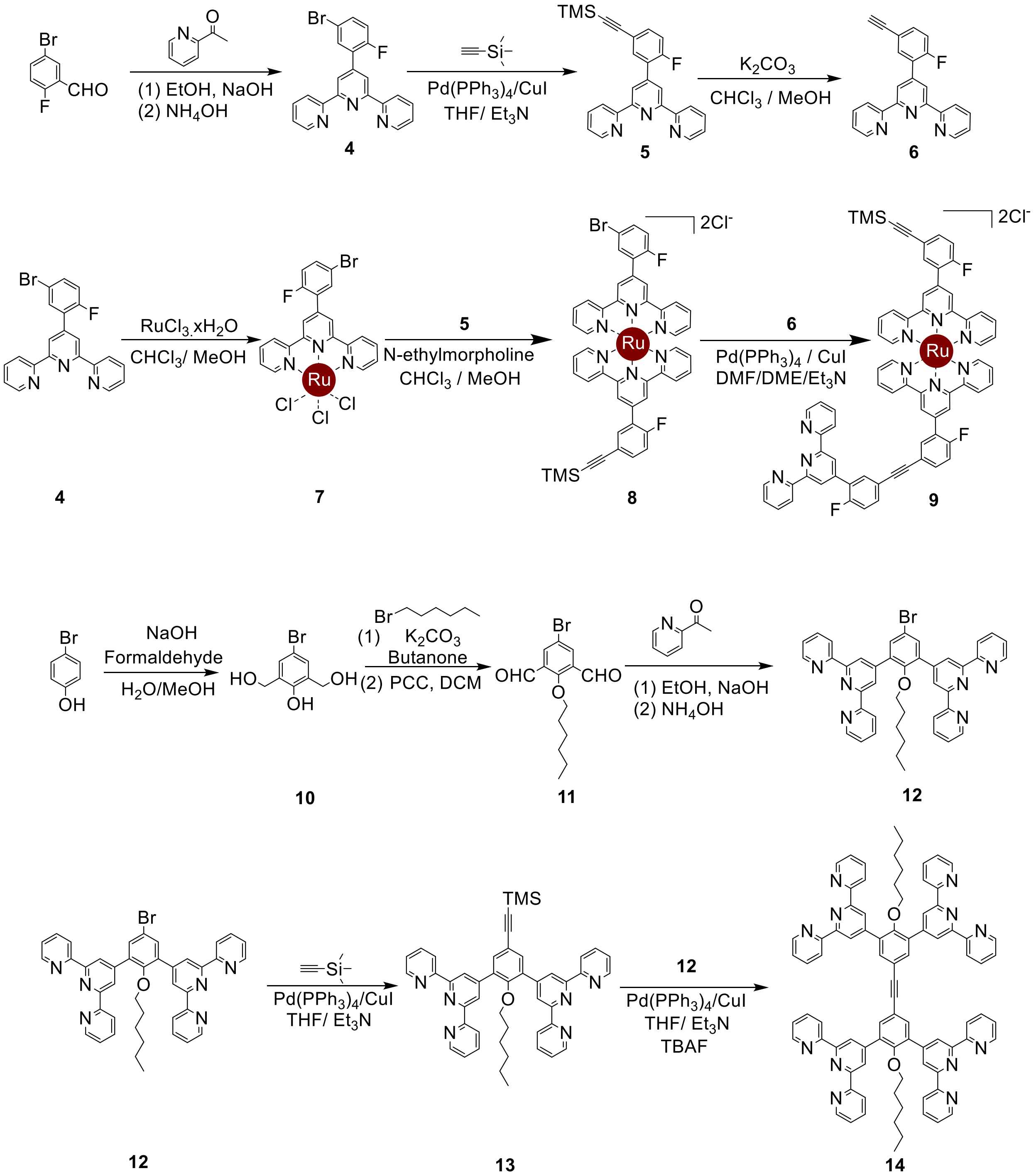


**Supplementary Figure 3**. Synthesis of Intermediates **4-14**

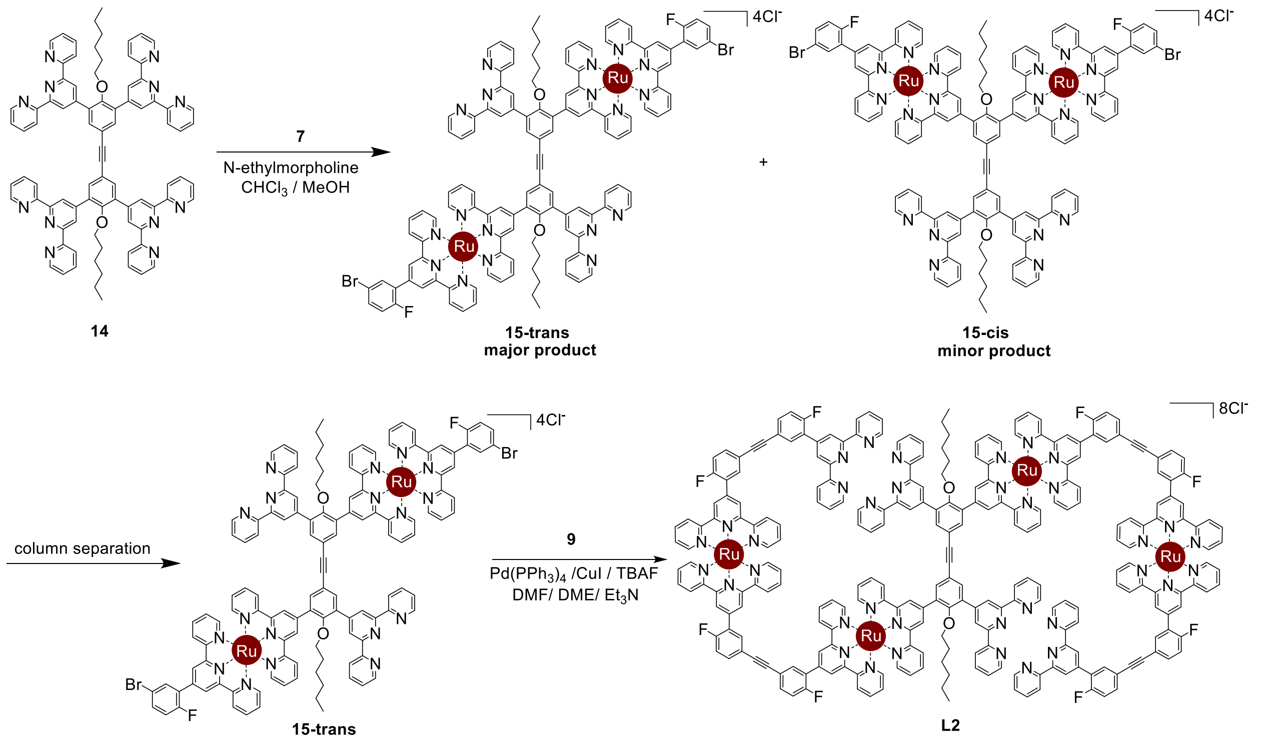


**Supplementary Figure 4**. Synthesis of Ligand **L2**

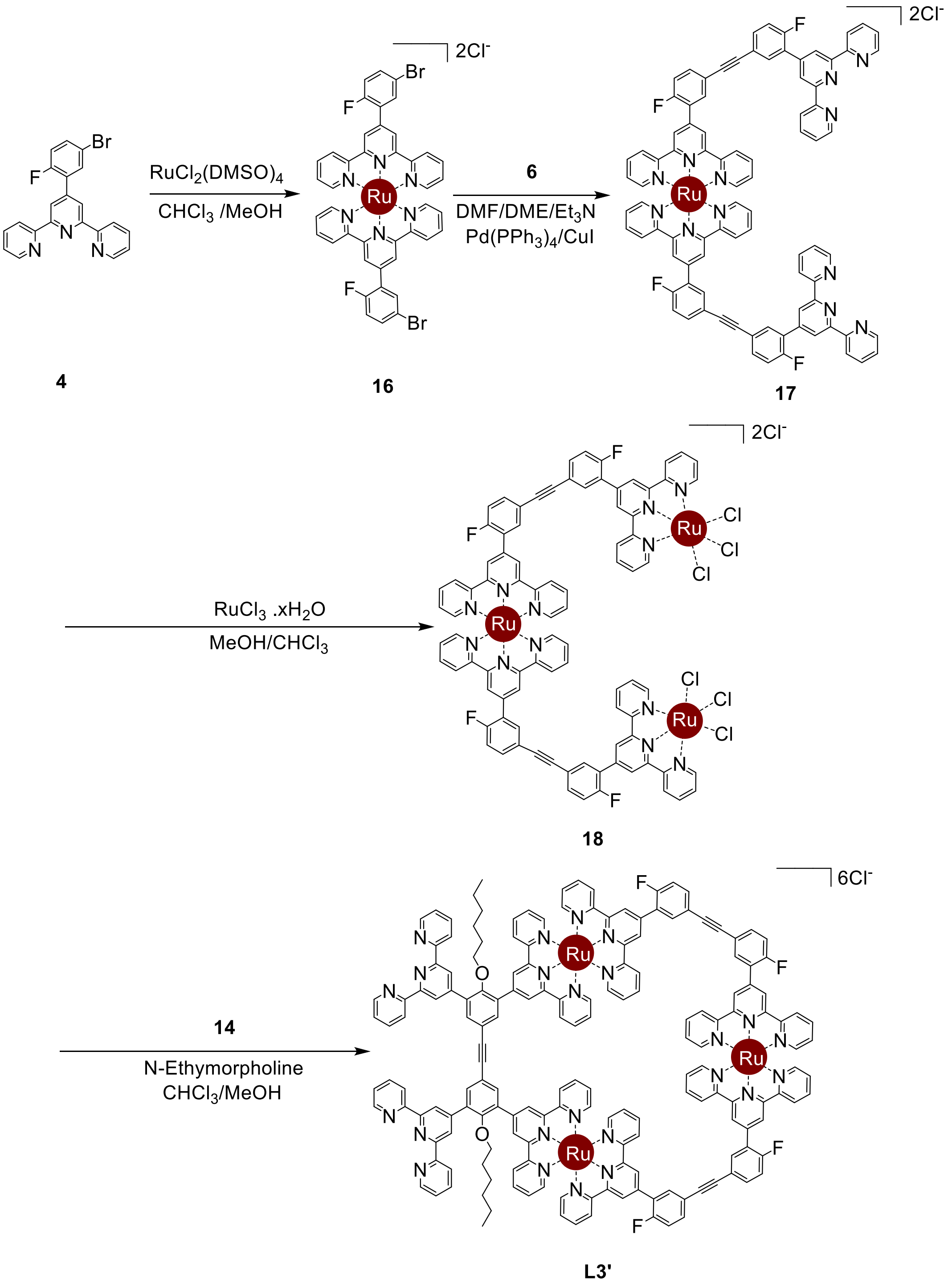


**Supplementary Figure 5**. Synthesis of Ligand **L3'**

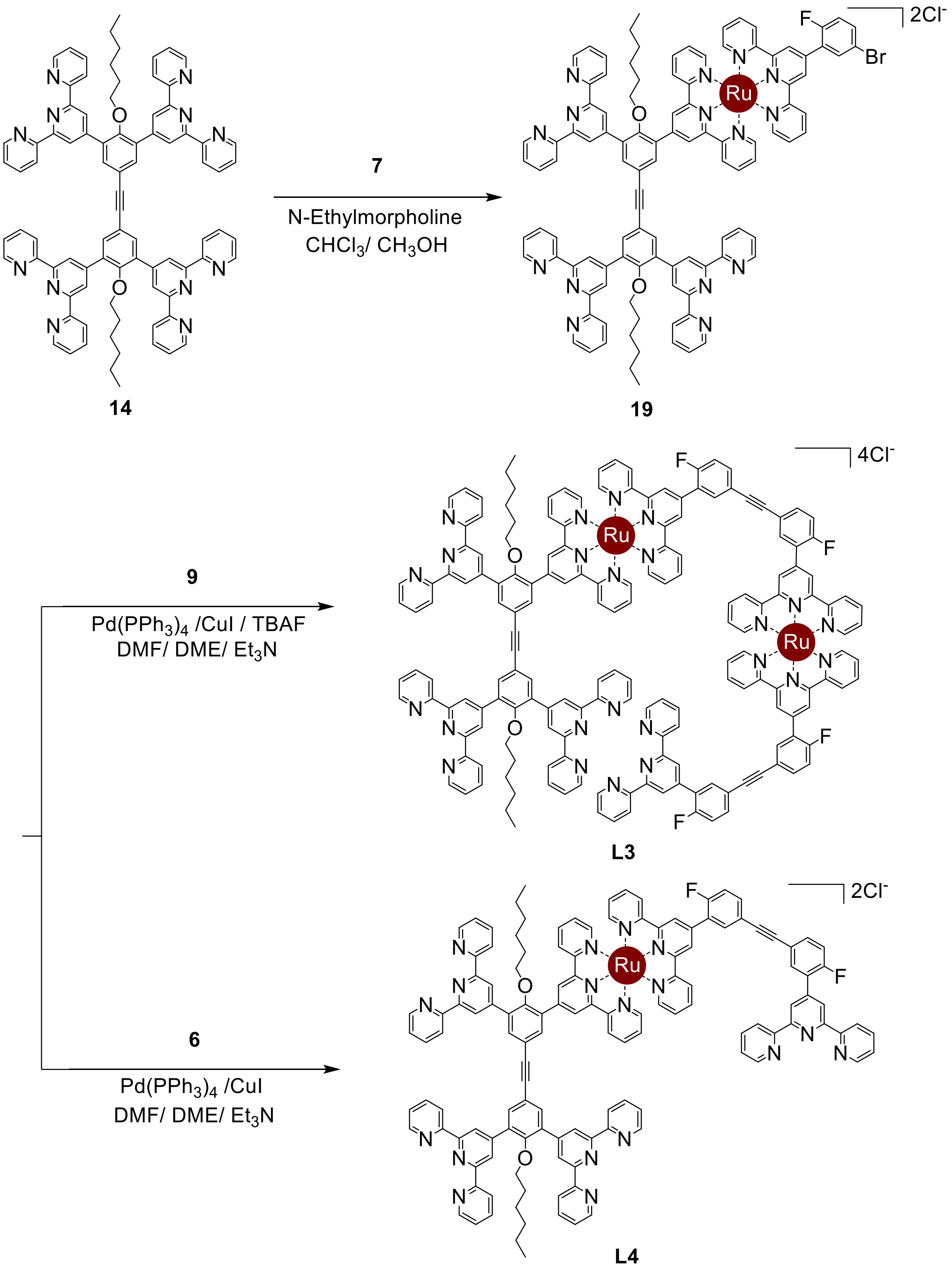


**Supplementary Figure 6**. Synthesis of Ligands **L3** & **L4**

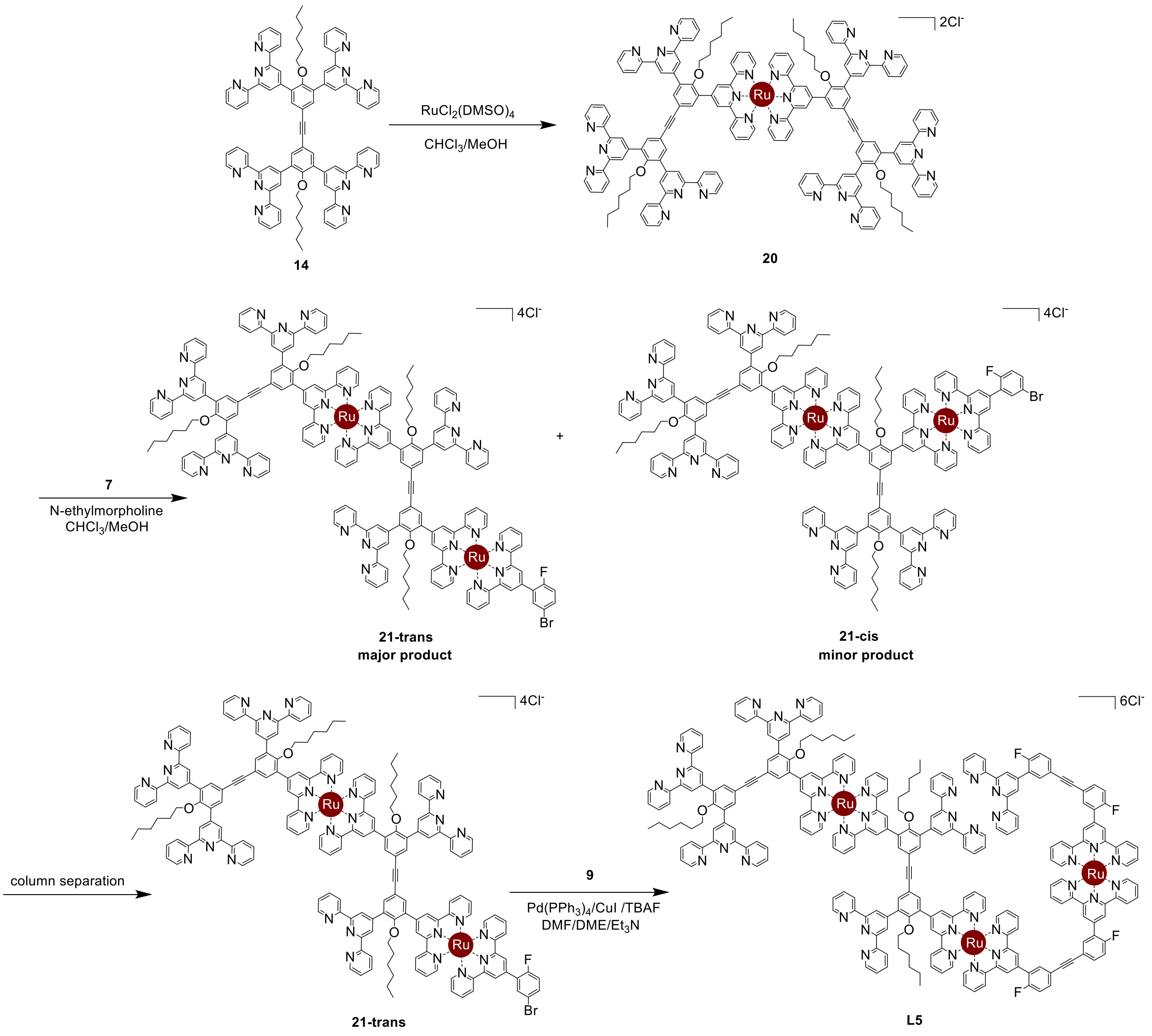


**Supplementary Figure 7**. Synthesis of Ligand **L5**

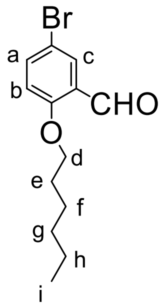


**Compound 1** To a solution of 2-hydroxyl-5-bromo benzaldehyde (5.0 g, 25.0 mmol) and 1-bromohexane (4.5 g, 27.5 mmol) in 80 mL of DMF, $K_2CO_3$ (6.9 g, 50.0 mmol) was added. The mixture was heated at 110 °C overnight and then cooled down to room temperature. After removing the solvent under reduced pressure, the crude product was purified by column chromatography on silica gel (hexane/DCM = 100/5) to give compound **1** as a pale yellow oil (6.2 g, 88% yield). $^{1}$H NMR (400 MHz, $CDCl_3$, 300K) δ 10.45 (s, 1H, $H^{-CHO}$ ), 7.91 (d, $J$ = 2.8 Hz, 1H, $H^{c}$ ), 7.60 (ddd, $J$ = 8.9, 2.4, 0.2 Hz, 1H, $H^{a}$), 6.89 (d, $J$ = 8.9 Hz, 1H, $H^{b}$), 4.10 (t, J = 6.4 Hz, 2H, $H^{d}$ ), 1.98 – 1.75 (m, 2H, $H^{e}$), 1.56 – 1.45 (m, 2H, $H^{f}$ ), 1.42 – 1.33 (m, 4H, $H^{g}$ &$H^{h}$), 0.92 (t, J = 6.3 Hz, 3H, $H^{i}$). $^{13}$C NMR (100 MHz, $CDCl_3$, 300K) δ 188.38, 160.42, 138.22, 130.77, 126.16, 114.58, 113.20, 69.00, 31.45, 28.93, 25.65, 22.53, 13.96.

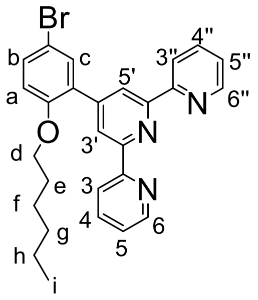


**Compound2.** To a solution of NaOH (3.2 g, 80 mmol) in 70 mL EtOH, 2-(hexyloxy)-5-bromo benzaldehyde (4.26 g, 15.0 mmol) and 2-acetylpyridine (4.0 g, 33.0 mmol) was added. After stirring at 25 ºC for 10 h, aqueous $NH_3 \cdot H_2O$ (50 mL) was added and the mixture was refluxed for

20 h. After cooling down to room temperature, the solvent was removed under reduced pressure, and the aqueous phase was extracted using DCM and the organic layer was washed with water for three times and dried over anhydrous $Na_2SO_4$. After removing the solvent under reduced pressure, the residue was purified by column chromatography on silica gel with DCM as eluent to afford compound **2** as white solid (5.2 g, 71% yield). $^1$H NMR (400 MHz, $CDCl_3$, 300K) δ 8.70 (ddd, $J$ = 4.8, 1.8, 0.9 Hz, 2H, tpy-$H^{6,6''}$), 8.68 – 8.65 (m, 2H, tpy-$H^{3,3''}$), 8.66 (s, 2H, tpy-$H^{3',5'}$), 7.86 (td, $J$ = 7.7, 1.8 Hz, 2H, tpy-$H^{4,4''}$), 7.68 (d, $J$ = 2.5 Hz, 1H, $H^{c}$), 7.44 (dd, $J$ = 8.8, 2.5 Hz, 1H, $H^{b}$), 7.37 – 7.28 (m, 2H, tpy-$H^{5,5''}$), 6.86 (d, $J$ = 8.8 Hz, 1H, $H^{a}$), 3.97 (t, $J$ = 6.2 Hz, 2H, $H^{d}$), 1.69 (dt, $J$ = 14.4, 6.3 Hz, 2H, $H^{-e}$), 1.44 – 1.29 (m, 2H, $H^{f}$), 1.21 – 1.03 (m, 4H, $H^{g}$ &$H^{h}$), 0.72 (t, $J$ = 7.1 Hz, 3H, $H^{i}$). $^{13}$C NMR (101 MHz, $CDCl_3$, 300K) δ 156.42, 155.64, 155.33, 149.20, 147.16, 136.89, 133.08, 132.55, 130.46, 123.77, 121.78, 121.34, 114.12, 112.99, 68.99, 31.61, 29.17, 25.85, 22.45, 14.02. ESI-TOF ($m/z$): Calcd. $[C_{27}H_{26}BrN_3O+H]^+$ for 488.13, found 488.13.

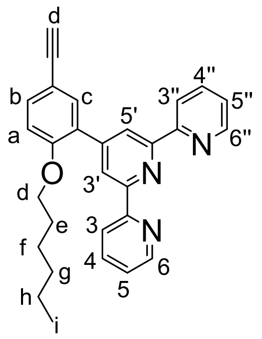


**Compound 3** Compound **2** (1.0 g, 2.0 mmol), $Pd(PPh_3)_4$ (111.5 mg, 0.1 mmol) and CuI (15.3 mg, 0.08 mmol) were mixed in a 100 mL Schlenk flask. After degassing and backfill with nitrogen for three times, 30 mL of THF and 30 mL of $Et_3N$ were added under nitrogen atmosphere. After the addition of TMSA (400 µL, 2.8 mmol), the mixture was stirred at 60 °C for 16h. After cooling down to room temperature, 100 mL of water was added. The mixture was extracted with

chloroform, and the organic layer was dried over anhydrous $Na_2SO_4$ and filtered through celite. After the evaporation of solvent under vacuum, 30 mL of chloroform and 30 mL of methanol was added, followed by the addition of $K_2CO_3$ (1.38 g, 10 mmol) in one portion. The suspension was then stirred at room temperature for 2h, and another 100 mL of water was added. With the extraction using chloroform, the organic layer was dried over anhydrous $Na_2SO_4$ and the solvent was evaporated under vacuum. The crude product was purified via column chromatography on silica gel ($CHCl_3$/MeOH = 100/1) to give compound **3** as a pale yellow solid (653 mg, 87% yield). $^1$H NMR (400 MHz, $CDCl_3$, 300K) δ 8.71 – 8.68 (m, 2H, , tpy-$H^{6,6''}$), 8.68 (s, 2H, tpy-$H^{3',5'}$), 8.66 (dt, *J* = 8.0, 1.1 Hz, 2H, tpy-$H^{3,3''}$), 7.85 (ddd, *J* = 8.0, 7.5, 1.8 Hz, 2H, tpy-$H^{4,4''}$), 7.73 (d, *J* = 2.1 Hz, 1H, $H^c$), 7.50 (dd, *J* = 8.5, 2.2 Hz, 1H, $H^b$), 7.31 (ddd, *J* = 7.5, 4.8, 1.2 Hz, 2H, tpy-$H^{5,5''}$), 6.93 (d, *J* = 8.6 Hz, 1H, $H^a$), 4.01 (t, *J* = 6.2 Hz, 2H, $H^d$), 3.03 (s, 1H, $H^d$), 1.71 (ddt, *J* = 9.3, 7.9, 6.2 Hz, 2H, $H^e$), 1.50 – 1.32 (m, 2H, $H^f$), 1.27 – 1.00 (m, 4H, $H^g$ &$H^h$), 0.72 (t, *J* = 7.2 Hz, 3H, $H^i$). $^{13}$C NMR (101 MHz, $CDCl_3$, 300K) δ 156.87, 156.51, 155.31, 149.20, 147.52, 136.81, 134.46, 133.97, 128.53, 123.70, 121.85, 121.29, 114.39, 112.17, 83.50, 76.34, 68.76, 31.61, 29.15, 25.86, 22.45, 14.01. ESI-TOF (*m/z*): Calcd. $[C_{29}H_{27}N_3O+H]^+$ for 434.22, found 434.21.

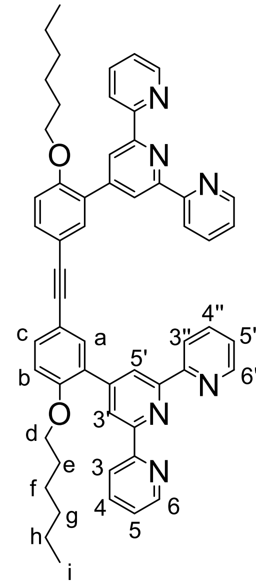


**Ligand L1** Compound **2** (400 mg, 0.82 mmol) and compound **3** (499 mg, 0.98 mmol) were mixed in a 100 mL Schlenk flask, $Pd(PPh_3)_4$ (47.3 mg, 0.041 mmol) and CuI (6.2 mg, 0.033 mmol) were added. After degassing and backfill with nitrogen for three times, 30 mL of THF and 30 mL of $Et_3N$ were added under nitrogen atmosphere. After the addition of TBAF (0.98 mL, 0.98 mmol, 1 mol/L in THF), the mixture was stirred at 70 °C for 16h. After that the reaction was cooled down to room temperature and 100 mL of water was added. The mixture was extracted with chloroform, and the organic layer was collected, dried over anhydrous sodium sulfate and the solvent was evaporated under vacuum. The crude product was purified via column chromatography on silica gel ($CHCl_3$/MeOH = 100/2) to give **L1** as a white solid (374 mg, 52.3% yield). $^1$H NMR (500 MHz, $CDCl_3$, 300K) δ 8.75 (s, 2H, tpy-$H^{3',5'}$), 8.73 (d, $J$ = 4.3 Hz, 2H, tpy-$H^{6,6''}$), 8.69 (d, $J$ = 7.9 Hz, 2H, tpy-$H^{3,3''}$), 7.88 (t, $J$ = 7.4 Hz, 2H tpy-$H^{4,4''}$), 7.81 (d, $J$ = 1.1 Hz, 1H, $H^a$), 7.57 (d, $J$ = 8.4 Hz, 1H, $H^c$), 7.39 – 7.31 (m, 2H, tpy-$H^{5,5''}$), 7.00 (d, $J$ = 8.6 Hz, 1H, $H^b$), 4.06 (t, $J$ = 6.1 Hz, 2H, $H^d$), 1.81 – 1.68 (m, 2H, $H^e$), 1.46 – 1.38 (m, 2H, $H^f$), 1.25 – 1.05 (m, 4H, $H^g$ &$H^h$), 0.76 (t, $J$ = 7.0 Hz, 3H, $H^i$). $^{13}$C NMR (126 MHz, $CDCl_3$, 300K) δ 156.47, 156.27, 155.16, 149.11, 147.71,

136.76, 133.78, 133.21, 128.48, 123.58, 121.90, 121.23, 115.83, 112.22, 88.25, 68.65, 31.55, 29.12, 25.81, 22.38, 13.95. ESI-TOF (*m/z*): Calcd. $[C_{56}H_{52}N_6O_2+H]^+$ for 841.42, found 841.43.

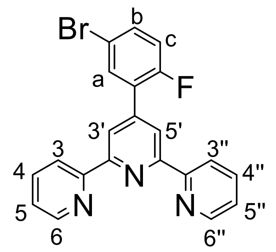


**Compound 4** To To a solution of NaOH (3.2 g, 80 mmol) in 70 ml EtOH, 4-bromo-2-fluorobenzaldehyde (3.03 g, 15.0 mmol) and 2-acetylpyridine (4.0 g, 33.0 mmol) was added. After stirring at 25 °C for 10 h, aqueous $NH_3 \cdot H_2O$ (50 mL) was added and the mixture was refluxed for 20 h. After cooling down to room temperature, the yellow precipitate was collected via vacuum filtration and washed with ethanol. The crude product was recrystallized using $CHCl_3$/EtOH to give compound **4** as a white solid (3.2 g, 53% yield). $^1$H NMR (400 MHz, $CDCl_3$, 300K) δ 8.75 (ddd, *J* = 4.8, 1.8, 0.9 Hz, 2H, tpy-$H^{6,6''}$), 8.69 (dt, *J* = 8.0, 1.0 Hz, 2H, tpy-$H^{3,3''}$), 8.66 (d, *J* = 1.5 Hz, 2H, tpy-$H^{3',5'}$), 7.92 (td, *J* = 7.8, 1.8 Hz, 2H, tpy-$H^{4,4''}$), 7.84 (dd, *J* = 6.7, 2.5 Hz, 1H, $H^a$), 7.52 (ddd, *J* = 8.7, 4.3, 2.5 Hz, 1H, $H^b$), 7.39 (ddd, *J* = 7.5, 4.8, 1.2 Hz, 2H, tpy-$H^{5,5''}$), 7.11 (dd, *J* = 10.0, 8.7 Hz, 1H, $H^c$). $^{13}$C NMR (101 MHz, $CDCl_3$, 300K) δ 160.23, 157.74, 155.85, 149.18, 144.14, 136.86, 133.23, 133.14, 128.94, 128.80, 123.95, 121.31, 120.71, 120.69, 118.20, 117.96, 117.04. $^{19}$F NMR (376 MHz, $CDCl_3$, 300K) δ -118.45. ESI-TOF (*m/z*): Calcd. $[C_{21}H_{13}BrN_3F+H]^+$ for 406.04, found 406.05.

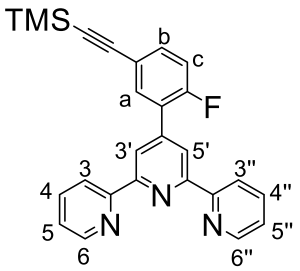


**Compound 5** Compound **4** (810 mg, 2.0 mmol), $Pd(PPh_3)_4$ (111.5 mg, 0.1 mmol) and CuI (15.3 mg, 0.08 mmol) were mixed in a 100 mL Schlenk flask. After degassing and backfill with nitrogen for three times, 30 mL of THF and 30 mL of $Et_3N$ were added under nitrogen atmosphere. After the addition of TMSA (400 µL, 2.8 mmol), the mixture was stirred at 60 °C for 16h. After cooling down to room temperature, 100 mL of water was added. The mixture was extracted with chloroform, and the organic layer was collected, dried over anhydrous sodium sulfate and the solvent was evaporated under vacuum. The crude product was purified via column chromatography on silica gel ($CHCl_3$/MeOH = 100/1) to give **3** as a pale yellow solid (600.1 mg, 71% yield). $^1$H NMR (400 MHz, $CDCl_3$, 300K) δ 8.72 (dt, $J$ = 3.9, 0.9 Hz, 2H, tpy-$H^{6,6''}$), 8.66 (d, $J$ = 8.0 Hz, 2H, tpy-$H^{3,3''}$), 8.64 (s, 2H, tpy-$H^{3',5'}$), 7.95 – 7.85 (m, 2H, tpy-$H^{4,4''}$), 7.82 (dd, $J$ = 7.3, 2.2 Hz, 1H, $H^{a}$), 7.50 (ddd, $J$ = 8.5, 4.7, 2.1 Hz, 1H, $H^{b}$), 7.34 (ddd, $J$ = 7.4, 4.8, 1.3 Hz, 2H, tpy-$H^{5,5''}$), 7.14 (dd, $J$ = 10.3, 8.5 Hz, 1H, $H^{c}$), 0.26 (s, 9H, $H^{\text{-TMS}}$). $^{13}$C NMR (101 MHz, $CDCl_3$, 300K) δ 161.10, 158.57, 156.07, 155.90, 149.28, 144.79, 136.98, 134.49, 134.46, 134.19, 134.11, 127.31, 127.17, 124.02, 121.45, 120.96, 120.93, 120.12, 120.08, 116.74, 116.50, 103.63, 94.82, 0.05. $^{19}$F NMR (376 MHz, $CDCl_3$, 300K) δ -115.34. ESI-TOF (*m/z*): Calcd. $[C_{26}H_{22}FN_3Si+H]^+$ for 424.16, found 424.16.

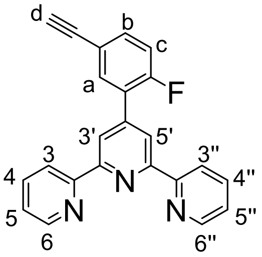


**Compound 6** Compound **5** (600.1 mg, 1.42 mmol) was dissolved in 30 mL chloroform, and 30 mL of methanol was added. After the addition of $K_2CO_3$ (588 mg, 4.26 mmol), the mixture was stirred at room temperature for 3 hours followed by the addition of 100 mL water. The mixture was extracted with chloroform, and the organic layer was collected, dried over anhydrous sodium sulfate and the solvent was evaporated under vacuum. The crude product was purified via flash column chromatography on silica gel ($CHCl_3$/MeOH = 100/1) to give **6** as a white solid (424 mg, 85% yield). $^{1}$H NMR (400 MHz, $CDCl_3$, 300K) δ 8.72 (ddd, *J* = 4.8, 1.8, 0.9 Hz, 2H, tpy-$H^{6,6''}$), 8.68 (t, *J* = 1.1 Hz, 2H, tpy-$H^{3,3''}$), 8.66 (s, 2H, tpy-$H^{3',5'}$), 7.93 – 7.85 (m, 2H, tpy-$H^{4,4''}$), 7.84 (dd, *J* = 5.1 Hz, 2.2 Hz , 1H, $H^{a}$), 7.53 (ddd, *J* = 8.5, 4.7, 2.2 Hz, 1H, $H^{b}$), 7.35 (ddd, *J* = 7.5, 4.8, 1.2 Hz, 2H, tpy-$H^{5,5''}$), 7.17 (dd, *J* = 10.2, 8.5 Hz, 1H, $H^{c}$), 3.10 (s, 1H, $H^{d}$). $^{13}$C NMR (101 MHz, $CDCl_3$, 300K) δ 161.17, 158.64, 155.92, 155.82, 149.17, 144.52, 136.85, 134.53, 134.23, 134.14, 127.33, 127.20, 123.90, 121.30, 120.81, 118.86, 116.74, 116.51, 82.20, 77.58. $^{19}$F NMR (376 MHz, $CDCl_3$, 300K) δ -113.76. ESI-TOF (*m/z*): Calcd. $[C_{23}H_{14}FN_3+H]^+$ for 352.13, found 352.13.

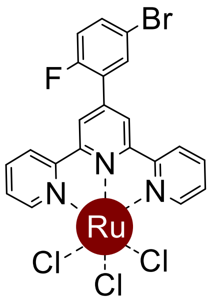


**Compound 7** To a flask containing compound 4 (2.0 g, 4.9 mmol), $RuCl_3.xH_2O$ (2.03 g, 9.8 mmol) was added. With the addition of 100 mL chloroform and 100 mL methanol, the brown slurry was

stirred under reflux for 24h. Afterwards the solvent was removed under vacuum, and the residue was washed with methanol for three times. The solid was collected via filtration and dried under vacuum to give compound **7** as a brown solid (2.8g, 83.6% yield). Due to the extremely poor solubility, compound **7** was directly used for the following steps without further characterization.

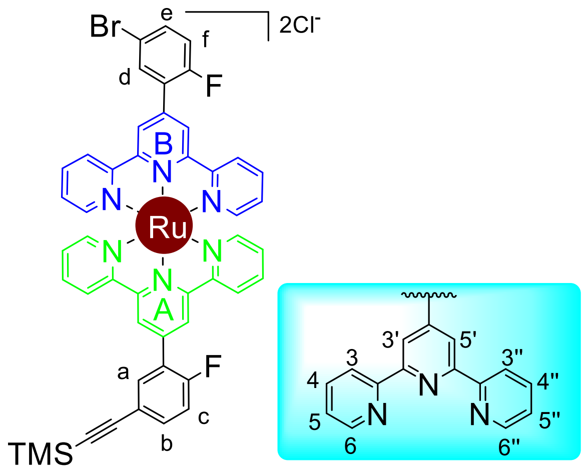


**Compound 8** To a flask containing compound 5 (251 mg, 0.6 mmol) and compound 7 (330 mg, 0.54 mmol), 80 mL chloroform and 80 mL methanol was added. After the addition of N-ethylmorphline (500 uL), the mixture was stirred under reflux for 36h when it became a dark red solution. After cooling down to room temperature, the solvent was evaporated under vacuum, and the crude product was purified via column chromatography on aluminum oxide (DCM/MeOH = 100/1) to give compound **8** as a dark red solid (306.2 mg, 63% yield). $^{1}$H NMR (500 MHz, $CDCl_3$, 300K) δ 9.03 (s, 2H, tpy$^{B}$-$H^{3',5'}$), 8.93 (s, 2H, tpy$^{A}$-$H^{3',5'}$), 8.74 (d, $J$ = 8.1 Hz, 2H, tpy$^{B}$-$H^{3,3''}$), 8.64 (d, $J$ = 8.1 Hz, 2H, tpy$^{A}$-$H^{3,3''}$), 8.55 (dd, $J$ = 6.9, 1.9 Hz, 1H, $H^{d}$), 8.27 (dd, $J$ = 7.4, 1.8 Hz, 1H, $H^{a}$), 7.96 (m, 4H, tpy$^{B}$-$H^{4,4''}$ & tpy$^{A}$-$H^{6,6''}$), 7.73 – 7.63 (m, 2H, $H^{b}$ & $H^{e}$), 7.55 (t, $J$ = 5.4 Hz, 4H, tpy$^{A}$-$H^{4,4''}$ & tpy$^{B}$-$H^{6,6''}$), 7.34 – 7.28 (m, 6H, tpy$^{A}$-$H^{5,5''}$, tpy$^{B}$-$H^{5,5''}$, $H^{c}$ & $H^{f}$), 0.28 (s, 9H, $H^{-TMS}$). $^{13}$C NMR (126 MHz, $CDCl_3$) δ 161.53, 159.13, 153.11, 151.97, 151.77, 149.92, 148.15, 146.80, 146.67, 145.13, 142.63, 140.40, 139.89, 139.15, 137.56, 137.47, 136.42, 135.07, 133.96, 132.45, 132.20, 130.47, 129.11, 127.39, 126.51, 125.81, 124.96, 124.06, 123.24, 117.89, 94.04, 90.17,

3.50. $^{19}$F NMR (376 MHz, $CDCl_3$, 300K) δ -115.10, -119.26. ESI-TOF (*m/z*): Calcd. $[C_{47}H_{35}BrCl_2F_2N_6RuSi-2Cl]^{2+}$ for 465.05, found 465.05.

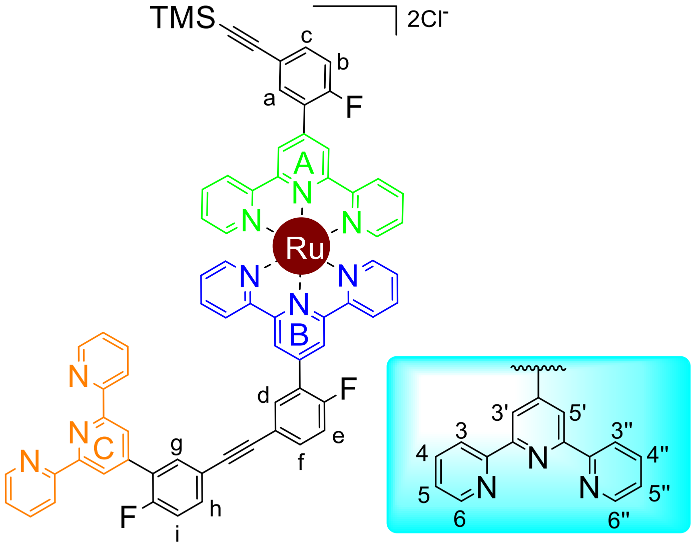


**Compound 9** Compound **8** (240 mg, 0.24 mmol), compound **6** (110 mg, 0.312 mmol), $Pd(PPh_3)_4$ (13.9 mg, 0.012 mmol) and CuI (2.3 mg, 0.012 mmol) were mixed in a 100 mL Schlenk flask. After degassing and backfill with nitrogen for three times, 15 mL of DMF, 15 mL of DME and 15 mL of $Et_3N$ were added under nitrogen atmosphere. The mixture was then stirred at 80 °C for 16h. After cooling down to room temperature, the solvent was evaporated under vacuum. The crude product was purified via column chromatography on aluminum oxide ($CHCl_3$/MeOH = 100/1) to give **9** as a dark red solid (247 mg, 81% yield). $^1$H NMR (500 MHz, $CDCl_3$, 300K) δ 9.03 (s, 2H, tpy$^B$-$H^{3',5'}$), 8.93 (s, 2H, tpy$^A$-$H^{3',5'}$), 8.74 (d, $J$ = 8.1 Hz, 2H, tpy$^B$-$H^{3,3''}$), 8.64 (d, $J$ = 8.1 Hz, 2H, tpy$^A$-$H^{3,3''}$), 8.55 (dd, $J$ = 6.9, 1.9 Hz, 1H, $H^e$), 8.27 (dd, $J$ = 7.4, 1.8 Hz, 1H, $H^b$), 7.96 (t, $J$ = 7.5 Hz, 4H, tpy$^B$-$H^{4,4''}$ & tpy$^A$-$H^{6,6''}$), 7.73 – 7.63 (m, 2H, $H^c$ & $H^f$), 7.55 (t, $J$ = 5.4 Hz, 4H, tpy$^A$-$H^{4,4''}$ & tpy$^B$-$H^{6,6''}$), 7.34 – 7.28 (m, 6H, tpy$^A$-$H^{5,5''}$, tpy$^B$-$H^{5,5''}$, $H^a$ & $H^d$), 0.28 (s, 9H, $H^{-TMS}$). $^{13}$C NMR (126 MHz, $CDCl_3$, 300K) δ 160.77, 160.64, 160.57, 158.74, 158.62, 158.55, 157.75, 157.62, 157.57, 155.92, 155.88, 155.03, 154.95, 154.93, 152.63, 152.54, 149.23, 144.68, 142.79, 138.38,

136.99, 135.03, 134.80, 134.74, 134.38, 134.25, 133.72, 128.37, 127.21, 125.38, 125.10, 124.88, 124.04, 123.94, 121.88, 121.43, 121.18, 120.90, 120.02, 119.67, 118.16, 117.24, 117.12, 116.93, 116.85, 116.74, 102.89, 96.07, 89.42, 88.03, -0.05. $^{19}$F NMR (376 MHz, $CDCl_3$) δ -114.15, -114.96, -115.06.ESI-TOF Calcd. $[C_{70}H_{48}Cl_2F_3N_9RuSi\text{-}2Cl]^{2+}$ for 600.64, found 600.64.

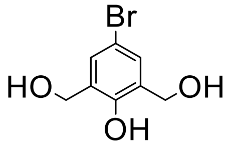


**Compound 10**. Compound **10** was synthesized exactly according to literature report[18]. To an aqueous solution of NaOH (25%, 50 mL) containing p-bromophenol (17.3 g, 0.1 mol) and methanol (25 mL) was added formaldehyde (38%, 90 mL) and the mixture was stirred for 12 days at room temperature. Then, a mixture of water (50 mL) and acetic acid (15 mL) was added. The reaction mixture was stirred for 8 h at room temperature to give a yellow precipitate, which was collected via filtration. The precipitate was then dissolved in 10% aqueous NaOH, followed by acidify with 2 M HCl until pH = 1. The crystals formed was collected via filtration and washed with water, dried to give a pale yellow solid (10.9 g, 47% yield).

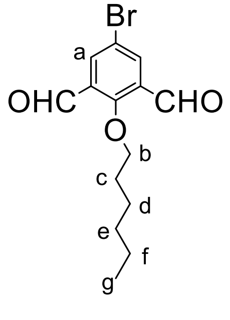


**Compound 11** Compound **11** was synthesized exactly according to literature report[19]. A mixture of 4-bromo-2,6-bis(hydroxymethyl)phenol (6.0 g, 25.9 mmol), 1-bromohexane (5.1 g, 31.1 mmol),

$K_2CO_3$ (7.2 g, 52.0 mmol) and methyl ethyl ketone (200 mL) was refluxed under $N_2$ for 24 h. The mixture was cooled to room temperature, filtrated and dried under vacuum at 60 °C for 6 hours. The white residue was dissolved using 200 mL of DCM, after which PCC (11.8 g, 75 mmol) and celite (20 g) was added. The mixture was stirred at room temperature for 5h with TLC analysis until disappearance of the material. After that the mixture was directly poured onto silica gel column with DCM as eluent to afford compound **11** as a white solid (6.98 g, 86% yield). $^1$H NMR (400 MHz, $CDCl_3$, 300K) δ 10.36 (s, 2H, $H^{-CHO}$), 8.20 (s, 2H, $H^a$), 4.14 (t, $J$ = 6.4 Hz, 2H, $H^b$), 1.95-1.87 (m, 2H, $H^c$), 1.56 – 1.45 (m, 2H, $H^d$), 1.40 – 1.35 (m, 4H, $H^e$ & $H^f$), 0.97 – 0.89 (t, $J$ = 6.4 Hz, 3H, $H^g$). $^{13}$C NMR (100 MHz, $CDCl_3$, 300K) δ 187.13, 139.03, 137.24, 131.76, 118.29, 81.04, 31.48, 29.85, 25.45, 22.49, 13.94.

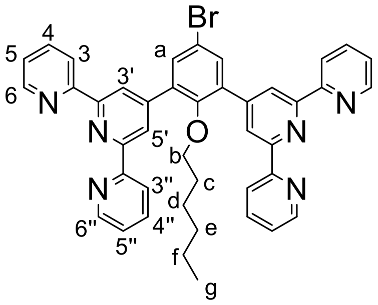


**Compound 12** Compound **12** was synthesized exactly according to literature report[20]. To a solution of NaOH powder (6.24 g, 156.0 mmol) in EtOH (350 mL), 5-bromo-2-(hexyloxy)benzene-1,3-dialdehyde (4.0 g, 13.0 mmol) and 2-acetylpyridine (7.5 g, 62 mmol) were added. After stirring at room temperature for 20 h, aqueous $NH_3$•$H_2O$ (150 mL) was added and the mixture was refluxed for 40 h. Upon cooling to room temperature, the precipitate was filtered and washed with cold ethanol, dried and recrystallized using $CHCl_3$/EtOH to give 6 as a white solid (4.8 g, 51% yield). $^1$H NMR (400 MHz, $CDCl_3$, 300K) δ 8.80 (s, 4H, tpy-$H^{3',5'}$), 8.77 (ddd, $J$ = 4.8, 1.8, 0.9 Hz, 4H, tpy-$H^{6,6''}$), 8.70 (dt, $J$ = 8.0, 1.0 Hz, 4H, tpy-$H^{3,3''}$), 7.91 (td, $J$ = 7.7, 1.8 Hz,

4H, tpy-$H^{4,4''}$), 7.80 (s, 2H, $H^{a}$ ), 7.38 (ddd, *J* = 7.5, 4.8, 1.2 Hz, 4H, tpy-$H^{5,5''}$), 3.37 (t, *J* = 6.4 Hz, 2H, $H^{b}$), 1.24 – 1.17 (dd, *J* = 9.1, 6.2 Hz, 2H, $H^{c}$), 0.92 (s, 2H, $H^{d}$), 0.83 – 0.66 (m, 4H, $H^{e}$ & $H^{f}$), 0.55 – 0.41 (t, *J* = 6.8 Hz, 3H, $H^{g}$). $^{13}$C NMR (100 MHz, $CDCl_3$, 300K) δ 156.10, 155.58, 153.98, 149.20, 147.08, 136.80, 135.89, 133.66, 123.82, 121.45, 121.25, 117.05, 74.33, 31.29, 29.70, 25.39, 22.11, 13.77. ESI-TOF (*m/z*): Calcd. $[C_{42}H_{35}BrN_6O+H]^+$ for 719.21, found 719.21.

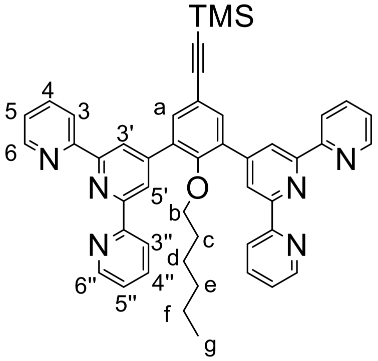


**Compound 13** Compound **12** (810 mg, 2.0 mmol), $Pd(PPh_3)_4$ (111.5 mg, 0.1 mmol) and CuI (15.3 mg, 0.08 mmol) were mixed in a 100 mL Schlenk flask. After degassing and backfill with nitrogen for three times, 40 mL of THF and 30 mL of $Et_3N$ were added under nitrogen atmosphere. After the addition of TMSA (400 µL, 2.8 mmol), the mixture was stirred at 60 °C for 16h. After cooling down to room temperature, 100 mL of water was added. The mixture was extracted with chloroform, and the organic layer was collected, dried over anhydrous sodium sulfate and the solvent was evaporated under vacuum. The crude product was purified via column chromatography on silica gel ($CHCl_3$/MeOH = 100/1) to give **3** as a white solid (785 mg, 94.7% yield). $^1$H NMR (500 MHz, $CDCl_3$) δ 8.78 (s, 4H, tpy-$H^{3',5'}$), 8.77 – 8.72 (m, 4H, tpy-$H^{6,6''}$), 8.68 (d, *J* = 7.9 Hz, 4H, tpy-$H^{3,3''}$), 7.88 (td, *J* = 7.7, 1.8 Hz, 4H, tpy-$H^{4,4''}$), 7.78 (s, 2H, $H^{a}$ ), 7.35 (ddd, *J* = 7.4, 4.8, 1.1 Hz, 4H, tpy-$H^{5,5''}$), 3.35 (t, *J* = 6.0 Hz, 2H, $H^{b}$), 1.17 (m, 2H, $H^{c}$), 0.91 (m, 2H, $H^{d}$), 0.77 – 0.67 (m, 4H, $H^{e}$ & $H^{f}$), 0.48 (t, *J* = 6.8 Hz, 3H, $H^{g}$), 0.34 – 0.21 (s, 9H, $H^{-TMS}$). $^{13}$C NMR (101 MHz, $CDCl_3$) δ 156.23, 155.10, 149.23, 147.70, 136.84, 134.80, 123.83, 121.61,

121.30, 119.59, 104.09, 94.84, 74.41, 31.36, 29.79, 25.46, 22.17, 13.83, 0.07. ESI-TOF (*m/z*): Calcd. $[C_{47}H_{44}SiN_6O+H]^+$ for 737.34, found 737.34.

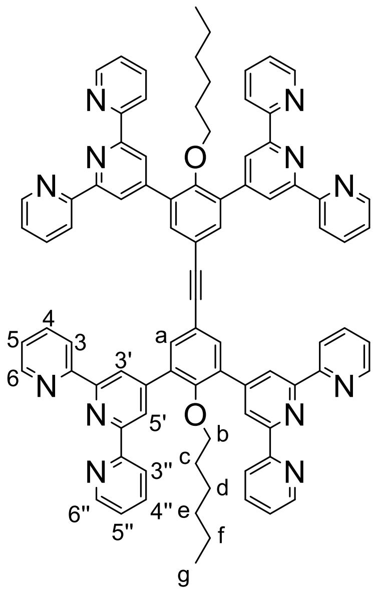


**Compound 14** Compound **12** (588 mg, 0.82 mmol) and compound **13** (719 mg, 0.98 mmol) were mixed in a 100 mL Schlenk flask, $Pd(PPh_3)_4$ (47.3 mg, 0.041 mmol) and CuI (6.2 mg, 0.033 mmol) were added. After degassing and backfill with nitrogen for three times, 30 mL of THF and 30 mL of $Et_3N$ were added under nitrogen atmosphere. After the addition of TBAF (0.98 mL, 0.98 mmol, 1 mol/L in THF), the mixture was stirred at 70 °C for 16h. After that the reaction was cooled down to room temperature and 100 mL of water was added. The mixture was extracted with chloroform, and the organic layer was collected, dried over anhydrous sodium sulfate and the solvent was evaporated under vacuum. The crude product was purified via column chromatography on silica gel ($CHCl_3$/MeOH = 100/2) to give compound **14** as a white solid (659 mg, 61.7% yield). $^1$H NMR (400 MHz, $CDCl_3$, 300K) δ 8.84 (s, 8H, tpy-$H^{3',5'}$), 8.74 (ddd, $J$ = 4.8, 1.8, 0.9 Hz, 8H, tpy-$H^{6,6''}$), 8.68 (dt, $J$ = 8.0, 1.0 Hz, 8H, tpy-$H^{3,3''}$), 7.89 (s, 4H, $H^a$), 7.89 – 7.83 (m, 8H, tpy-$H^{4,4''}$), 7.33 (ddd, $J$ = 7.5, 4.8, 1.2 Hz, 8H, tpy-$H^{5,5''}$), 3.41 (t, $J$ = 6.1 Hz, 4H, $H^b$), 1.28 – 1.15 (m, 4H, $H^c$), 0.93 (m, 4H, $H^d$), 0.82 – 0.63 (m, 8H, $H^e$ & $H^f$), 0.49 (t, $J$ = 6.7 Hz, 6H, $H^g$). $^{13}$C NMR (101 MHz, $CDCl_3$,

300K) δ 156.37, 156.36, 155.64, 155.08, 149.31, 147.81, 136.83, 134.48, 134.41, 132.23, 132.13, 132.04, 132.01, 128.65, 128.53, 123.79, 121.79, 121.33, 119.61, 89.11, 74.52, 31.42, 29.86, 25.52, 22.23, 13.88. ESI-TOF (*m/z*): Calcd. $[C_{76}H_{70}N_{12}O_2+H]^+$ for 1303.58, found 1303.57.

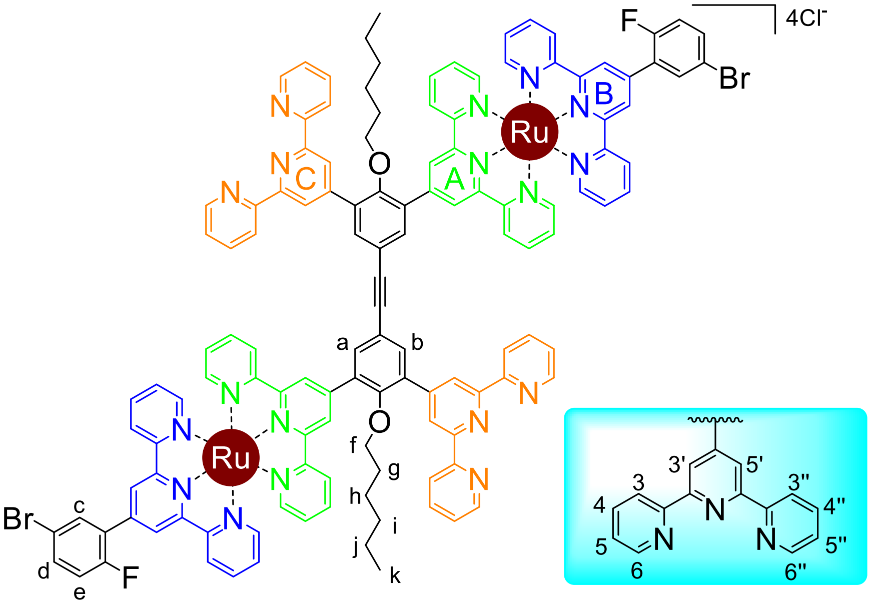


**Compound 15-trans** To a flask containing compound **14** (300 mg, 0.23 mmol) and compound **7** (281 mg, 0.46 mmol), 80 mL chloroform and 80 mL methanol was added. After the addition of N-ethylmorphline (500 uL), the mixture was stirred under reflux for 36h when it became a dark red solution. After cooling down to room temperature, the solvent was evaporated under vacuum, and the crude product was purified via column chromatography on aluminum oxide (DCM/MeOH = 100/1.5) to give compound **15** as a dark red solid (410 mg, 56.7% yield). $^1$H NMR (500 MHz, $CDCl_3$, 300K) δ 9.43 (s, 4H, tpyA-$H^{3',5'}$), 9.05 (s, 4H, tpyB-$H^{3',5'}$), 8.97 (d, *J* = 8.1 Hz, 4H, tpyC-$H^{6,6''}$), 8.90 (s, 4H, tpyC-$H^{3',5'}$), 8.77 (m, 12H, tpyA-$H^{3,3''}$, tpyB-$H^{3,3''}$ & tpyC-$H^{3,3''}$), 8.50 (dd, *J* = 6.8, 2.0 Hz, 2H, $H^c$), 7.97 (m, 14H, tpyA-$H^{4,4''}$, tpyB-$H^{4,4''}$, tpyC-$H^{4,4''}$& $H^b$), 7.65 (ddd, *J* = 8.4, 4.0, 2.4 Hz, 2H, $H^d$), 7.54 (d, *J* = 5.4 Hz, 4H, tpyA-$H^{6,6''}$), 7.44 – 7.36 (m, 8H, tpyA-$H^{5,5''}$, tpyC-$H^{5,5''}$), 7.32 (d, *J* = 5.3 Hz, 4H, tpyB-$H^{6,6''}$), 7.22 – 7.26 (m, 6H, tpyB-$H^{5,5''}$, $H^e$), 3.64 (t, *J* = 5.9

Hz, 4H, $H^f$), 1.41 – 1.31 (m, 4H, $H^g$), 1.13 – 1.03 (m, 4H, $H^i$), 0.89 – 0.73 (m, 8H, $H^i$ & $H^j$), 0.46 (t, $J$ = 7.2 Hz, 6H, $H^k$). $^{13}$C NMR (151 MHz, $CDCl_3$, 300K) δ 159.79, 158.12, 158.09, 157.91, 157.83, 157.57, 155.82, 155.72, 155.32, 155.25, 155.23, 154.97, 154.84, 154.76, 154.65, 152.58, 151.55, 151.42, 151.18, 151.06, 151.03, 147.15, 145.65, 142.62, 142.50, 138.72, 138.66, 138.53, 138.46, 134.67, 134.62, 131.73, 131.65, 131.62, 128.39, 128.16, 127.99, 127.90, 127.82, 125.62, 124.42, 124.28, 124.11, 123.73, 121.03, 120.81, 118.72, 118.48, 118.42, 118.23, 118.07, 89.61, 67.24, 31.22, 30.06, 25.81, 15.58, 13.37. $^{19}$F NMR (376 MHz, $CDCl_3$, 300K) δ -118.95. ESI-TOF (*m/z*): Calcd. $[C_{128}H_{96}Br_2Cl_4F_2N_{18}O_2\text{-}4Cl]^{4+}$ for 579.11, found 579.12. Note: Isomers does form during the reaction. Flash column chromatography on aluminum oxide (DCM/MeOH = 100/2) gave a mixture of cis and trans isomers. $^1$H NMR indicated that the trans- compound is the major product. By slowly adjusting the gradient from DCM/MeOH = 100/1 to DCM/MeOH = 100/1.5 we were able to isolate compound **15** as trans compound. (Supplementary Figure 3, 103). Due to the small amount, we were unable to isolate **15-cis**.

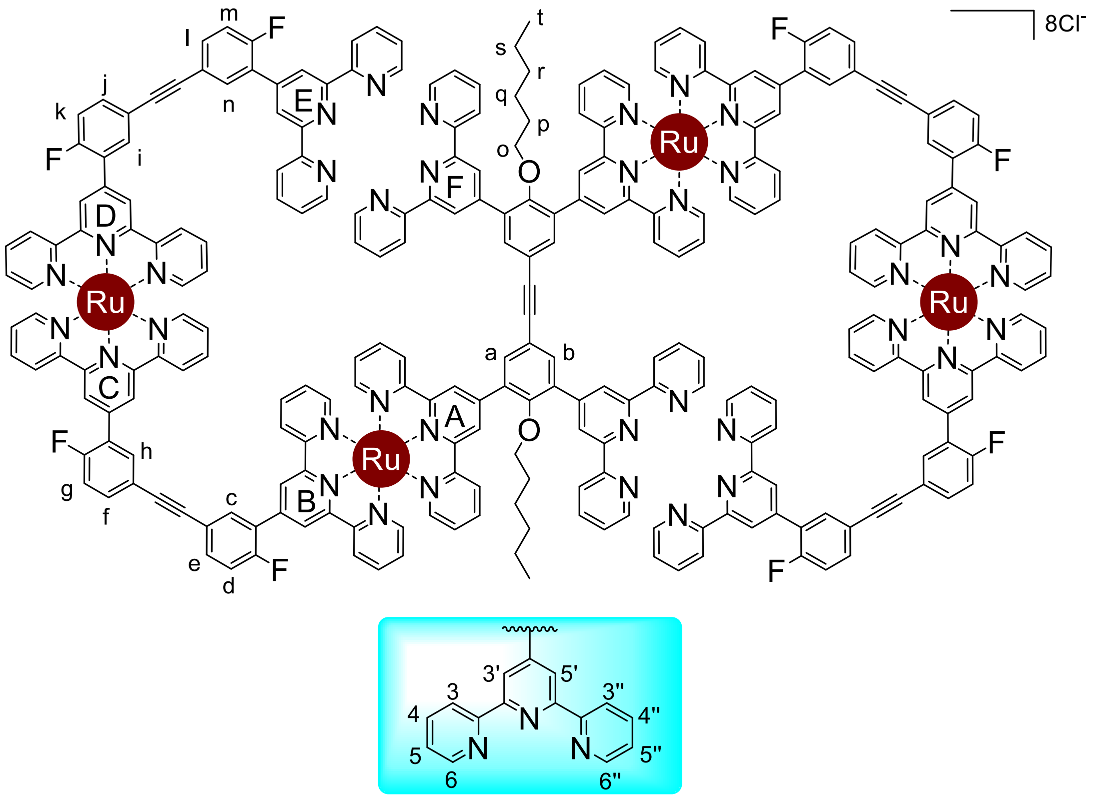

**Ligand L2** Compound **15** (80 mg, 0.032 mmol) and compound **9** (103.5 mg, 0.081 mmol) were mixed in a 100 mL Schlenk flask, $Pd(PPh_3)_4$ (3.8 mg, 0.0032 mmol) and CuI (0.46 mg, 0.0024 mmol) were added. After degassing and backfill with nitrogen for three times, 15 mL of DMF, 10 mL of DME and 30 mL of $Et_3N$ were added under nitrogen atmosphere. After the addition of TBAF (81 uL, 0.081 mmol, 1 mol/L in THF), the mixture was stirred at 80 °C for 16h. After that the reaction was cooled down to room temperature and the solvent was evaporated under vacuum. The crude product was purified via column chromatography on aluminum oxide ($CHCl_3$/MeOH = 100/2) to give **L2** as a dark red solid (58.9 mg, 39% yield). $^1H$ NMR (400 MHz, $CDCl_3$ with 10% $CD_3OD$, 300K) δ 9.30 (s, 4H, , tpyA-$H^{3',5'}$), 9.17 (s, 4H, tpyB-$H^{3',5'}$), 9.14 (s, 4H, tpyC-$H^{3',5'}$), 8.95 (s, 4H, tpyD-$H^{3',5'}$), 8.84 (m , 12H, tpyA-$H^{3,3''}$, tpyB-$H^{3,3''}$, tpyC-$H^{3,3''}$), 8.81 (s, 8H, tpyE-$H^{3',5'}$ & tpyF-$H^{3',5'}$ ), 8.74 (d, $J$ = 7.3 Hz, 4H, $H^c$, $H^h$ ), 8.71 – 8.51 (m, 32H, tpyE-$H^{3',5'}$, tpyE-$H^{3,3''}$, tpyE-$H^{6,6''}$, tpyD-$H^{3,3''}$, tpyC-$H^{3,3''}$, tpyF-$H^{3,3''}$, tpyF-$H^{6,6''}$, $H^a$, $H^b$), 8.27 (d, $J$ = 7.2 Hz, 2H, $H^i$), 8.06 –

7.81 (m, 26H, , tpyA,B,C,D,E,F-$H^{4,4''}$, $H^{n}$), 7.80 – 7.65 (m, 18H, tpyB,C,D-$H^{6,6''}$, $H^{e}$, $H^{f}$, $H^{j}$), 7.63 – 7.58 (m, 6H, tpyA-$H^{6,6''}$, $H^{i}$ ), 7.47 – 7.15 (m, 32H, tpyA,B,C,D,E,F-$H^{5,5''}$, $H^{d,g,k,m}$), 3.57 (s, 4H, $H^{o}$), 1.50 – 1.20 (m, 4H, $H^{p}$), 1.15– 0.93 (m, 4H, $H^{q}$), 0.91 – 0.49 (m, 8H, $H^{r,s}$), 0.35 (t, $J$ = 7.1 Hz, 6H, $H^{t}$). $^{13}$C NMR (126 MHz, $CDCl_3$ with 10% $CD_3OD$, 300K) δ 160.88, 158.85, 157.64, 155.87, 155.20, 155.18, 154.88, 154.88, 152.12, 151.91, 151.49, 149.11, 147.18, 147.12, 145.74, 144.77, 143.19, 138.73, 137.26, 135.63, 134.84, 134.35, 133.96, 131.87, 128.24, 127.91, 125.50, 125.23, 125.23, 124.81, 124.17, 124.17, 123.69, 123.68, 123.60, 121.70, 121.31, 120.92, 119.67, 117.15, 89.58, 88.95, 87.82, 75.24, 31.30, 29.70, 25.91, 22.22, 13.67. $^{19}$F NMR (376 MHz, $CDCl_3$ with 10% $CD_3OD$, 300K) δ -114.25, -114.55, -114.76. ESI-TOF (*m/z*): Calcd. $[C_{262}H_{174}F_8N_{36}Ru_4Cl_8O_2\text{-}8Cl]^{8+}$ for 551.88, found 551.88; Calcd. $[C_{262}H_{174}F_8N_{36}Ru_4Cl_8O_2\text{-}7Cl]^{7+}$ for 635.72, found 635.72; Calcd. $[C_{262}H_{174}F_8N_{36}Ru_4Cl_8O_2\text{-}6Cl]^{6+}$ for 747.50, found 747.50; Calcd. $[C_{262}H_{174}F_8N_{36}Ru_4Cl_8O_2\text{-}5Cl]^{5+}$ for 903.99, found 903.99; Calcd. $[C_{262}H_{174}F_8N_{36}Ru_4Cl_8O_2\text{-}4Cl]^{4+}$ for 1138.74, found 1138.74.

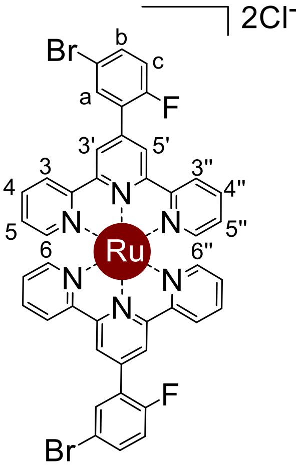

**Compound 16** To a flask containing compound **4** (500 mg, 1.45 mmol), $RuCl_2(DMSO)_4$ (336 mg, 0.7 mmol) was added. With the addition of 100 mL chloroform and 100 mL methanol, the mixture was stirred under reflux for 24h when it gradually become a uniform red solution. Afterwards the solvent was removed under vacuum, and the residue was purified via column chromatography on aluminum oxide ($CHCl_3$/MeOH = 100/1) to give compound **16** as a red solid (399 mg, 58% yield). $^1$H NMR (500 MHz, DMSO-$d_6$, 300K) δ 9.26 (s, 2H, tpy-$H^{3',5'}$), 8.93 (d, $J$ = 8.1 Hz, 2H, tpy-$H^{3,3''}$), 8.30 (dd, $J$ = 6.9, 2.6 Hz, 1H, $H^a$), 8.01 (td, $J$ = 7.8, 1.5 Hz, 2H, tpy-$H^{4,4''}$), 7.86 (ddd, $J$ = 8.8, 4.3, 2.5 Hz, 1H, $H^b$), 7.58 (dd, $J$ = 10.4, 8.8 Hz, 1H, $H^c$), 7.51 (d, $J$ = 5.7 Hz, 1H, tpy-$H^{6,6''}$), 7.27 (ddd, $J$ = 7.2, 5.5, 1.3 Hz, 1H, tpy-$H^{5,5''}$) $^{13}$C NMR (126 MHz, DMSO-$d_6$, 300K) δ 163.98, 161.99, 161.50, 159.12, 155.98, 146.57, 142.59, 139.06, 137.67, 137.56, 132.20, 130.53, 130.42, 129.08, 127.50, 122.56, 122.37, 122.00. $^{19}$F NMR (376 MHz, DMSO-$d_6$, 300K) δ -118.45. ESI-TOF ($m/z$): Calcd. $[C_{42}H_{26}Br_2Cl_2F_2N_6Ru\text{-}2Cl]^{2+}$ for 455.98, found 455.98.

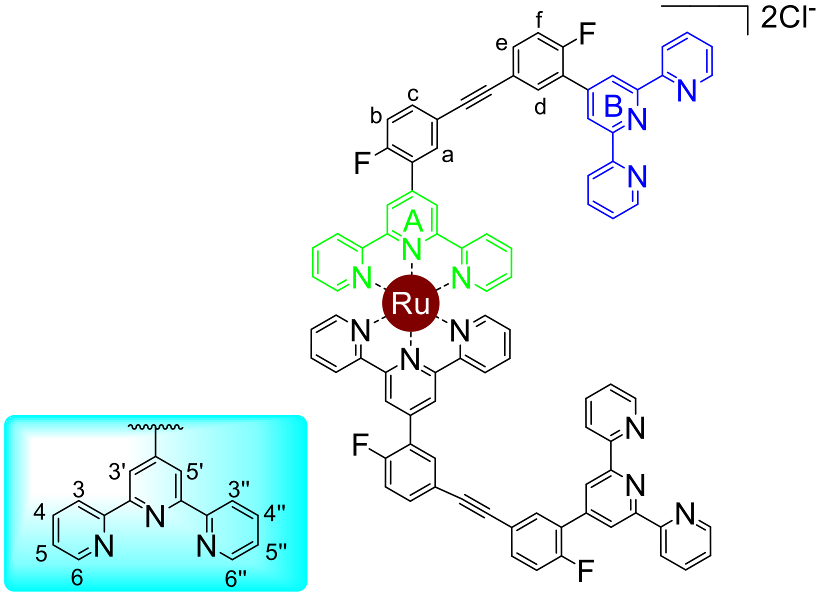


**Compound 17** Compound **16** (200 mg, 0.19 mmol) and compound **6** (200 mg, 0.57 mmol) were mixed in a 100 mL Schlenk flask, $Pd(PPh_3)_4$ (21.9 mg, 0.019 mmol) and CuI (3.6 mg, 0.019 mmol) were added. After degassing and backfill with nitrogen for three times, 30 mL of DMF, 30 mL of

DME and 30 mL of $Et_3N$ were added under nitrogen atmosphere. The mixture was stirred at 80 °C for 16h. After that the reaction was cooled down to room temperature and the solvent was evaporated under vacuum. The crude product was purified via column chromatography on aluminum oxide ($CHCl_3$/MeOH = 100/1) to give compound **17** as a dark red solid (175 mg, 58.6% yield). $^1$H NMR (400 MHz, DMSO-$d_6$, 300K) δ 9.41 (s, 2H, tpyA-$H^{3', 5'}$), 9.04 (d, $J$ = 7.9 Hz, 2H, tpyA-$H^{3, 3''}$), 8.77 (ddd, $J$ = 4.8, 1.8, 0.9 Hz, 2H, tpyB-$H^{6', 6''}$), 8.71 (dt, $J$ = 8.0, 1.1 Hz, 2H, tpyB-$H^{3, 3''}$), 8.69 (d, $J$ = 1.4 Hz, 1H, tpyB-$H^{3', 5'}$), 8.43 (dd, $J$ = 7.5, 2.1 Hz, 1H, $H^{a}$), 8.08 (dt, $J$ = 7.9, 1.7 Hz, 2H, tpyA-$H^{4, 4''}$), 8.07 (dt, $J$ = 7.7, 1.5 Hz, 2H, tpyB-$H^{4, 4''}$), 8.02 (m, 1H, $H^{d}$), 7.98 (ddd, $J$ = 8.6, 4.8, 2.1 Hz, 1H, $H^{c}$), 7.85 (ddd, $J$ = 8.6, 4.7, 2.2 Hz, 1H, $H^{e}$), 7.75 (dd, $J$ = 10.6, 8.6 Hz, 1H, $H^{b}$), 7.65 – 7.60 (m, 1H, $H^{f}$), 7.61 – 7.53 (m, 4H, tpyA-$H^{6, 6''}$ & tpyB-$H^{5, 5''}$), 7.32 (ddd, $J$ = 7.3, 5.6, 1.3 Hz, 2H, tpyB-$H^{5, 5''}$). $^{13}$C NMR (151 MHz, DMSO-$d_6$, 300K) δ 158.99, 158.90, 158.06, 155.95, 155.25, 154.95, 152.70, 149.85, 149.74, 144.31, 141.88, 140.55, 138.74, 138.58, 138.21, 138.06, 135.38, 134.04, 133.87, 128.37, 128.24, 127.11, 126.25, 126.16, 125.32, 125.15, 124.34, 123.94, 121.53, 121.45, 120.55, 119.79, 118.22, 118.07, 117.86, 88.94, 88.81. $^{19}$F NMR (376 MHz, DMSO-$d_6$, 300K) δ -114.97, -115.86. ESI-TOF ($m/z$): Calcd. $[C_{88}H_{52}Cl_2F_4N_{12}Ru\text{-}2Cl]^{2+}$ for 727.17, found 727.18.

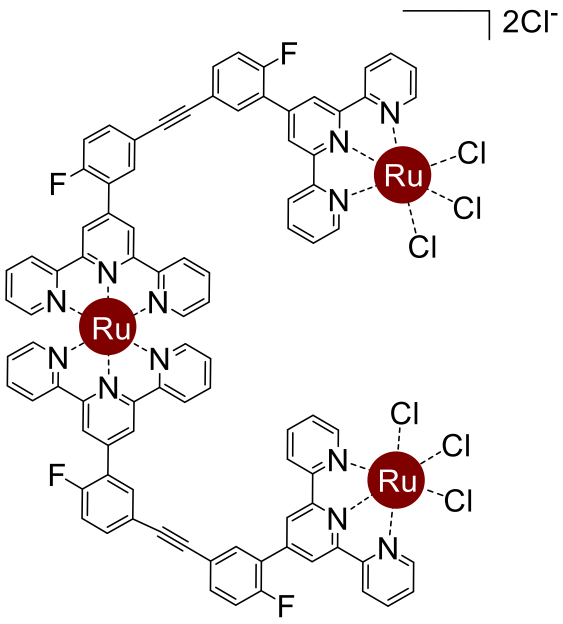


**Compound 18** Compound **18** was synthesized using the method similar to compound **7**. To a flask containing compound **17** (100 mg, 0.06 mmol), $RuCl_3.xH_2O$ (49.7 mg, 0.24 mmol) was added. With the addition of 100 mL chloroform and 100 mL methanol, the brown slurry was stirred under reflux for 24h. Afterwards the solvent was removed under vacuum, and the residue was washed with methanol for three times. The solid was collected and dried under vacuum to give compound **18** as a brown solid (2.8g, 83.6% yield). Due to the extremely poor solubility, compound **18** was directly used for the following steps without further characterization.

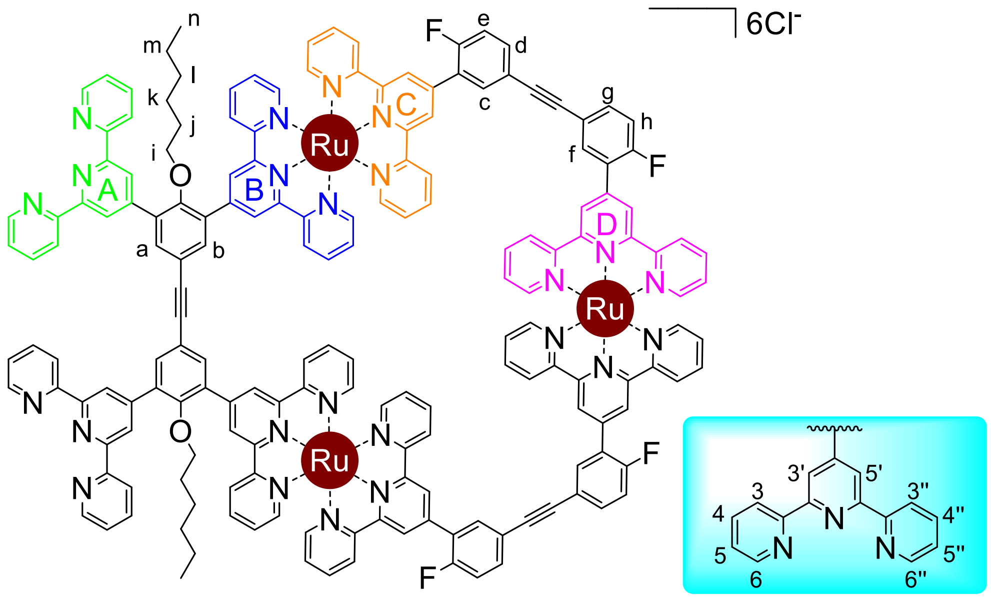


**Ligand L3'** To a flask containing compound **18** (60 mg, 0.031 mmol) and compound **14** (80 mg, 0.06 mmol), 100 mL chloroform and 100 mL methanol was added. After the addition of N-ethylmorphline (500 uL), the mixture was stirred under reflux for 36h when it became a dark red solution. After cooling down to room temperature, the solvent was evaporated under vacuum, and the crude product was purified via column chromatography on aluminum oxide (DCM/MeOH = 100/1) to give **L3'** as a dark red solid (26.5 mg, 27.3% yield). $^{1}$H NMR (500 MHz, DMSO-$d_6$) δ 9.54 (s, 4H, tpyB-$H^{3',5'}$), 9.51 (s, 8H, tpyC,D-$H^{3',5'}$), 9.21 – 9.08 (m, 12H, tpyB,C,D-$H^{3,3''}$), 8.95 (s, 4H, tpyA-$H^{3',5'}$), 8.85 – 8.75 (m, 8H, tpyA-$H^{3,3''}$, tpyA-$H^{6,6''}$), 8.58 – 8.48 (m, 4H, $H^{c.f}$), 8.38 – 8.33 (m, 2H, $H^{b}$), 8.28 (d, $J$ = 2.1 Hz, 2H, $H^{a}$), 8.19 – 7.97 (m, 20H, tpyA,B,C,D-$H^{4,4''}$, $H^{d,g}$), 7.83 – 7.77 (m, 4H, $H^{e,h}$), 7.67 – 7.51 (m, 16H, tpyB,C,D-$H^{6,6''}$, tpyA-$H^{5,5''}$), 7.28 – 7.38 (m, 12H, tpyB,C,D-$H^{5,5''}$), 3.74 (s, 4H, $H^{i}$), 1.38 (s, 4H, $H^{j}$), 1.26 – 1.08 (m, 4H, $H^{k}$), 0.93 – 0.74 (m, 4H, $H^{l}$), 0.62 (dq, $J$ = 14.9, 7.5, 6.5 Hz, 4H, $H^{m}$), 0.26 (t, $J$ = 7.3 Hz, 6H, $H^{n}$). $^{13}$C NMR (126 MHz, DMSO, 300K) δ 172.42, 160.98, 158.96, 158.31, 158.25, 158.18, 155.86, 155.82, 155.71, 155.40, 155.38, 155.36, 155.15, 155.03, 152.81, 152.42, 149.85, 147.11, 144.97, 142.01, 141.94, 138.73, 138.10, 135.59, 135.35, 135.07, 134.75, 134.61, 134.02, 128.35, 126.47, 126.38, 126.29, 125.49,

125.19, 125.09, 124.28, 121.50, 121.32, 119.96, 119.68, 118.31, 118.12, 89.77, 89.18, 79.71, 77.05, 75.04, 74.53, 56.13, 31.37, 29.61, 25.42, 13.86. $^{19}F$ NMR (376 MHz, DMSO-$d_6$) δ -112.87. ESI-TOF (*m/z*): Calcd. $[C_{174}H_{122}Cl_6F_4N_{24}Ru_3O_2\text{-}6Cl]^{6+}$ for 493.45, found 493.45; Calcd. $[C_{174}H_{122}Cl_6F_4N_{24}Ru_3O_2\text{-}5Cl]^{5+}$ for 599.14, found 599.14; Calcd. $[C_{174}H_{122}Cl_6F_4N_{24}Ru_3O_2\text{-}4Cl]^{4+}$ for 757.67. found 757.67; Calcd. $[C_{174}H_{122}Cl_6F_4N_{24}Ru_3O_2\text{-}3Cl]^{3+}$ for 1021.87, found 1021.88.

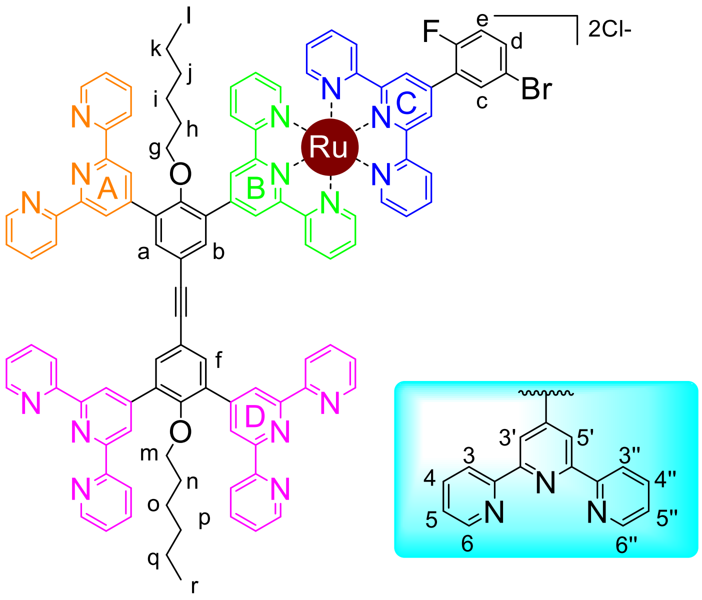


**Compound 19** To a flask containing compound **14** (650 mg, 0.5 mmol) and compound **7** (152 mg, 0.25 mmol), 150 mL chloroform and 150 mL methanol was added. After the addition of N-ethylmorphline (500 uL), the mixture was stirred under reflux for 36h when it became a dark red solution. After cooling down to room temperature, the solvent was evaporated under vacuum, and the crude product was purified via column chromatography on aluminum oxide (DCM/MeOH = 100/1) to give compound **19** as a dark red solid (258 mg, 55.7% yield). $^{1}H$ NMR (500 MHz, $CDCl_3$, 300K) δ 9.08 (s, 2H, tpyB-$H^{3',5'}$), 9.03 (s, 2H, tpyC-$H^{3',5'}$), 8.84 (s, 2H, tpyA-$H^{3',5'}$), 8.77 (d, $J$ = 11.2 Hz, 2H, tpyB-$H^{3,3''}$), 8.75 (s, 4H, tpyD-$H^{3',5'}$), 8.73 – 8.63 (m, 18H, tpyA-$H^{3,3''}$, tpyC-$H^{3,3''}$,

tpyD-$H^{3,3''}$, tpyA-$H^{6,6''}$, tpyD-$H^{6,6''}$), 8.46 (dd, $J$ = 7.0, 2.5 Hz, 1H, $H^{c}$), 8.20 (d, $J$ = 2.1 Hz, 1H, $H^{b}$), 8.03 – 7.93 (m, 5H, tpyB-$H^{4,4''}$, tpyC-$H^{4,4''}$, $H^{a}$), 7.92 – 7.81 (m, 8H, tpyA-$H^{4,4''}$, tpyD-$H^{4,4''}$, $H^{f}$), 7.62 (ddd, $J$ = 8.8, 4.4, 2.5 Hz, 1H, $H^{d}$), 7.55 (d, $J$ = 5.5 Hz, 2H, tpyB-$H^{6,6''}$), 7.37-7.30 (m, 10H, tpyA-$H^{5,5''}$, tpyB-$H^{5,5''}$, tpyD-$H^{5,5''}$, tpyC-$H^{6,6''}$), 7.24 – 7.15 (m, 3H, tpyC-$H^{5,5''}$ & $H^{e}$), 3.61 (t, $J$ = 6.0 Hz, 2H, $H^{g}$), 3.37 (t, $J$ = 6.1 Hz, 2H, $H^{m}$), 1.30 (m, 2H, $H^{h}$), 1.21 (m, 2H, $H^{n}$), 1.03 (p, $J$ = 7.4 Hz, 2H, $H^{i}$), 0.93 – 0.61 (m, 10H, $H^{j}$, $H^{k}$, $H^{o}$, $H^{p}$, $H^{q}$), 0.47 – 0.42 (m, 3H, $H^{l}$), 0.39 (t, $J$ = 7.1 Hz, 3H, $H^{r}$). $^{13}$C NMR (126 MHz, $CDCl_3$, 300K) δ 160.02, 158.02, 157.74, 157.44, 155.92, 155.73, 155.61, 155.53, 154.95, 154.83, 154.73, 152.39, 151.38, 149.11, 147.28, 146.95, 145.41, 142.45, 138.81, 138.56, 137.18, 137.00, 134.89, 134.63, 134.58, 134.34, 134.23, 131.56, 130.87, 128.78, 128.54, 128.14, 126.33, 126.23, 126.03, 125.02, 124.13, 124.09, 123.97, 121.50, 121.42, 121.34, 121.29, 120.57, 119.40, 118.31, 118.24, 118.21, 118.12, 90.16, 88.86, 75.28, 74.55, 31.32, 31.27, 30.13, 29.67, 25.91, 25.39, 22.20, 13.76, 13.71. $^{19}$F NMR (376 MHz, $CD_3CN$, 300K) δ -118.92. ESI-TOF ($m/z$): Calcd. $[C_{107}H_{83}BrCl_2FN_{15}RuO_2\text{-}2Cl]^{2+}$ for 904.75, found 904.74.

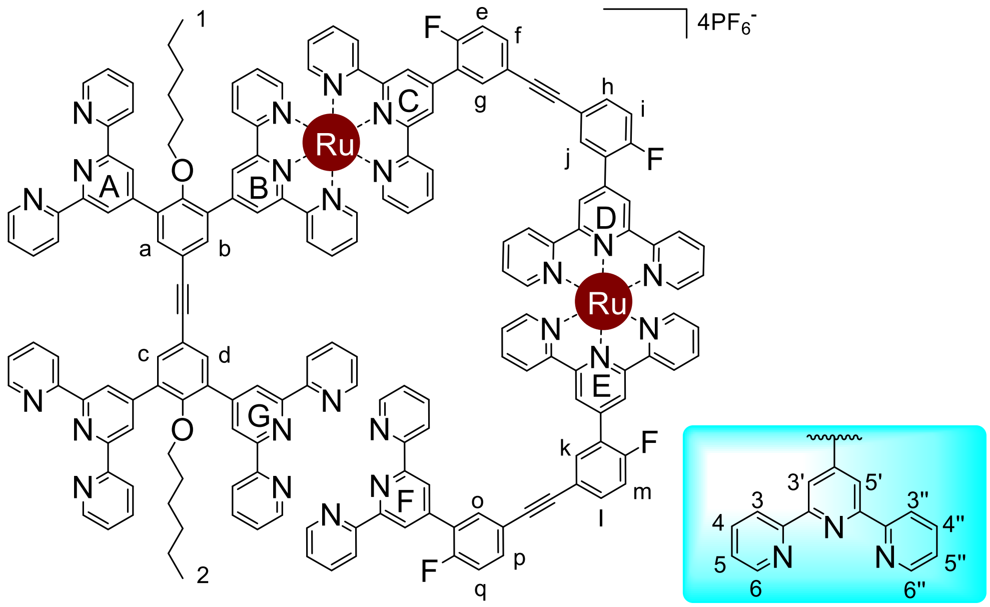

**Ligand L3** Compound **19** (91.8 mg, 0.049 mmol) and compound **9** (80.7 mg, 0.064 mmol) were mixed in a 100 mL Schlenk flask, $Pd(PPh_3)_4$ (2.9 mg, 2.5 μmol) and CuI (0.38 mg, 2.0 μmol) were added. After degassing and backfill with nitrogen for three times, 15 mL of DMF, 10 mL of DME and 30 mL of $Et_3N$ were added under nitrogen atmosphere. Upon addition of TBAF (65 μL, 0.065 mmol, 1.0 mol/L in THF), the mixture was stirred at 80 °C for 16h. After that the reaction was cooled down to room temperature and the solvent was evaporated under vacuum. The crude product was purified via column chromatography on aluminum oxide ($CHCl_3$/MeOH = 100/1.8) to give compound **L3** with $Cl^-$ as the counterion as a dark red solid. The solid was dissolved in a mixed solvent of 10 mL of chloroform and 80 mL of methanol, and 200 mg of $NH_4PF_6$ was added. The mixture was stirred at room temperature for 30 min, the red precipitate formed was collected via filtration, washed with methanol and dried in vacuum to give **L3** with $PF_6^-$ as the counterion (54.1 mg, 28.5% yield). $^1$H NMR (500 MHz, $CD_3CN$, 300K) δ 9.08 (s, 2H, tpyB-$H^{3',5'}$), 9.02 (s, 2H, tpyE-$H^{3',5'}$), 9.01 (s, 4H, tpyC,D-$H^{3',5'}$), 8.93 (s, 4H, tpyA-$H^{3',5'}$), 8.80 (s, 2H, tpyH,G-$H^{3',5'}$), 8.78 – 8.69 (m, 18H, tpyF-$H^{3',5'}$, tpyA,F,G,H-$H^{3,3''}$, tpyA,F,G,H-$H^{6,6''}$), 8.65 – 8.59 (m, 6H, tpyC,D,E-$H^{3,3''}$), 8.56 (d, *J* = 8.1 Hz, 2H, tpyB-$H^{3,3''}$), 8.38 – 8.30 (m, 2H, $H^{g,j}$), 8.27 (dd, *J* = 7.4, 2.2 Hz, 1H, $H^k$), 8.23 – 8.21 (m, 1H, $H^b$), 8.10 – 7.88 (m, 23H, tpyA,B,C,D,E,F,G,H-$H^{6,6''}$, $H^{a,c,d,n,h,l,f}$), 7.78 (ddd, *J* = 8.6, 4.7, 2.2 Hz, 1H, $H^o$), 7.68 – 7.55 (m, 3H, $H^{e,i,m}$ ), 7.54 – 7.39 (m, 16H, tpyB,C,D,E-$H^{6,6''}$, tpyA,F,G,H-$H^{5,5''}$), 7.30 – 7.15 (m, 8H, tpyB,C,D,E-$H^{5,5''}$), 3.65 (t, *J* = 5.9 Hz, 2H, alkyl chain 1- $H^{-OCH_2-}$ ), 3.35 (t, *J* = 5.9 Hz, 2H, alkyl chain 2- $H^{-OCH_2-}$), 1.47 – 1.31 (m, 2H, alkyl chain 1- $H^{-CH_2-}$), 1.23 – 1.12 (m, *J* = 7.3 Hz, 2H, alkyl chain 2- $H^{-CH_2-}$), 1.08 (q, *J* = 7.7 Hz, 2H, alkyl chain 1- $H^{-CH_2-}$), 0.85 – 0.78 (m, 4H, alkyl chain 1,2- $H^{-CH_2-}$ ), 0.75 – 0.61 (m, 6H, alkyl chain 1,2- $H^{-CH_2-}$), 0.43 (t, *J* = 6.7 Hz, 2H, alkyl chain 1- $H^{-CH_3}$), 0.37 (t, *J* = 7.2 Hz, 2H, alkyl chain 2- $H^{-CH_3}$). $^{13}$C NMR (101 MHz, $CD_3CN$, 300K) δ 159.22, 158.68, 158.13, 158.03, 157.89,

157.12, 156.46, 155.85, 155.85, 155.61, 155.41, 155.38, 155.20, 155.05, 155.04, 154.54, 153.43, 152.62, 152.42, 151.67, 151.13, 150.81, 149.35, 148.58, 147.08, 144.38, 143.19, 142.80, 142.31, 142.12, 140.54, 140.29, 138.25, 137.30, 135.32, 134.46, 134.07, 127.60, 124.40, 123.55, 121.21, 121.11, 120.98, 120.46, 97.02, 94.96, 75.06, 74.47, 31.16, 30.93, 30.93, 29.80, 29.49, 29.49, 25.82, 25.28, 25.27, 22.05, 21.90, 21.90, 13.04. $^{19}F$ NMR (376 MHz, $CD_3CN$, 300K) δ -115.18, -115.63, -115.92. ESI-TOF (*m/z*): Calcd. $[C_{174}H_{122}P_4F_{28}N_{24}Ru_2O_2\text{-}4PF_6]^{4+}$ for 714.71, found 714.71; Calcd. $[C_{174}H_{122}P_4F_{28}N_{24}Ru_2O_2\text{-}3PF_6]^{3+}$ for 1001.26, found 1001.26; Calcd. $[C_{174}H_{122}P_4F_{28}N_{24}Ru_2O_2\text{-}2PF_6]^{2+}$ for 1574.37, found 1574.37.

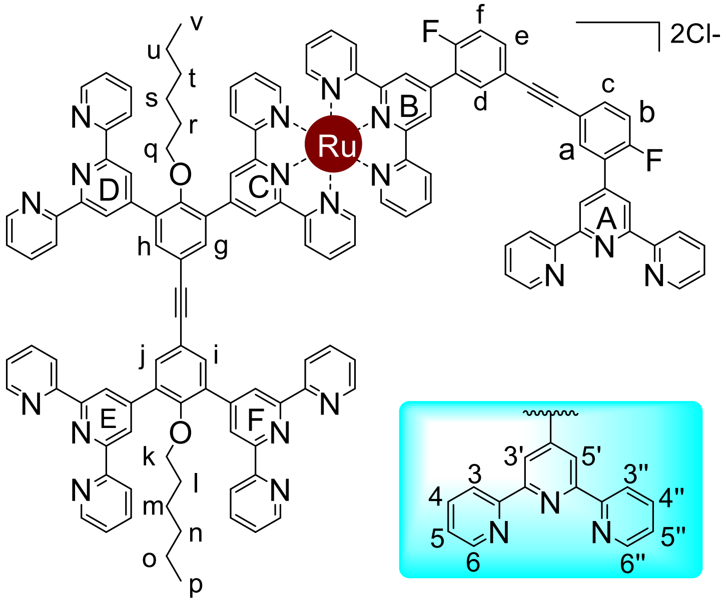

**Ligand L4** Compound **19** (400 mg, 0.21 mmol) and compound **6** (149.3 mg, 0.42 mmol) were mixed in a 100 mL Schlenk flask, $Pd(PPh_3)_4$ (13 mg, 0.011 mmol) and CuI (5 mg, 0.011 mmol) were added. After degassing and backfill with nitrogen for three times, 15 mL of DMF, 10 mL of DME and 30 mL of $Et_3N$ were added under nitrogen atmosphere. The mixture was stirred at 80 °C for 16h. After that the reaction was cooled down to room temperature and the solvent was evaporated under vacuum. The crude product was purified via column chromatography on aluminum oxide ($CHCl_3$/MeOH = 100/1) to give ligand **L4** as a dark red solid (259 mg, 57.5 %

yield). $^1$H NMR (500 MHz, $CDCl_3$, 300K) δ 8.99 (s, 2H, tpyB-$H^{3',5'}$ ), 8.98 (s, 2H, tpyC-$H^{3',5'}$), 8.87 (s, 2H, tpyD-$H^{3',5'}$), 8.80 – 8.74 (m, 2H, tpyB-$H^{3,3''}$), 8.77-8.73 (m, 4H, tpyD-$H^{3,3''}$ & tpyD-$H^{6,6''}$), 8.72 (s, 4H, tpyE(F)-$H^{3',5'}$), 8.71 – 8.50 (m, 15H, , tpyE(F)-$H^{6,6''}$, tpyA-$H^{3',5'}$, tpyA, C, E(F)-$H^{3,3''}$, tpyA-$H^{6,6''}$, $H^g$), 8.22 (d, $J$ = 2.0 Hz, 1H, $H^g$), 8.04 – 7.79 (m, 17H, tpyA, B, C, D, E(F)-$H^{4,4''}$, $H^h$, $H^a$, $H^i$, $H^j$), 7.76 – 7.69 (m, 2H, $H^c$, $H^e$), 7.58 (d, $J$ = 5.5 Hz, 2H, tpyB-$H^{6,6''}$), 7.46 (m, 4H, tpyB-$H^{5,5''}$ & tpyA-$H^{5,5''}$), 7.44 – 7.26 (m, 13H, tpyC-$H^{6,6''}$, tpyD-$H^{5,5''}$, tpyE(F)-$H^{5,5''}$, tpyC-$H^{5,5''}$, $H^f$), 7.14 (dd, $J$ = 10.1, 8.6 Hz, 1H, $H^b$), 3.55 (t, $J$ = 5.9 Hz, 2H, $H^q$), 3.42 (t, $J$ = 6.1 Hz, 2H, $H^k$), 1.24 (m, 4H, $H^r$ & $H^l$), 0.95 (m, 2H, $H^m$ & $H^s$), 0.80 – 0.59 (m, 8H, $H^o$, $H^n$, $H^u$ & $H^t$), 0.52 – 0.42 (t, $J$ = 7.2 Hz, 3H, $H^t$), 0.37 (t, $J$ = 7.1 Hz, 1H, $H^p$). $^{13}$C NMR (126 MHz, $CDCl_3$, 300K) δ 160.67, 158.64, 157.68, 157.30, 155.93, 155.75, 155.66, 155.52, 154.91, 154.80, 154.61, 152.60, 151.52, 149.10, 147.37, 146.99, 145.11, 144.51, 142.58, 138.68, 138.41, 137.10, 137.03, 135.60, 135.12, 134.85, 134.63, 134.44, 134.19, 133.28, 131.37, 128.63, 128.19, 126.87, 126.76, 125.86, 124.87, 124.15, 123.98, 123.87, 123.73, 121.46, 121.35, 120.84, 120.58, 119.78, 119.44, 117.40, 117.21, 116.90, 116.72, 90.22, 89.27, 88.89, 88.26, 75.29, 74.62, 31.31, 30.12, 29.68, 25.93, 25.42, 22.19, 22.12, 13.78, 13.73. $^{19}$F NMR (376 MHz, $CDCl_3$, 300K) δ -114.32, -114.41. ESI-TOF (*m/z*): Calcd. $[C_{130}H_{96}F_2N_{18}RuCl_2O_2\text{-}2Cl]^{2+}$ for 1040.35, found 1040.35.

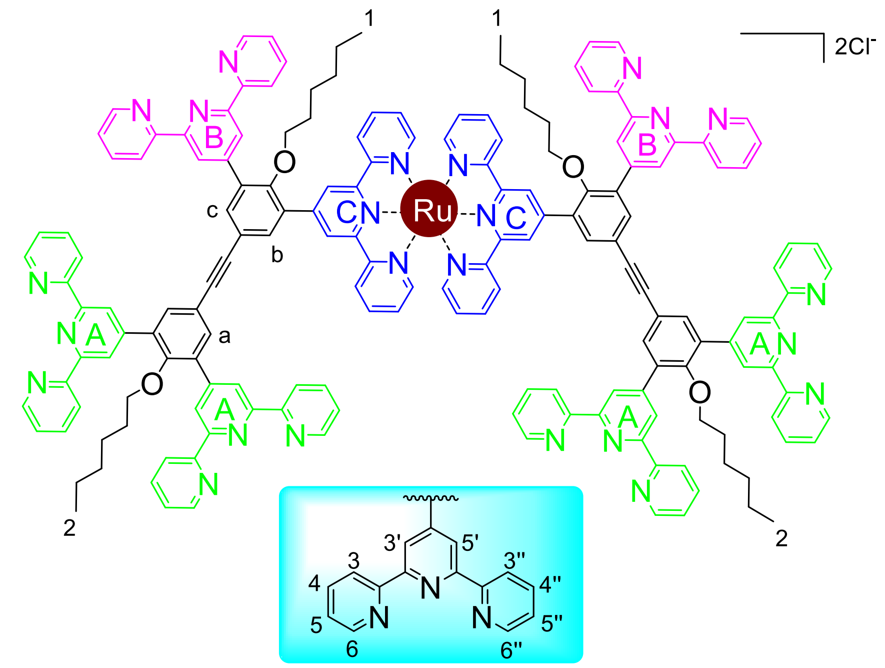


**Compound 20** To a flask containing compound **14** (300 mg, 0.23 mmol), $RuCl_2(DMSO)_4$ (27.9 mg, 0.057 mmol) was added. With the addition of 150 mL chloroform and 150 mL methanol, the mixture was stirred under reflux for 24h when it gradually became a uniform red solution. Afterwards the solvent was removed under vacuum, and the residue was purified via column chromatography on aluminum oxide ($CHCl_3$/MeOH = 100/1) to give compound **20** as a red solid (88.6 mg, 56% yield). $^1$H NMR (500 MHz, $CDCl_3$) δ 9.13 (s, 4H, tpyC-$H^{3',5'}$), 8.83 (s, 4H, tpyB-$H^{3',5'}$), 8.75 (s, 8H, tpyA-$H^{3',5'}$), 8.72 – 8.66 (m, 20H, tpyC, B-$H^{3,3''}$, tpyA,B-$H^{6,6''}$), 8.63 (d, $J$ = 8.0 Hz, 8H, tpyA-$H^{3,3''}$). 8.19 (d, $J$ = 2.0 Hz, 2H, $H^b$), 8.06 – 7.96 (m, 6H, $H^c$, tpyC-$H^{4,4''}$), 7.93 – 7.83 (m, 16H, $H^a$, tpyA,B-$H^{4,4''}$), 7.42 (dd, $J$ = 5.6, 1.4 Hz, 4H, tpyC-$H^{6,6''}$), 7.40 – 7.27 (m, 16H, tpyC,B,A-$H^{5,5''}$), 3.62 (t, $J$ = 6.0 Hz, 4H, alkyl chain 1-$H^{-OCH_2-}$), 3.37 (t, $J$ = 6.0 Hz, 4H, alkyl chain 2-$H^{-OCH_2-}$), 1.36 – 1.28 (m, 4H, , alkyl chain 1-$H^{-CH_2-}$), 1.19 – 1.11 (m, 4H, alkyl chain 2-$H^{-CH_2-}$), 1.08 – 0.99 (m, 4H, alkyl chain 2-$H^{-CH_2-}$), 0.87 (t, $J$ = 7.7 Hz, 4H, alkyl chain 1-$H^{-CH_2-}$), 0.78 (p, $J$ = 7.1 Hz, 4H, alkyl chain 2-$H^{-CH_2-}$), 0.74 – 0.61 (m, $J$ = 9.4, 7.5, 5.3 Hz, 12H, alkyl chain 1,2-$H^{-CH_2-}$), 0.45 – 0.42 (m, 3H, alkyl chain 1-$H^{CH_3}$), 0.38 (t, $J$ = 7.2 Hz, 3H, alkyl chain 2-$H^{CH_3}$). $^{13}$C

NMR (126 MHz, $CDCl_3$, 300K) δ 160.45, 157.91, 157.81, 157.64, 157.48, 156.09, 156.01, 155.84, 155.56, 154.98, 154.88, 152.47, 151.73, 151.23, 148.30, 146.23, 145.52, 143.40, 138.91, 138.72, 137.54, 137.31, 137.01, 136.55, 135.23, 134.80, 133.58, 131.21, 129.30, 128.53, 128.04, 127.43, 125.70, 125.45, 125.03, 124.31, 123.54, 122.41, 121.23, 114.45, 93.45, 91.53, 89.02, 79.45, 72.55, 31.30, 30.17, 30.02, 25.88, 25.41, 24.21, 23.94, 12.28. ESI-TOF (*m/z*): Calcd. $[C_{172}H_{140}N_{24}RuCl_2O_4\text{-}2Cl]^{2+}$ for 1353.53, found 1353.50.

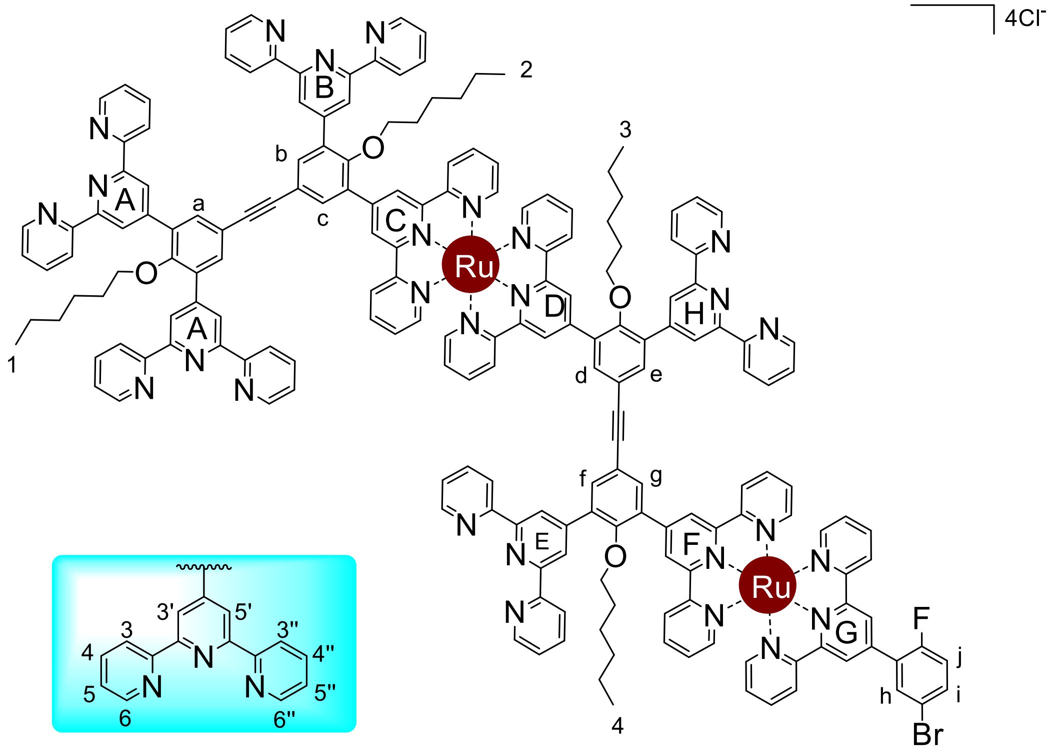


**Compound 21-trans** Compound **20** (400 mg, 0.144 mmol) and compound **7** (82 mg, 0.048 mmol) were mixed in a 200 mL round bottom flask, 100 mL of methanol and 100 mL of chloroform was added. After the addition of N-ethylmorphline (500 uL), the mixture was stirred under reflux for 24h. After that the reaction was cooled down to room temperature and the solvent was evaporated under vacuum. The crude product was purified via column chromatography on aluminum oxide

($CHCl_3$/MeOH = 100/1.5) to give compound **20** as a dark red solid (83.7 mg, 52% yield). $^1$H NMR (400 MHz, $CDCl_3$) δ 9.49 (s, 4H, tpyC-$H^{3',5'}$ & tpyD-$H^{3',5'}$), 9.16 (s, 2H, tpyG-$H^{3',5'}$), 9.14 (s, 2H, tpyF-$H^{3',5'}$), 9.11 – 9.04 (m, 6H, tpyC-$H^{3,3''}$, tpyD-$H^{3,3''}$ & ), 8.93 – 8.88 (m, 9H, tpyB-$H^{3',5'}$, tpyE-$H^{3',5'}$, tpyH-$H^{3',5'}$,tpyF-$H^{3,3''}$), 8.82 – 8.65 (m, 26H, tpyA-$H^{3',5'}$, tpyA,B,E,H,G-$H^{3,3''}$, tpyA,B,E,H-$H^{6,6''}$, ), 8.58 (dd, $J$ = 7.0, 2.4 Hz, 1H, $H^h$), 8.31 (s, 1H, $H^g$), 7.80-8.05 (m, 25H, tpyA,B,C,D,E,F,G,H-$H^{4,4''}$, $H^{a,b,c,d,e,f}$), 7.60 – 7.51 (m, 1H, $H^i$), 7.62 – 7.27 (m, 26H, tpyC,D,F,G-$H^{6,6''}$, tpyA,B,C,D,E,F,G,H-$H^{5,5''}$), 7.07 (t, $J$ = 9.5 Hz, 1H, $H^j$ ), 3.72 – 3.57 (m, 6H, alkyl chain 2,3,4 – $H^{-OCH_2-}$), 3.40 (t, $J$ = 6.1 Hz, 2H, alkyl chain 1 – $H^{-OCH_2-}$), 1.40 – 1.29 (m, 6H, alkyl chain 2,3,4 – $H^{-CH_2-}$), 1.23 – 1.15 (m, 2H, alkyl chain 1 – $H^{-CH_2-}$), 1.14 – 1.10 (m, 6H, alkyl chain 2,3,4 – $H^{-CH_2-}$), 0.91 – 0.65 (m, 18H, alkyl chain 1,2,3,4 – $H^{-CH_2-}$), 0.51 – 0.40 (m, 9H, alkyl chain 2,3,4 – $H^{-CH_3}$)0.38 (t, $J$ = 7.1 Hz, 3H, alkyl chain 1 – $H^{-CH_3}$). $^{13}$C NMR (126 MHz, $CDCl_3$, 300K) δ 160.45, 157.91, 157.81, 157.64, 157.48, 156.09, 156.01, 155.84, 155.56, 154.98, 154.88, 152.47, 151.98, 151.73, 151.23, 149.23, 147.43, 147.28, 147.20, 145.73, 142.46, 138.83, 138.72, 138.63, 137.23, 137.01, 136.88, 135.93, 134.81, 134.42, 134.25, 131.30, 128.71, 128.28, 128.02, 125.98, 125.70, 125.53, 125.12, 124.28, 124.06, 123.87, 121.40, 121.09, 119.26, 118.23, 90.16, 88.98, 75.32, 74.56, 31.32, 30.17, 29.69, 25.90, 25.41, 22.24, 22.12, 13.76. $^{19}$F NMR (376 MHz, $CDCl_3$, 300K) δ -118.95. ESI-TOF (*m/z*): Calcd. $[C_{193}H_{153}N_{27}Ru_2BrFCl_4O_4\text{-}2Cl]^{2+}$ for 1596.96, found 1596.92. Note that isomers do form during the reaction. flash column chromatography on aluminum oxide (DCM/MeOH = 100/2) gave a mixture of cis and trans isomers. $^1$H NMR indicated that the trans compound is the major product. By slowly adjusting the gradient from DCM/MeOH = 100/1 to DCM/MeOH = 100/1.5 we were able to isolate compound **21** as trans compound. (Supplementary Figure 6, 104). Due to the small amount, we were unable to isolate **21-cis**.

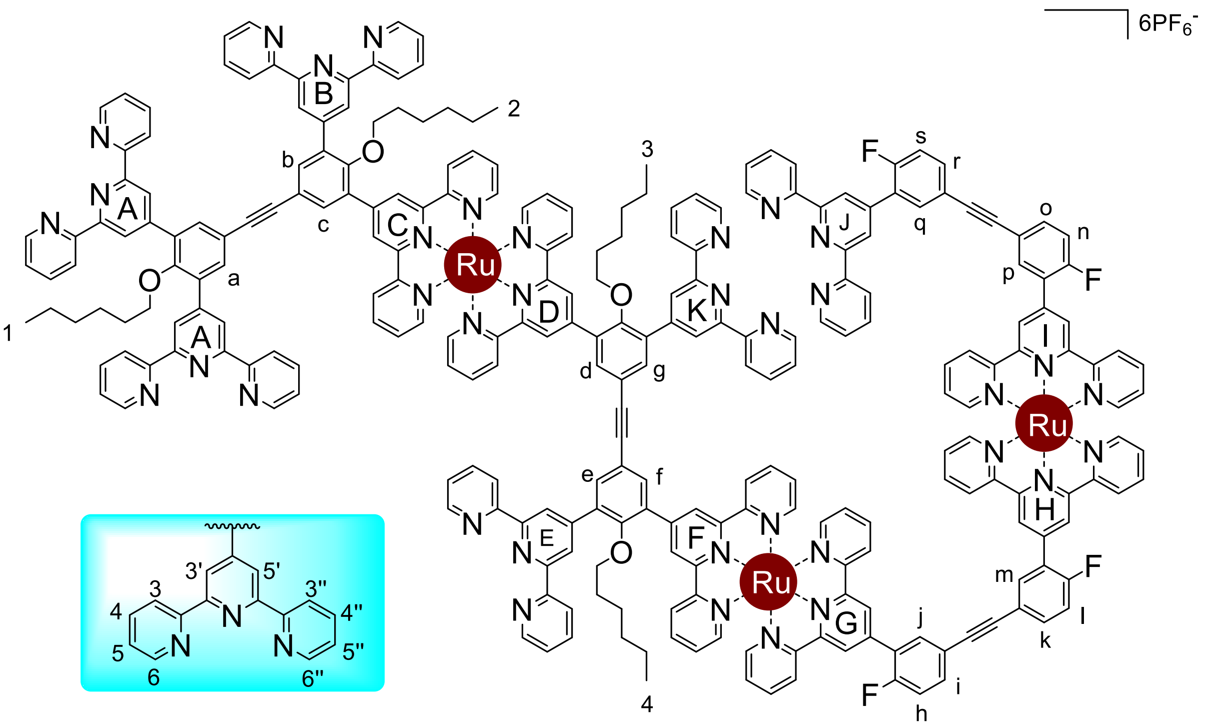


**Ligand L5** Compound **21** (100 mg, 0.03 mmol) and compound **9** (82 mg, 0.064 mmol) were mixed in a 100 mL Schlenk flask, $Pd(PPh_3)_4$ (1.9 mg, 0.0016 mmol) and CuI (0.23 mg, 0.0012 mmol) were added. After degassing and backfill with nitrogen for three times, 15 mL of DMF, 10 mL of DME and 30 mL of $Et_3N$ were added under nitrogen atmosphere. With the addition of TBAF (70 uL, 0.07 mmol), the mixture was stirred at 80 °C for 16h. After that the reaction was cooled down to room temperature and the solvent was evaporated under vacuum. The crude product was purified via column chromatography on aluminum oxide ($CHCl_3$/MeOH = 100/2) to give compound **17** as a dark red solid. The solid was dissolved in a mixed solvent of 10 mL of chloroform and 80 mL of methanol, and 200 mg of $NH_4PF_6$ was added. The mixture was stirred at room temperature for 30 min, the red precipitate formed was collected via filtration, washed with methanol and dried in vacuum to give **L5** with $PF_6^-$ as the counterion (103.2 mg, 67% yield). $^1$H NMR (600 MHz, $CD_3CN$) δ 9.16 (s, 2H, tpyE-$H^{3',5'}$), 9.15 (s, 2H, tpyD-$H^{3',5'}$), 9.13 (s, 2H, tpyG-$H^{3',5'}$), 9.04 (s, 2H, tpyH-$H^{3',5'}$), 9.01 (s, 2H, tpyI-$H^{3',5'}$), 8.99 (s, 2H, tpyJ-$H^{3',5'}$), 8.98 (s, 4H,

tpyC,L-$H^{3',5'}$), 8.94 (s, 2H, tpyF-$H^{3',5'}$), 8.82 (s, 4H, tpyA(B)-$H^{3',5'}$), 8.81– 8.79 (m, 10H, tpyC,L-$H^{6,6''}$, , tpyC,L-$H^{3,3''}$, tpyK-$H^{3',5'}$), 8.79 – 8.70 (m, 12H, tpyK-$H^{6,6''}$, tpyJ-$H^{6,6''}$, tpyF-$H^{6,6''}$, tpyF-$H^{3,3''}$, tpyA(B)-$H^{3,3''}$), 8.68 – 8.58 (m, 14H, tpyD,E,G,H,I,J,K-$H^{3,3''}$), 8.33 (ddd, *J* = 7.6, 5.8, 2.2 Hz, 2H, $H^k$, $H^n$), 8.31 (dd, *J* = 5.0, 2.1 Hz, 2H, $H^e$, $H^d$), 8.25 (dd, *J* = 7.4, 2.2 Hz, 1H, $H^q$), 8.23 (d, *J* = 2.1 Hz, 1H, $H^g$), 8.15 (t, *J* = 2.3 Hz, 2H, $H^c$, $H^h$), 8.05 – 8.00 (m, 5H, tpyC,L-$H^{4,4''}$, $H^f$), 8.00 – 7.91 (m, 25H, tpyA,B,D,E,F,G,H,I,J,K-$H^{4,4''}$, $H^{a,b,t,j,m}$), 7.89 (ddd, *J* = 8.6, 4.7, 2.1 Hz, 1H, $H^p$), 7.75 (ddd, *J* = 8.6, 4.8, 2.2 Hz, 1H, $H^s$), 7.63 (ddd, *J* = 10.2, 8.7, 1.3 Hz, 2H, $H^{i,j}$), 7.57 (dd, *J* = 10.5, 8.6 Hz, 1H, $H^o$), 7.54 – 7.43 (m, 22H, tpyA,B,C,F,K-$H^{5,5''}$, tpyD,E,G,H,I,J-$H^{6,6''}$), 7.41 (dd, *J* = 10.7, 8.6 Hz, 1H, $H^r$), 7.29 – 7.19 (m, 12H, tpyD,E,G,H,I,J-$H^{5,5''}$), 3.72 (q, *J* = 5.5 Hz, 4H, alkyl chain 2,3,– $H^{-OCH_2-}$), 3.68 (t, *J* = 5.8 Hz, 2H, alkyl chain 4 – $H^{-OCH_2-}$), 3.38 (t, *J* = 5.8 Hz, 2H, alkyl chain 1 – $H^{-OCH_2-}$), 1.55 – 1.32 (m, 8H, alkyl chain 1,2,3,4 – $H^{-CH_2-}$), 1.23 – 1.03 (m, 10H, alkyl chain 1,2,3,4 – $H^{-CH_2-}$), 0.93 – 0.80 (m, 10H, alkyl chain 1,2,3,4 – $H^{-CH_2-}$), 0.79 – 0.64 (m, 14H, alkyl chain 1,2,3,4 – $H^{-CH_2-}$), 0.44 (t, *J* = 6.8 Hz, 3H, alkyl chain 1 – $H^{-CH_3}$), 0.42 – 0.36 (m, 9H, alkyl chain 2,3,4 – $H^{-CH_3}$). $^{13}$C NMR (101 MHz, $CD_3CN$, 300K) δ 159.34, 158.68, 158.55, 158.13, 157.89, 155.99, 155.85, 155.61, 155.38, 155.05, 153.71, 153.43, 152.62, 152.42, 151.13, 149.35, 147.21, 147.08, 145.75, 145.13, 144.38, 142.22, 142.12, 138.23, 137.30, 135.31, 134.88, 134.46, 134.07, 133.25, 132.95, 127.60, 124.44, 124.39, 123.54, 121.21, 121.11, 120.98, 120.46, 96.50, 75.07, 74.47, 31.16, 29.80, 25.82, 25.28, 22.05, 13.04. $^{19}$F NMR (376 MHz, $CD_3CN$, 300K) δ -115.20, -115.55, -115.91. ESI-TOF (*m/z*): Calcd. $[C_{260}H_{192}N_{36}Ru_3F_{40}P_6O_4\text{-}3PF_6]^{3+}$ for 1566.06, found 1566.06, Calcd. $[C_{260}H_{192}N_{36}Ru_3F_{40}P_6O_4\text{-}4PF_6]^{4+}$ for 1138.31, found 1138.31, Calcd. $[C_{260}H_{192}N_{36}Ru_3F_{40}P_6O_4\text{-}5PF_6]^{5+}$ for 881.65, found 881.65, Calcd. $[C_{260}H_{192}N_{36}Ru_3F_{40}P_6O_4\text{-}6PF_6]^{6+}$ for 710.55, found 710.55.

**Synthesis of supramolecules C1-C5**

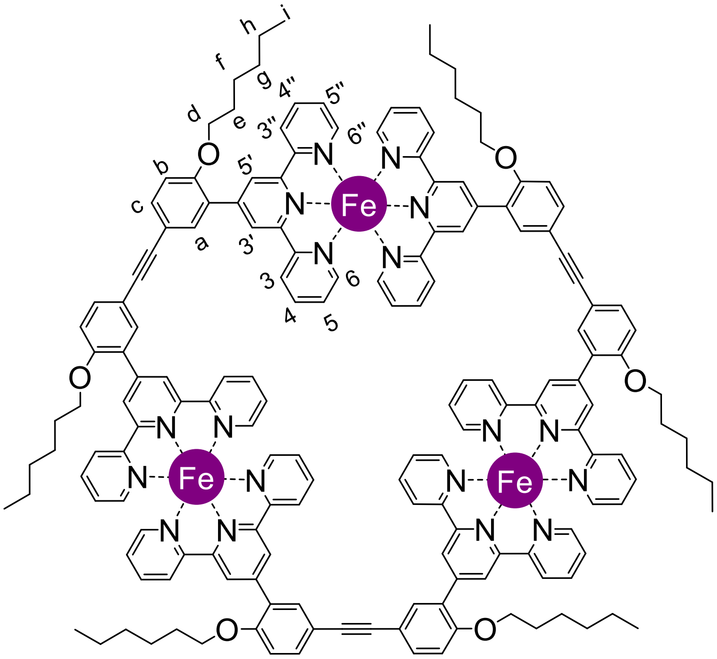


**C1** To a flask containing **L1** (80 mg, 0.095 mmol) and $FeSO_4•7H_2O$ (26.5 mg, 0.095 mmol), 30 mL of chloroform and 30 mL of methanol was added. The mixture was stirred at 80 °C for 36h, then solvent was evaporated under vacuum. The crude product was purified via column chromatography on silica gel with acetonitrile/saturated aqueous $KNO_3$ (100/5, v/v) as the eluent to afford **N1**, which was dried and further washed with water to remove extra $KNO_3$. Afterwards 3 mL of acetonitrile was added to dissolve the precipitate, and the solution was added dropwise to saturated $NH_4PF_6$ solution in methanol to afford purple precipitate. The precipitate was isolated via centrifugation and further washed with methanol, dried in vacuum to give **C1** with $PF_6^-$ as the counterion for further characterizations (34.4 mg, 30.5% yield). $^1$H NMR (500 MHz, $CD_3CN$) δ 9.22 (s, 12H, 12H, tpy-$H^{3',5'}$), 8.53 (dd, $J$ = 8.0, 1.2 Hz, 12H, 12H, tpy-$H^{3,3''}$), 8.26 (d, $J$ = 2.0 Hz, 6H, $H^a$), 7.98 – 7.82 (m, 18H, $H^c$ & tpy-$H^{4,4''}$), 7.45 (d, $J$ = 8.6 Hz, 6H, $H^b$), 7.23 – 7.16 (m, 12H, tpy-$H^{6,6''}$), 7.09 (t, $J$ = 6.0, 0.9 Hz, 12H, $H^{5,5''}$), 4.38 (t, $J$ = 5.9 Hz, 12H, $H^d$), 1.96 – 2.05 (m, 12H, $H^e$)1.58 (p, $J$ = 7.8 Hz, 12H, $H^f$), 1.25 (p, $J$ = 7.5 Hz, 12H, $H^g$), 1.00 (h, $J$ = 7.4 Hz, 12H, $H^h$), 0.52

(t, $J$ = 7.3 Hz, 18H, $H^{t}$). $^{13}$C NMR (151 MHz, $CD_3CN$, 300K) δ 159.66, 158.01, 156.76, 152.95, 147.53, 138.80, 134.94, 133.77, 127.30, 126.41, 124.20, 123.62, 115.96, 113.47, 88.22, 69.10, 31.34, 29.01, 25.91, 22.24, 13.14. DOSY-NMR (600 MHz, $CD_3CN$, 300K): log D = -9.25. ESI-TOF (*m/z*): 448.2 $[M-6PF_6^{-}]^{6+}$ (calcd. *m/z* 448.2), 566.8 $[M-5PF_6^{-}]^{5+}$ (calcd. *m/z* 566.8), 744.7 $[M-4PF_6^{-}]^{4+}$ (calcd. *m/z* 744.7), 1041.3 $[M-3PF_6^{-}]^{3+}$ (calcd. *m/z* 1041.3).

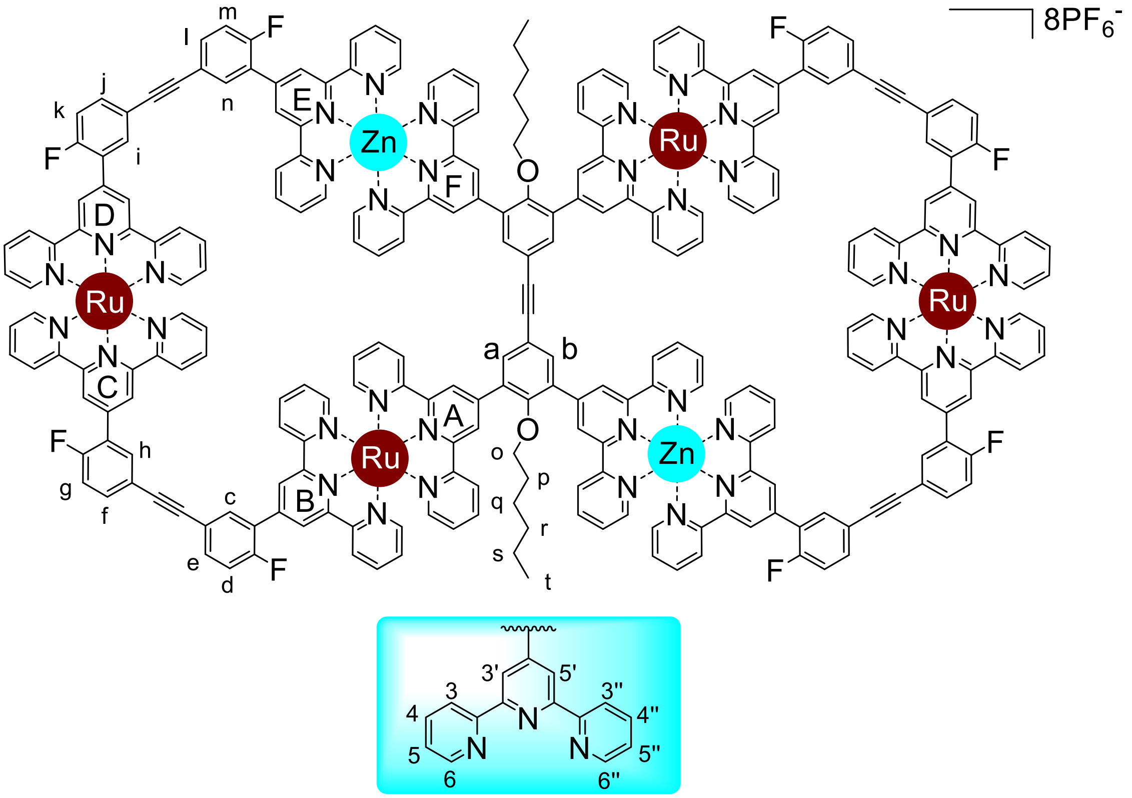


**C2 L2** (5.2 mg, 1.1 μmol) was dissolved in a mixed solvent of 1.0 mL of chloroform and 1.0 mL of methanol, and $Zn(NO_3)_2 \cdot 6H_2O$ (0.65 mg, 2.2 μmol) was dissolved in 2.0 mL of methanol. The two solutions were mixed together and heated under 50 °C for 8h. After that the solution was poured into 15 mL of saturated solution of $NH_4PF_6$ in methanol and the precipitate formed was collected via centrifugation and washed with methanol, dried in vacuum to afford **C2** with $PF_6^{-}$ as the counterion for further characterizations (6.5 mg, 95% yield). $^1$H NMR (500 MHz, $CD_3CN$,

300K) δ 9.27 (s, 2H, tpyF-$H^{3',5'}$), 9.25 (s, 2H, tpyA-$H^{3',5'}$), 9.04 (s, 2H, tpyE-$H^{3',5'}$), 9.03 (s, 2H, tpyC-$H^{3',5'}$), 9.01 (s, 2H, tpyD-$H^{3',5'}$), 8.97 (s, 2H, tpyB-$H^{3',5'}$), 8.85 (d, $J$ = 8.2 Hz, 2H, tpyA-$H^{3,3''}$), 8.73 (dd, $J$ = 8.2, 2.0 Hz, 4H, tpyF,B-$H^{3,3''}$), 8.68 – 8.60 (m, 6H, tpyC,D,E-$H^{3,3''}$), 8.52 (d, $J$ = 2.1 Hz, 1H, $H^{b}$), 8.41 (d, $J$ = 2.0 Hz, 1H, $H^{a}$), 8.36 – 8.13 (m, 8H, $H^{n,h,i,c}$, tpyF,A-$H^{4,4''}$, $H^{m,g,k,d}$), 8.07 – 7.88 (m, 8H, tpyB,C,D,E-$H^{4,4''}$), 7.71 – 7.42 (m, 14H, $H^{e,f,j,l}$, tpyF,A-$H^{5,5''}$, tpyB,C,D,E-$H^{6,6''}$ ), 7.31 – 7.15 (m, 12H, tpyB-$H^{5,5''}$, tpyA,F-$H^{6,6''}$, tpyC,D,E-$H^{5,5''}$), 3.83 (t, $J$ = 5.7 Hz, 2H, $H^{o}$), 1.47 (dt, $J$ = 11.2, 5.8 Hz, 2H, $H^{p}$), 1.38 – 1.18 (m, 2H, $H^{q}$), 0.94 (dq, $J$ = 15.5, 7.5 Hz, 2H, $H^{r}$), 0.74 (h, $J$ = 7.4 Hz, 2H, $H^{s}$), 0.36 (t, $J$ = 7.3 Hz, 3H, $H^{t}$). $^{13}$C NMR (151 MHz, $CD_3CN$, 300K) δ 161.09, 160.74, 159.05, 157.88, 155.37, 153.38, 152.58, 152.32, 149.67, 148.78, 148.59, 147.90, 145.74, 142.23, 141.46, 138.18, 135.28, 134.40, 130.88, 127.60, 125.86, 124.61, 124.20, 123.55, 123.53, 122.60, 120.18, 88.27, 75.60, 31.26, 30.14, 26.04, 22.12, 12.99. $^{19}$F NMR (376 MHz, $CD_3CN$, 300K) δ -115.30. DOSY-NMR (600 MHz, $CD_3CN$, 300K): log D = -9.40. ESI-TOF (*m/z*): 483.3 $[M\text{-}10PF_6^-]^{10+}$ (calcd. *m/z* 483.3), 553.3 $[M\text{-}9PF_6^-]^{9+}$ (calcd. *m/z* 553.3), 640.3 $[M\text{-}8PF_6^-]^{8+}$ (calcd. *m/z* 640.3), 752.5 $[M\text{-}7PF_6^-]^{7+}$ (calcd. *m/z* 752.5), 902.2 $[M\text{-}6PF_6^-]^{6+}$ (calcd. *m/z* 902.2), 1111.5 $[M\text{-}5PF_6^-]^{5+}$ (calcd. *m/z* 1111.5), 1425.7 $[M\text{-}4PF_6^-]^{4+}$ (calcd. *m/z* 1425.7).

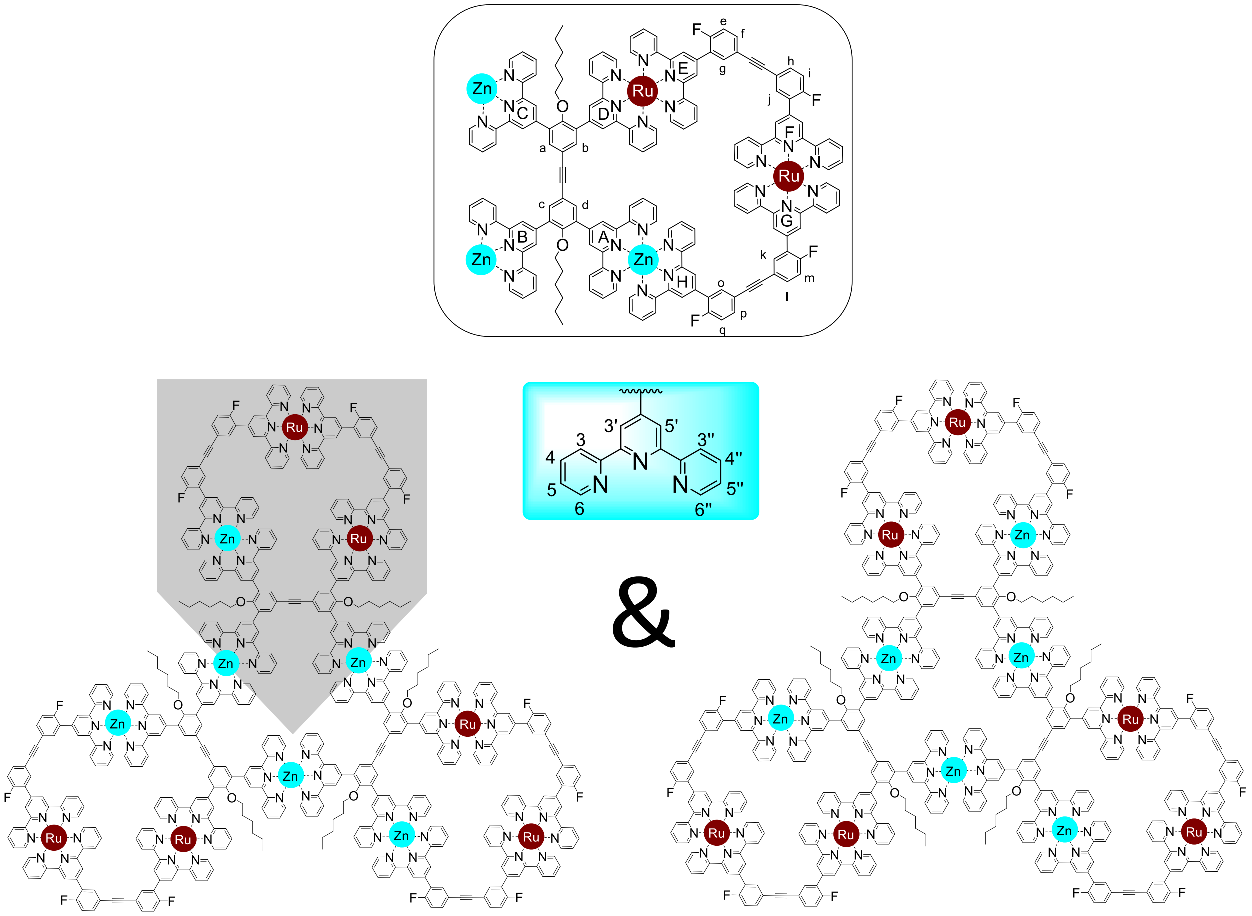

**C3 L3** (3.5 mg, 1.0 μmol) was dissolved in 2.0 mL of acetonitrile while $Zn(NO_3)_2$•$6H_2O$ (0.6 mg, 2.0 μmol) was dissolved in 0.5 mL of methanol. The two solutions were mixed together and heated under 50 °C for 8h. After that the solution was poured into 15 mL of saturated solution of $NH_4PF_6$ in methanol and the precipitate formed was collected via centrifugation and washed with methanol, dried in vacuum to afford **C3** with $PF_6^-$ as the counterion (3.8 mg, 93% yield). Due to the existing of isomers and overlap between the two species, only the major (taller) peaks were signed in NMR spectrum. $^1$H NMR (600 MHz, $CD_3CN$, 300K) δ 9.34 (s, 2H, tpyB-$H^{3',5'}$), 9.32 (s, 2H, tpyC-$H^{3',5'}$), 9.30 (s, 2H, tpyA-$H^{3',5'}$), 9.29 (s, 2H, tpyD-$H^{3',5'}$), 9.09 – 8.98 (m, 8H, tpyH,E,F,G-$H^{3',5'}$), 8.87 (t, $J$ = 6.7 Hz, 4H, tpyB,C-$H^{3,3''}$), 8.81 (d, $J$ = 8.1 Hz, 2H, tpyA-$H^{3,3''}$), 8.77 – 8.72 (m, 2H, tpyD-$H^{3,3''}$), 8.72 – 8.63 (m, 2H, tpyH-$H^{3,3''}$), 8.61 – 8.58 (m, 6H, tpyE,F,G-$H^{3,3''}$), 8.55 (s, 1H, $H^a$), 8.52 (s, 1H, $H^c$), 8.50 (s, 2H, $H^{b,d}$), 8.38 – 8.18 (m, 12H, tpyA,B,C,H-$H^{4,4''}$, $H^{g,j,k,o}$), 8.05 – 7.87 (m,

20H, tpyD,E,F,G-$H^{4,4''}$, tpyA,B,C,H-$H^{6,6''}$, $H^{f,h,l,p}$), 7.68 – 7.59 (m, 4H, $H^{g,m,i,e}$), 7.57 – 7.38 (m, 12H, tpyD,E,F,G-$H^{6,6''}$, tpyA,B,C,H-$H^{5,5''}$), 7.23 (m, 8H, tpyD,E,F,G-$H^{5,5''}$), 3.94 – 3.72 (m, 4H, alkyl-$H^{-OCH_2-}$), 1.53 – 1.45 (m, 4H, alkyl-$H^{-CH_2-}$), 1.34 – 1.25 (m, 4H, alkyl-$H^{-CH_2-}$), 1.00 – 0.92 (m, 4H, alkyl-$H^{-CH_2-}$), 0.34 (dt, $J$ = 13.8, 7.2 Hz, 6H, alkyl-$H^{-CH_3}$). $^{13}$C NMR (151 MHz, $CD_3CN$, 300K) δ 159.06, 157.89, 155.58, 155.37, 152.94, 152.57, 152.33, 149.62, 147.99, 147.68, 145.10, 143.07, 141.54, 141.43, 139.62, 138.18, 135.27, 134.41, 130.08, 128.55, 127.60, 124.62, 124.21, 123.52, 88.29, 75.72, 31.27, 30.24, 26.11, 22.09, 15.14, 12.96. $^{19}$F NMR (376 MHz, $CD_3CN$, 300K) δ -114.95, -115.07, -115.27. DOSY-NMR (600 MHz, $CD_3CN$, 300K): log D = -9.56. ESI-TOF (*m/z*): 891.6 $[M\text{-}12PF_6^-]^{12+}$ (calcd. *m/z* 891.6), 985.9 $[M\text{-}11PF_6^-]^{11+}$ (calcd. *m/z* 985.9), 1099.0 $[M\text{-}10PF_6^-]^{10+}$ (calcd. *m/z* 1099.0), 1237.2 $[M\text{-}9PF_6^-]^{9+}$ (calcd. *m/z* 1237.2), 1409.9 $[M\text{-}8PF_6^-]^{8+}$ (calcd. *m/z* 1409.9), 1632.1 $[M\text{-}7PF_6^-]^{7+}$ (calcd. *m/z* 1632.1), 1928.2 $[M\text{-}6PF_6^-]^{6+}$ (calcd. *m/z* 1928.2), 2342.9 $[M\text{-}5PF_6^-]^{5+}$ (calcd. *m/z* 2342.9).

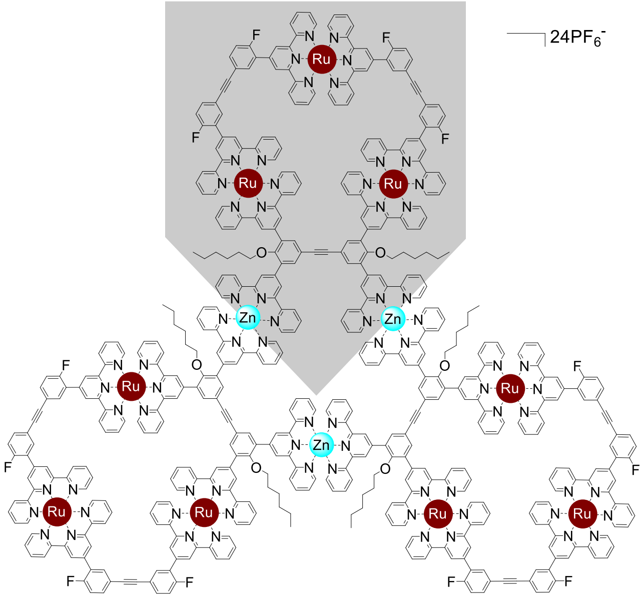


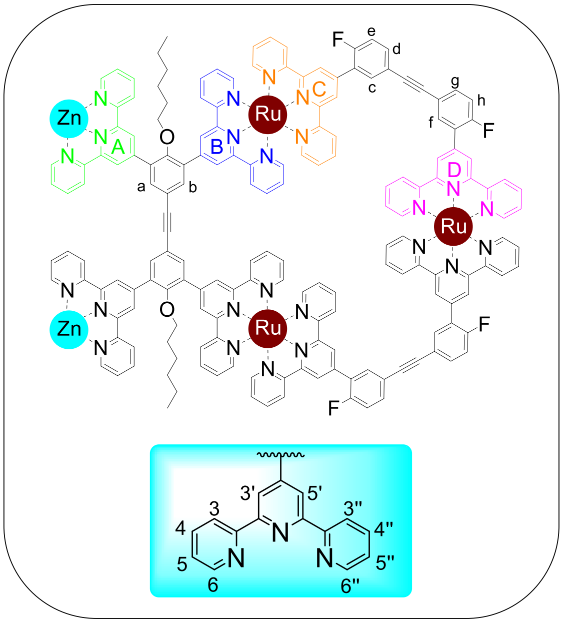


**C3’ L3’** (3.0 mg, 0.95 μmol) was dissolved in a mixed solvent of 1.0 mL of chloroform and 1.0 mL of methanol, while $Zn(NO_3)_2$•$6H_2O$ (0.28 mg, 0.95 μmol) was dissolved in 2.0 mL of methanol. The two solutions were mixed together and heated under 50 °C for 8h. After that the solution was poured into 15 mL of saturated solution of $NH_4PF_6$ in methanol and the precipitate formed was collected via centrifugation and washed with methanol, dried in vacuum to afford **C3’** with $PF_6^-$ as the counterion (3.7 mg, 94% yield). $^1$H NMR (500 MHz, $CD_3CN$, 300K) δ 9.37 (s, 2H, tpyA-$H^{3',5'}$), 9.33 (s, 2H, tpyB-$H^{3',5'}$), 9.05 (s, 2H, tpyC-$H^{3',5'}$), 9.03 (s, 2H, tpyD-$H^{3',5'}$), 8.90 (d, $J$ = 8.1 Hz, 2H, tpyA-$H^{3,3''}$), 8.77 (d, $J$ = 8.1 Hz, 2H, tpyB-$H^{3,3''}$), 8.70 – 8.62 (m, 4H, tpyC,D-$H^{3,3''}$), 8.58 (s, 1H, $H^{b}$), 8.53 (s, 1H, $H^{a}$), 8.48 – 8.20 (m, 4H, $H^{c,f}$, tpyA-$H^{4,4''}$), 8.20 – 7.80 (m, 8H, $H^{e,h}$, tpyB,C,D-$H^{4,4''}$), 7.70 – 7.60 (m, 2H, $H^{d,g}$), 7.59 – 7.40 (m, 8H, tpyA-$H^{5,5''}$tpyB,C,D-$H^{6,6''}$), 7.35 – 7.17 (m, 8H, tpyA-$H^{6,6''}$, tpyB,C,D-$H^{5,5''}$), 3.91 (s, 2H, alkyl-$H^{-OCH_2-}$), 1.65 – 1.16 (m, 4H, alkyl-$H^{-CH_2-}$), 1.08 – 0.93 (m, 2H, alkyl- $H^{-CH_2-}$), 0.78 – 0.75 (m, 2H, alkyl-$H^{-CH_2-}$), 0.34 (t, $J$ = 7.2 Hz, 3H, alkyl-$H^{-CH_3}$). $^{13}$C NMR (151 MHz, $CD_3CN$) δ 160.75, 159.06, 158.02, 157.89, 155.37, 155.21, 153.34, 152.58, 149.60, 147.89, 144.67, 144.35, 142.23, 141.56, 138.23, 135.30, 134.41, 133.55, 133.10, 129.89, 127.92, 127.62, 125.96, 124.62, 124.20, 123.52, 120.21, 88.27, 75.73, 31.29, 30.25,

26.14, 22.11, 12.96. $^{19}$F NMR (376 MHz, $CD_3CN$, 300K) δ -115.30. DOSY-NMR (600 MHz, $CD_3CN$, 300K): log D = -9.57. ESI-TOF (*m/z*): 639.6 $[M-16PF_6^-]^{16+}$ (calcd. *m/z* 639.6), 691.9 $[M-15PF_6^-]^{15+}$ (calcd. *m/z* 691.9), 751.6 $[M-14PF_6^-]^{14+}$ (calcd. *m/z* 751.6), 820.6 $[M-13PF_6^-]^{13+}$ (calcd. *m/z* 820.6), 901.1 $[M-12PF_6^-]^{12+}$ (calcd. *m/z* 901.1), 996.2 $[M-11PF_6^-]^{11+}$ (calcd. *m/z* 996.2), 1426.2 $[M-10PF_6^-]^{10+}$ (calcd. *m/z* 1110.3), 1249.8 $[M-9PF_6^-]^{9+}$ (calcd. *m/z* 1249.8), 1424.1 $[M-8PF_6^-]^{8+}$ (calcd. *m/z* 1424.1), 1648.2 $[M-7PF_6^-]^{7+}$ (calcd. *m/z* 1648.2).

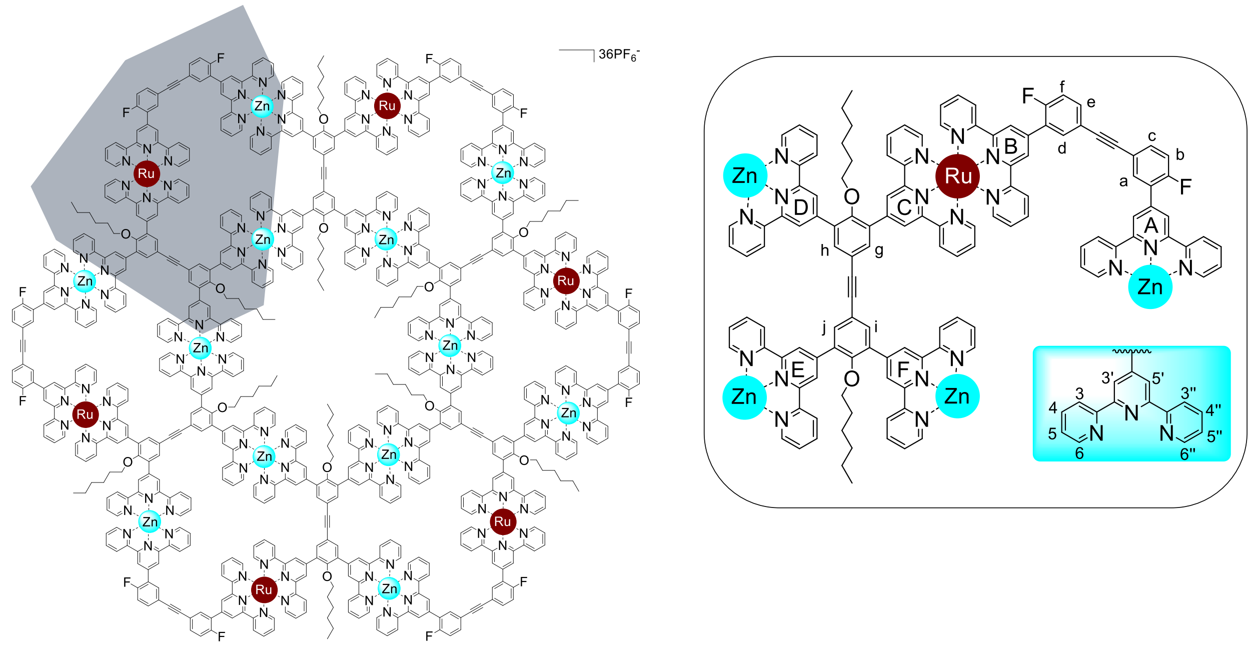


**C4 L4** (4.0 mg, 1.82 μmol) was dissolved in a mixed solvent of 1.0 mL of chloroform and 1.0 mL of methanol, while $Zn(NO_3)_2$•$6H_2O$ (1.1 mg, 3.63 μmol) was dissolved in 2.0 mL of methanol. The two solutions were mixed together and heated under 50 °C for 8h. After that the solution was poured into 15 mL of saturated solution of $NH_4PF_6$ in methanol and the precipitate formed was

collected via centrifugation and washed with methanol, dried in vacuum to afford **C4** with $PF_6^-$ as the counterion. (5.2 mg, 93% yield). $^1$H NMR (600 MHz, $CD_3CN$, 300K) δ 9.35 (s, 4H, tpyE,F-*H*$^{3',5'}$), 9.33 (s, 2H, tpyD-*H*$^{3',5'}$), 9.32 (s, 2H, tpyA-*H*$^{3',5'}$), 9.06 (s, 2H, tpyC-*H*$^{3',5'}$), 9.04 (s, 2H, tpyB-*H*$^{3',5'}$), 8.92 – 8.83 (m, 6H, tpyE,F,D-*H*$^{3,3''}$), 8.77 (d, *J* = 8.2 Hz, 2H, tpyA-*H*$^{3,3''}$), 8.73 (d, *J* = 8.1 Hz, 2H, tpyB-*H*$^{3,3''}$), 8.67 (d, *J* = 8.2 Hz, 2H, tpyC-*H*$^{3,3''}$), 8.58 (d, *J* = 1.9 Hz, 1H, *H*$^{h}$), 8.53 (s, 2H, *H*$^{i,j}$), 8.50 (d, *J* = 1.8 Hz, 1H, *H*$^{a}$), 8.36 – 8.32 (m, 1H, *H*$^{g}$), 8.31 – 8.16 (m, 9H, *H*$^{d}$, tpyD,E,F,B-*H*$^{4,4''}$), 8.07 – 7.90 (m, 14H, tpyA,C-*H*$^{4,4''}$, tpyD,E,F,B-*H*$^{6,6''}$, *H*$^{c,e}$), 7.65 (ddd, *J* = 10.0, 8.0, 5.4 Hz, 2H, *H*$^{f,b}$), 7.57 – 7.51 (m, 4H, tpyA,C-*H*$^{6,6''}$), 7.50 – 7.42 (m, 8H, tpyB,D,E,F-*H*$^{5,5''}$), 7.34 – 7.22 (m, 4H, tpyA,C-*H*$^{5,5''}$), 3.88 (s, 2H, alkyl-*H*$^{-OCH_2-}$), 3.77 (s, 2H, alkyl-*H*$^{-OCH_2-}$), 1.55 – 1.38 (m, 4H, alkyl-*H*$^{-CH_2-}$), 1.36 – 1.19 (m, 4H, alkyl-*H*$^{-CH_2-}$), 1.06 – 0.56 (m, 8H, alkyl-*H*$^{-CH_2-}$), 0.34 (t, *J* = 7.3 Hz, 3H, alkyl-*H*$^{-CH_3}$), 0.27 (t, *J* = 7.3 Hz, 3H, alkyl-*H*$^{-CH_3}$). $^{13}$C NMR (126 MHz, $CD_3CN$, 300K) δ 158.95, 158.82, 158.07, 155.75, 155.66, 155.43, 155.26, 153.13, 152.88, 152.76, 152.39, 150.80, 149.80, 149.62, 148.35, 148.08, 147.87, 147.80, 147.72, 144.41, 142.32, 141.51, 140.67, 138.30, 136.84, 136.09, 135.50, 134.19, 133.62, 133.19, 133.11, 129.42, 127.72, 127.23, 125.91, 125.49, 125.38, 124.78, 124.51, 124.32, 123.71, 123.35, 120.65, 120.41, 91.62, 89.44, 89.16, 88.47, 88.17, 75.80, 31.30, 31.25, 30.24, 26.12, 22.49, 22.14, 22.00, 12.98, 12.90. $^{19}$F NMR (376 MHz, $CD_3CN$, 300K) δ -115.19. DOSY-NMR (600 MHz, $CD_3CN$, 300K): log D = -9.63. ESI-TOF (*m/z*): 694.6 $[M-22PF_6^-]^{22+}$ (calcd. *m/z* 694.6), 734.6 $[M-21PF_6^-]^{21+}$ (calcd. *m/z* 734.6), 778.5 $[M-20PF_6^-]^{20+}$ (calcd. *m/z* 778.5), 827.1 $[M-19PF_6^-]^{19+}$ (calcd. *m/z* 827.1), 881.1 $[M-18PF_6^-]^{18+}$ (calcd. *m/z* 881.1), 941.5 $[M-17PF_6^-]^{17+}$ (calcd. *m/z* 941.5), 1009.4 $[M-16PF_6^-]^{16+}$ (calcd. *m/z* 1009.4), 1086.4 $[M-15PF_6^-]^{15+}$ (calcd. *m/z* 1086.4), 1174.3 $[M-14PF_6^-]^{14+}$ (calcd. *m/z* 1174.3), 1275.8 $[M-13PF_6^-]^{13+}$ (calcd. *m/z* 1275.8), 1394.2 $[M-12PF_6^-]^{12+}$ (calcd. *m/z* 1394.2), 1534.1 [M-

11PF$_6^-$]$^{11+}$ (calcd. *m/z* 1534.1), 1702.0 [M-10PF$_6^-$]$^{10+}$ (calcd. *m/z* 1702.0), 1907.3 [M-9PF$_6^-$]$^{9+}$ (calcd. *m/z* 1907.3), 2163.8 [M-8PF$_6^-$]$^{8+}$ (calcd. *m/z* 2163.8).

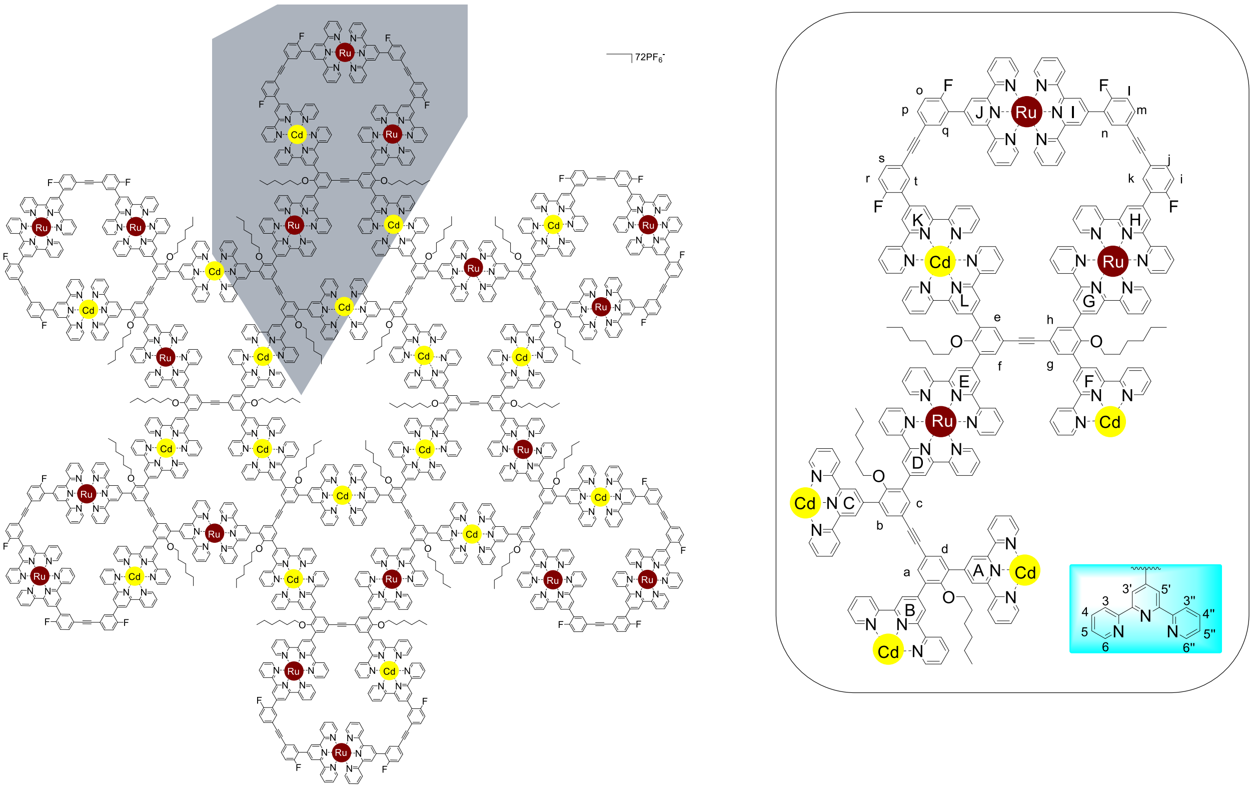

**C5 L5** (3.5 mg, 0.68 μmol) was dissolved in 2.0 mL of acetonitrile, while Cd(NO$_3$)$_2$•4H$_2$O (0.63 mg, 2.05 μmol) was dissolved in 0.3 mL of methanol. The two solutions were mixed together and heated under 50 °C for 8h. After that the solution was poured into 15 mL of saturated solution of NH$_4$PF$_6$ in methanol and the precipitate formed was collected via centrifugation and washed with methanol, dried in vacuum to afford **C5** with PF$_6^-$ as the counterion (4.1 mg, 95% yield). $^1$H NMR (600 MHz, CD$_3$CN, 300K) δ 9.24 – 9.39 (m, 16H, tpyC,D,L,A,B,K,F,E,G-*H*$^{3',5'}$), 9.10 (s, 2H, tpyH-*H*$^{3',5'}$), 9.05 (s, 2H, tpyI-*H*$^{3',5'}$), 9.02 (s, 2H, tpyJ-*H*$^{3',5'}$), 8.97 (m, 2H, tpyK-*H*$^{3,3''}$), 8.91 (m, 4H, tpyA,B-*H*$^{3,3''}$), 8.85 (m, 4H, tpyD,E-*H*$^{3,3''}$), 8.80 (m, 4H, tpyC,L-*H*$^{3,3''}$), 8.73 (m, 4H, tpyF,G-*H*$^{3,3''}$), 8.65 – 8.60 (m, 6H, tpyH,I,J-*H*$^{3,3''}$), 8.58 (s, 2H, *H*$^a$ & *H*$^b$), 8.49 (s, 1H, *H*$^c$), 8.46 (s, 1H, *H*$^f$),

8.40– 8.30 (m, 7H, $H^{k,n,q}$, tpyA,B-$H^{4,4''}$), 8.30 – 8.25 (m, 8H, tpyE,D-$H^{4,4''}$, $H^{d,e,h,s}$), 8.25 – 8.13 (m, 4H, tpyK,F-$H^{4,4''}$), 8.10 – 8.05 (m, 6H, $H^{t,g}$, tpyC,L-$H^{4,4''}$), 8.05 – 7.85 (m, 15H, tpyD,E,G,H,I,J-$H^{4,4''}$, $H^{j,m,p}$), 7.70 – 7.57 (m, 15H, $H^{o,r,i,l}$, tpyK,F-$H^{6,6''}$, tpyA,B-$H^{5,5''}$, tpyC,L-$H^{6,6''}$), 7.57 – 7.45 (m, 8H, tpyD,E,G,H,-$H^{6,6''}$), 7.45 – 7.40 (m, 4H, tpyI,J-$H^{6,6''}$), 7.40 – 7.37 (m, 4H, tpyA,B-$H^{6,6''}$), 7.37 – 7.18 (m, 12H, tpyD,E,G,H,I,J-$H^{5,5''}$) 3.85 – 3.73 (m, 8H, alkyl- $H^{-OCH_2^-}$), 1.70 – 1.24 (m, 16H, alkyl- $H^{-CH_2^-}$), 1.12 – 0.80 (m, 8H, alkyl- $H^{-CH_2^-}$), 0.80 – 0.60 (m, 8H, alkyl- $H^{-CH_2^-}$), 0.41–0.25 (m, 12H, alkyl- $H^{-CH_3}$). $^{13}$C NMR (151 MHz, $CD_3CN$) δ 160.65, 159.07, 157.89, 155.37, 152.58, 148.71, 147.02, 145.30, 142.22, 141.53, 138.18, 137.43, 134.40, 133.19, 127.60, 125.95, 124.62, 123.53, 120.19, 88.09, 75.62, 31.30, 30.22, 26.12, 22.12, 13.02. $^{19}$F NMR (376 MHz, $CD_3CN$, 300K) δ -115.31. DOSY-NMR (600 MHz, $CD_3CN$, 300K): log D = -9.81. ESI-TOF (*m/z*):1008.6 $[M-33PF_6^-]^{33+}$ (calcd. *m/z* 1008.6), 1044.6 $[M-32PF_6^-]^{32+}$ (calcd. *m/z* 1044.6), 1083.0 $[M-31PF_6^-]^{31+}$ (calcd. *m/z* 1083.0), 1124.0 $[M-30PF_6^-]^{30+}$ (calcd. *m/z* 1124.0), 1167.7 $[M-29PF_6^-]^{29+}$ (calcd. *m/z* 1167.7), 1214.6 $[M-28PF_6^-]^{28+}$ (calcd. *m/z* 1214.6), 1264.9 $[M-27PF_6^-]^{27+}$ (calcd. *m/z* 1264.9), 1319.2 $[M-26PF_6^-]^{26+}$ (calcd. *m/z* 1319.2), 1377.7 $[M-25PF_6^-]^{25+}$ (calcd. *m/z* 1377.7), 1441.2 $[M-24PF_6^-]^{24+}$ (calcd. *m/z* 1441.2), 1510.1 $[M-23PF_6^-]^{23+}$ (calcd. *m/z* 1510.1), 1585.4 $[M-22PF_6^-]^{22+}$ (calcd. *m/z* 1585.4), 1667.8 $[M-21PF_6^-]^{21+}$ (calcd. *m/z* 1667.8), 1758.4 $[M-20PF_6^-]^{20+}$ (calcd. *m/z* 1758.4), 1858.6 $[M-19PF_6^-]^{19+}$ (calcd. *m/z* 1858.6), 1969.9 $[M-18PF_6^-]^{18+}$ (calcd. *m/z* 1969.9), 2094.3 $[M-17PF_6^-]^{17+}$ (calcd. *m/z* 2094.3), 2234.3 $[M-16PF_6^-]^{16+}$ (calcd. *m/z* 2234.3), 2392.9 $[M-15PF_6^-]^{15+}$ (calcd. *m/z* 2392.9), 2574.1 $[M-14PF_6^-]^{14+}$ (calcd. *m/z* 2574.1).

**ESI-MS for ligands L1-L5**

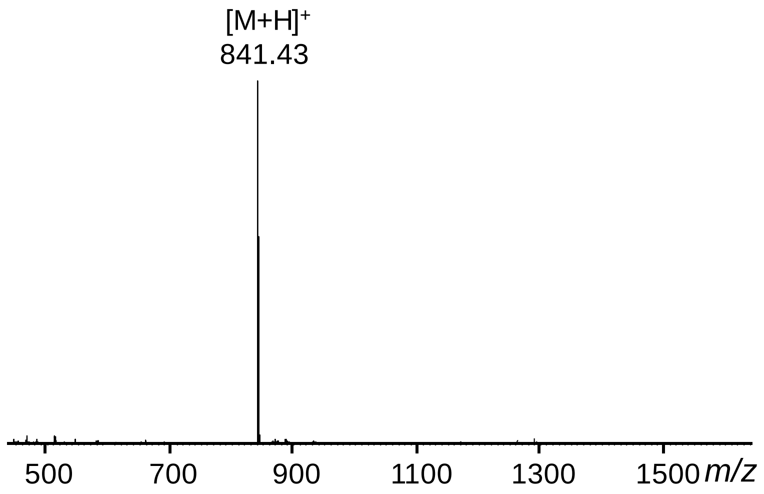


**Supplementary Figure 8.** ESI-MS for ligand **L1**

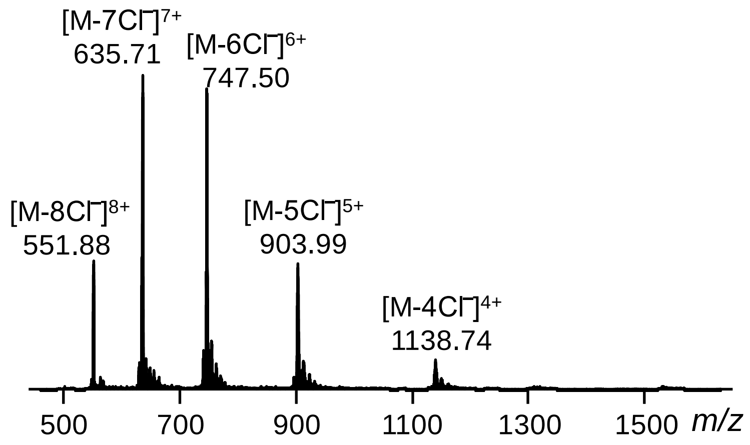


**Supplementary Figure 9.** ESI-MS for ligand **L2**

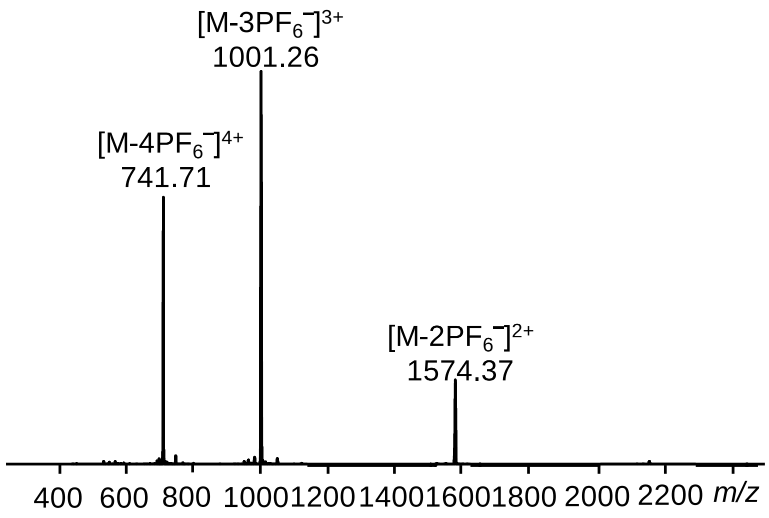


**Supplementary Figure 10.** ESI-MS for ligand **L3**

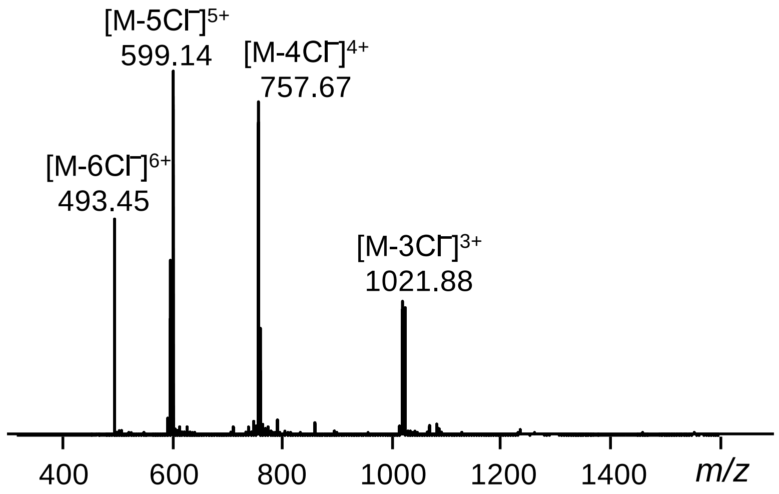


**Supplementary Figure 11.** ESI-MS for ligand **L3'**

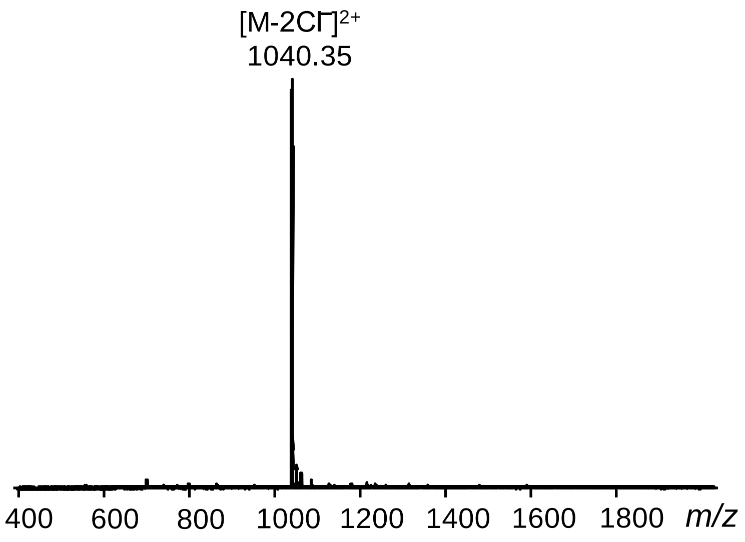


**Supplementary Figure 12.** ESI-MS for ligand **L4**

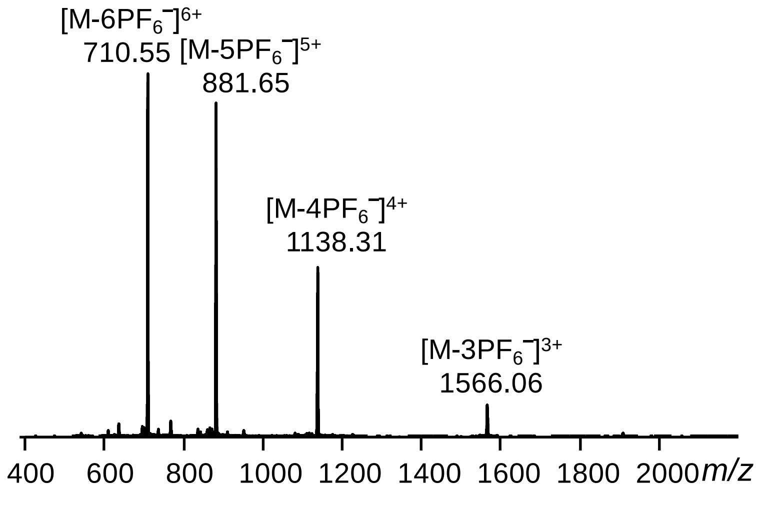


**Supplementary Figure 13.** ESI-MS for ligand **L5**

## ESI-MS for supramolecules C1-C5

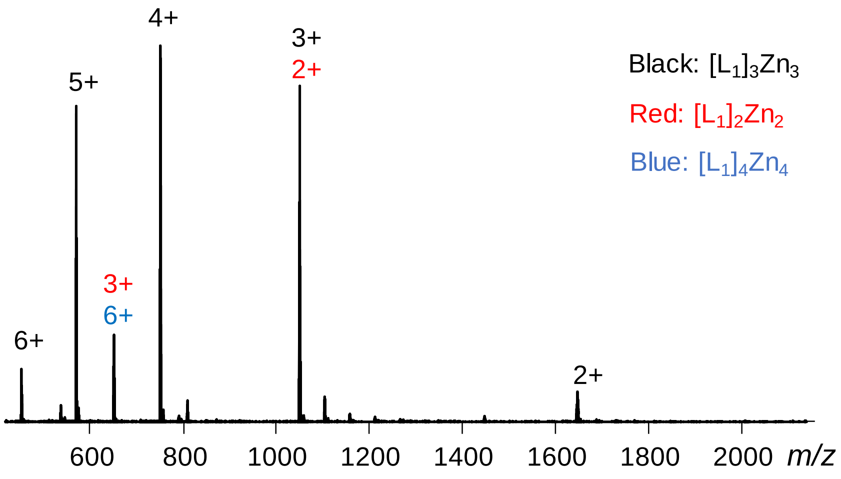


**Supplementary Figure 14.** ESI-MS for self-assembly of **L1** with Zn(II)

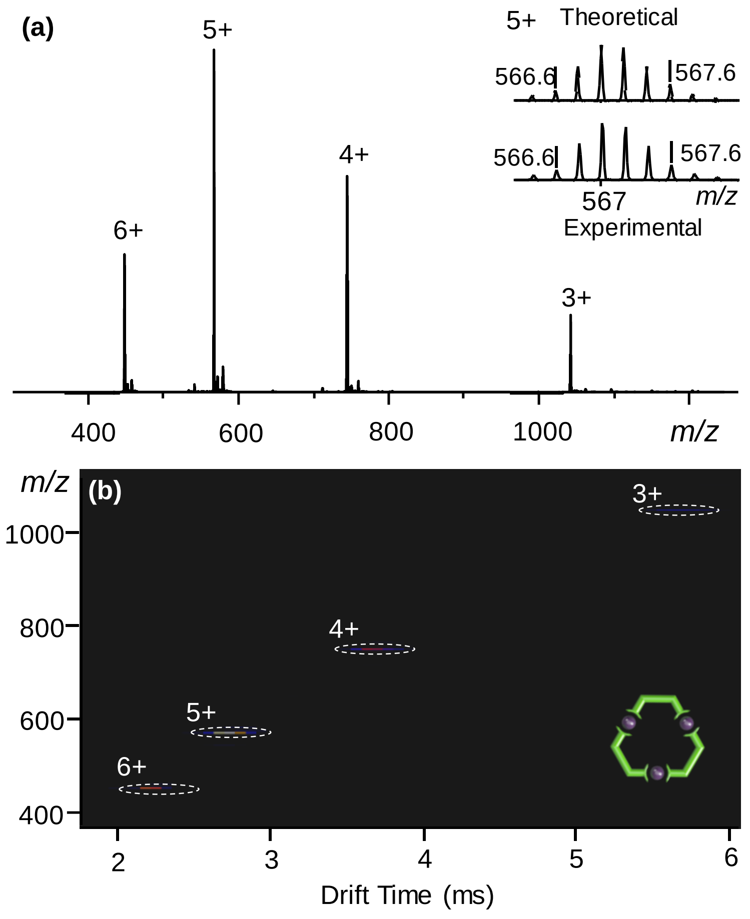


**Supplementary Figure 15.** ESI-MS **(a)** and TWIM-MS **(b)** for **C1**

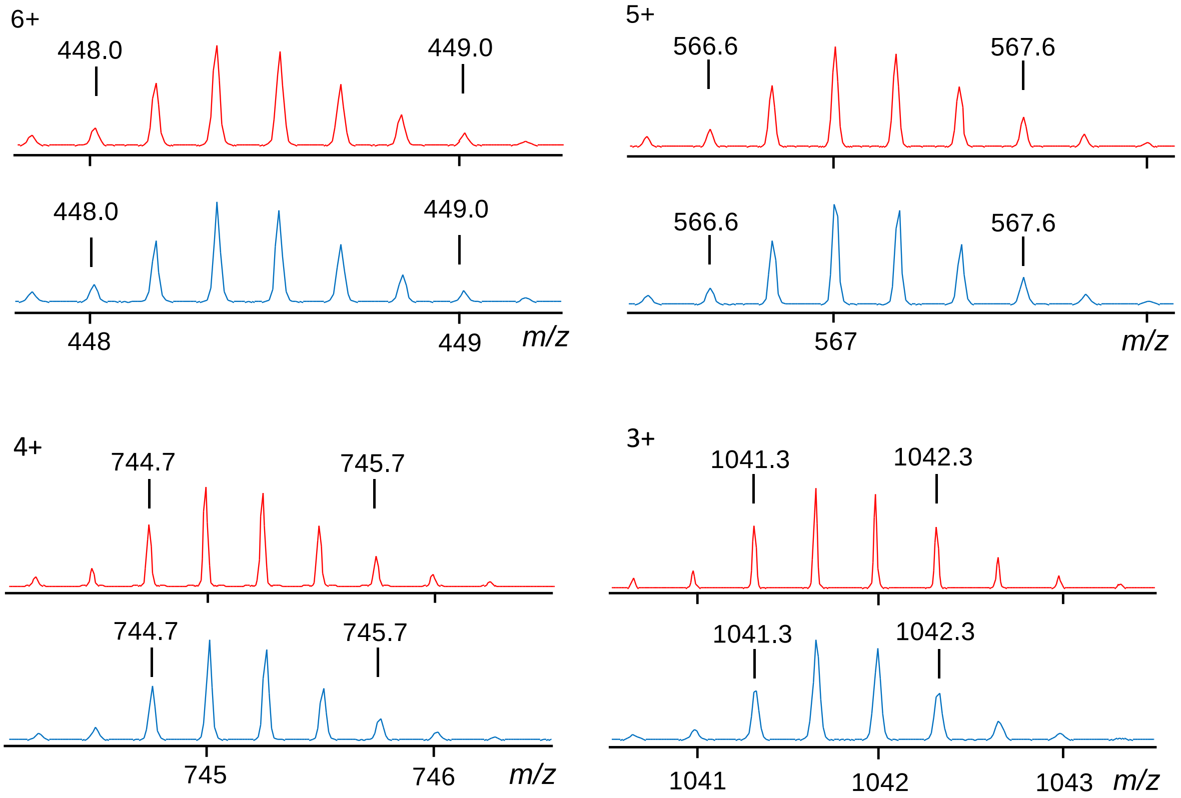


**Supplementary Figure 16.** Theoretical isotope (red) and experimental isotope (blue) for **C1**

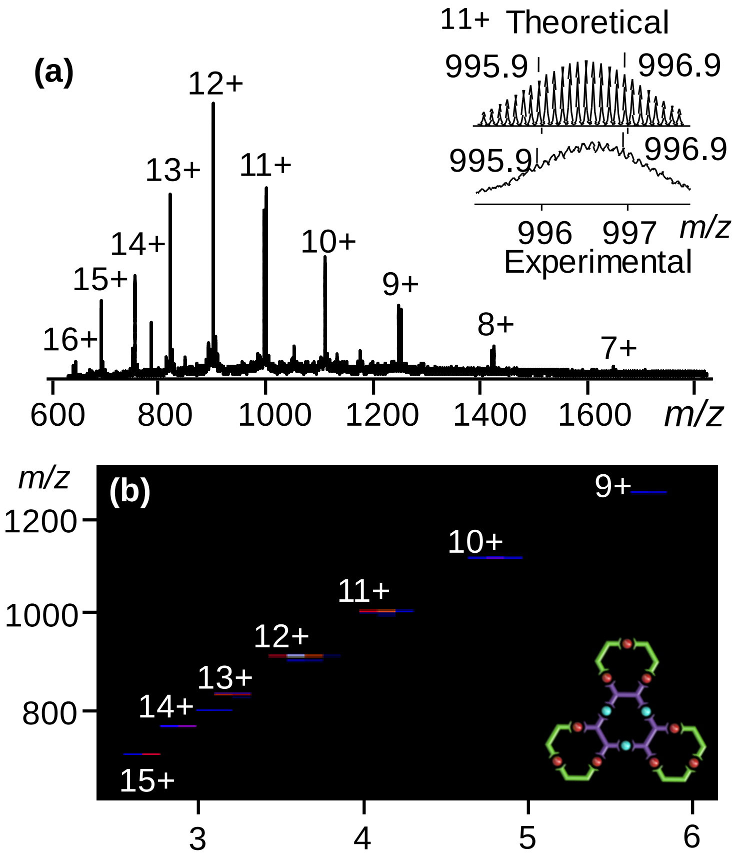


**Supplementary Figure 17.** ESI-MS **(a)** and TWIM-MS **(b)** for **C3'**

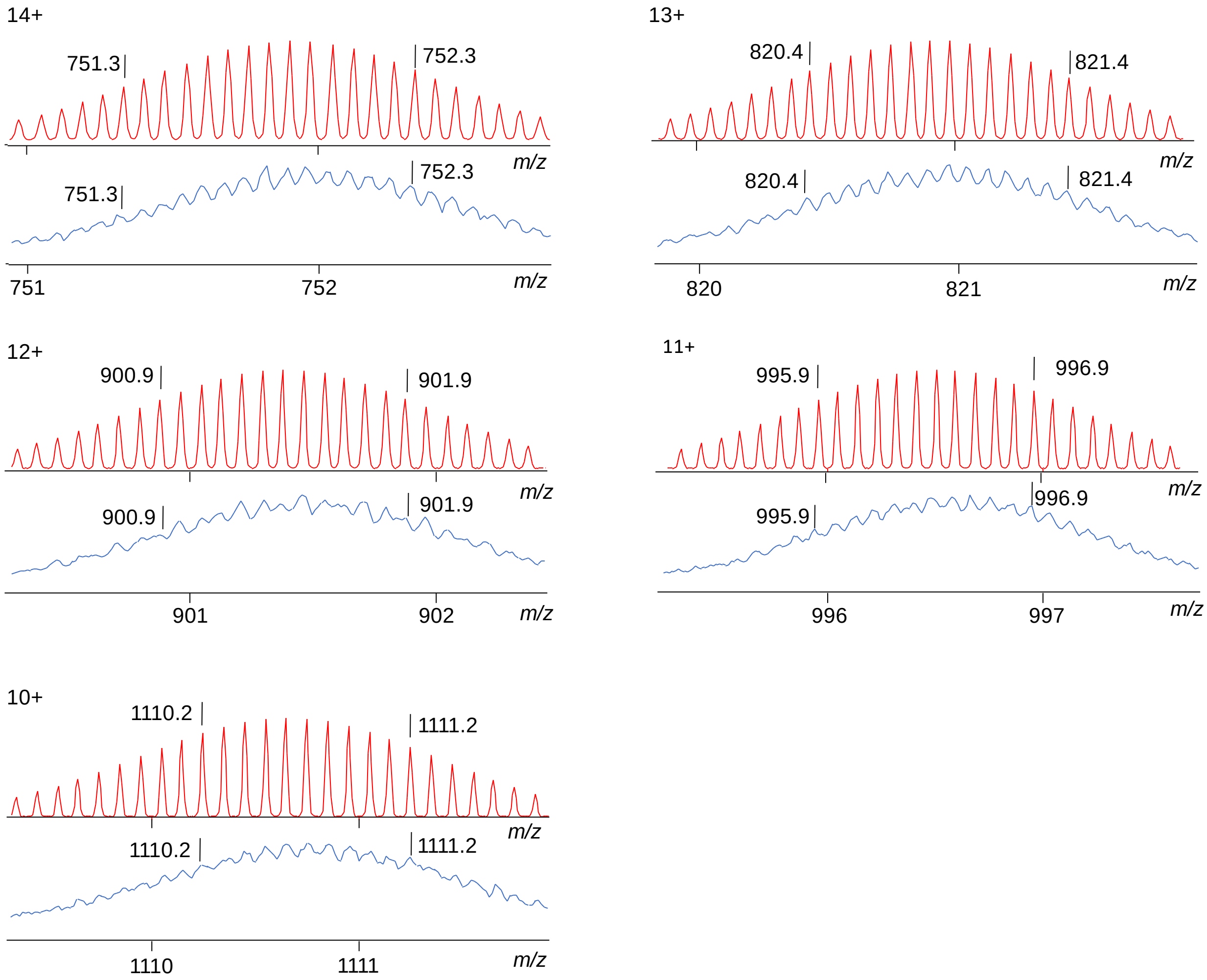


**Supplementary Figure 18.** Theoretical isotope (red) and experimental isotope (blue) for **C3’**

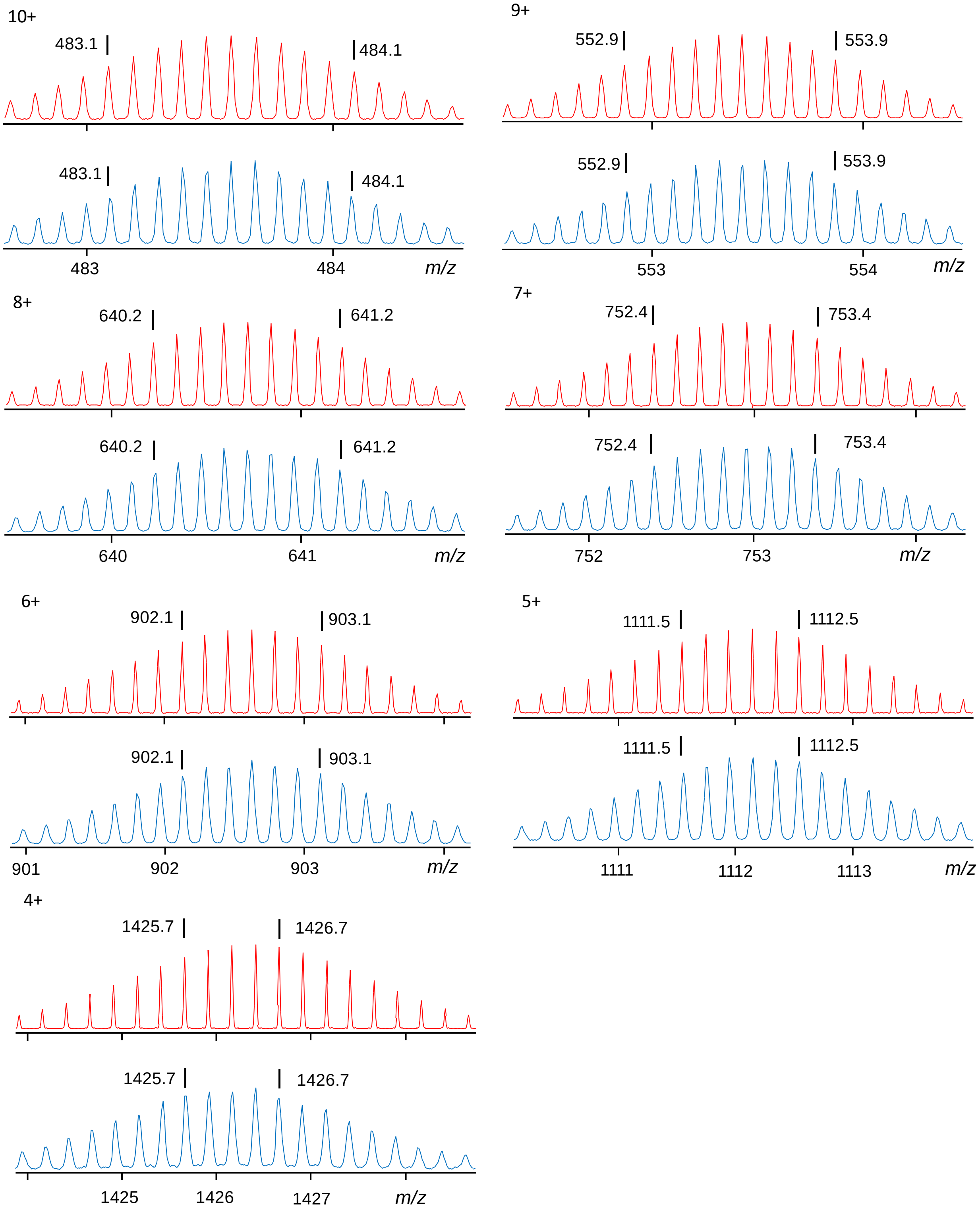


**Supplementary Figure 19.** Theoretical isotope (red) and experimental isotope (blue) for **C2**

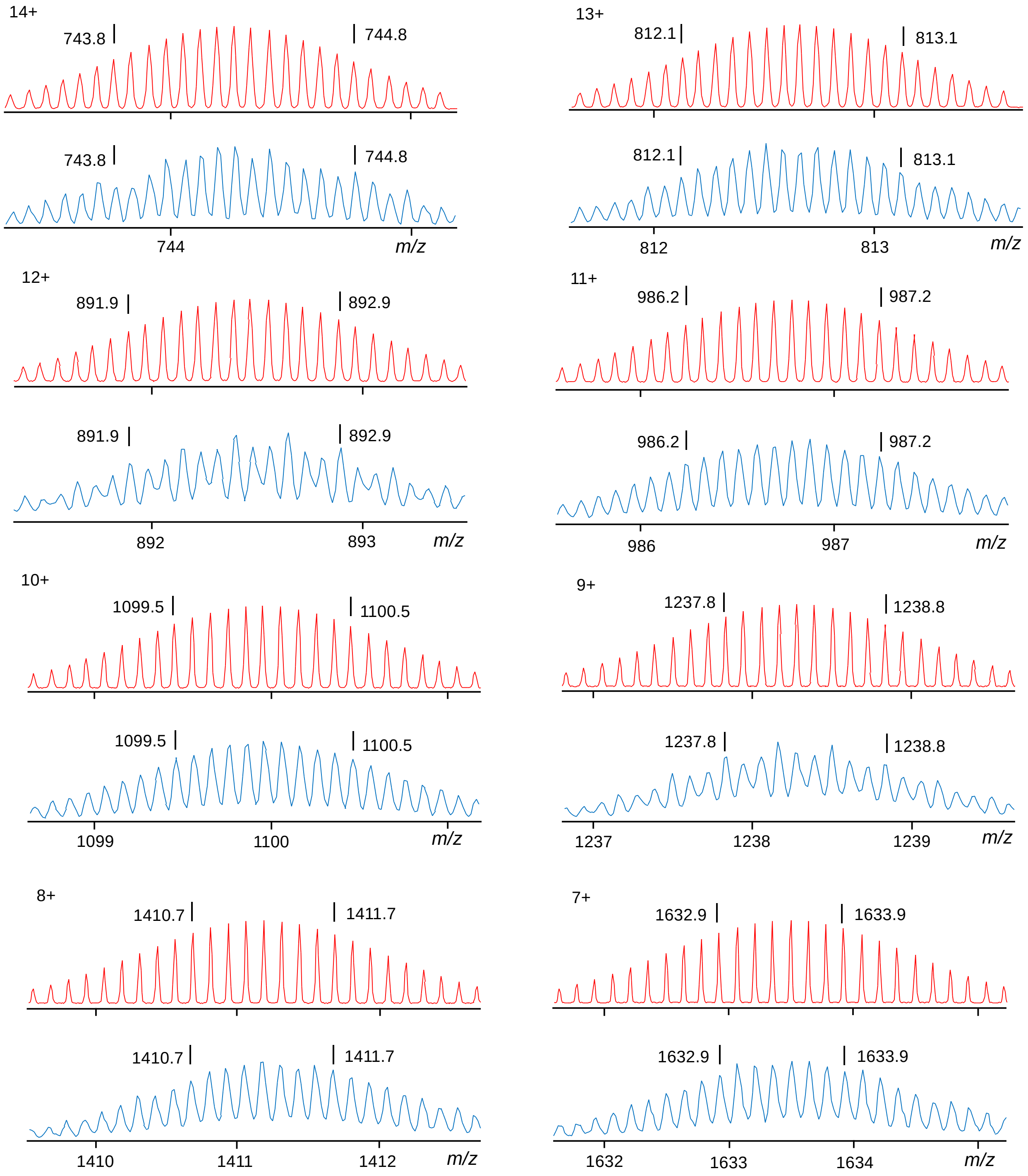


**Supplementary Figure 20.** Theoretical isotope (red) and experimental isotope (blue) for **C3**

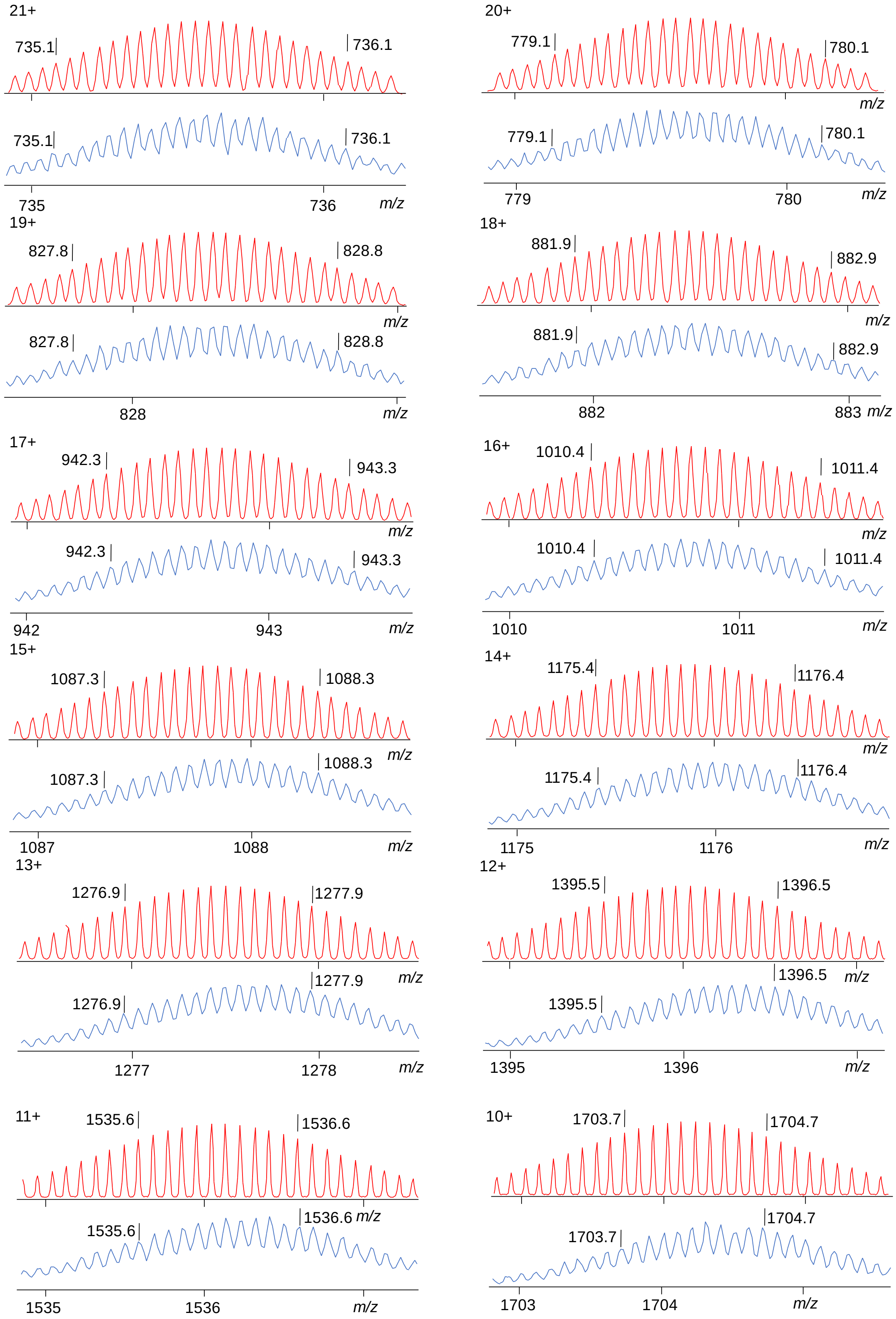


**Supplementary Figure 21.** Theoretical isotope (red) and experimental isotope (blue) for **C4**

## AFM, TEM, and STM images

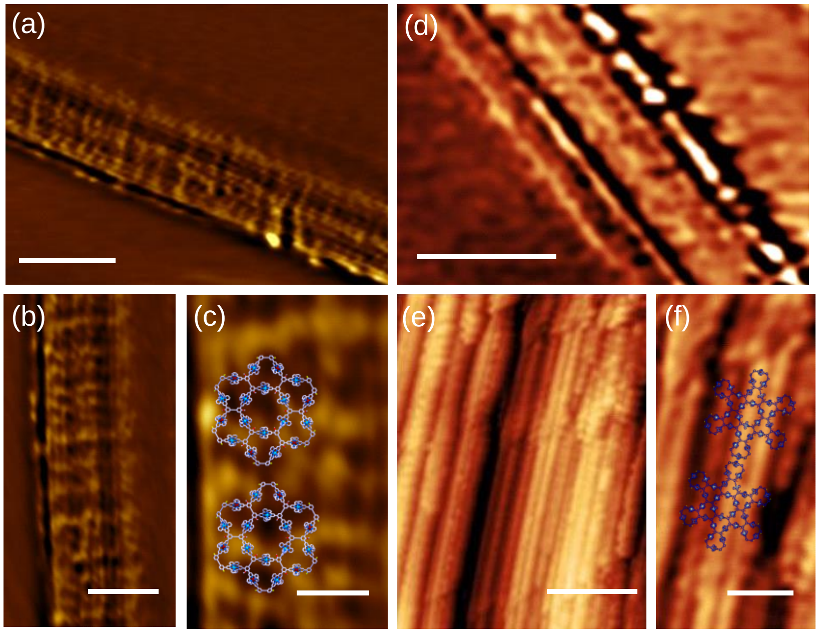


**Supplementary Figure 22.** STM image for **C4** and **C5**. **a C4** (scale bar, 20 nm); **b C4** (scale bar, 20 nm); **c C4 (**scale bar, 5 nm); **d C5** (scale bar, 10 nm); **e C5** (scale bar, 20 nm); **f C5** (scale bar, 10 nm).

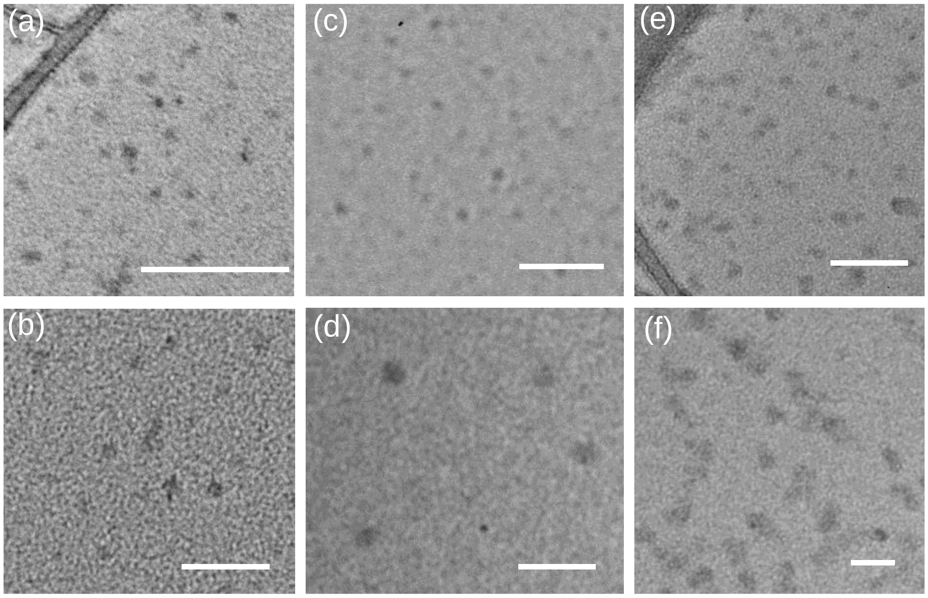


**Supplementary Figure 23.** TEM image for **C3**, **C4** and **C5**. **a C3** (scale bar, 100 nm); **b C3** (scale bar, 20 nm); **c C4** (scale bar, 100 nm); **d C4** (scale bar, 20 nm); **e C5** (scale bar, 100 nm); **f C5** (scale bar, 20 nm).

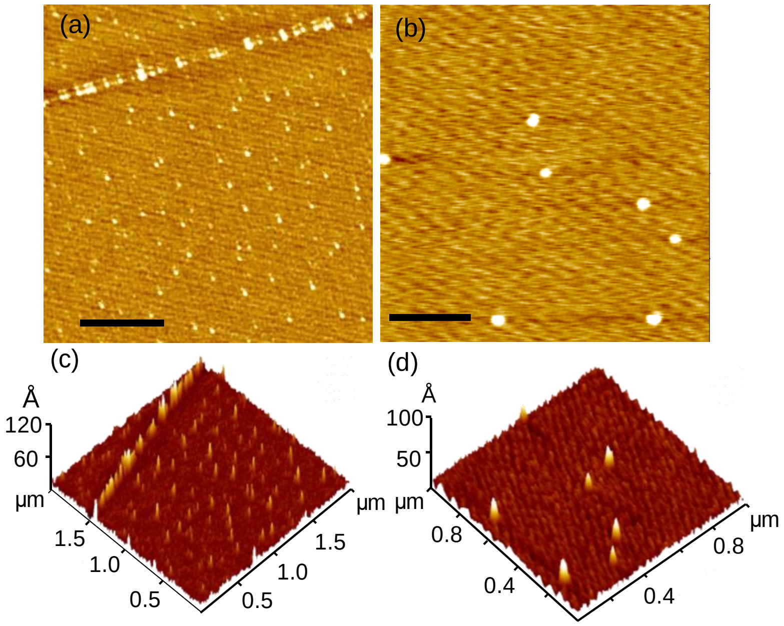


**Supplementary Figure 24.** AFM image for **C4** and **C5.** 2D AFM image of **a C4** (scale bar, 500 nm); **b C5** (scale bar, 250 nm). 3D AFM images of **c C4** and **d C5**.

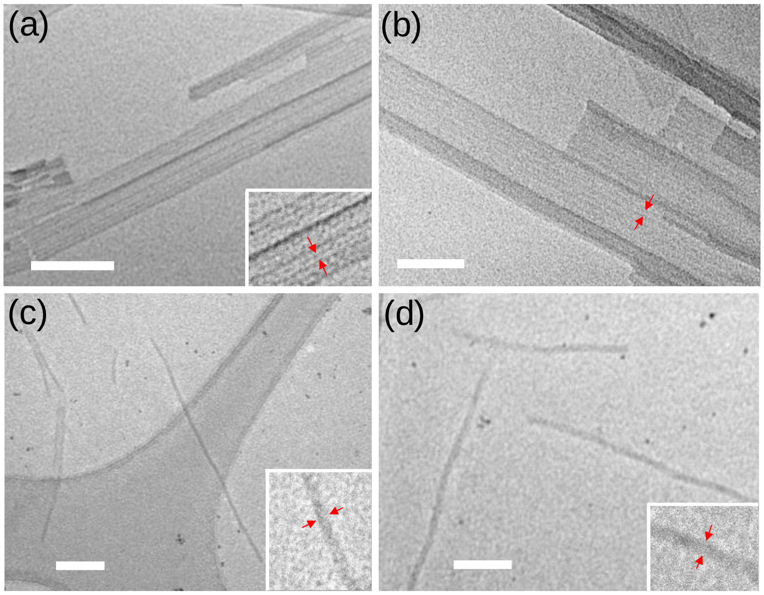


**Supplementary Figure 25.** TEM image and zoom-in images for nanotubes of **C4** and **C5. a C4** (scale bar, 100 nm); **b C4** (scale bar, 40 nm); **c C5** (scale bar, 40 nm); **d C5** (scale bar, 40 nm).

**Experimental and theoretical collision cross sections**

| | Drift Time (ms) | CCS [$Å^2$] | CCS average [$Å^2$] | CCS (calcd. avg) [$Å^2$] |
|---|---|---|---|---|
| C2 | 5.15 (5+) | 887.5 | 901.7 (9.8) | 920.1 ± 16.5 |
| | 3.96 (6+) | 897.1 | | |
| | 3.15 (7+) | 907.6 | | |
| | 2.62 (8+) | 912.5 | | |
| | 2.22 (9+) | 904.0 | | |
| | | | | |
| C3 | 6.89 (8+) | 1723.4 | 1753.4 (28.7) | 1759.4 ± 40.3 |
| | 5.68 (9+) | 1736.9 | | |
| | 4.81 (10+) | 1741.7 | | |
| | 4.19 (11+) | 1739.3 | | |
| | 3.68 (12+) | 1787.3 | | |
| | 3.32 (13+) | 1791.6 | | |
| | | | | |
| C3’ | 5.73 (9+) | 1721.7 | 1726.9 (17.6) | 1763.5 ± 35.1 |
| | 4.82 (10+) | 1717.7 | | |
| | 4.13 (11+) | 1724.8 | | |
| | 3.58 (12+) | 1720.7 | | |
| | 3.13 (13+) | 1714.6 | | |
| | 2.87 (14+) | 1762.2 | | |
| | | | | |
| C4 | 7.28 (11+) | 2454.9 | 2442.2 (30.0) | 2470.6 ± 33.0 |
| | 6.28 (12+) | 2461.1 | | |
| | 5.40 (13+) | 2441.9 | | |
| | 4.74 (14+) | 2436.3 | | |
| | 4.19 (15+) | 2426.7 | | |
| | 3.75 (16+) | 2422.8 | | |
| | 3.31 (17+) | 2387.5 | | |
| | 3.09 (18+) | 2424.9 | | |
| | 2.87 (19+) | 2446.9 | | |
| | 2.65 (20+) | 2452.2 | | |
| | 2.54 (21+) | 2508.6 | | |
| | | | | |
| C5 | 6.84 (23+) | 4896.2 | 4821.1 (41) | 4880.2 ± 169.2 |
| | 6.28 (24+) | 4851.2 | | |
| | 5.84 (25+) | 4831.1 | | |
| | 5.51 (26+) | 4846.3 | | |
| | 5.07 (27+) | 4777.9 | | |
| | 4.85 (28+) | 4819.6 | | |
| | 4.52 (29+) | 4775.3 | | |
| | 4.30 (30+) | 4787.1 | | |

| | | | | |
|---|---|---|---|---|
| | 4.08 (31+) | 4785.0 | | |
| | 3.86 (32+) | 4768.3 | | |
| | 3.75 (33+) | 4828.2 | | |
| | 3.68 (34+) | 4881.3 | | |
| | 3.62 (35+) | 4827.4 | | |

**Supplementary Table 1**. Experimental collision cross section and theoretical collision cross section of supramolecules **C2-C5**.

## NMR spectra for ligands L1-L5

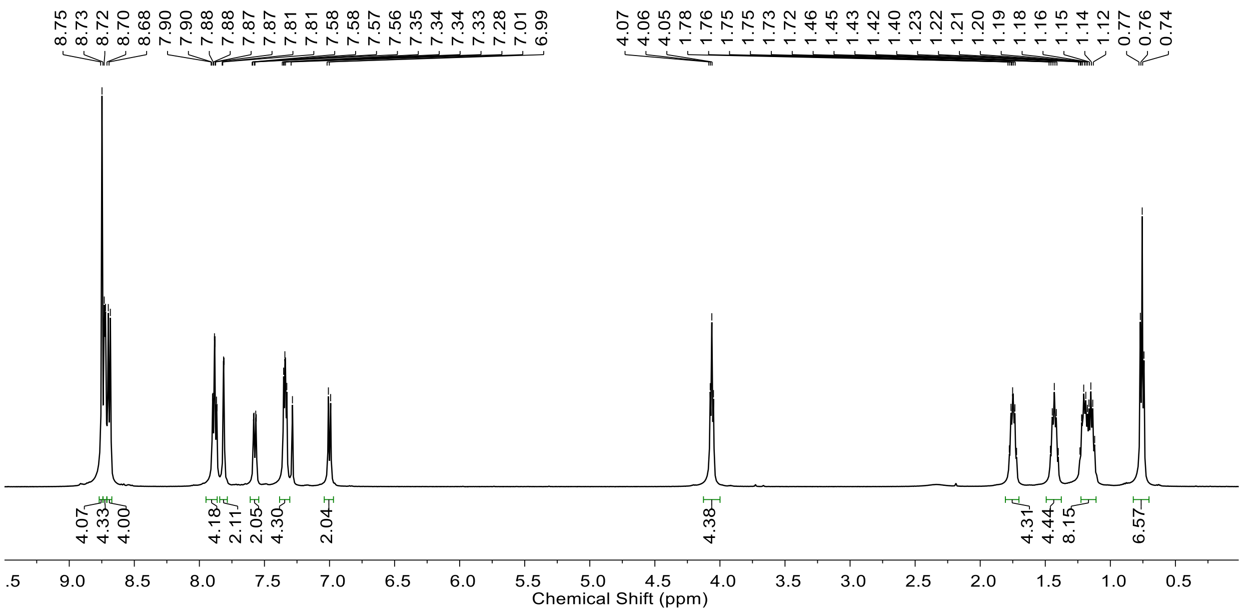


**Supplementary Figure 26.** $^{1}$H NMR spectrum (500 MHz, $CDCl_3$, 300K) for ligand **L1**

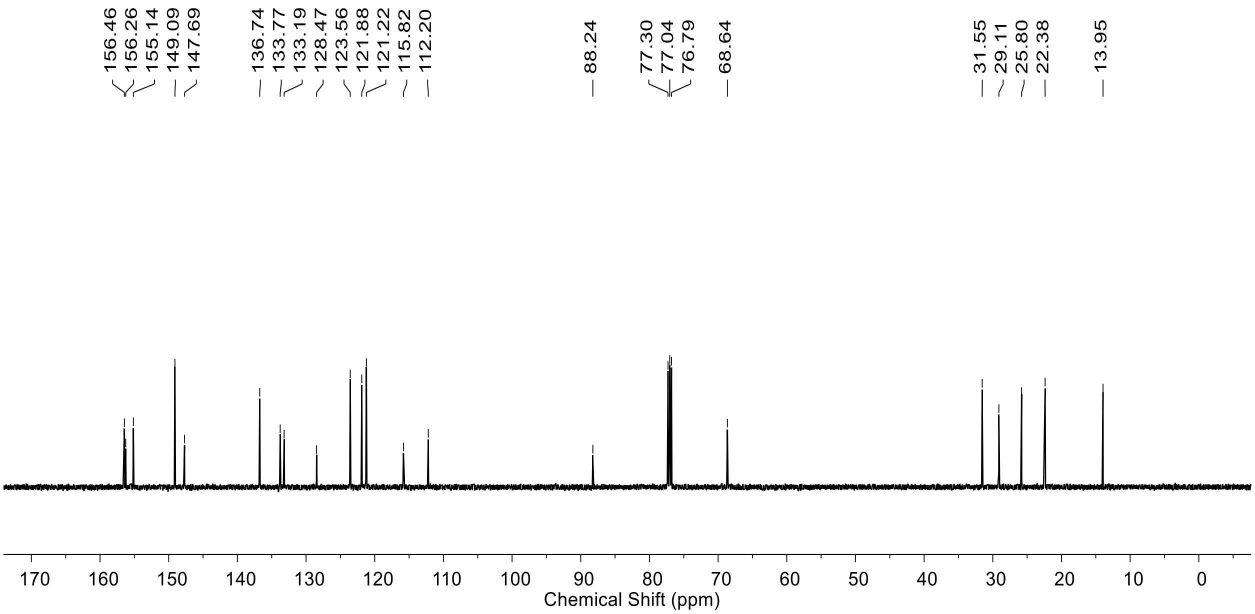


**Supplementary Figure 27.** $^{13}$C NMR spectrum (126 MHz, $CDCl_3$, 300K) for ligand **L1**

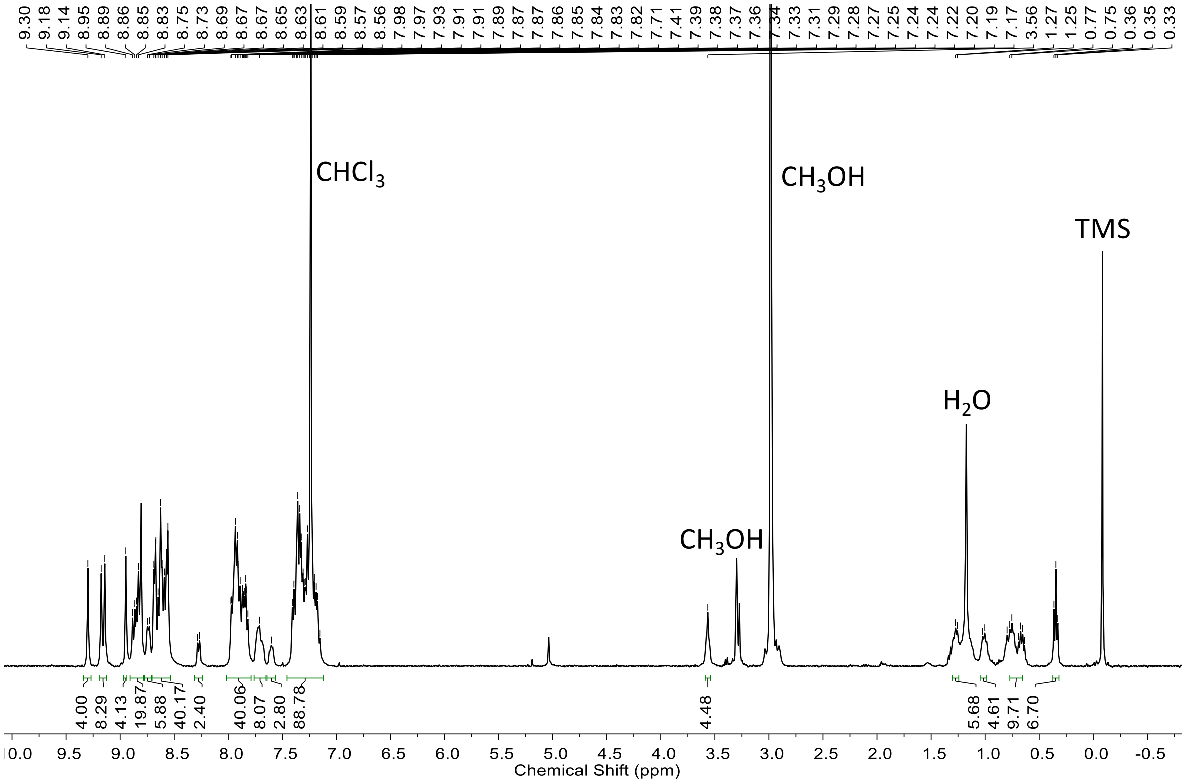


**Supplementary Figure 28.** $^{1}H$ NMR spectrum (400 MHz, $CDCl_3$+10% $CD_3OD$, 300K) for ligand **L2**

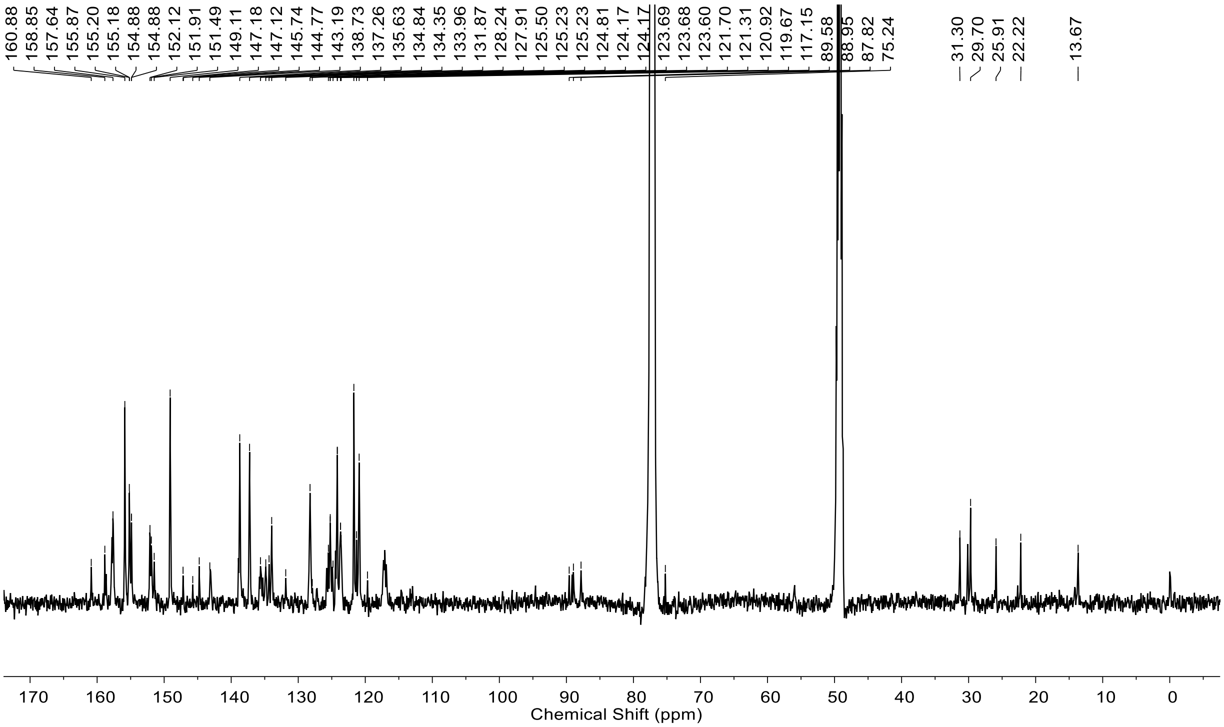


**Supplementary Figure 29.** $^{13}C$ NMR spectrum (125 MHz, $CDCl_3$+10% $CD_3OD$, 300K) for ligand **L2**

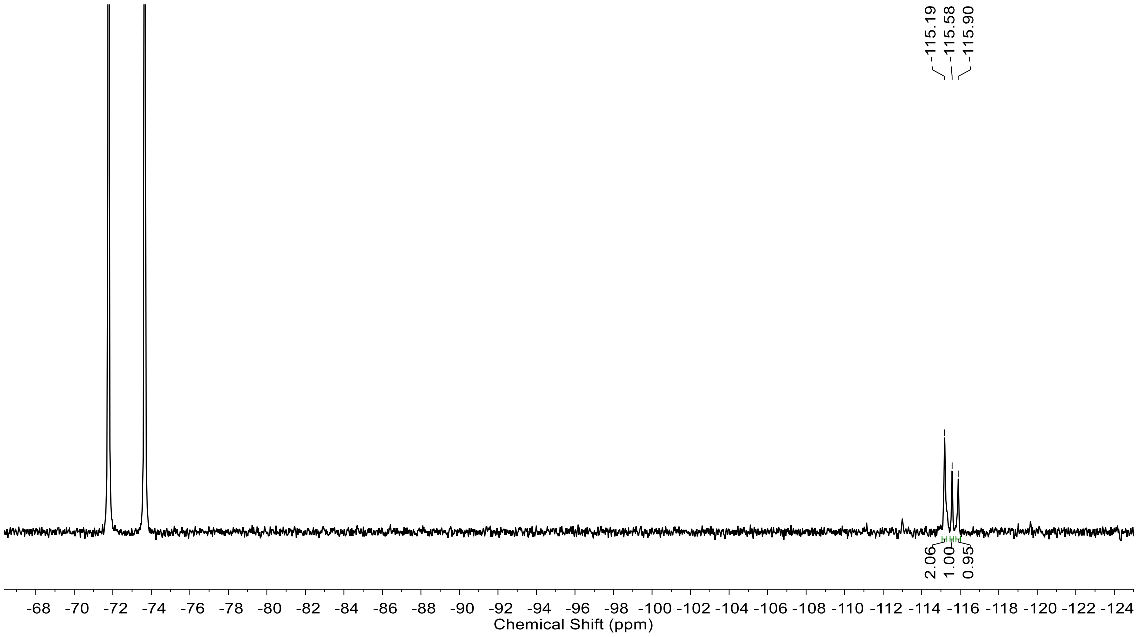


**Supplementary Figure 30.** $^{19}F$ NMR spectrum (376 MHz, $CD_3CN$, 300K) for ligand **L2**

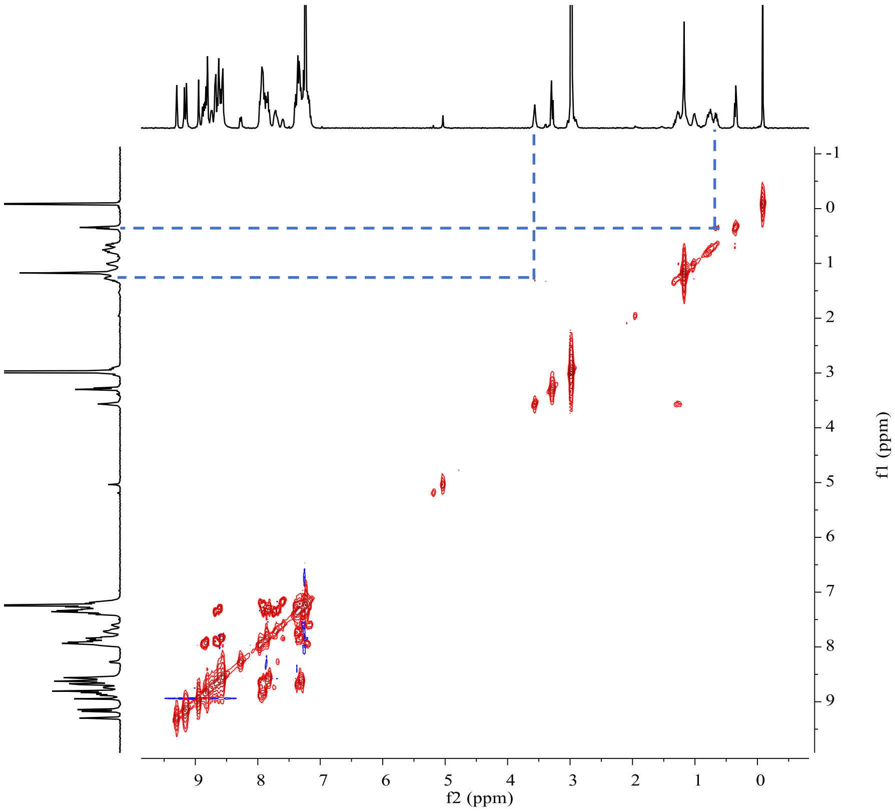


**Supplementary Figure 31.** 2D COSY NMR spectrum (400 MHz, $CDCl_3$+10% $CD_3OD$, 300K) for ligand **L2**

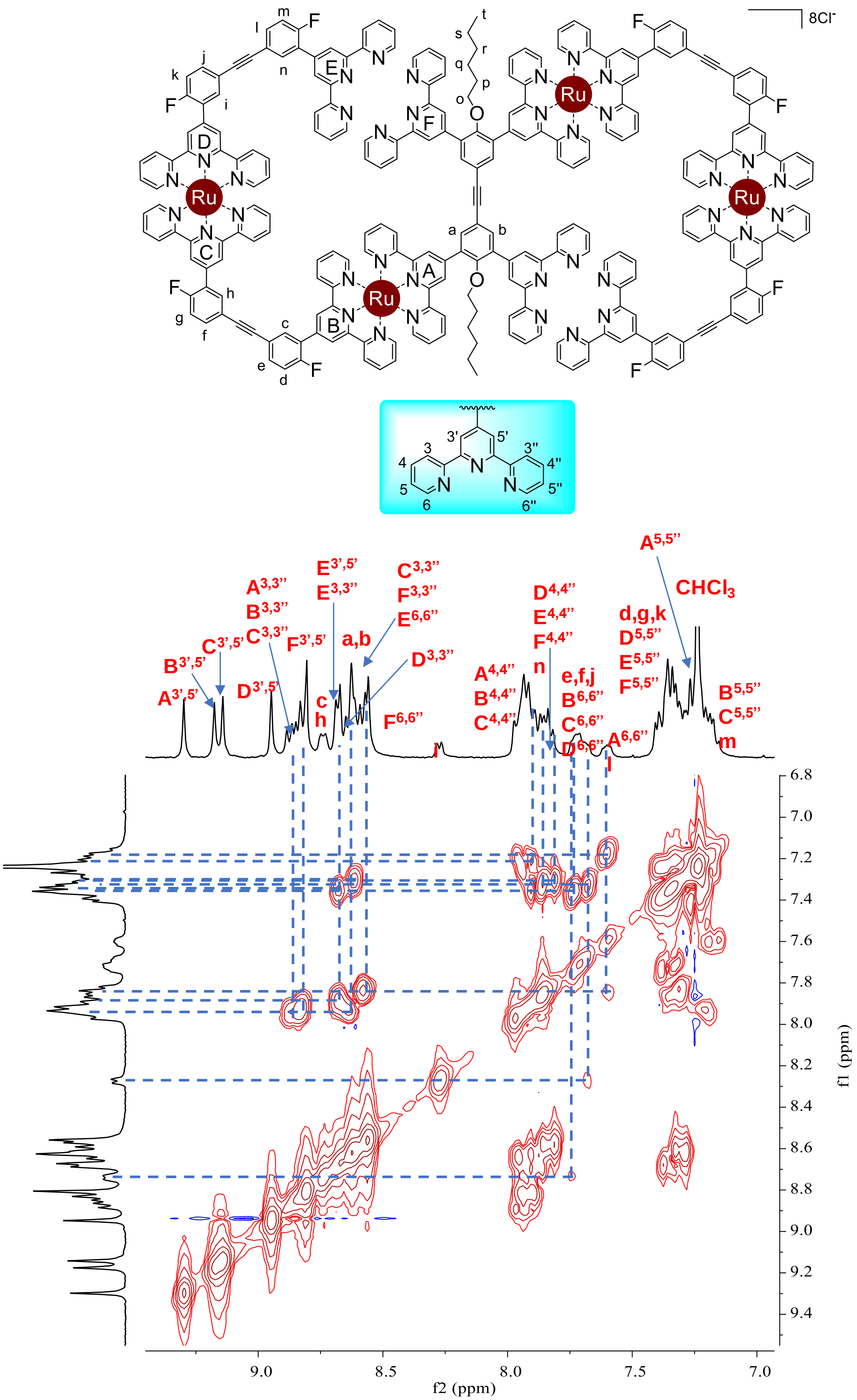


**Supplementary Figure 32.** 2D COSY NMR spectrum (400 MHz, $CDCl_3$+10% $CD_3OD$, 300K) for ligand **L2** (aromatic region).

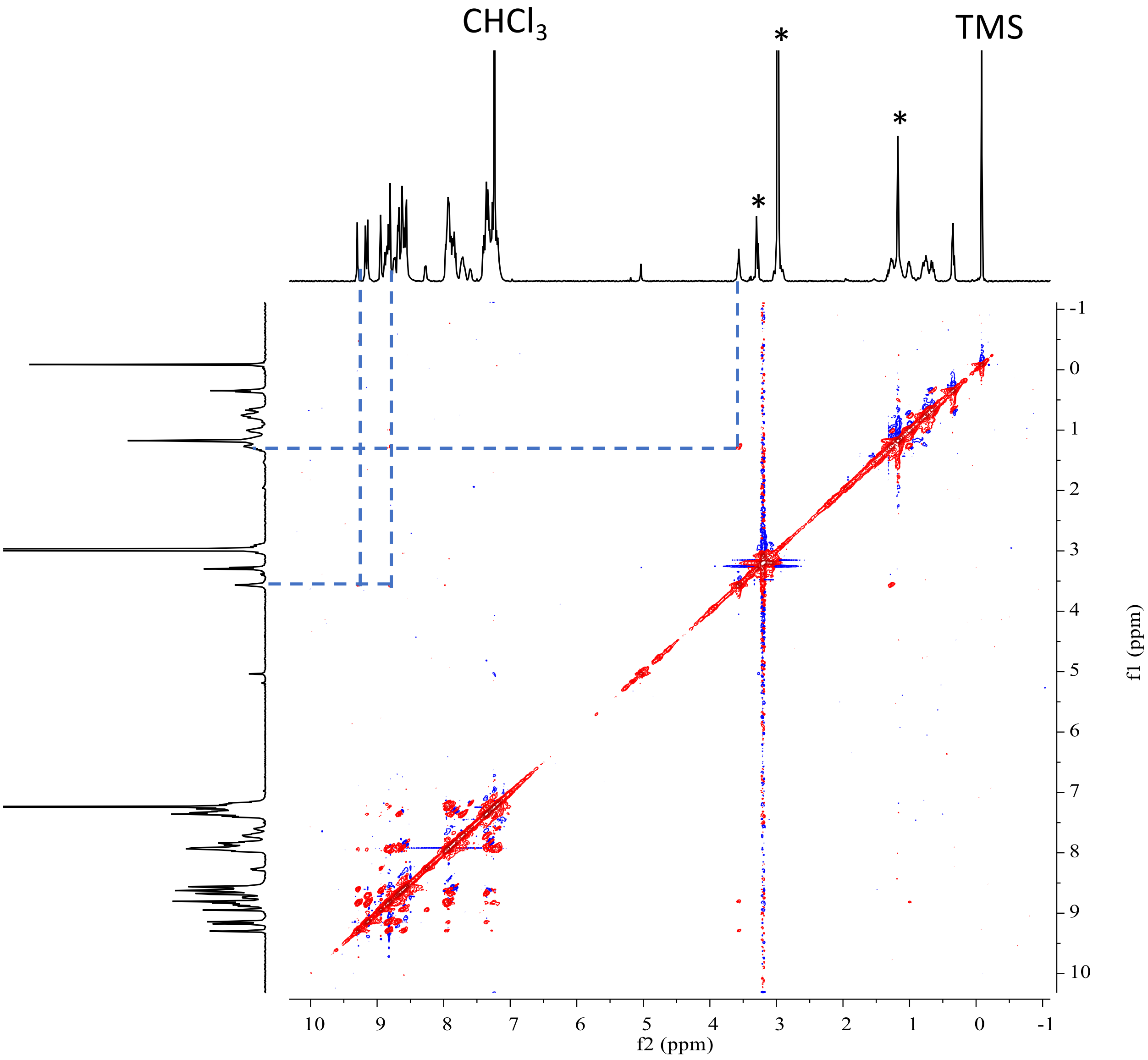


**Supplementary Figure 33.** 2D NOESY NMR spectrum (400 MHz, $CDCl_3$+10% $CD_3OD$, 300K) for ligand **L2.** ***** represent residual solvent.

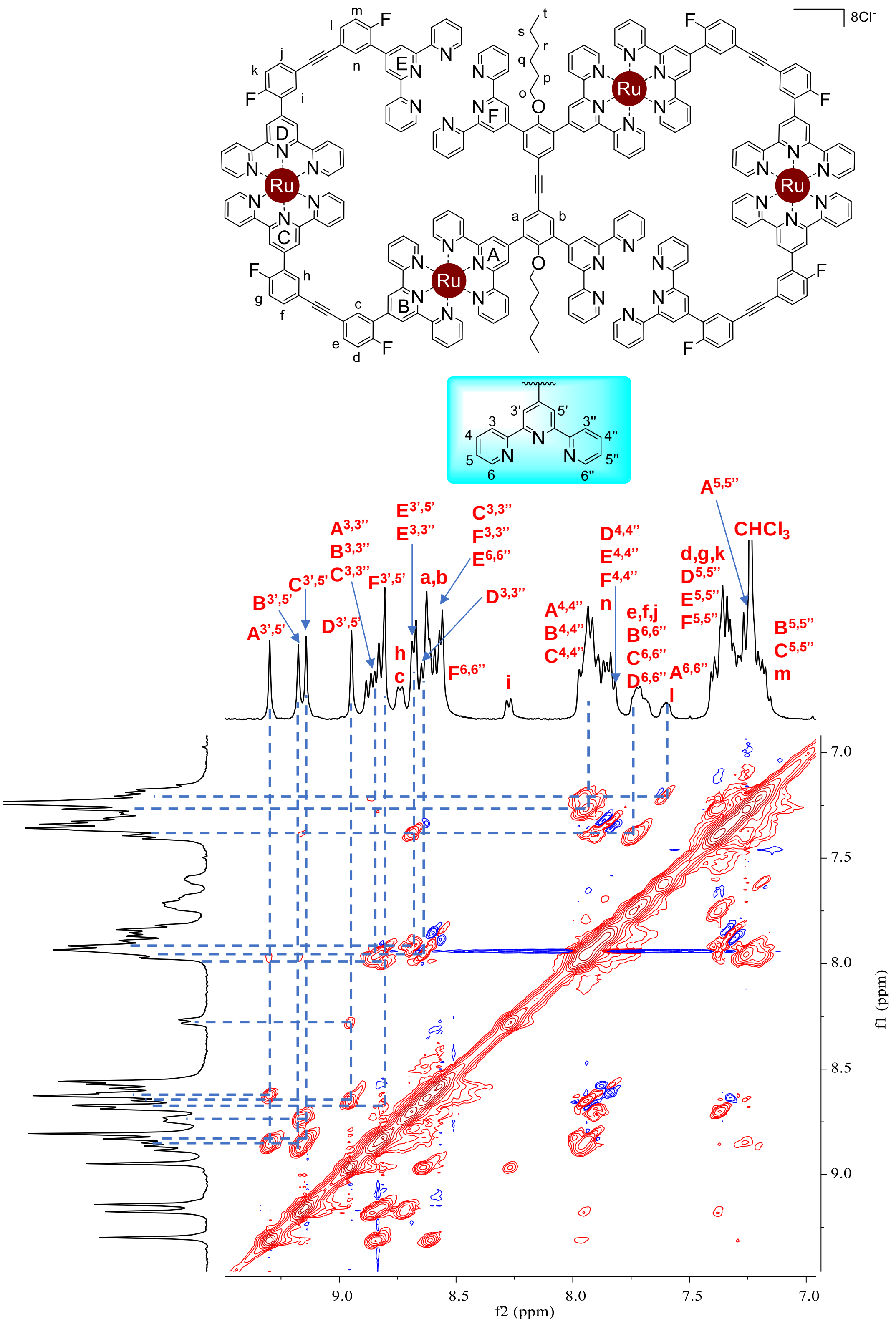

**Supplementary Figure 34.** 2D NOESY NMR spectrum (400 MHz, $CDCl_3$+10% $CD_3OD$, 300K) for ligand **L2** (aromatic region).

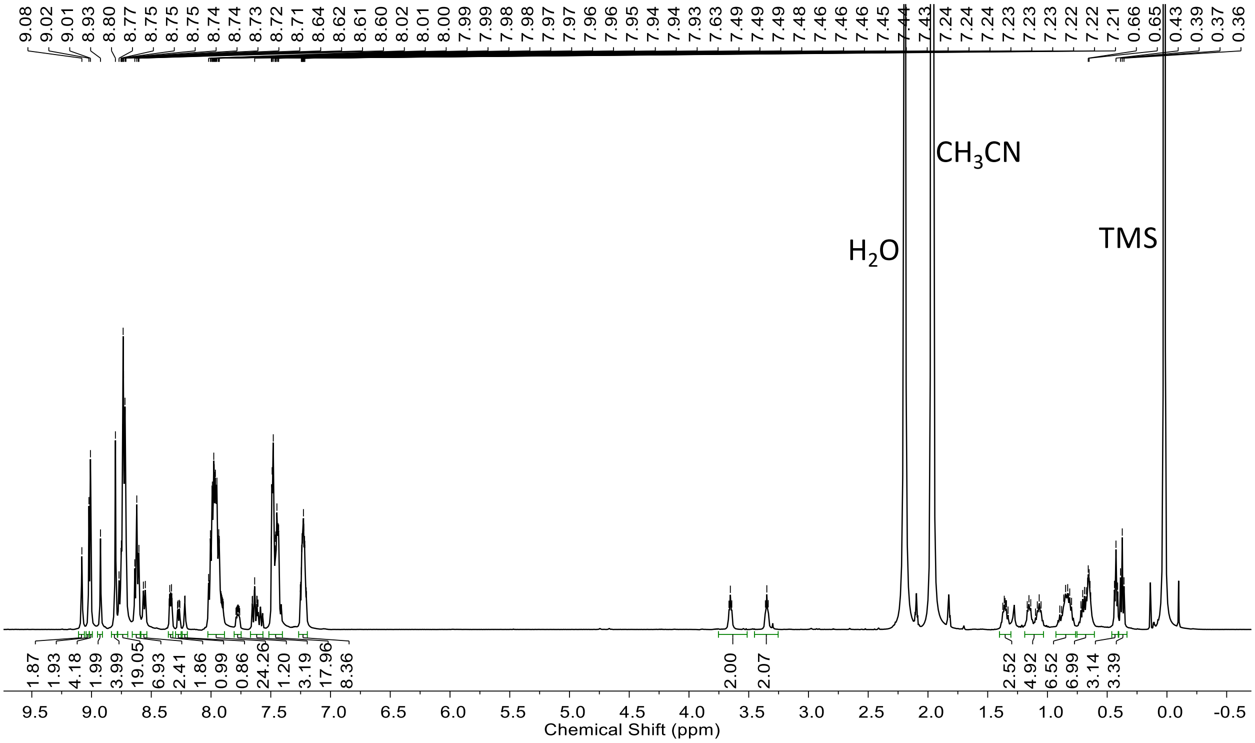


**Supplementary Figure 35.** $^{1}H$ NMR NMR spectrum (500 MHz, $CD_3CN$, 300K) for ligand **L3.**

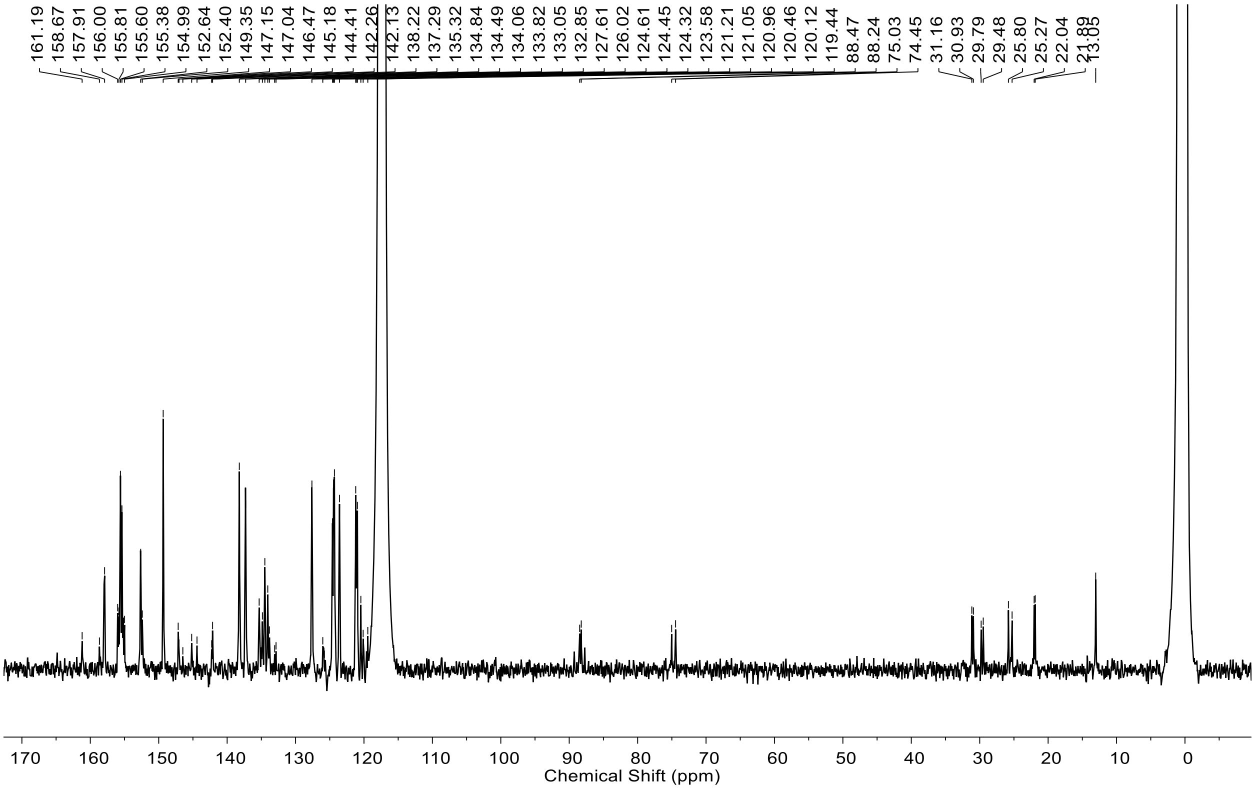


**Supplementary Figure 36.** $^{13}C$ NMR NMR spectrum (100 MHz, $CD_3CN$, 300K) for ligand **L3.**

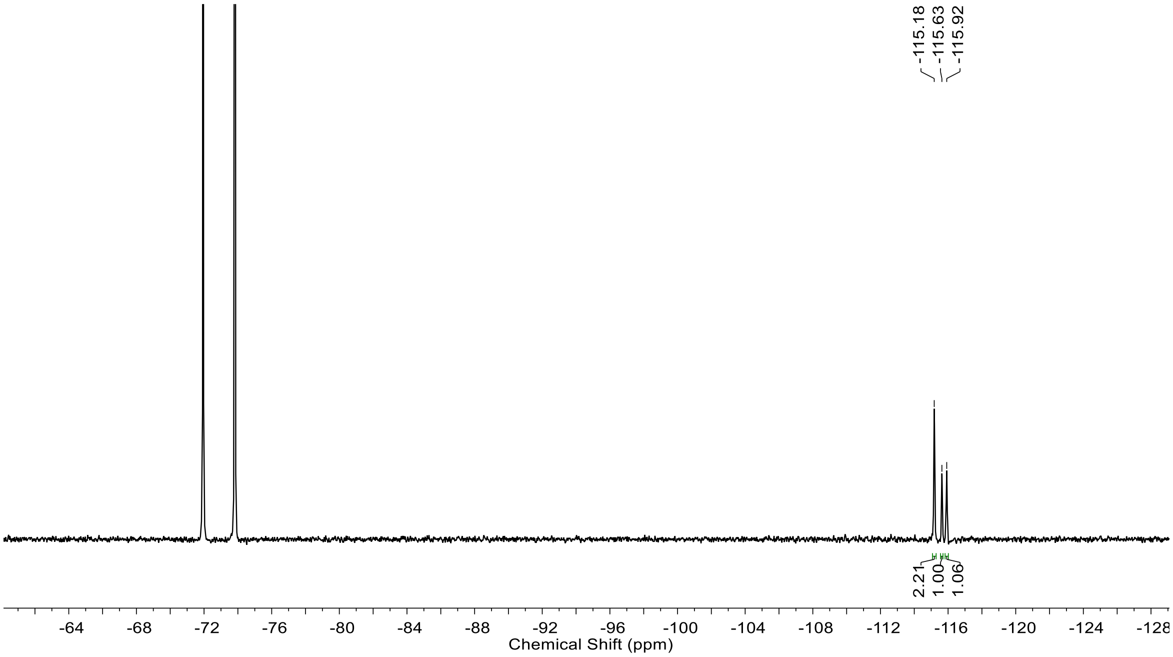


**Supplementary Figure 37.** $^{19}$F NMR NMR spectrum (376 MHz, $CD_3CN$, 300K) for ligand **L3.**

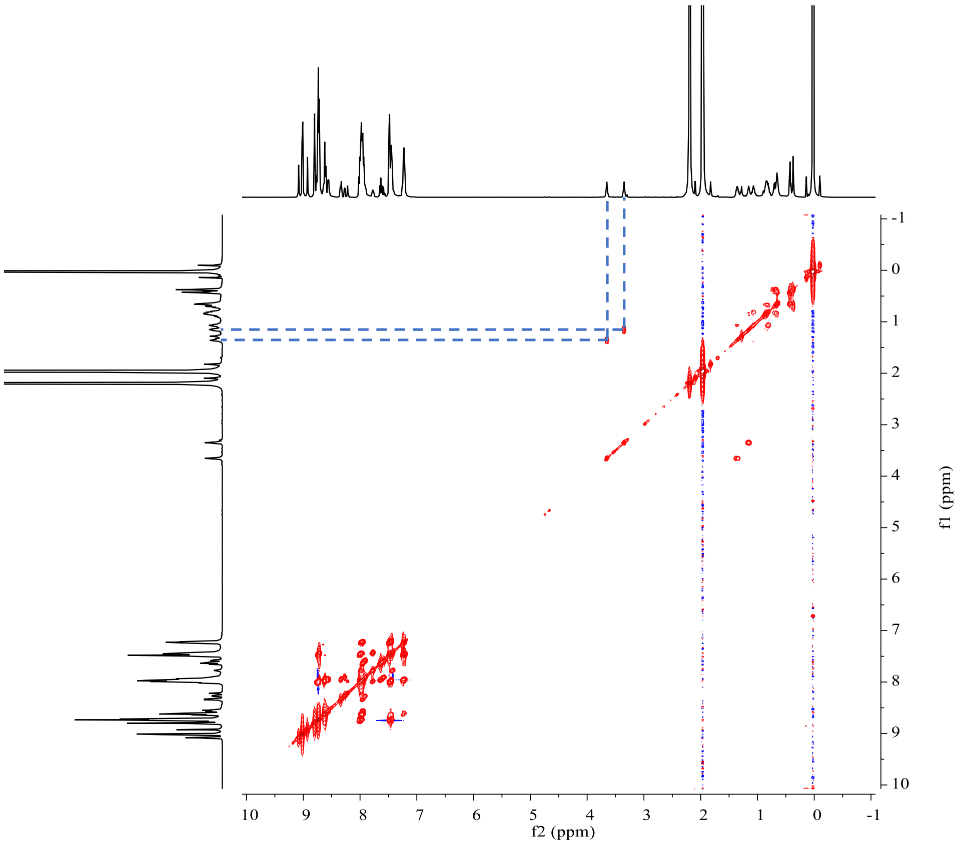


**Supplementary Figure 38.** 2D COSY NMR spectrum (500 MHz, $CD_3CN$, 300K) for ligand **L3**

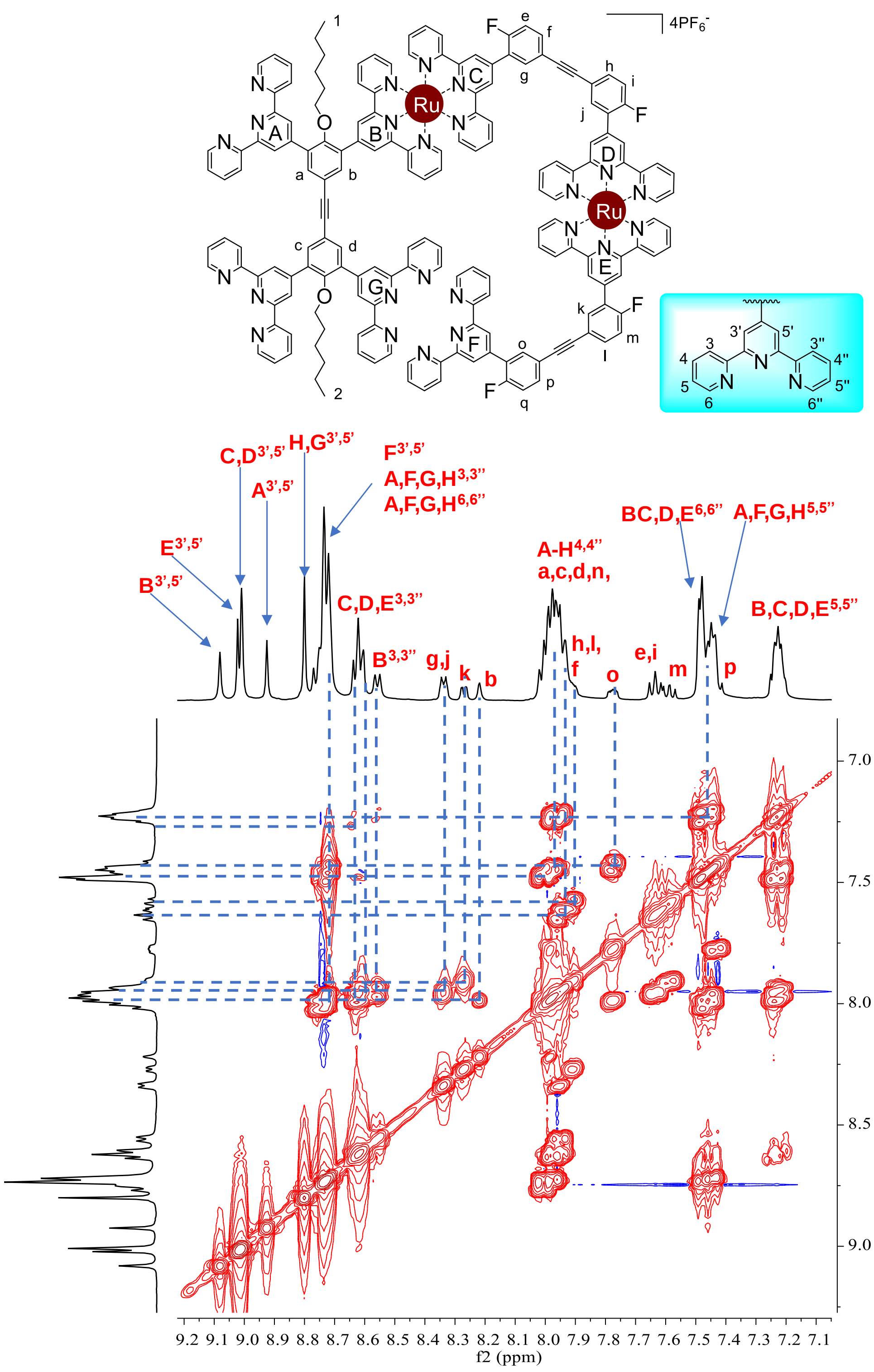


**Supplementary Figure 39.** 2D COSY NMR spectrum (500 MHz, $CD_3CN$, 300K) for ligand **L3** (aromatic region)

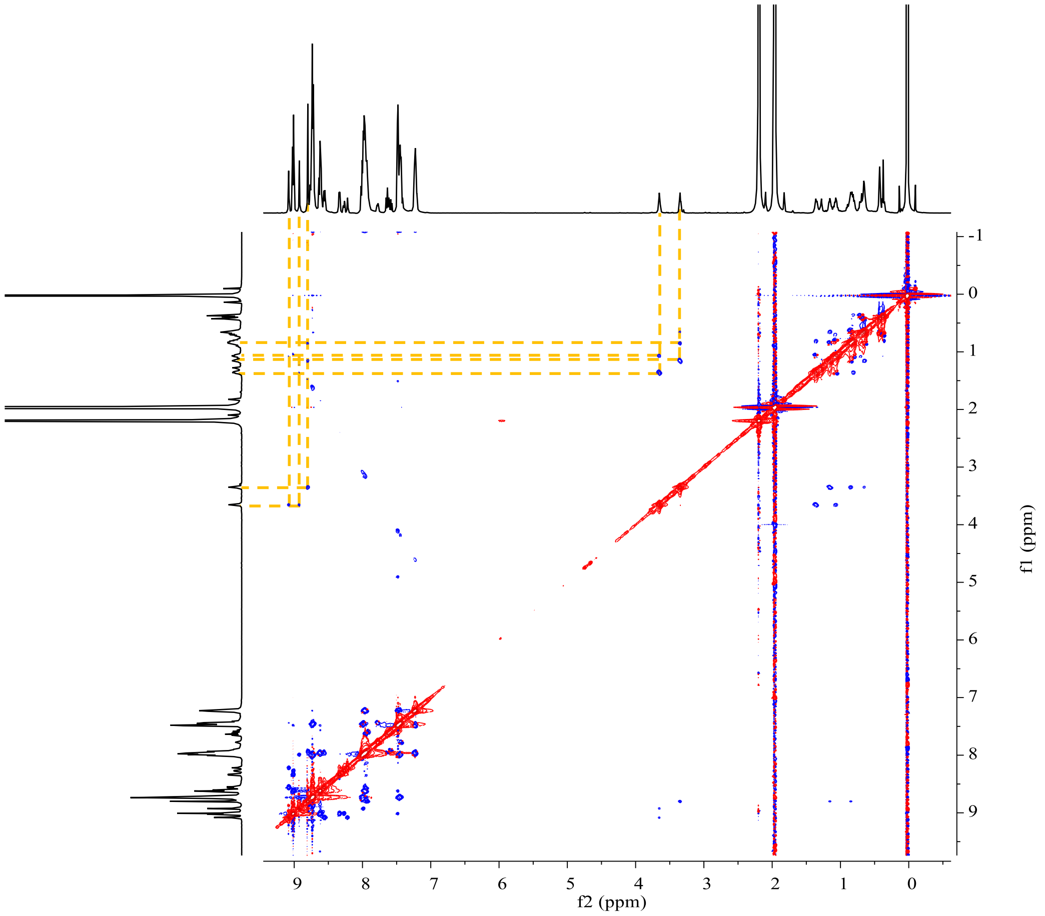


**Supplementary Figure 40.** 2D ROESY NMR spectrum (500 MHz, $CD_3CN$, 300K) for ligand **L3**

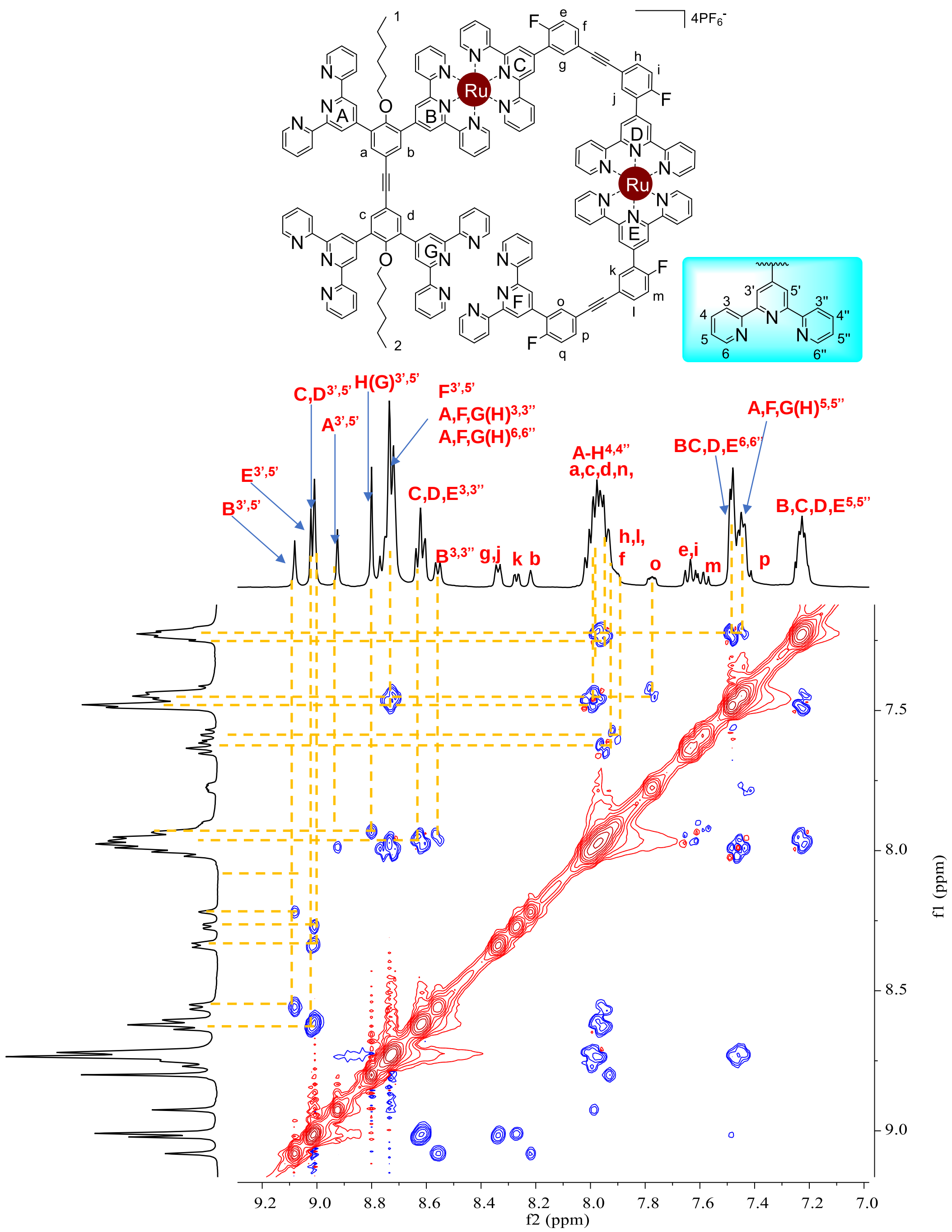

**Supplementary Figure 41.** 2D ROESY NMR spectrum (500 MHz, $CD_3CN$, 300K) for ligand **L3** (aromatic region)

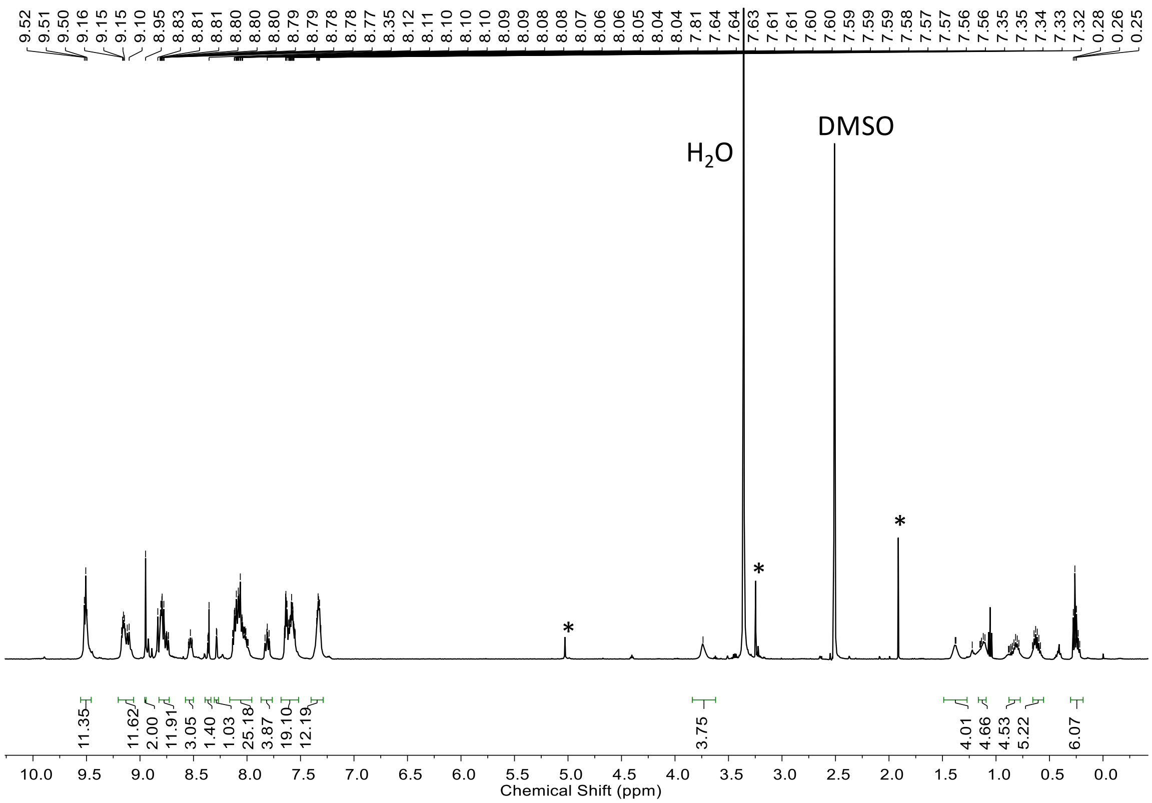


**Supplementary Figure 42.** $^{1}H$ NMR NMR spectrum (500 MHz, DMSO-$d_6$, 300K) for ligand **L3'.** * represent residue solvent.

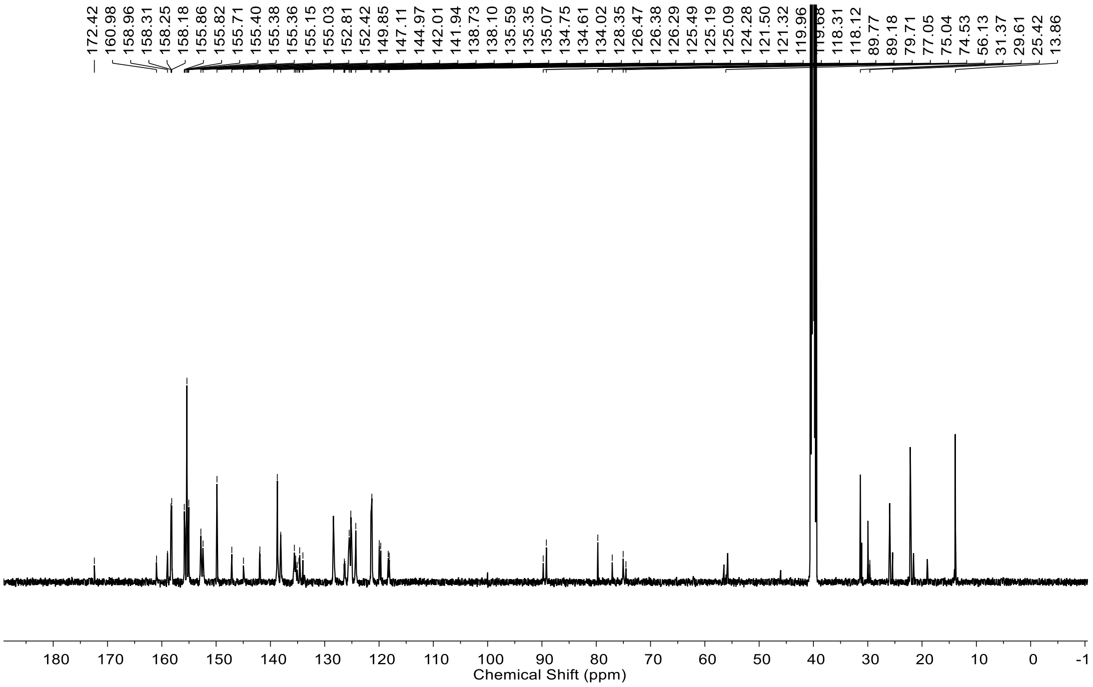


**Supplementary Figure 43.** $^{13}C$ NMR NMR spectrum (125 MHz, DMSO-$d_6$, 300K) for ligand **L3'.**

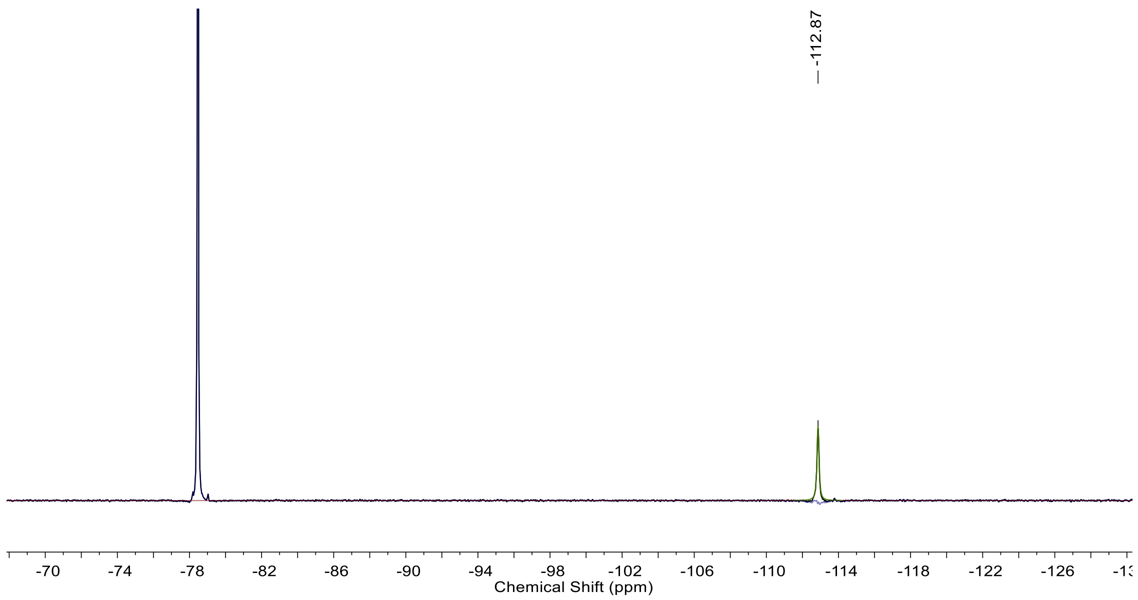


**Supplementary Figure 44.** $^{19}F$ NMR NMR spectrum (376 MHz, DMSO-$d_6$, 300K) for ligand **L3'**

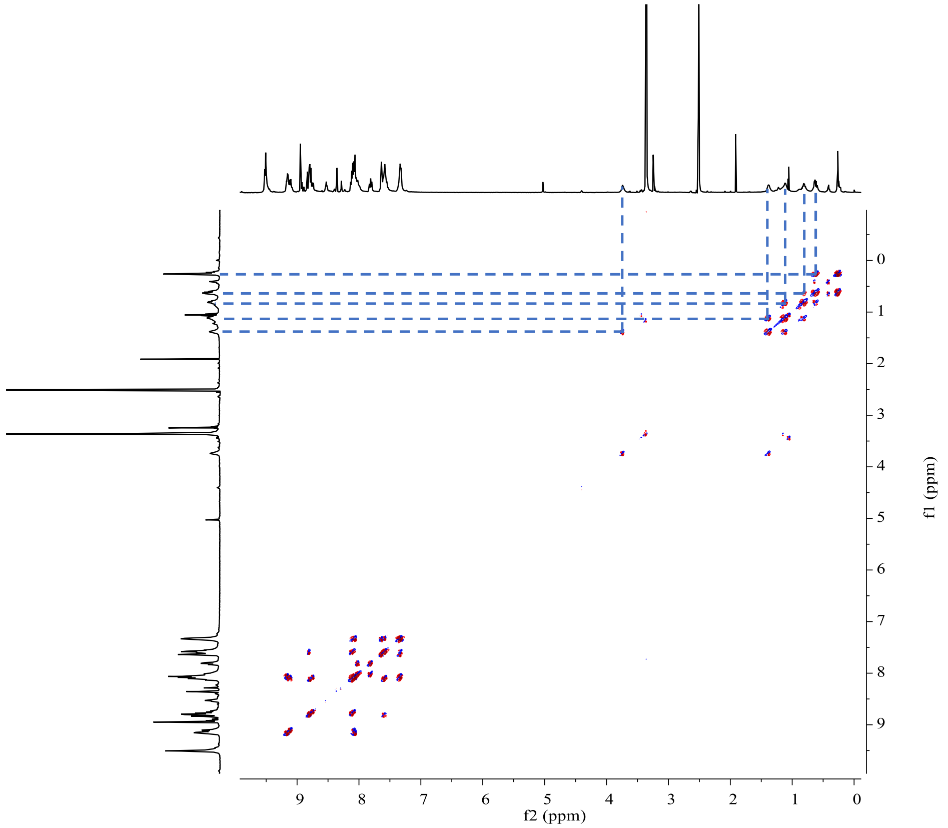


**Supplementary Figure 45.** 2D COSY NMR NMR spectrum (500 MHz, DMSO-$d_6$, 300K) for ligand **L3'**

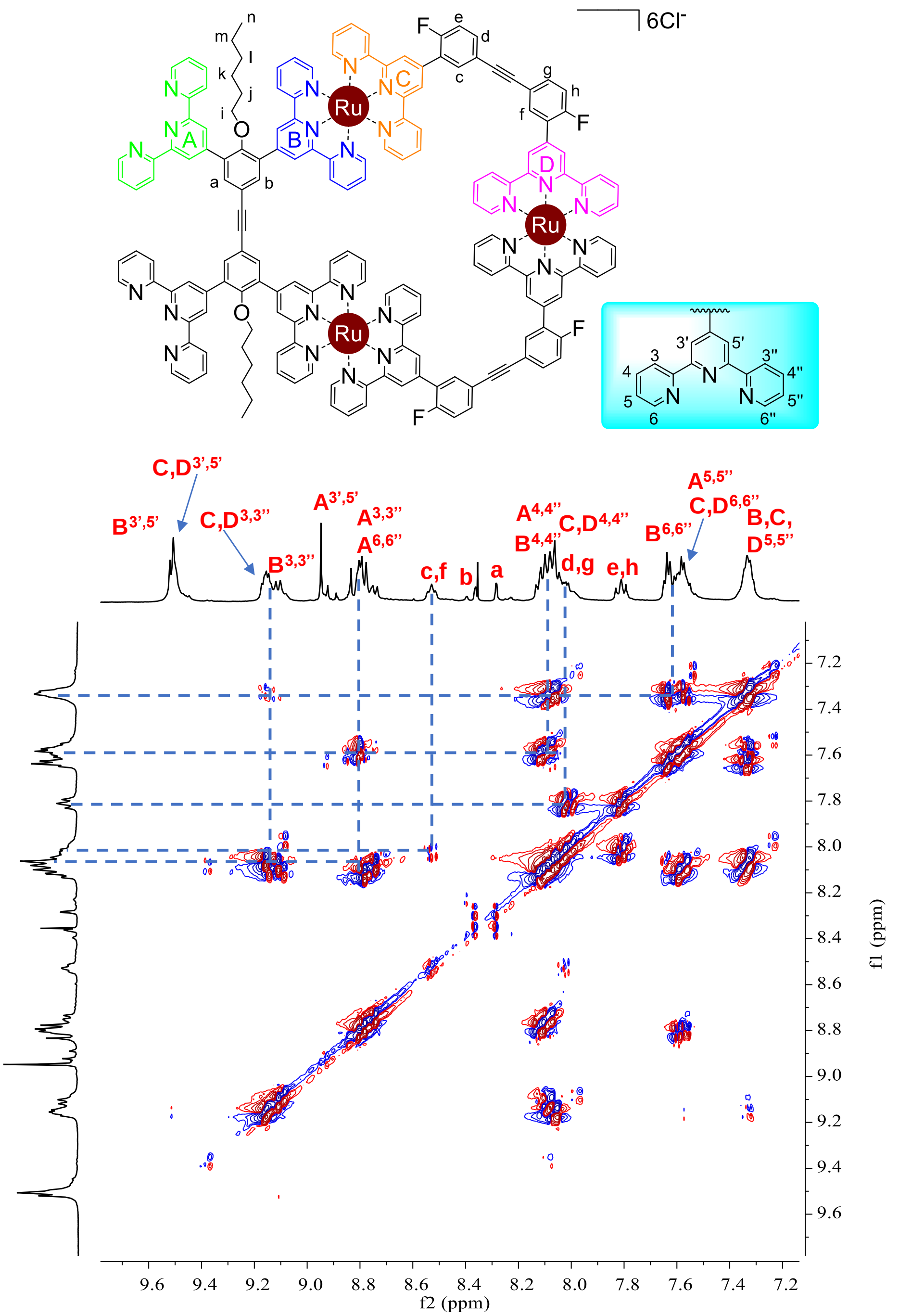


**Supplementary Figure 46.** 2D COSY NMR NMR spectrum (500 MHz, DMSO-$d_6$, 300K) for ligand **L3’** (aromatic region)

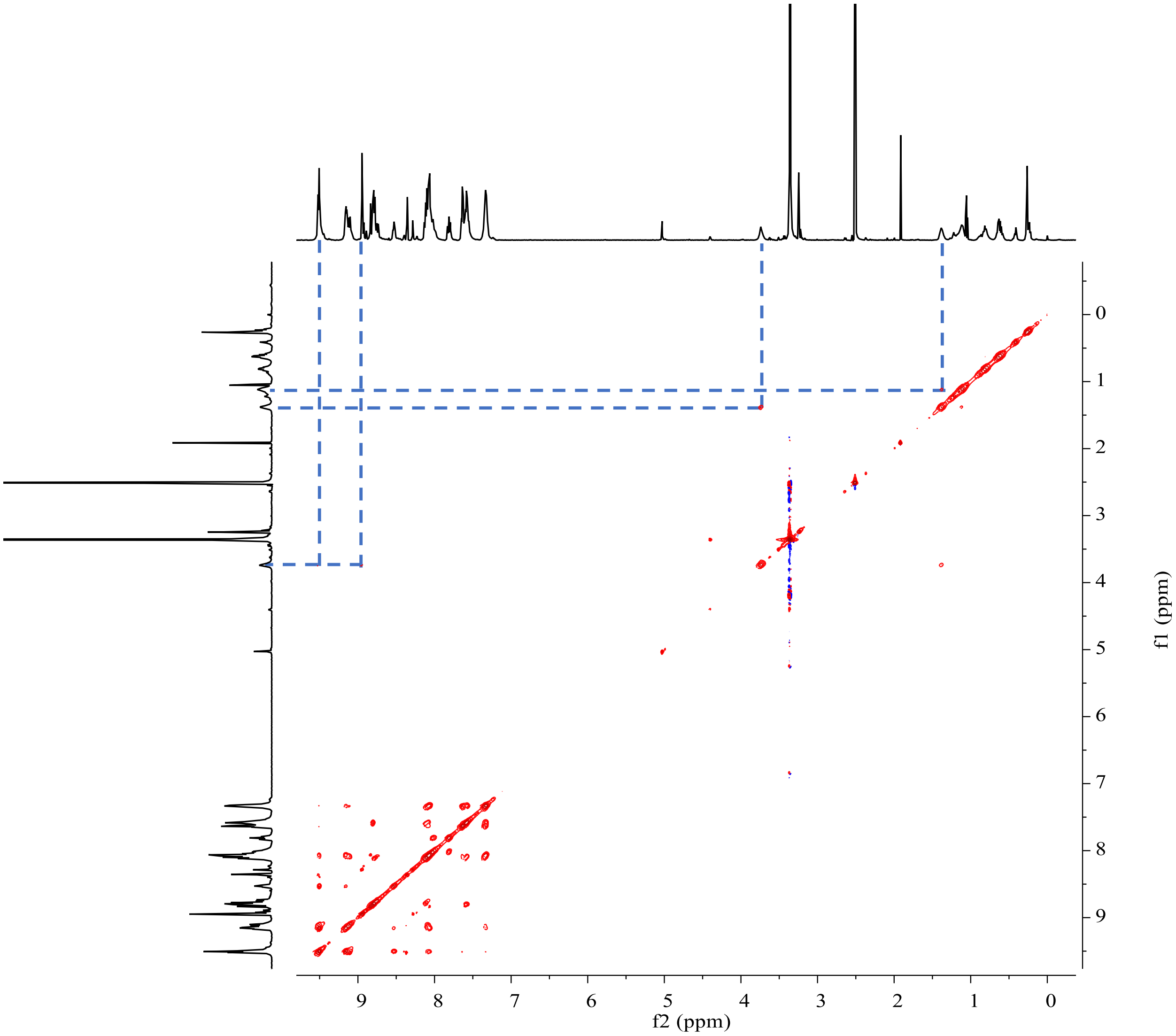


**Supplementary Figure 47.** 2D NOESY NMR spectrum (500 MHz, DMSO-$d_6$, 300K) for ligand **L3'**

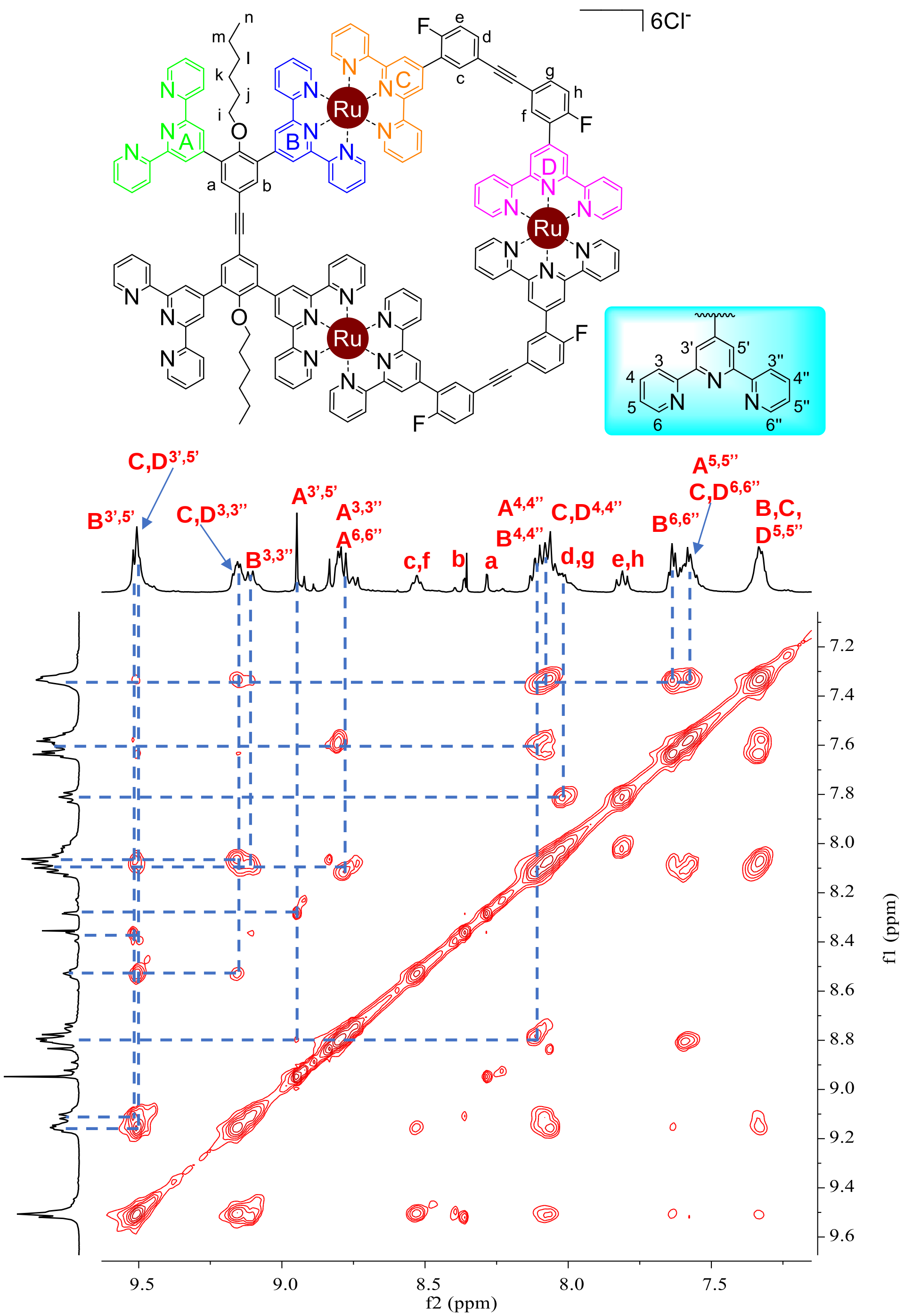


**Supplementary Figure 48.** 2D NOESY NMR NMR spectrum (500 MHz, DMSO-$d_6$, 300K) for ligand **L3'** (aromatic region)

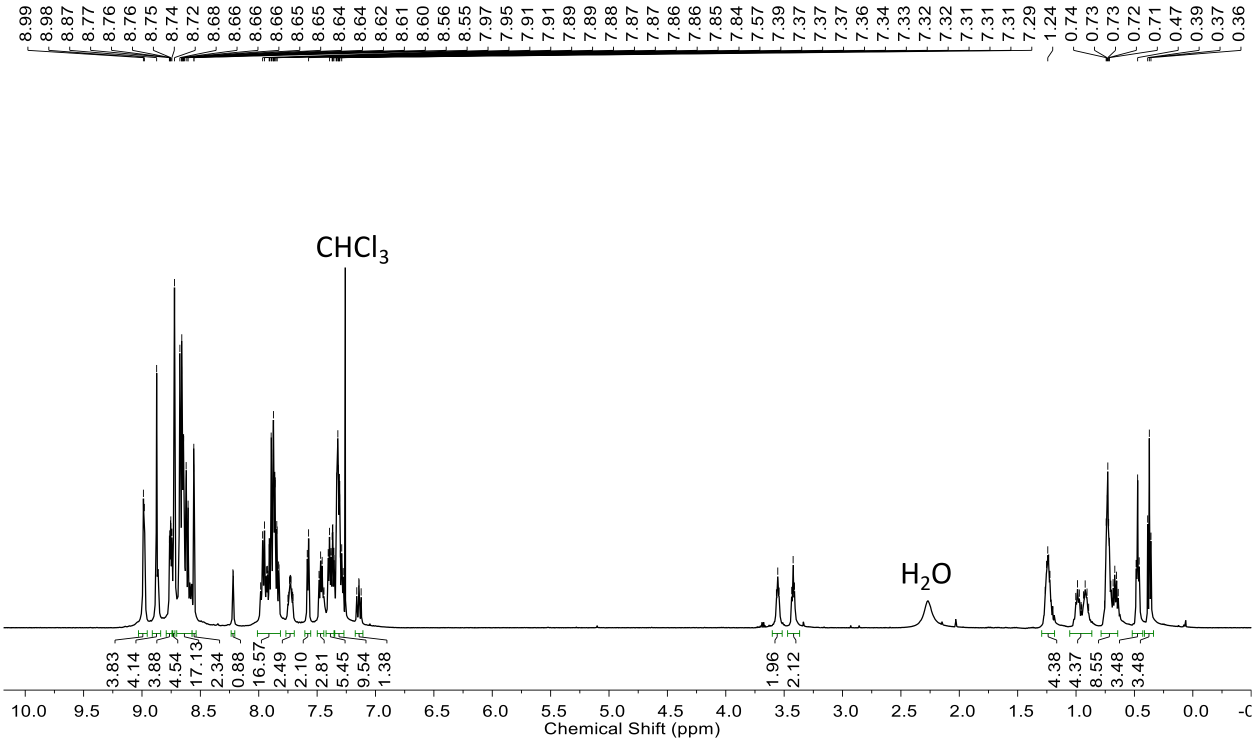


**Supplementary Figure 49.** $^{1}$H NMR NMR spectrum (500 MHz, $CDCl_3$, 300K) for ligand **L4**.

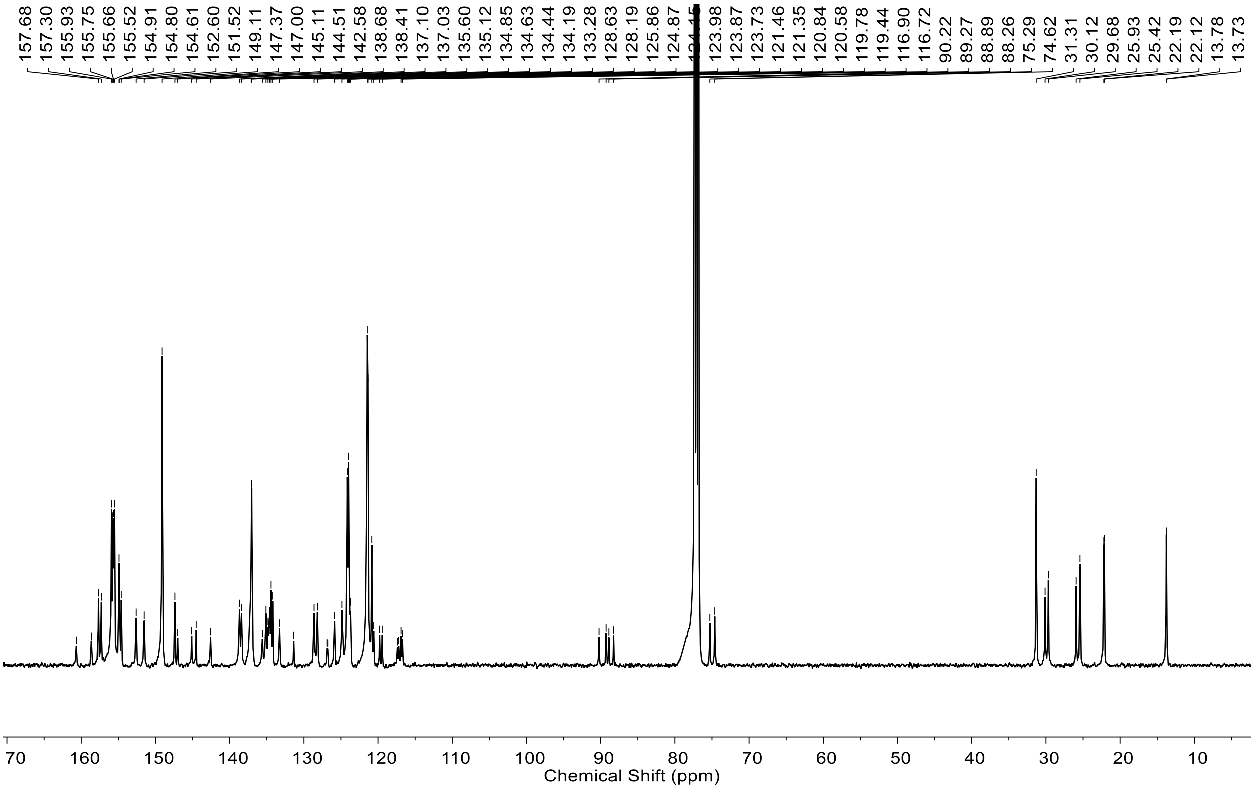


**Supplementary Figure 50.** $^{13}$C NMR NMR spectrum (125 MHz, $CDCl_3$, 300K) for ligand **L4**.

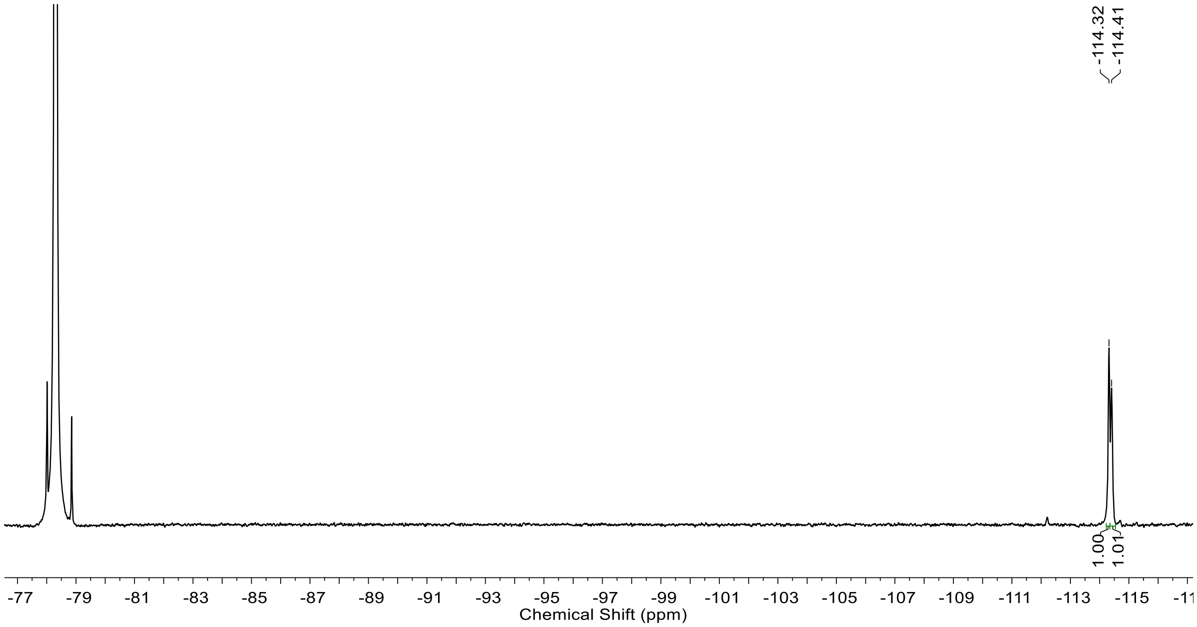


**Supplementary Figure 51.** $^{19}F$ NMR NMR spectrum (376 MHz, $CDCl_3$, 300K) for ligand **L4**

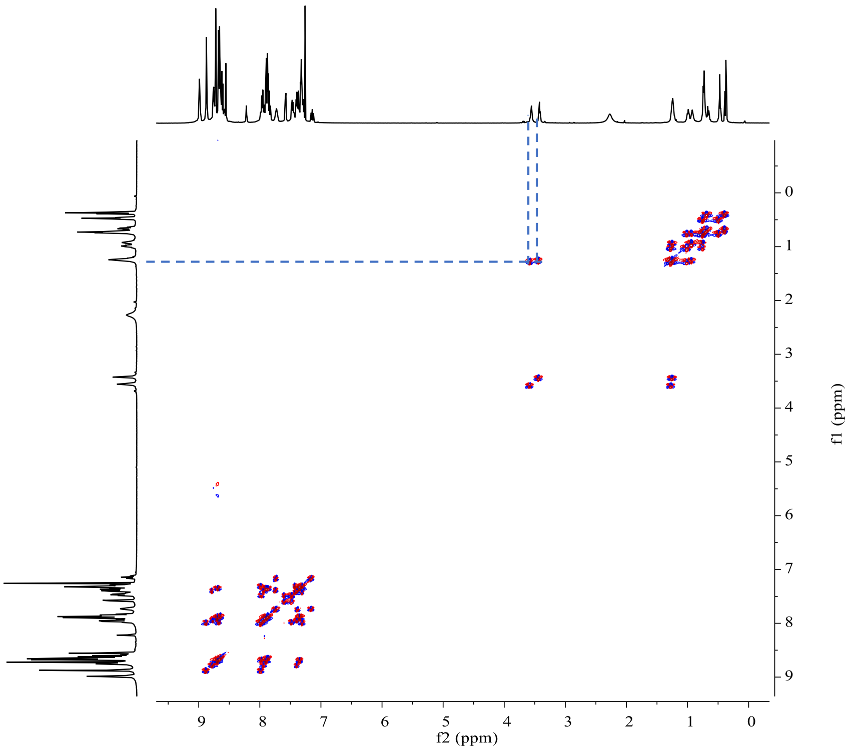


**Supplementary Figure 52.** 2D COSY NMR NMR spectrum (500 MHz, $CDCl_3$, 300K) for ligand **L4**

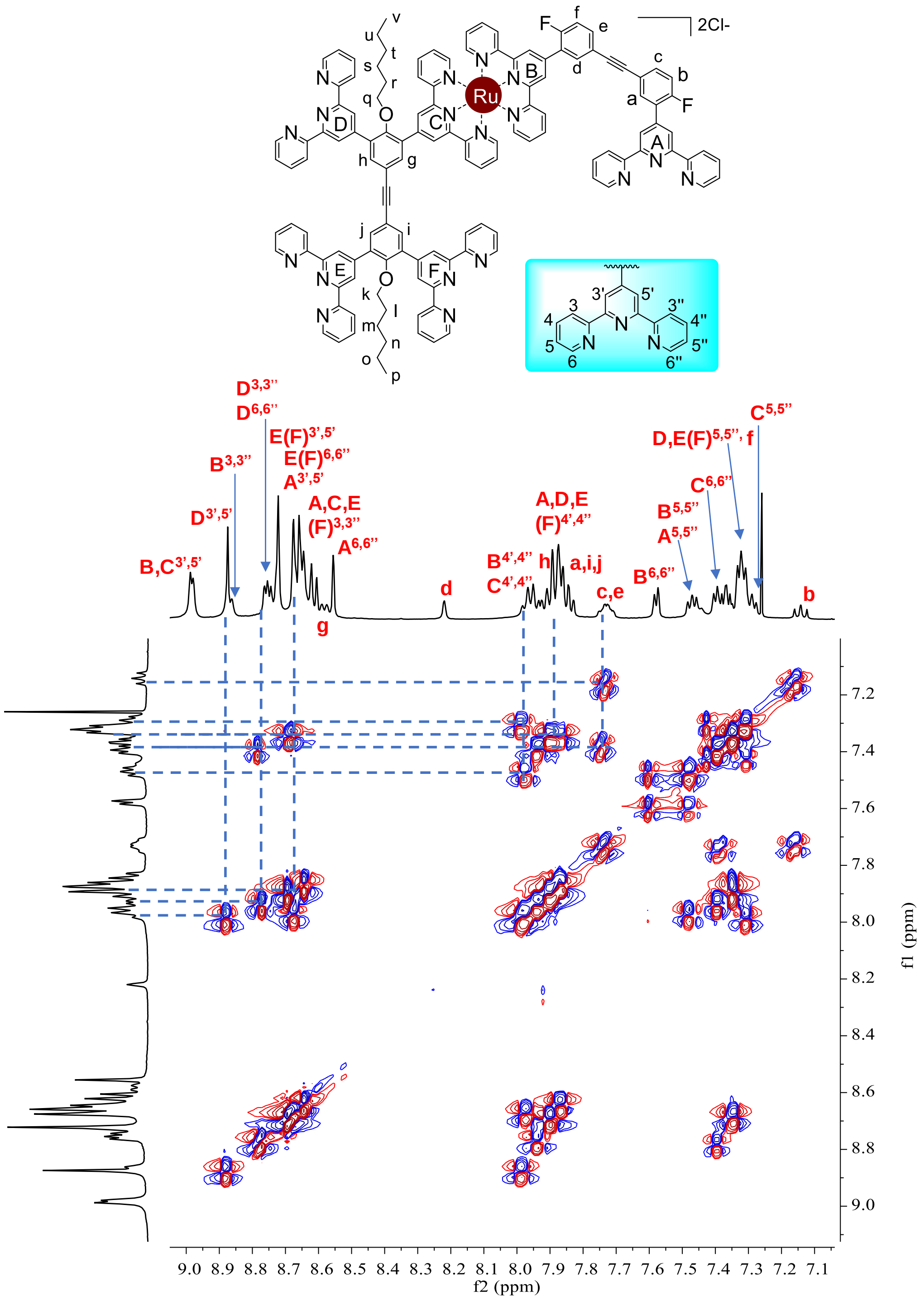


**Supplementary Figure 53.** 2D COSY NMR NMR spectrum (500 MHz, $CDCl_3$, 300K) for ligand **L4** (aromatic region).

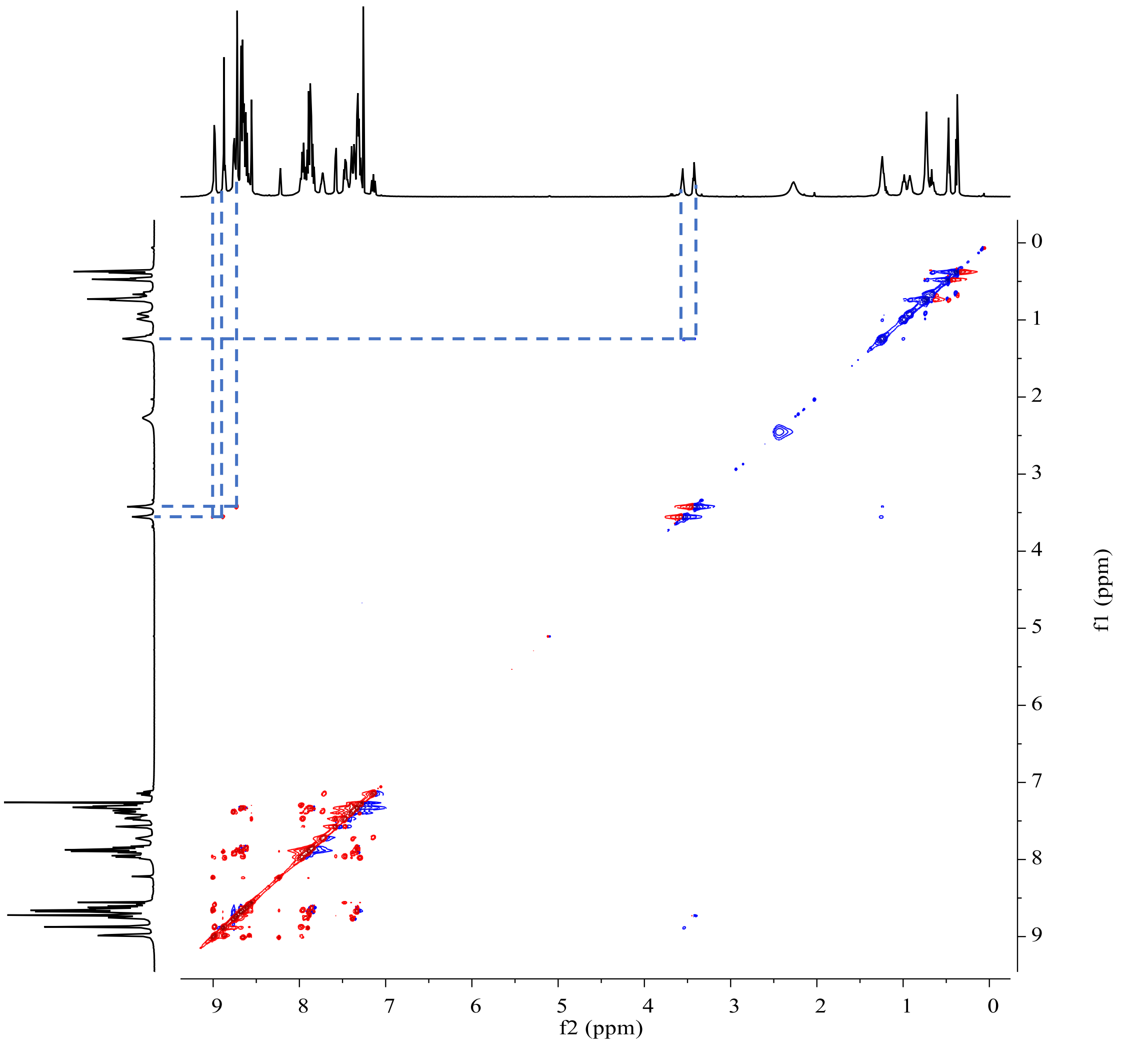


**Supplementary Figure 54.** 2D ROESY NMR NMR spectrum (500 MHz, $CDCl_3$, 300K) for ligand **L4**.

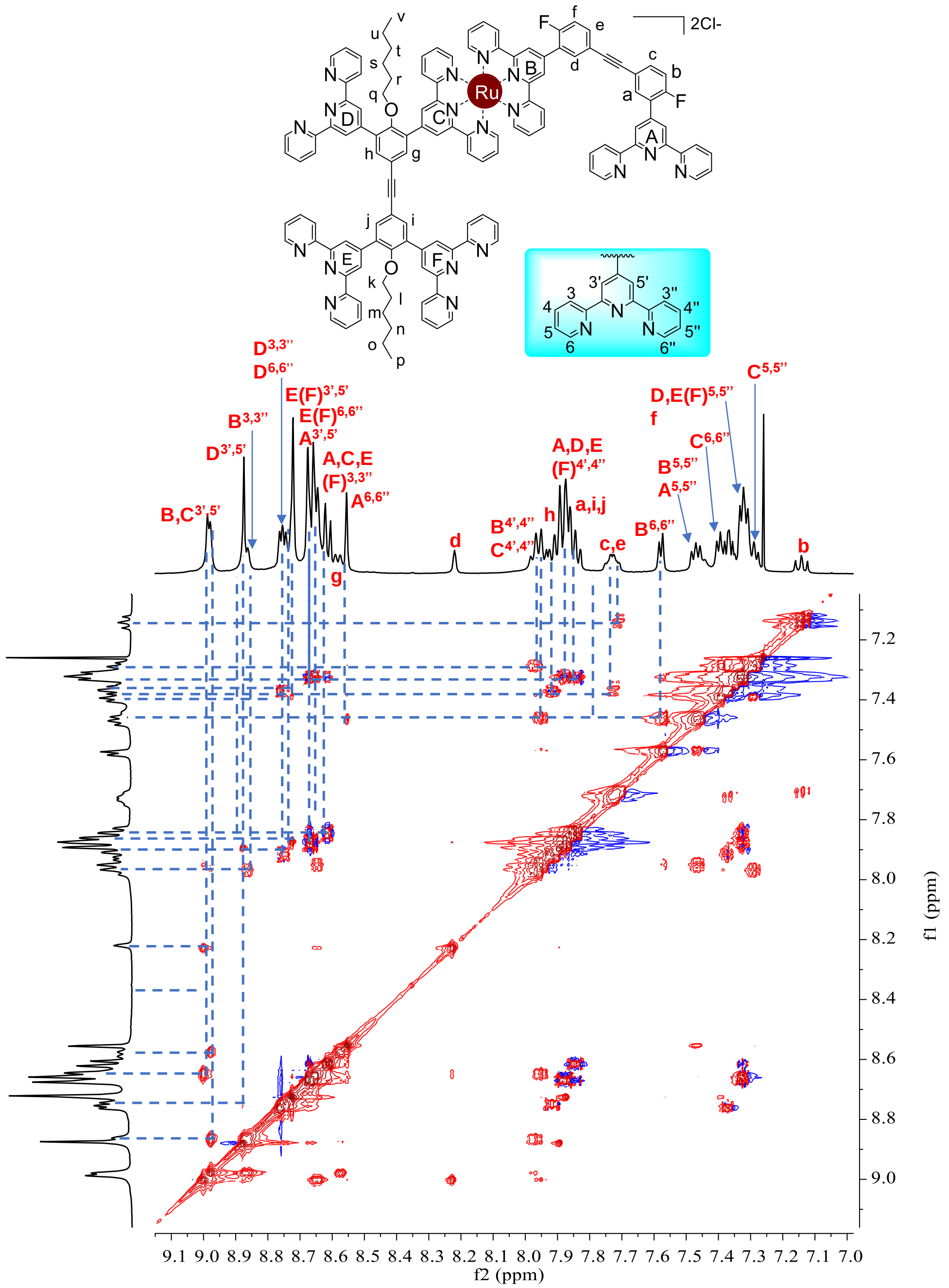

**Supplementary Figure 55.** 2D ROESY NMR NMR spectrum (500 MHz, $CDCl_3$, 300K) for ligand **L4** (aromatic region).

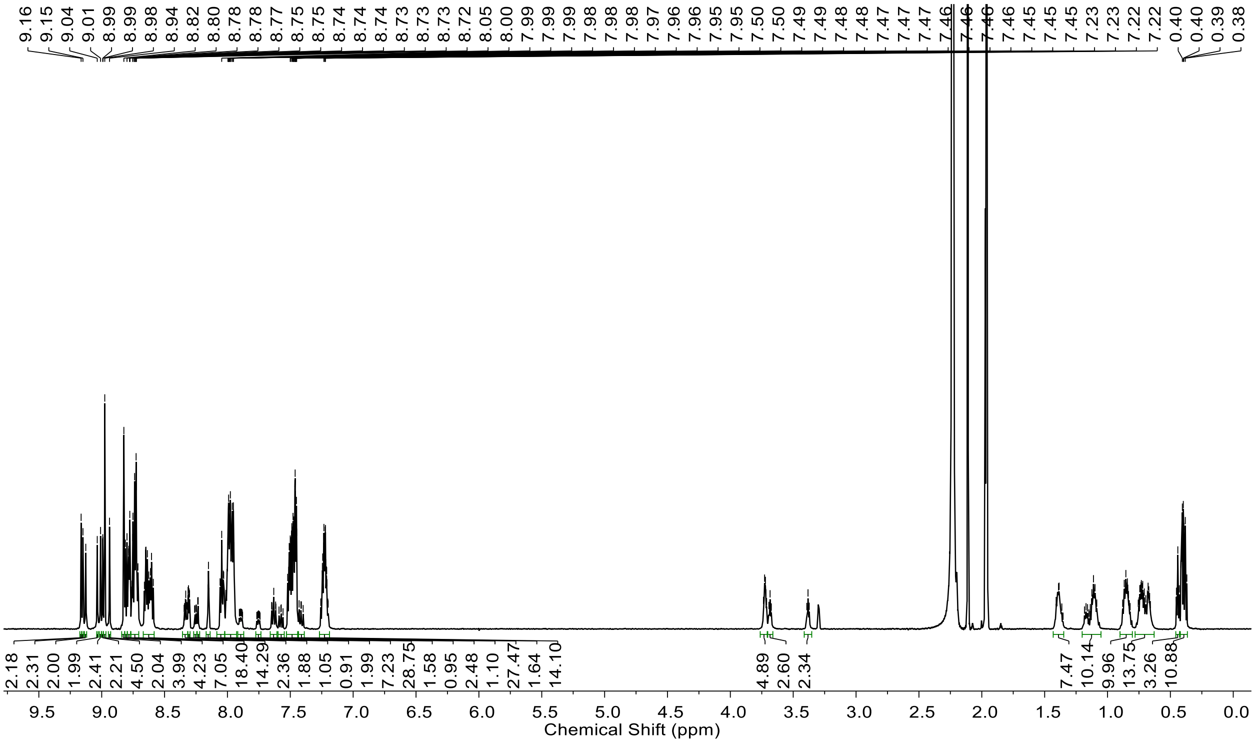


**Supplementary Figure 56.** $^{1}H$ NMR NMR spectrum (600 MHz, $CD_3CN$, 300K) for ligand **L5.**

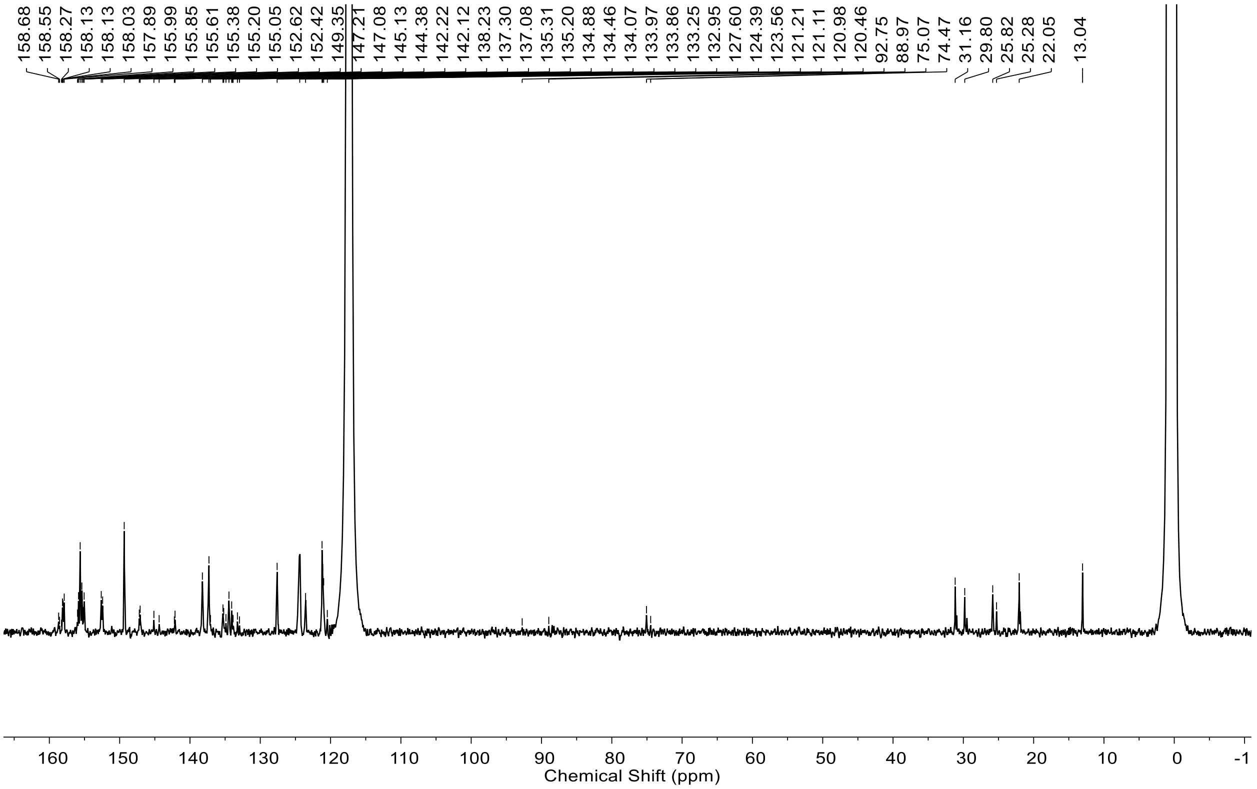


**Supplementary Figure 57.** $^{13}C$ NMR NMR spectrum (100 MHz, $CD_3CN$, 300K) for ligand **L5.**

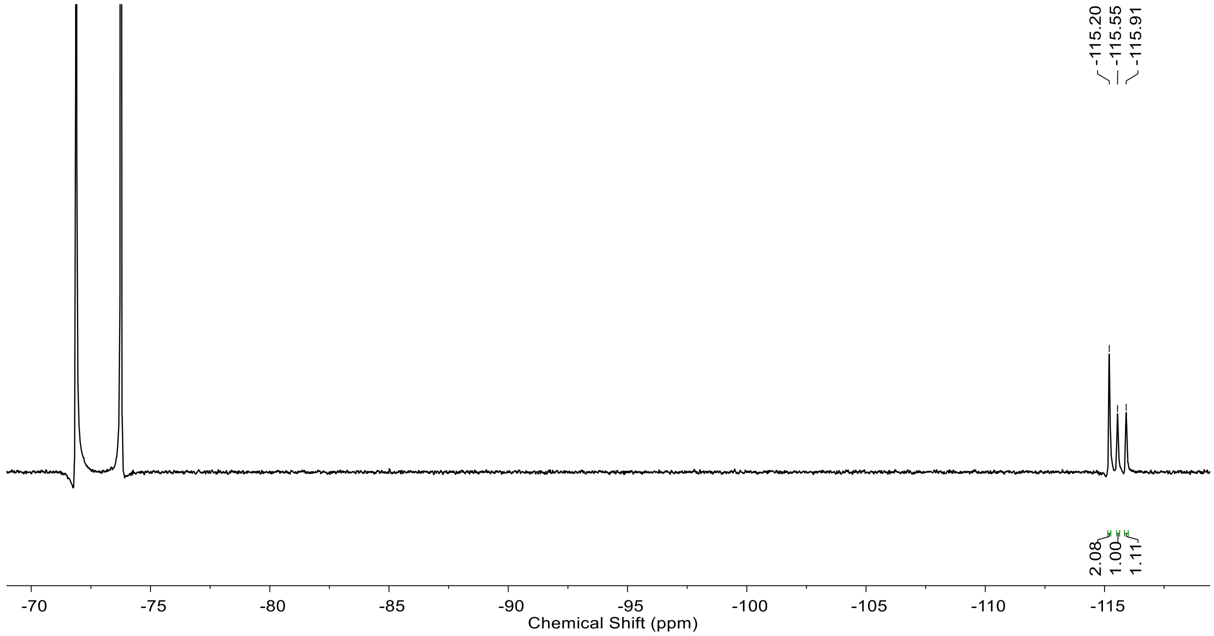


**Supplementary Figure 58.** $^{19}$F NMR NMR spectrum (376 MHz, $CD_3CN$, 300K) for ligand **L5.**

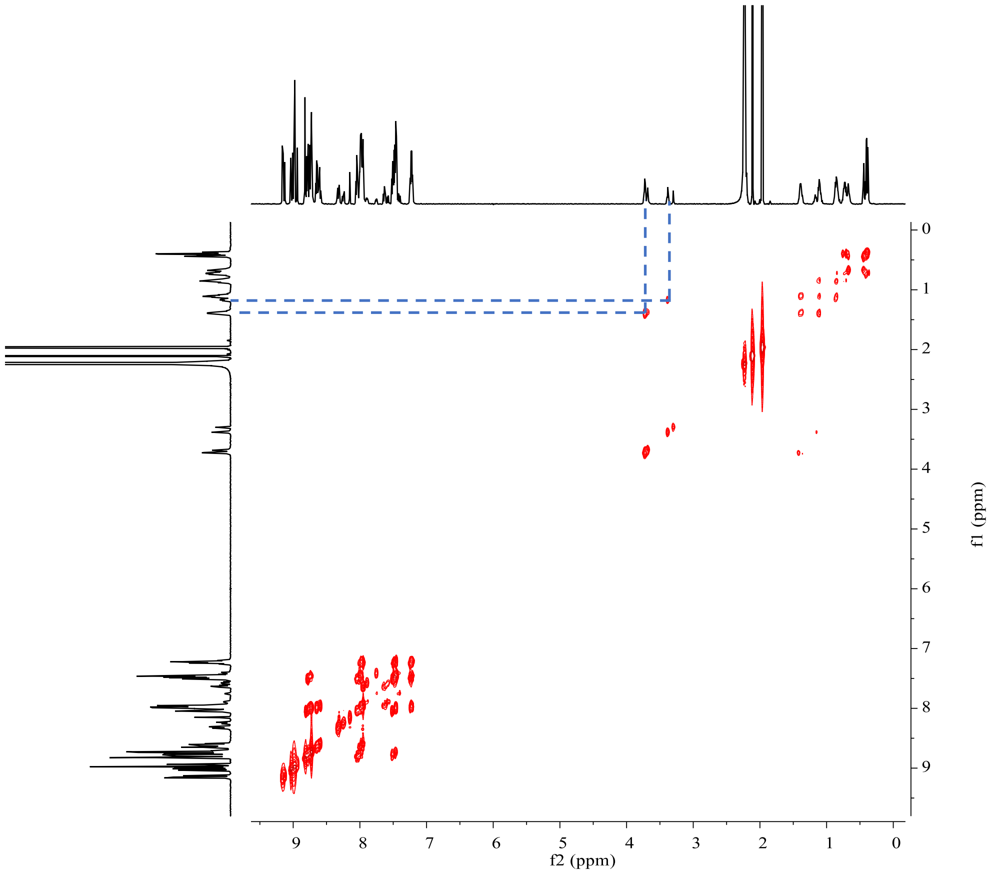


**Supplementary Figure 59.** 2D COSY NMR NMR spectrum (600 MHz, $CD_3CN$, 300K) for ligand **L5.**

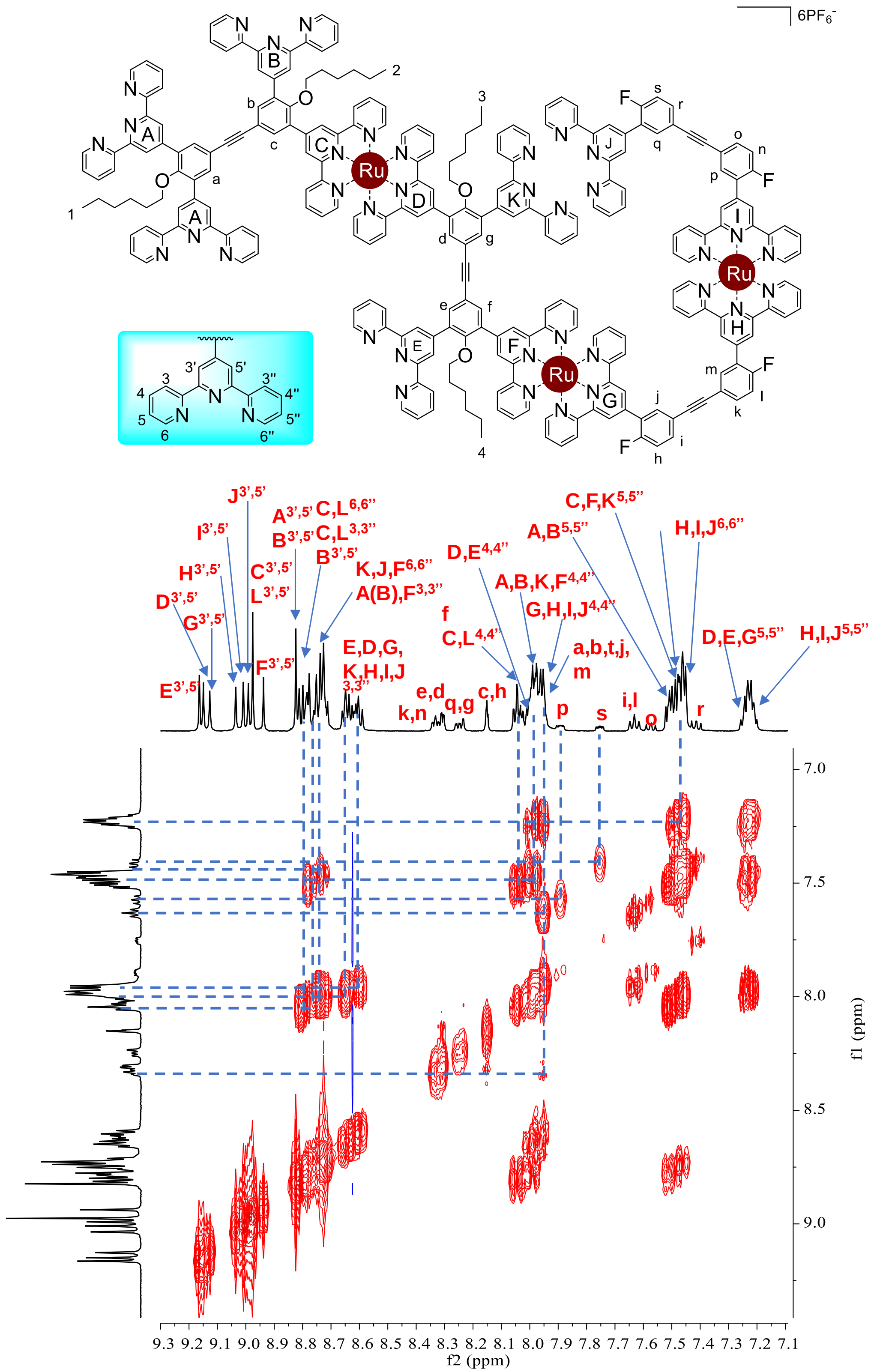

**Supplementary Figure 60.** 2D COSY NMR NMR spectrum (600 MHz, $CD_3CN$, 300K) for ligand **L5** (aromatic region).

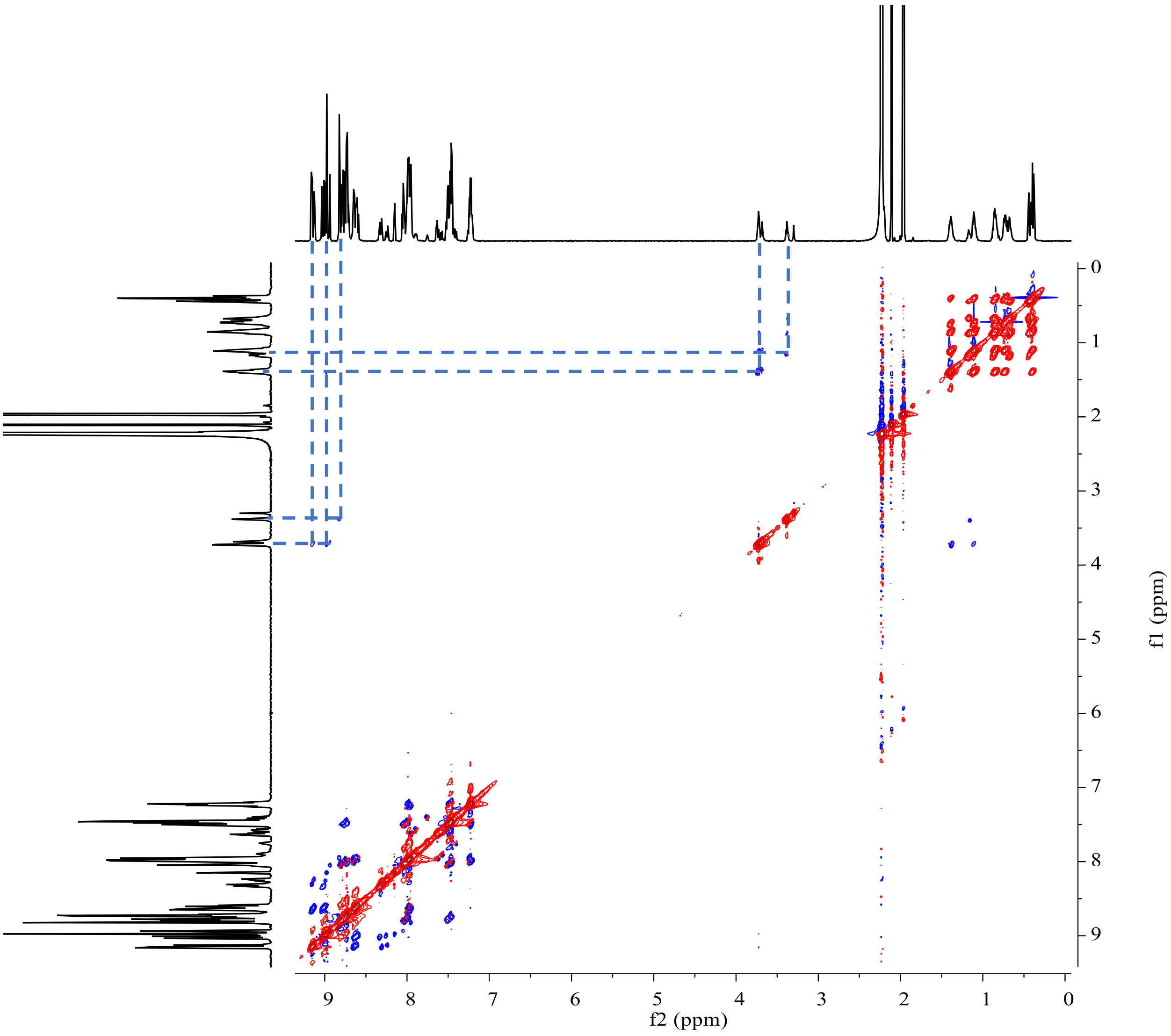


**Supplementary Figure 61.** 2D ROESY NMR spectrum (600 MHz, $CD_3CN$, 300K) for ligand **L5**.

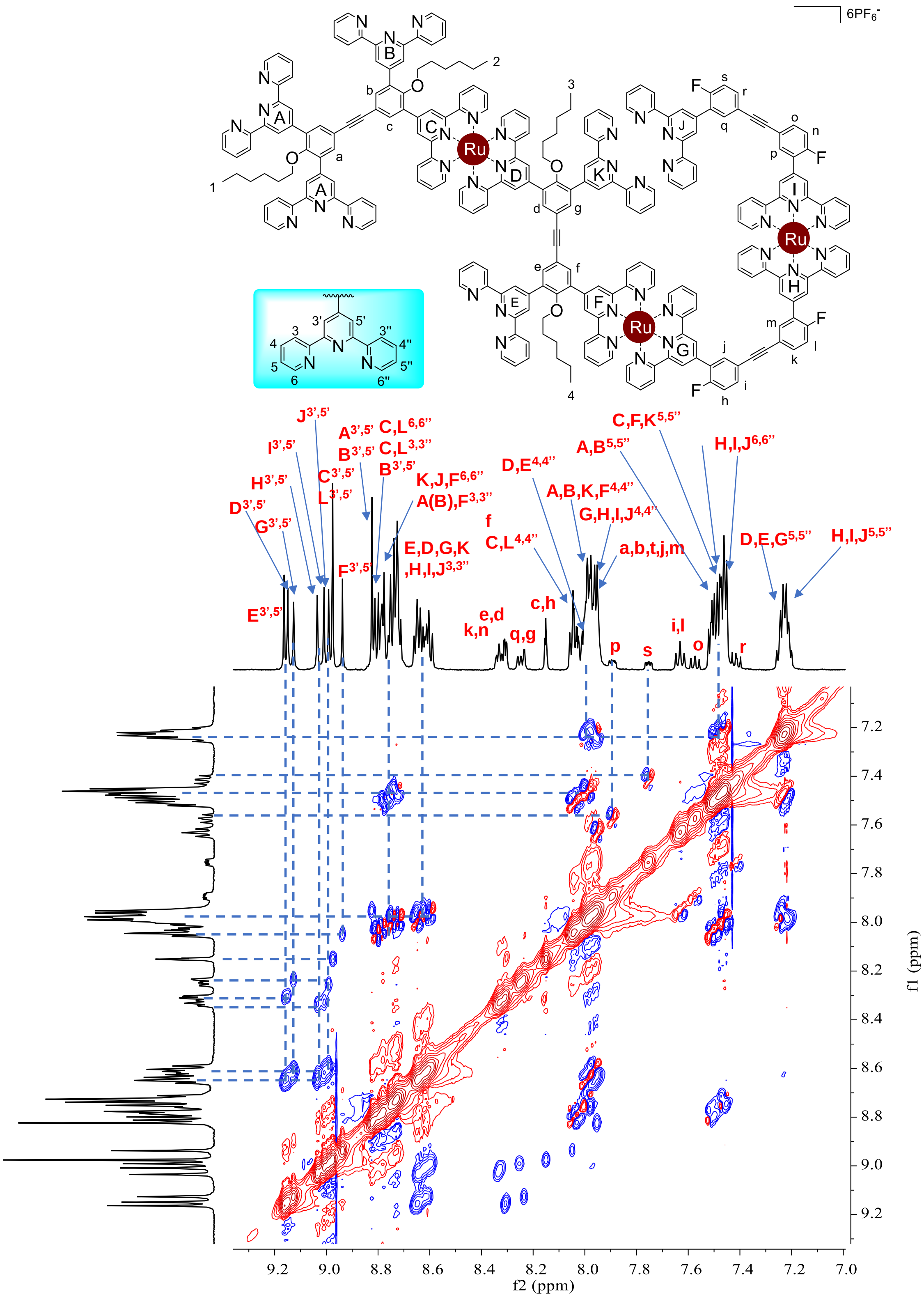


**Supplementary Figure 62.** 2D ROESY NMR spectrum (600 MHz, $CD_3CN$, 300K) for ligand **L5** (aromatic region).

# NMR spectra for supramolecules C1-C5

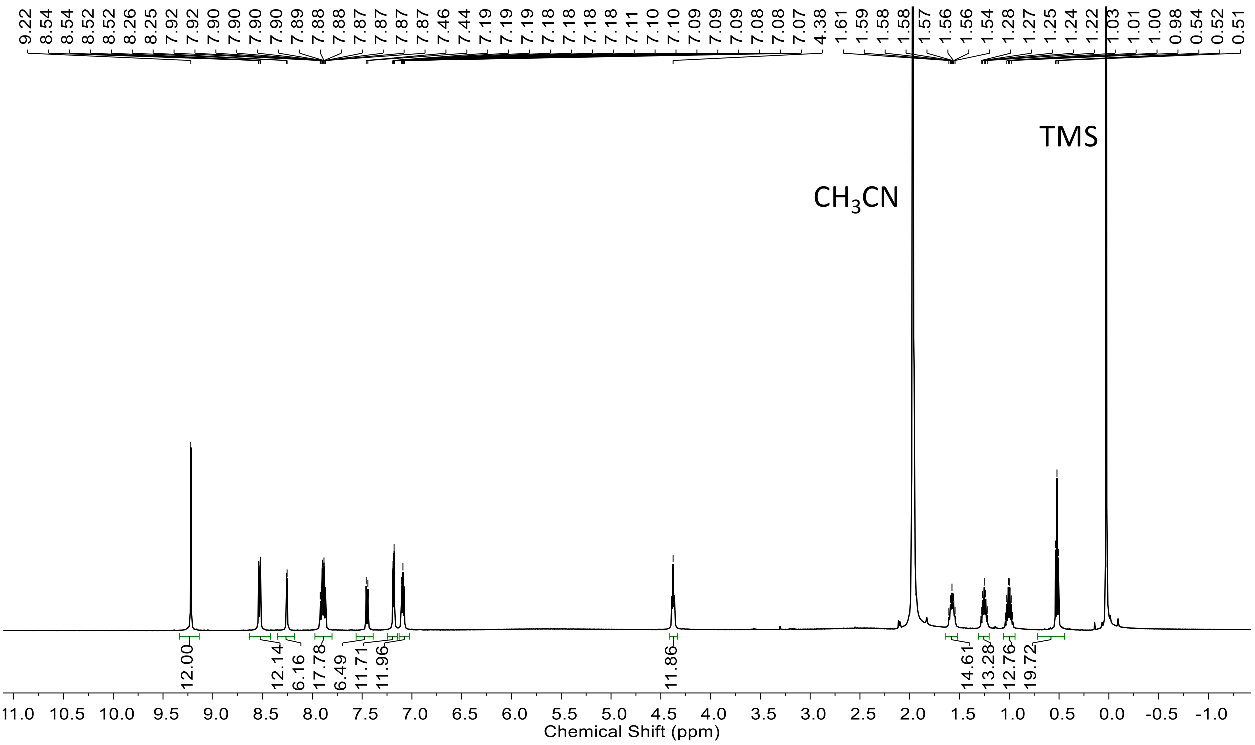


**Supplementary Figure 63.** $^{1}$H NMR spectrum (600 MHz, $CD_3CN$, 300K) for supramolecule **C1**

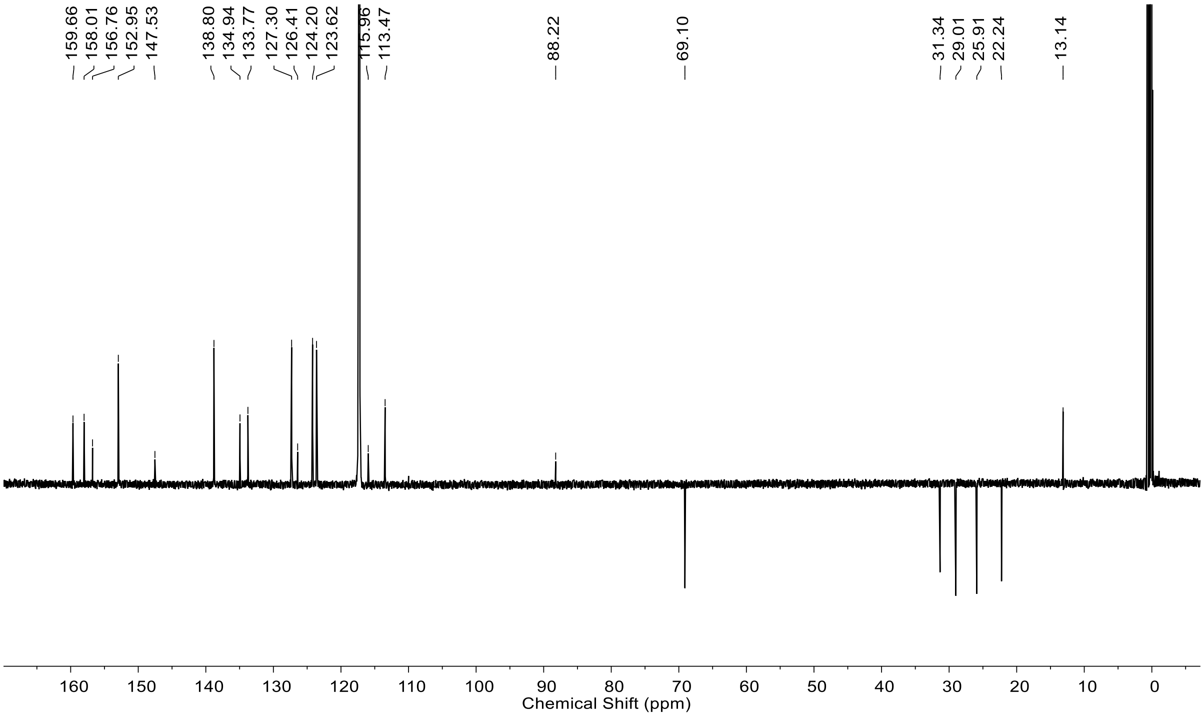


**Supplementary Figure 64.** DEPT Q NMR spectrum (600 MHz, $CD_3CN$, 300K) for supramolecule **C1**.

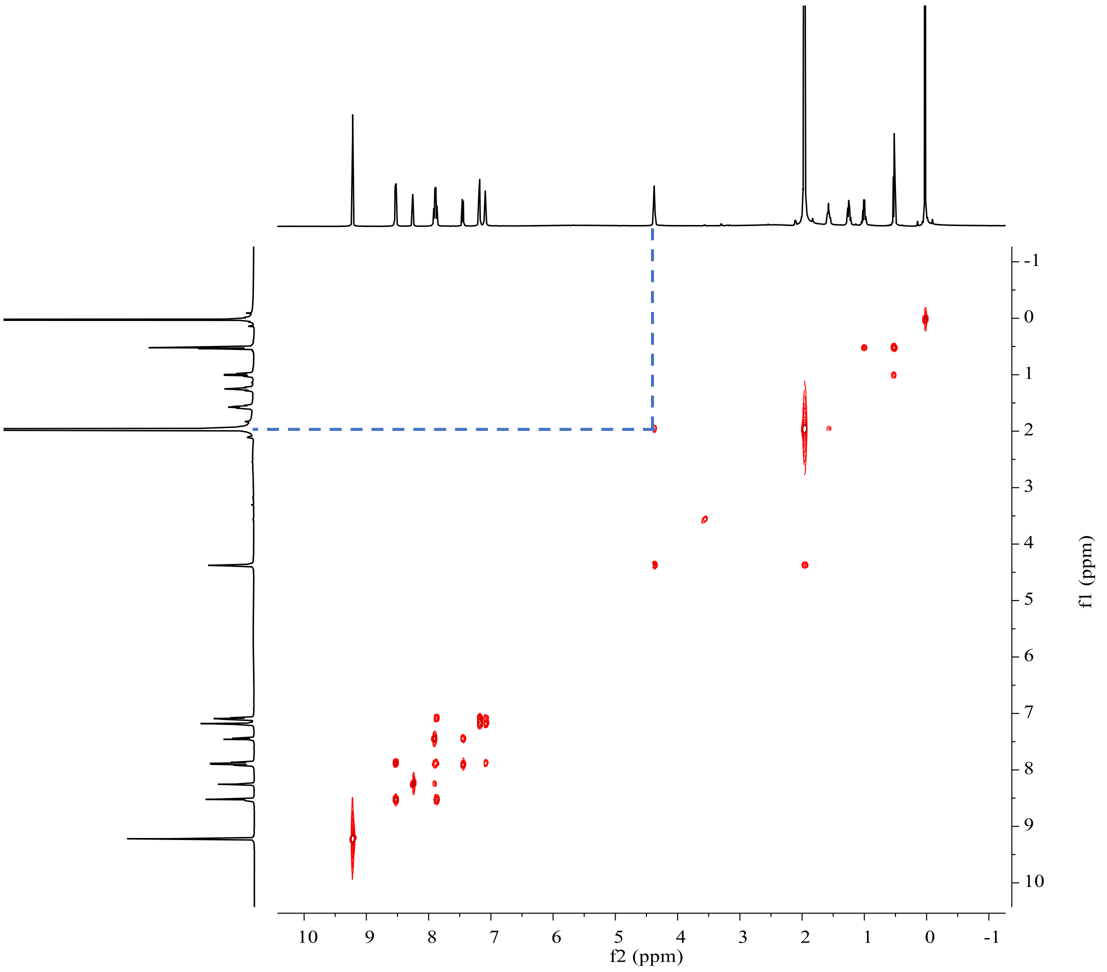


**Supplementary Figure 65.** 2D COSY NMR spectrum (600 MHz, $CD_3CN$, 300K) for supramolecule **C1**.

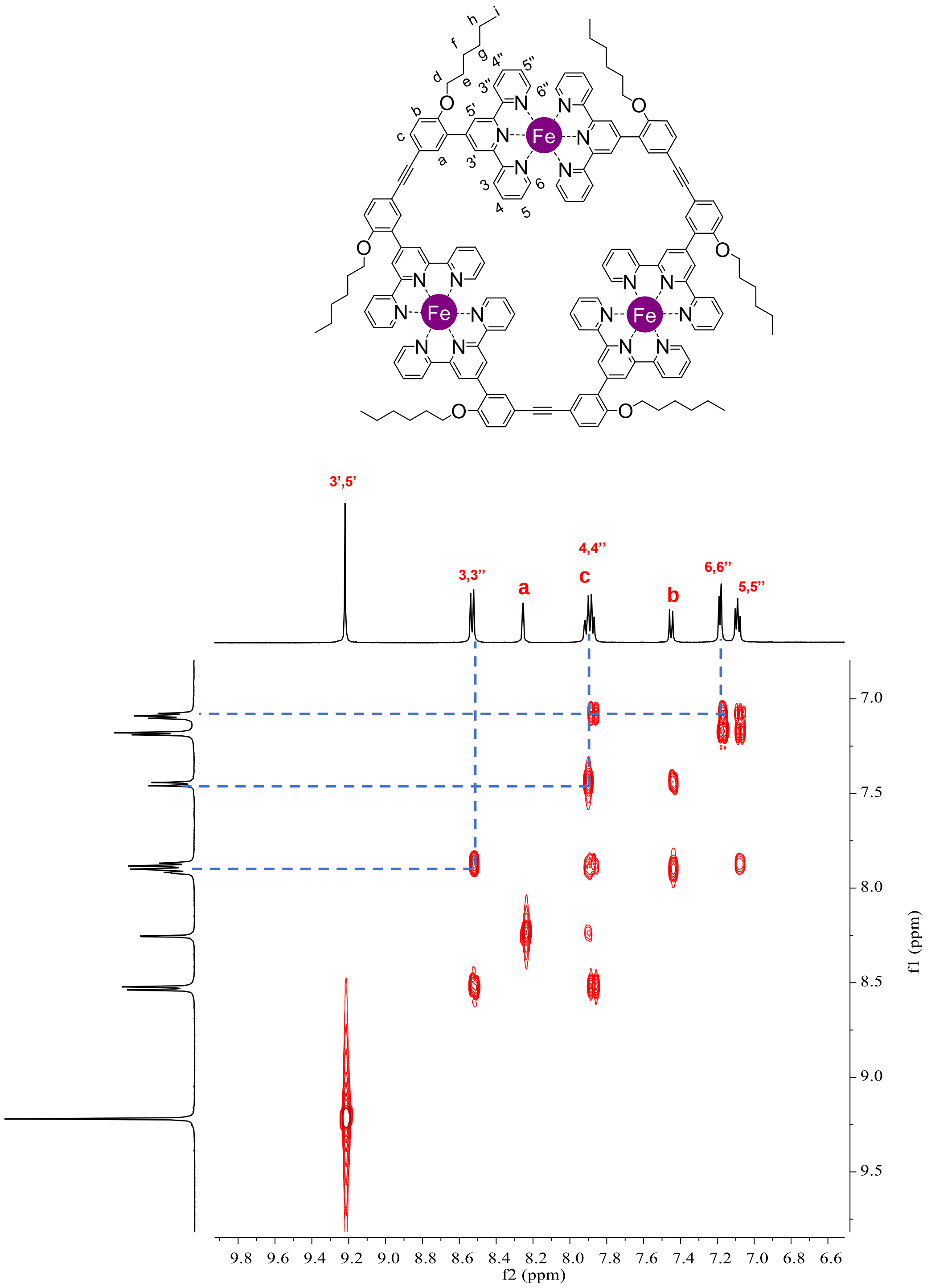


**Supplementary Figure 66.** 2D COSY NMR spectrum (600 MHz, $CD_3CN$, 300K) for supramolecule **C1**. (aromatic region)

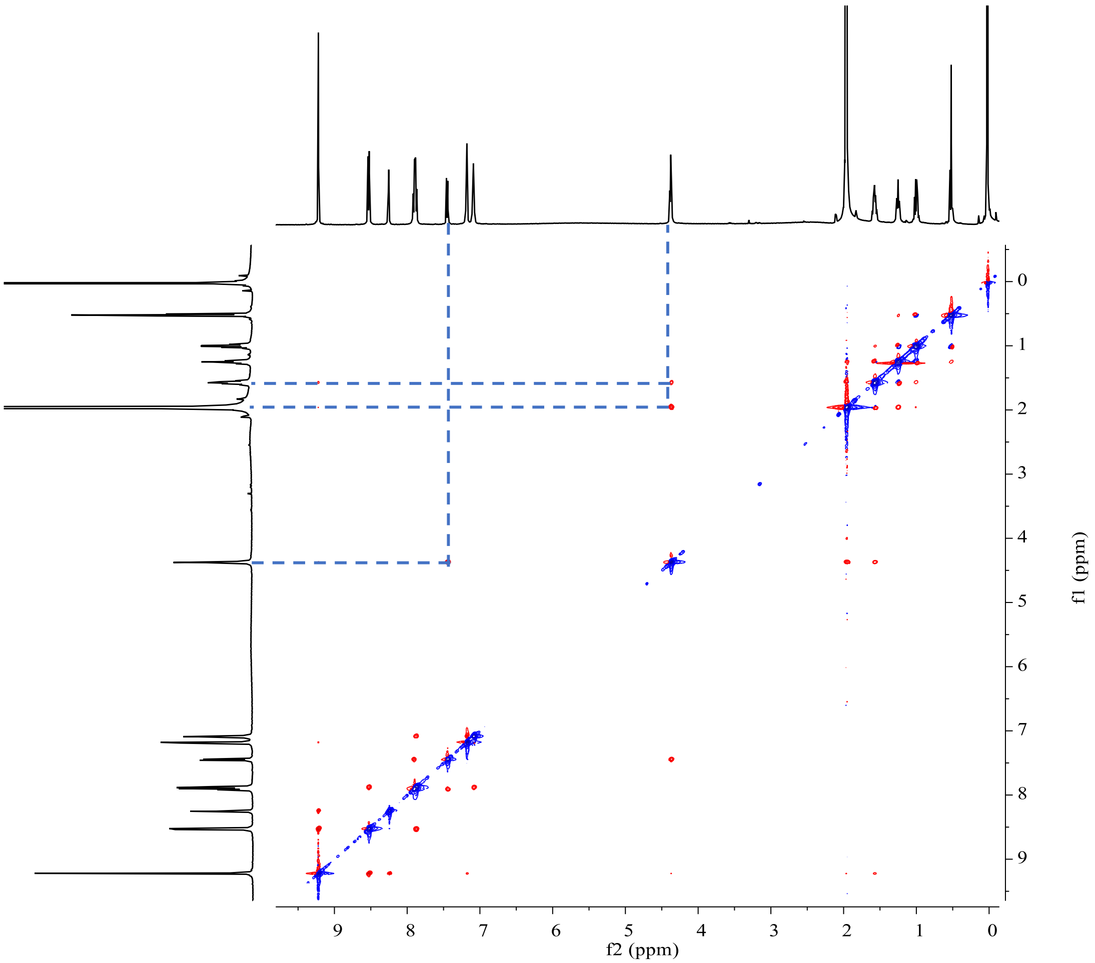


**Supplementary Figure 67.** 2D ROESY NMR spectrum (600 MHz, $CD_3CN$, 300K) for supramolecule **C1**.

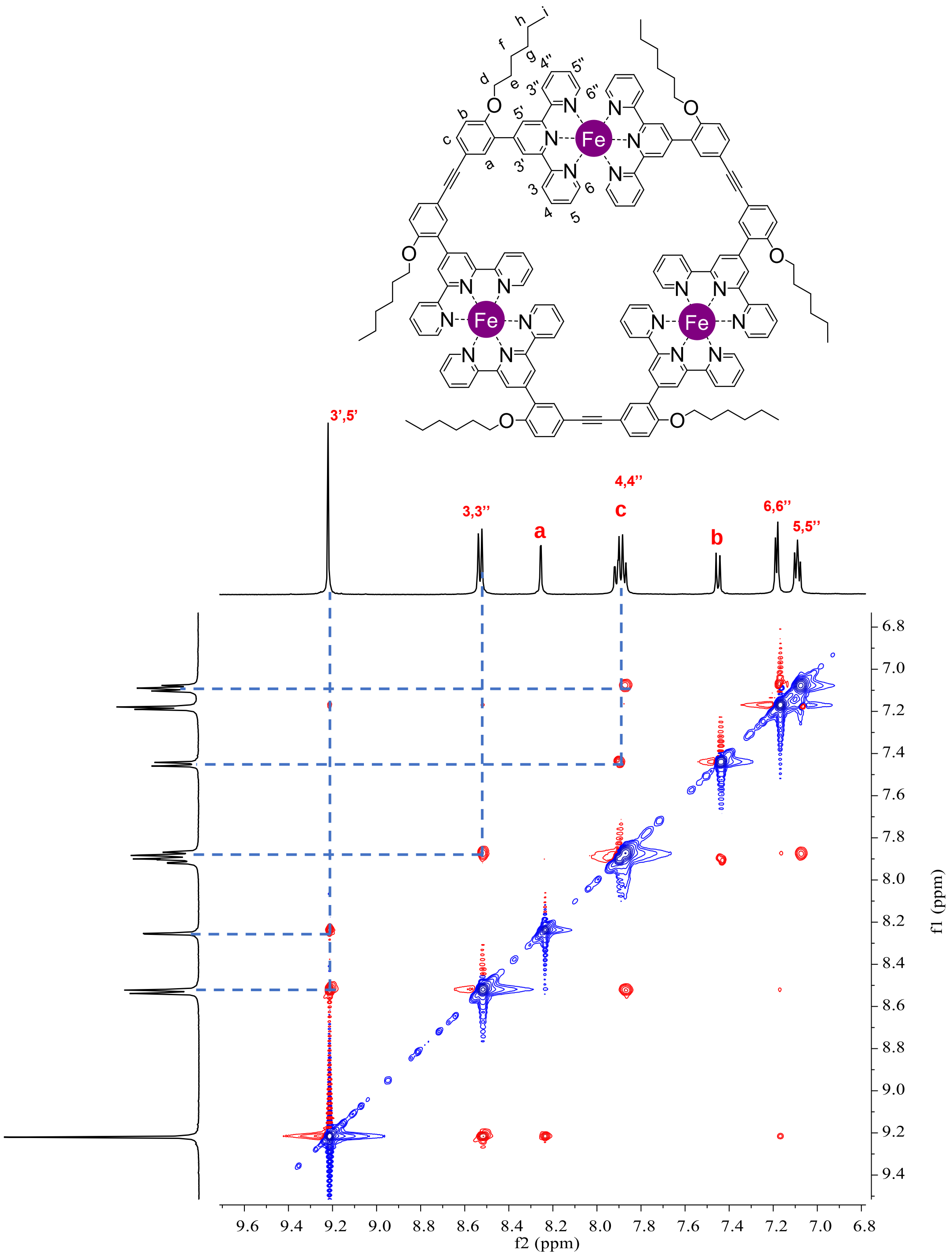


**Supplementary Figure 68.** 2D ROESY NMR spectrum (600 MHz, $CD_3CN$, 300K) for supramolecule **C1**. (aromatic region)

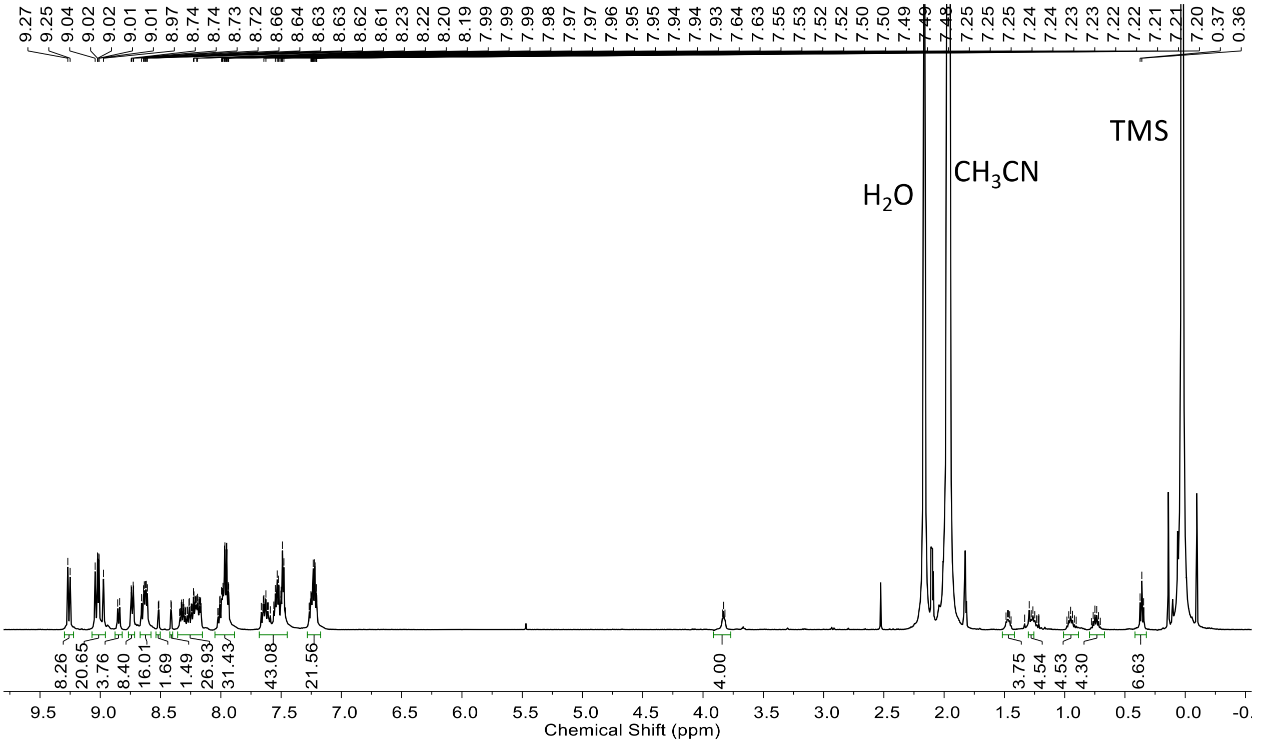


**Supplementary Figure 69.** $^{1}H$ NMR spectrum (500 MHz, $CD_3CN$, 300K) for supramolecule **C2**.

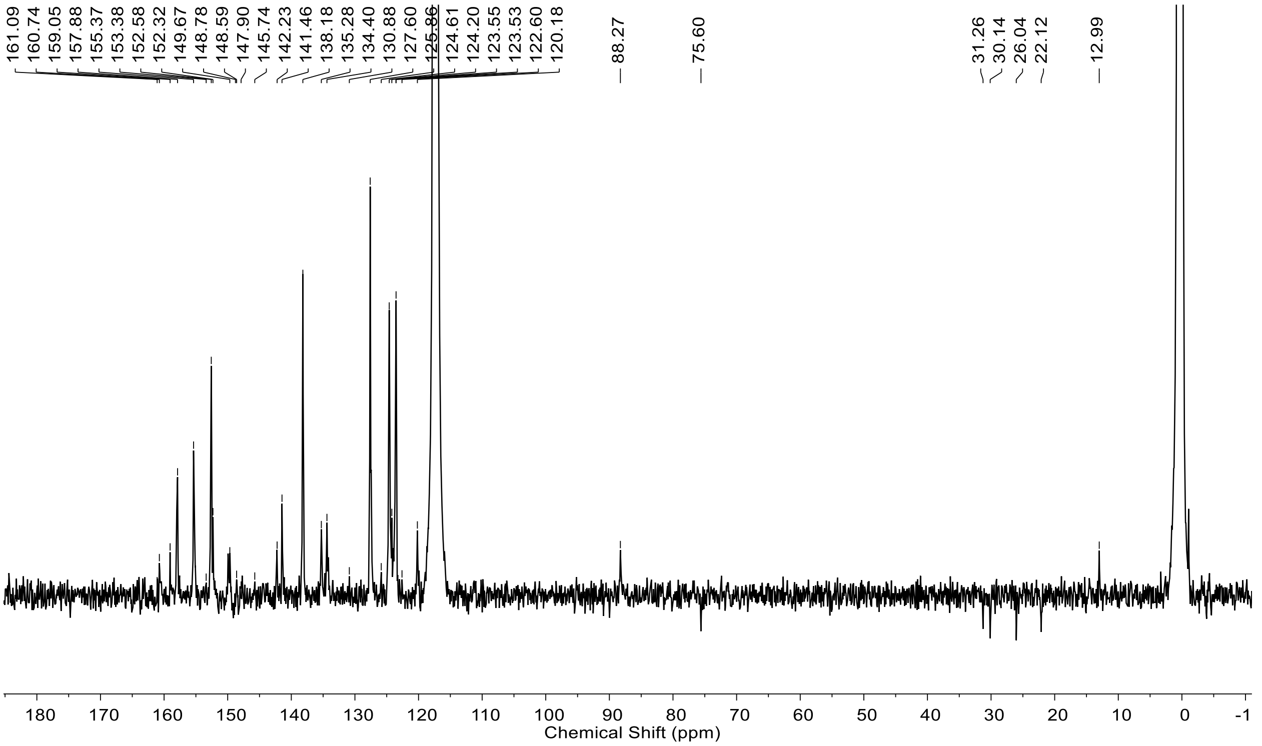


**Supplementary Figure 70.** DEPT Q NMR spectrum (600 MHz, $CD_3CN$, 300K) for supramolecule **C2.**

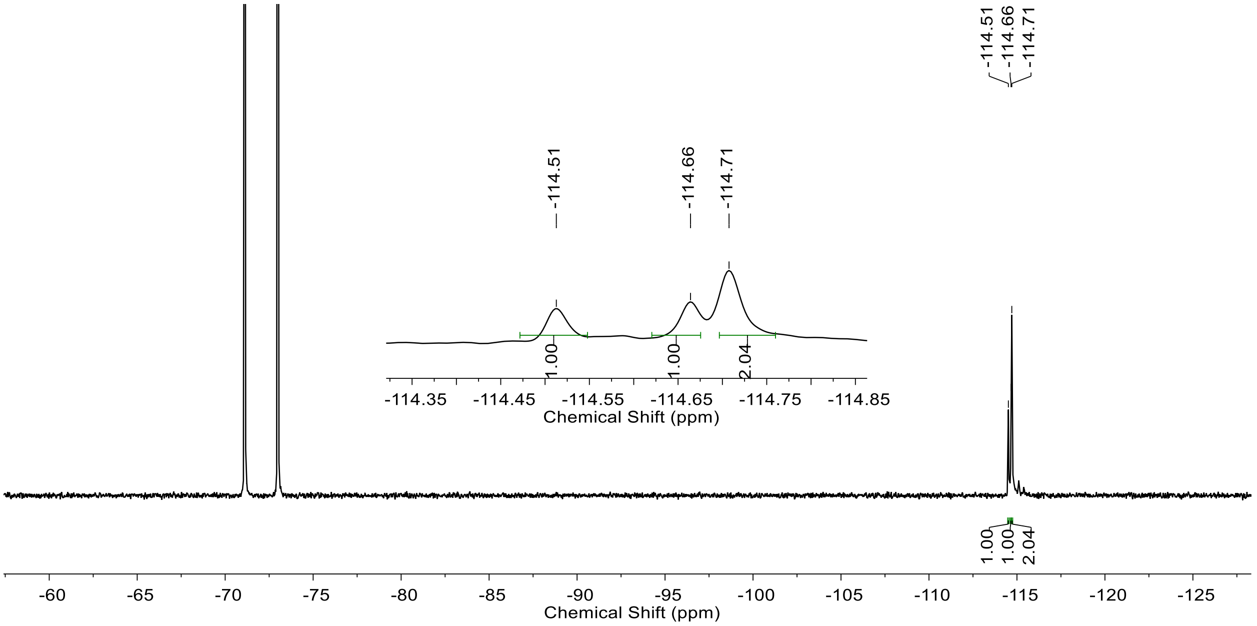


**Supplementary Figure 71.** $^{19}F$ NMR spectrum (376 MHz, $CD_3CN$, 300K) for supramolecule **C2.**

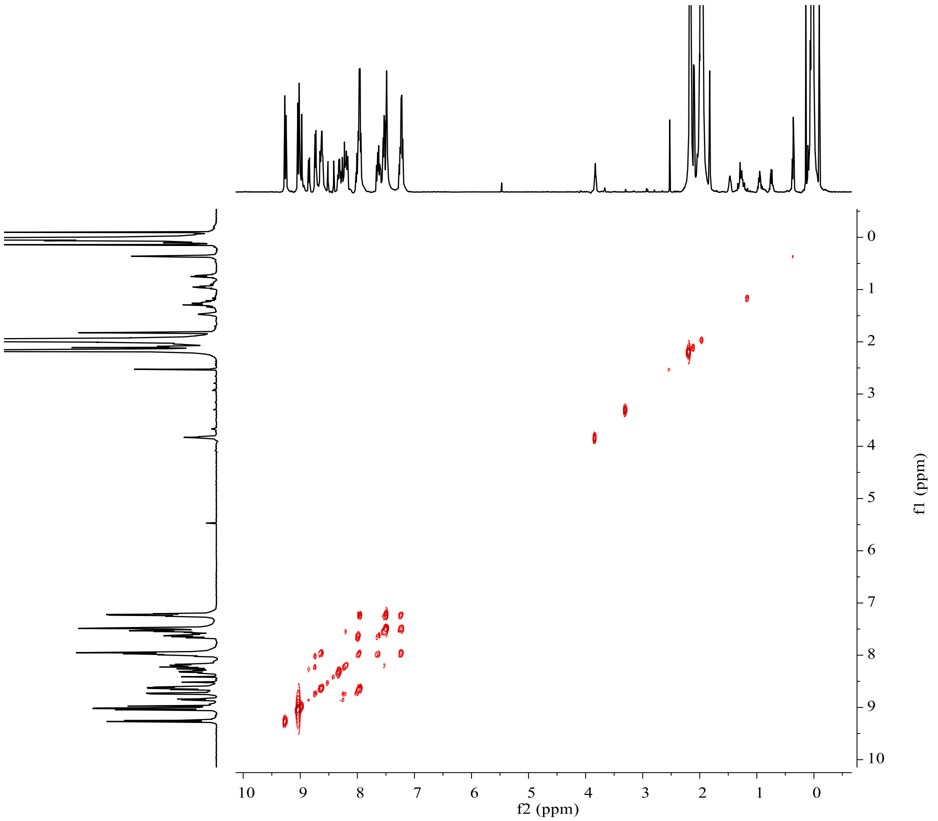


**Supplementary Figure 72.** 2D COSY NMR spectrum (600 MHz, $CD_3CN$, 300K) for supramolecule **C2.**

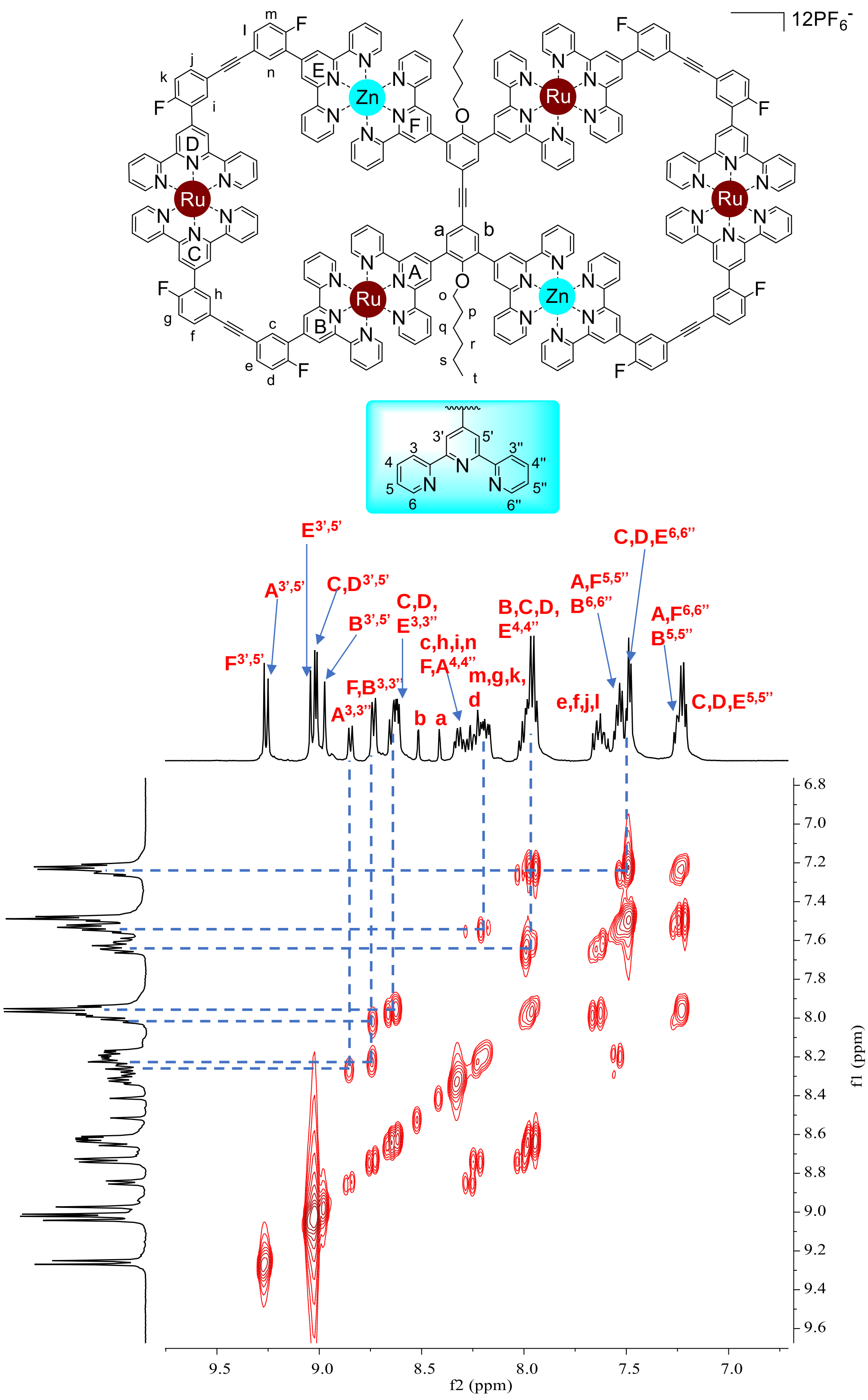


**Supplementary Figure 73.** 2D COSY NMR spectrum (600 MHz, $CD_3CN$, 300K) for supramolecule **C2** (aromatic region).

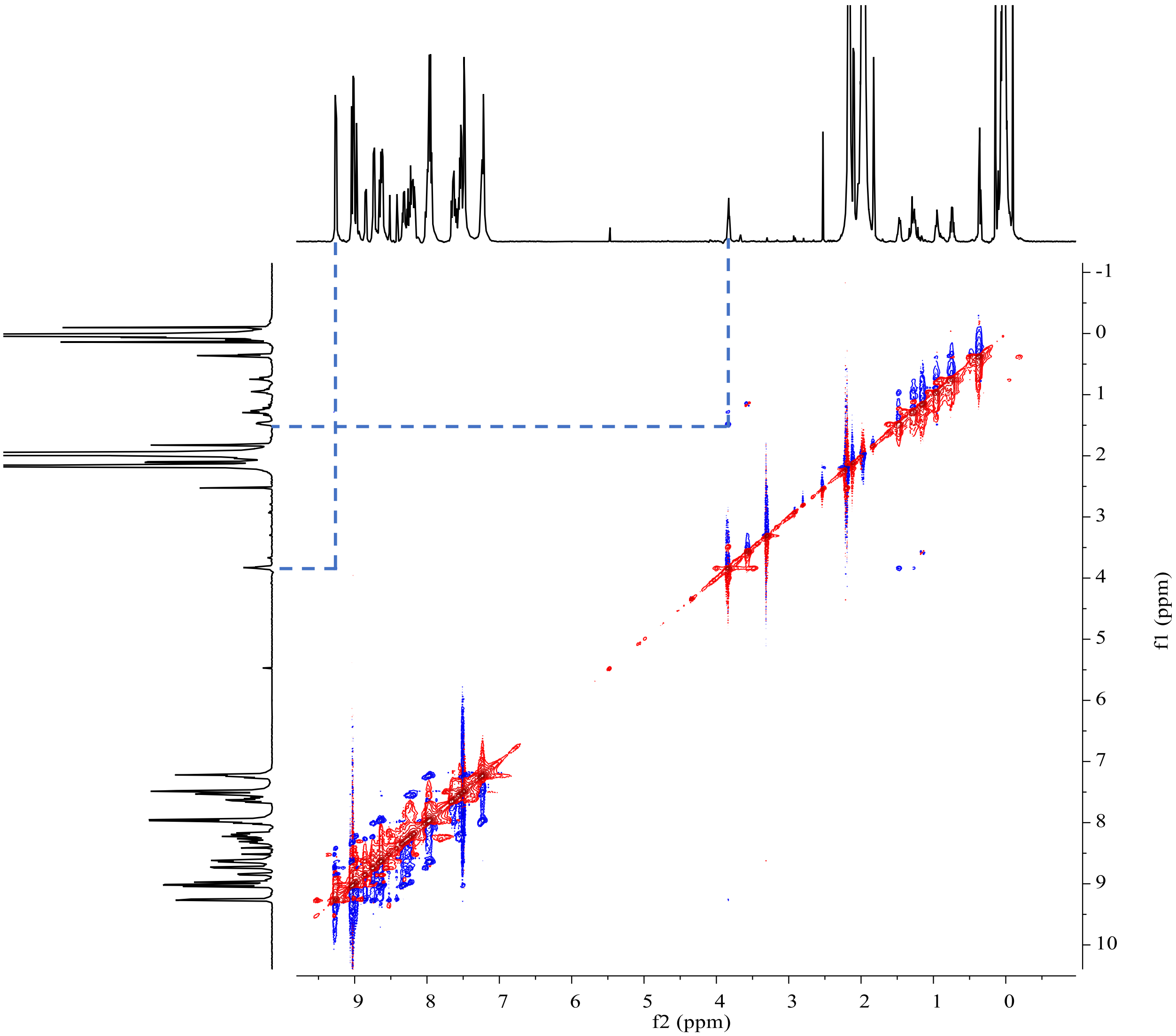


**Supplementary Figure 74.** 2D ROESY NMR spectrum (600 MHz, $CD_3CN$, 300K) for supramolecule **C2.**

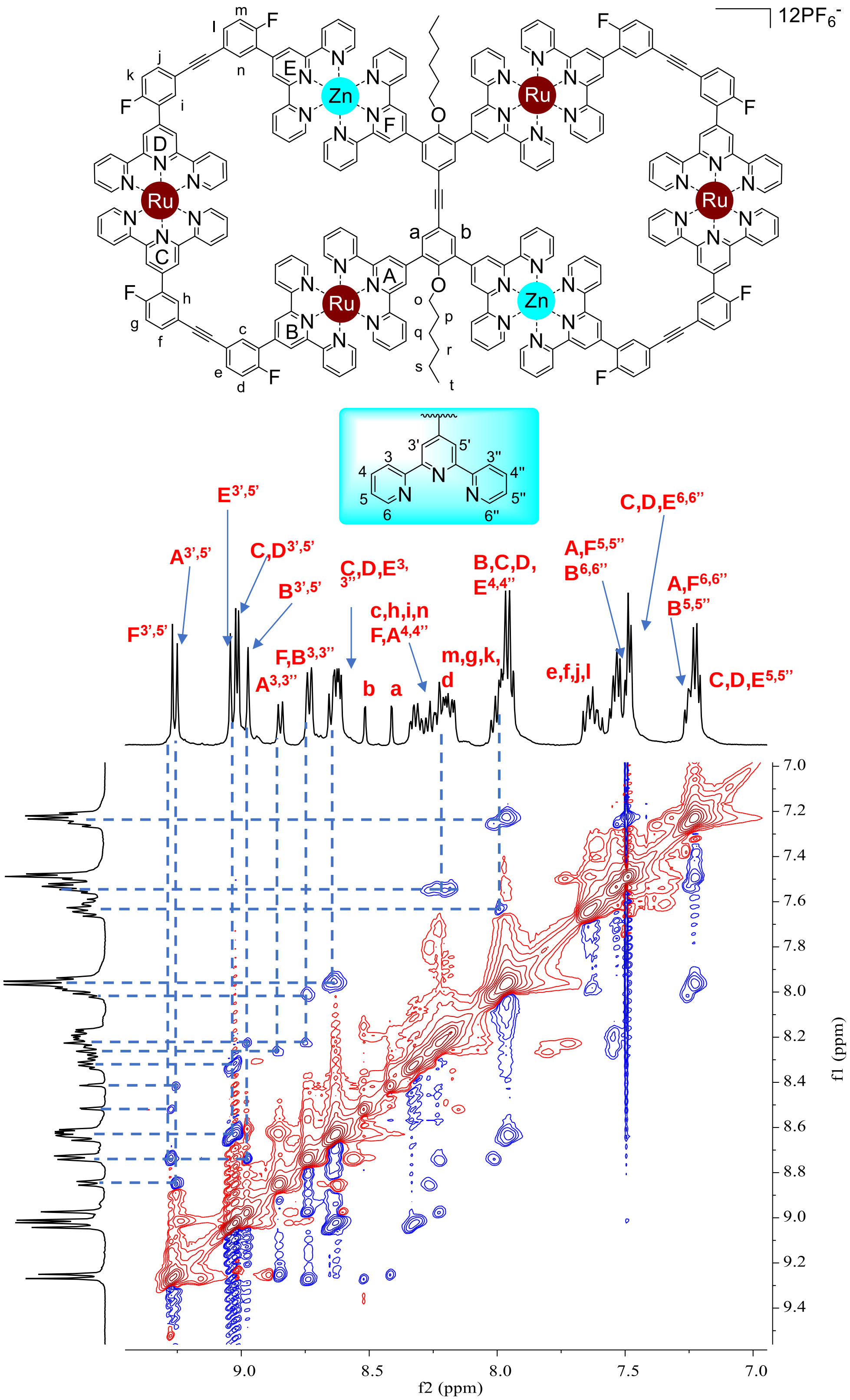


**Supplementary Figure 75.** 2D ROESY NMR spectrum (600 MHz, $CD_3CN$, 300K) for supramolecule **C2** (aromatic region).

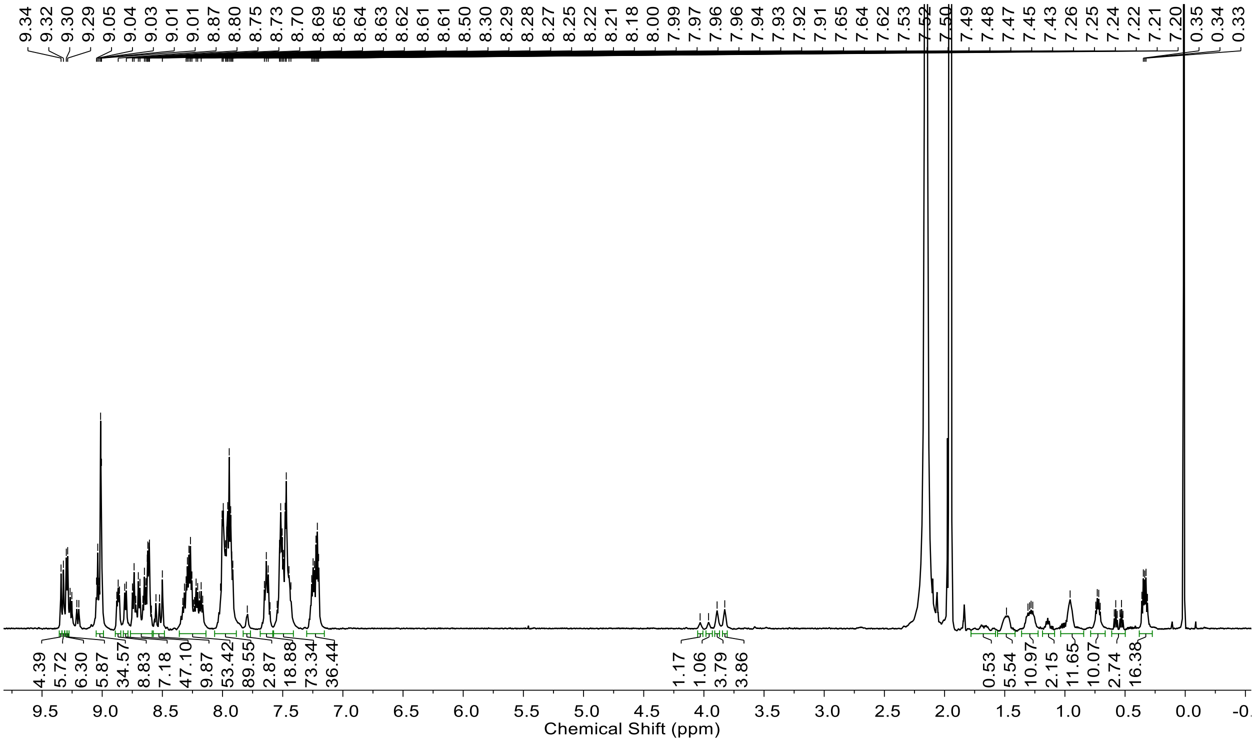


**Supplementary Figure 76.** $^{1}H$ NMR spectrum (600 MHz, $CD_3CN$, 300K) for supramolecule **C3**.

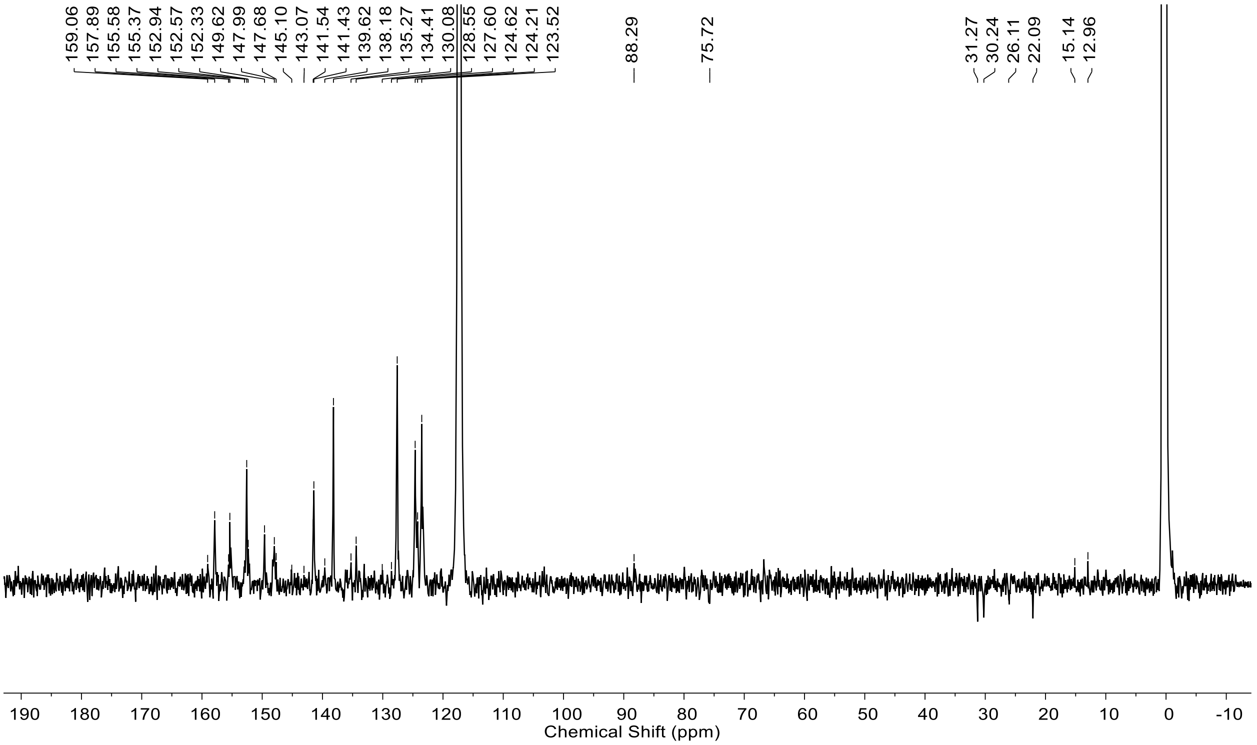


**Supplementary Figure 77.** DEPT Q NMR spectrum (125 MHz, $CD_3CN$, 300K) for supramolecule **C3**.

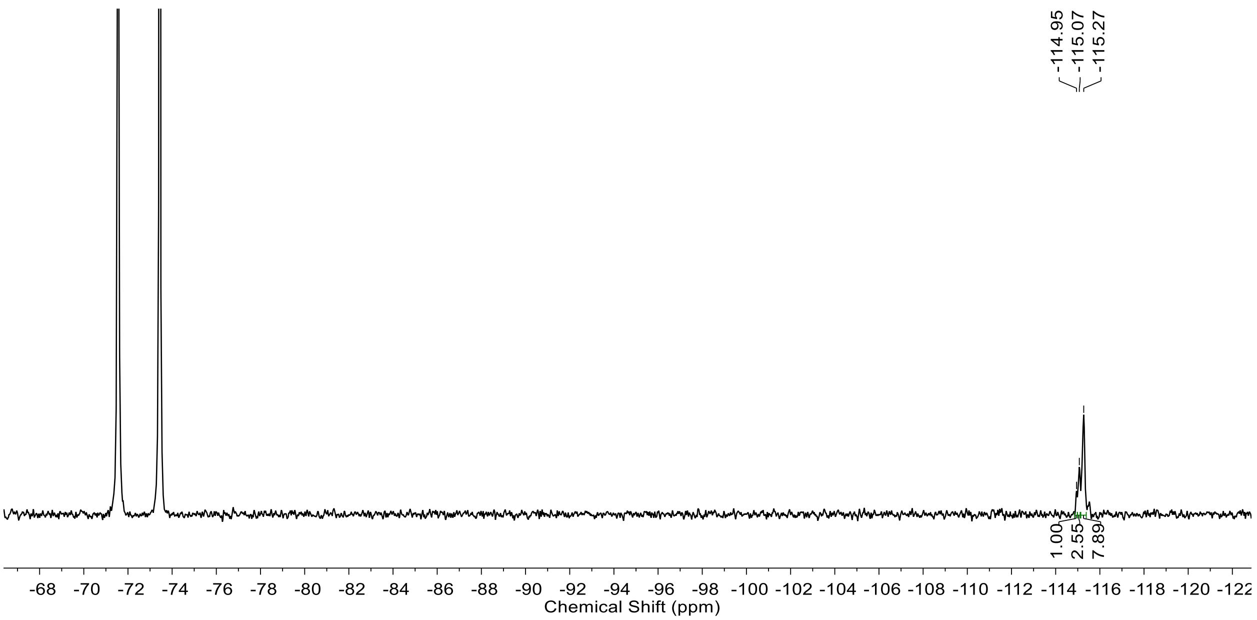


**Supplementary Figure 78.** $^{19}F$ NMR spectrum (376 MHz, $CD_3CN$, 300K) for supramolecule **C3**.

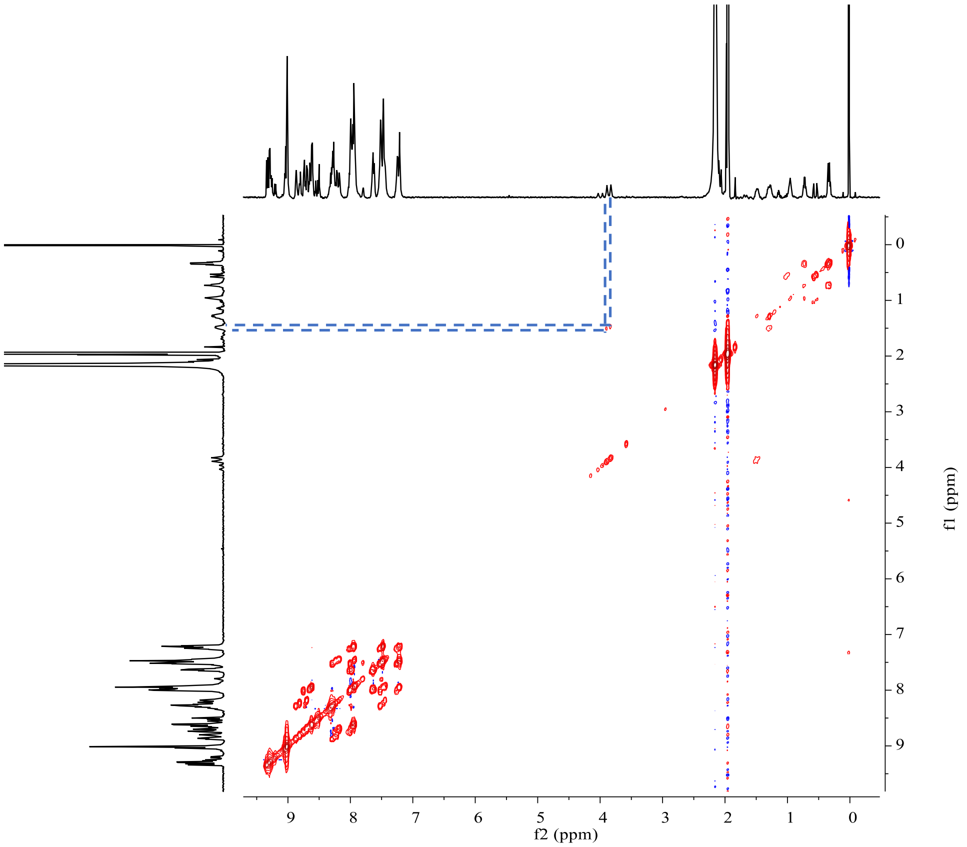


**Supplementary Figure 79.** 2D COSY NMR spectrum (600 MHz, $CD_3CN$, 300K) for supramolecule **C3**.

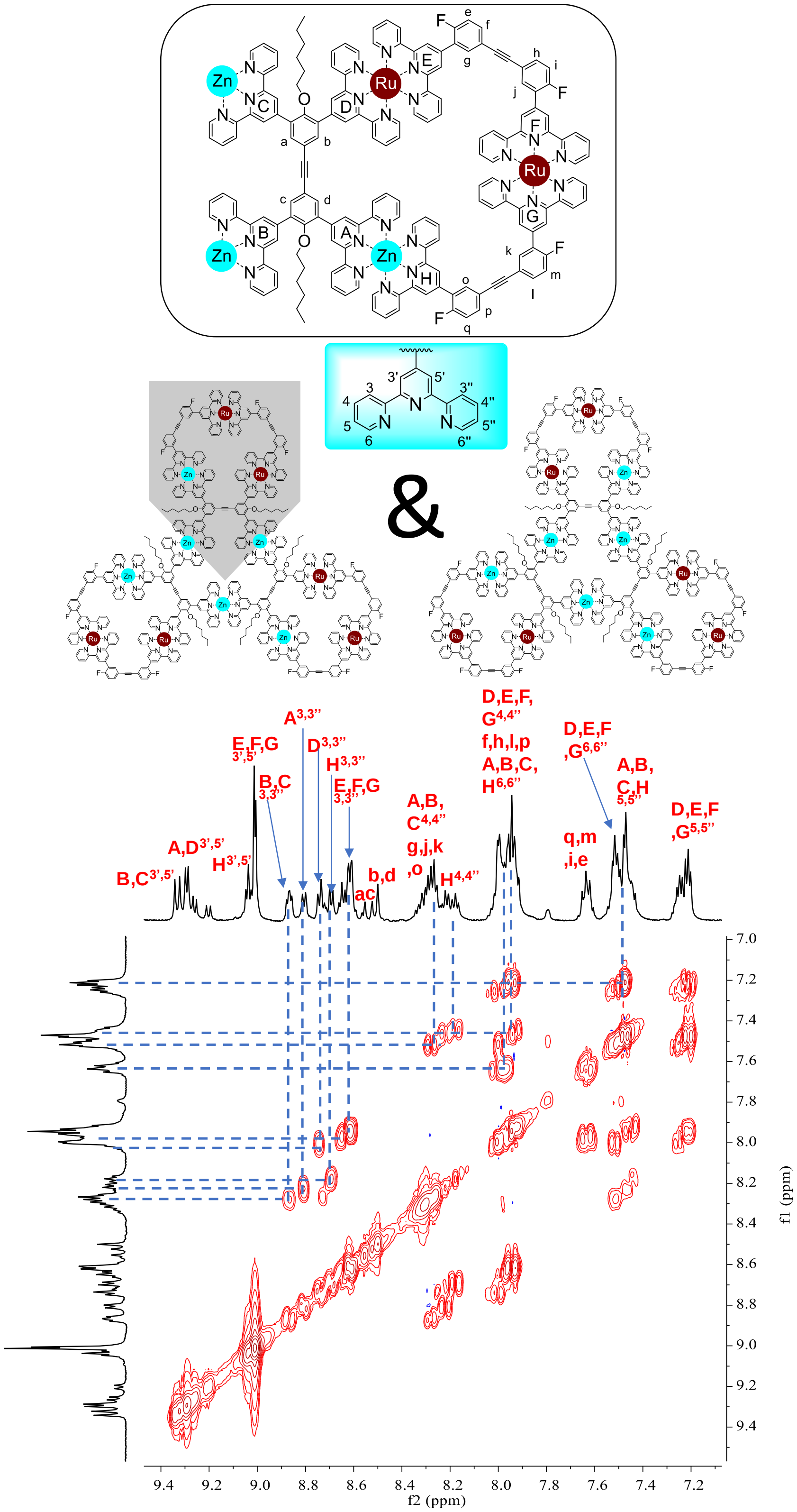


**Supplementary Figure 80.** 2D COSY NMR spectrum (600 MHz, $CD_3CN$, 300K) for supramolecule **C3** (aromatic region). Due to existing of isomers, only major peaks were signed.

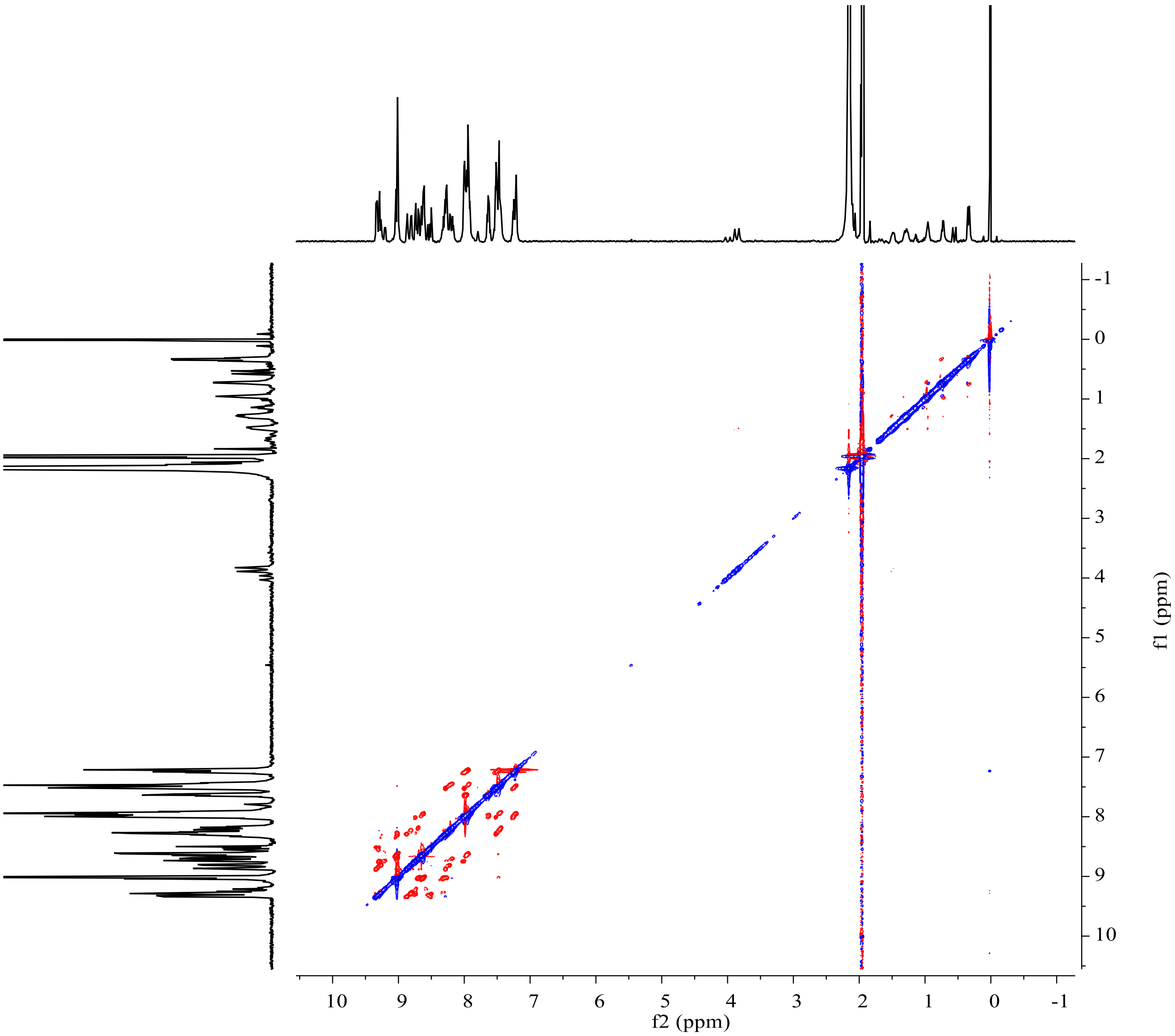


**Supplementary Figure 81.** 2D ROESY NMR spectrum (600 MHz, $CD_3CN$, 300K) for supramolecule **C3**.

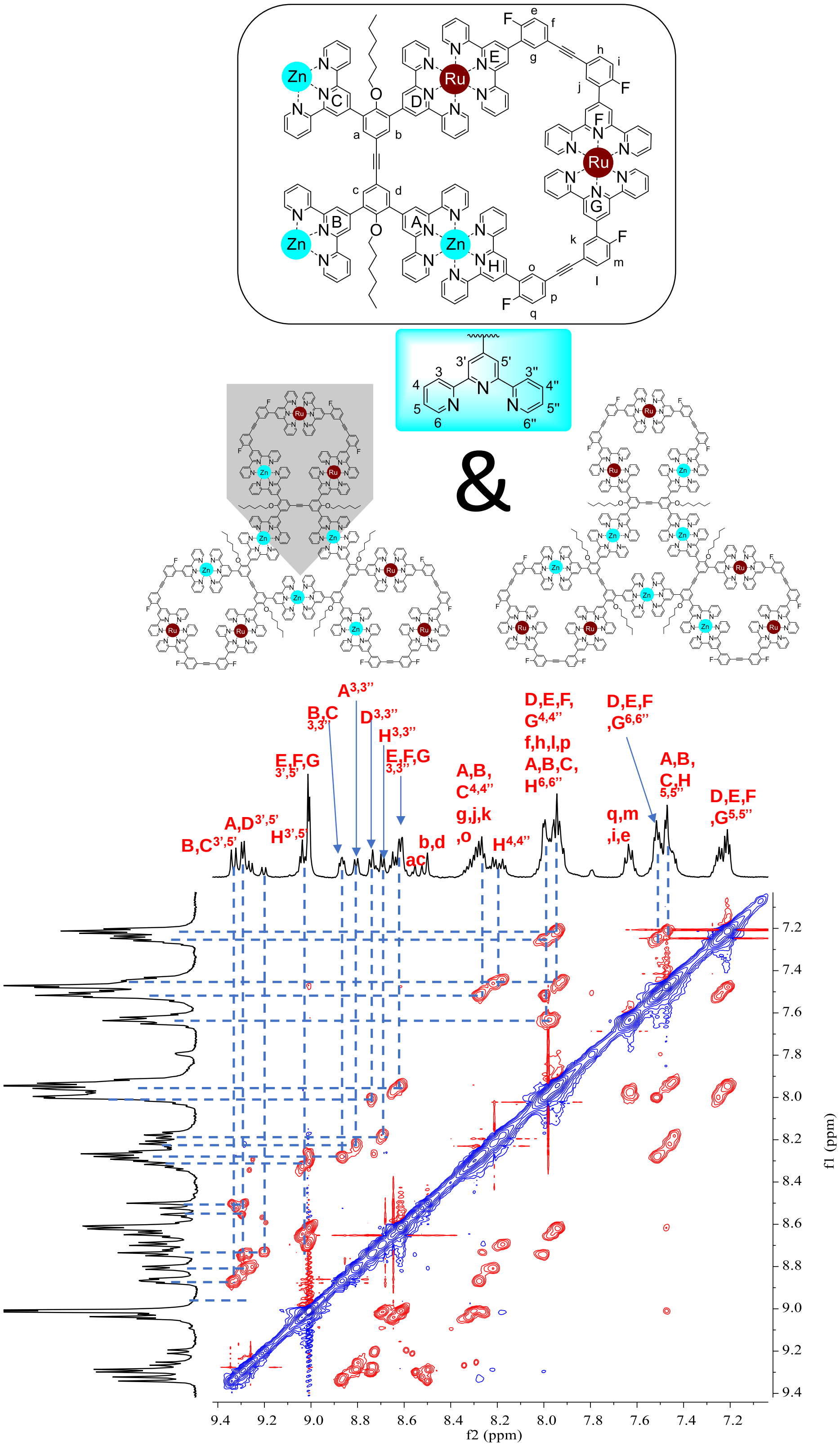

**Supplementary Figure 82.** 2D ROESY NMR spectrum (600 MHz, $CD_3CN$, 300K) for supramolecule **C3** (aromatic region). Due to existing of isomers, only major peaks were signed.

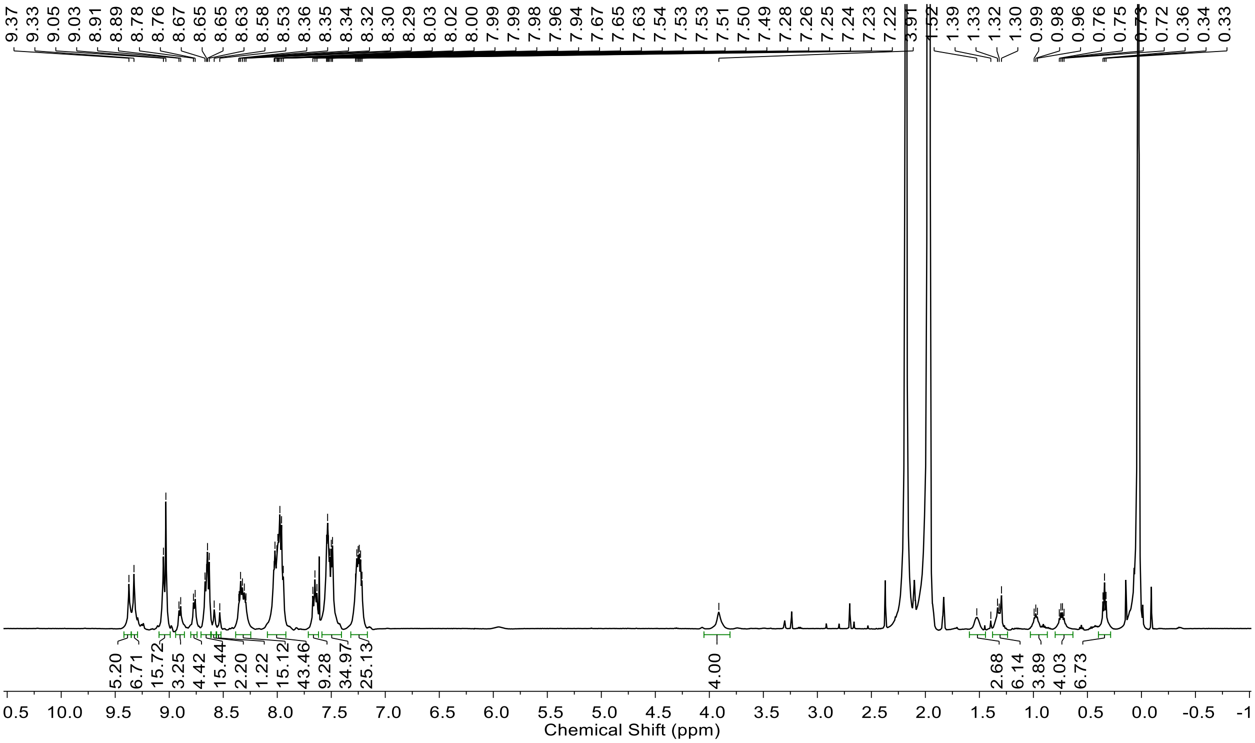


**Supplementary Figure 83.** $^{1}$H NMR spectrum (500 MHz, $CD_3CN$, 300K) for supramolecule **C3'**.

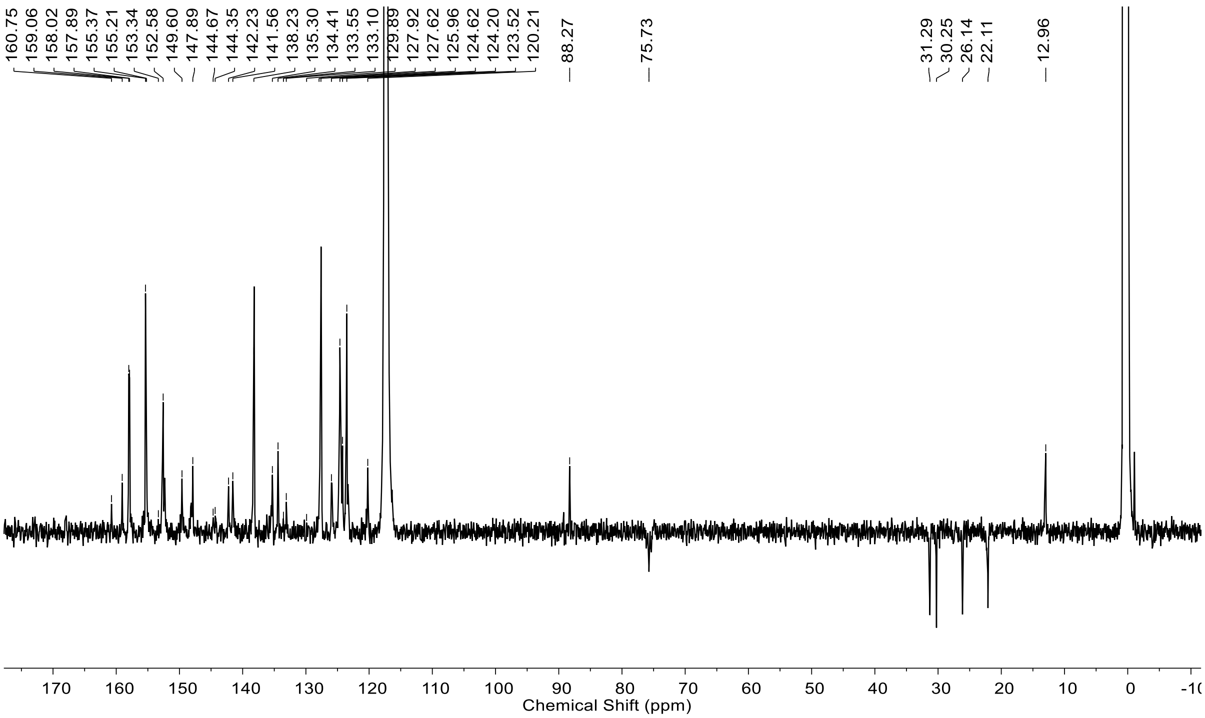


**Supplementary Figure 84.** DEPT Q NMR spectrum (150 MHz, $CD_3CN$, 300K) for supramolecule **C3'**.

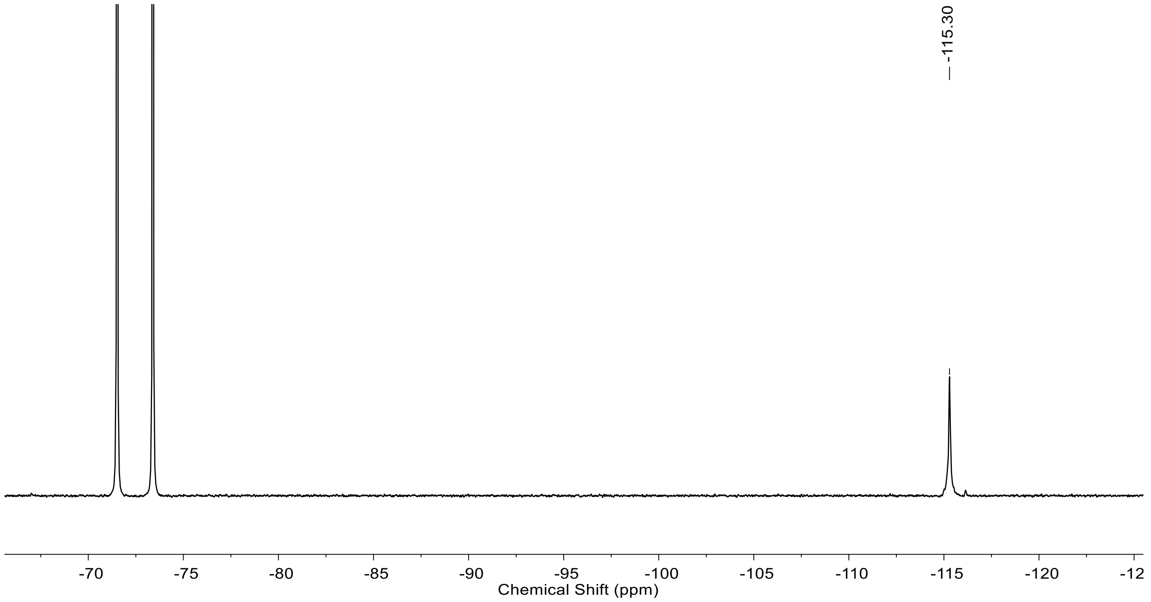


**Supplementary Figure 85.** $^{19}$F NMR spectrum (376 MHz, $CD_3CN$, 300K) for supramolecule **C3'**.

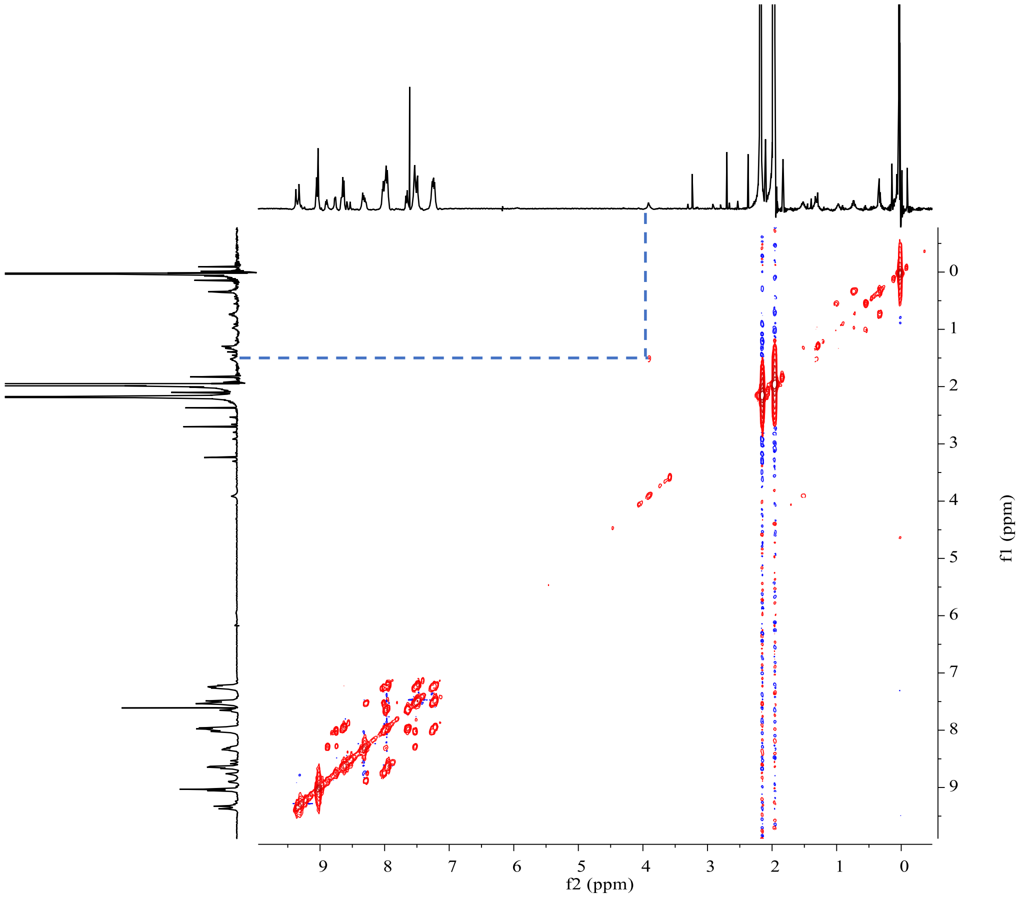


**Supplementary Figure 86.** 2D COSY NMR spectrum (500 MHz, $CD_3CN$, 300K) for supramolecule **C3'**.

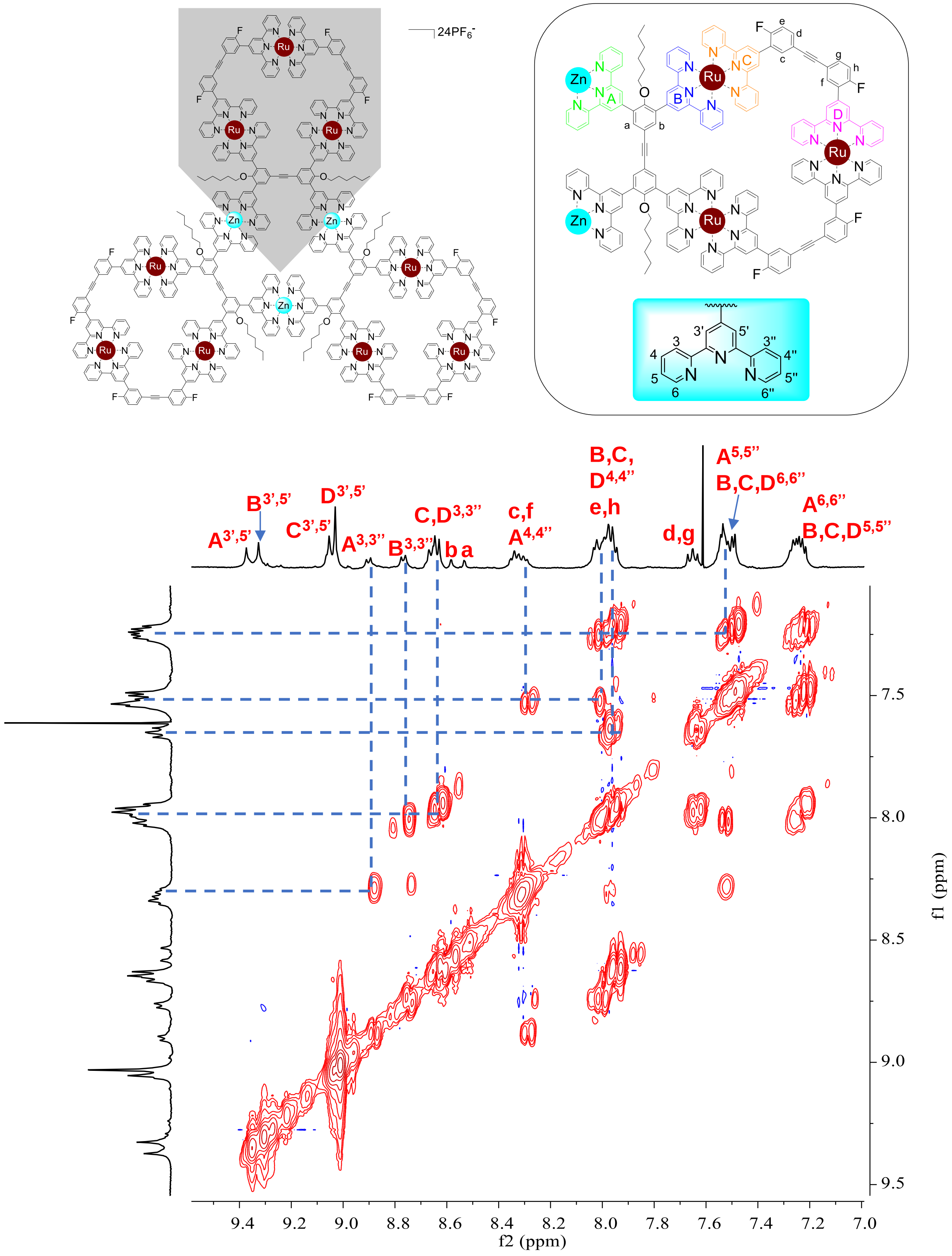

**Supplementary Figure 87.** 2D COSY NMR spectrum (500 MHz, $CD_3CN$, 300K) for supramolecule **C3’** (aromatic region).

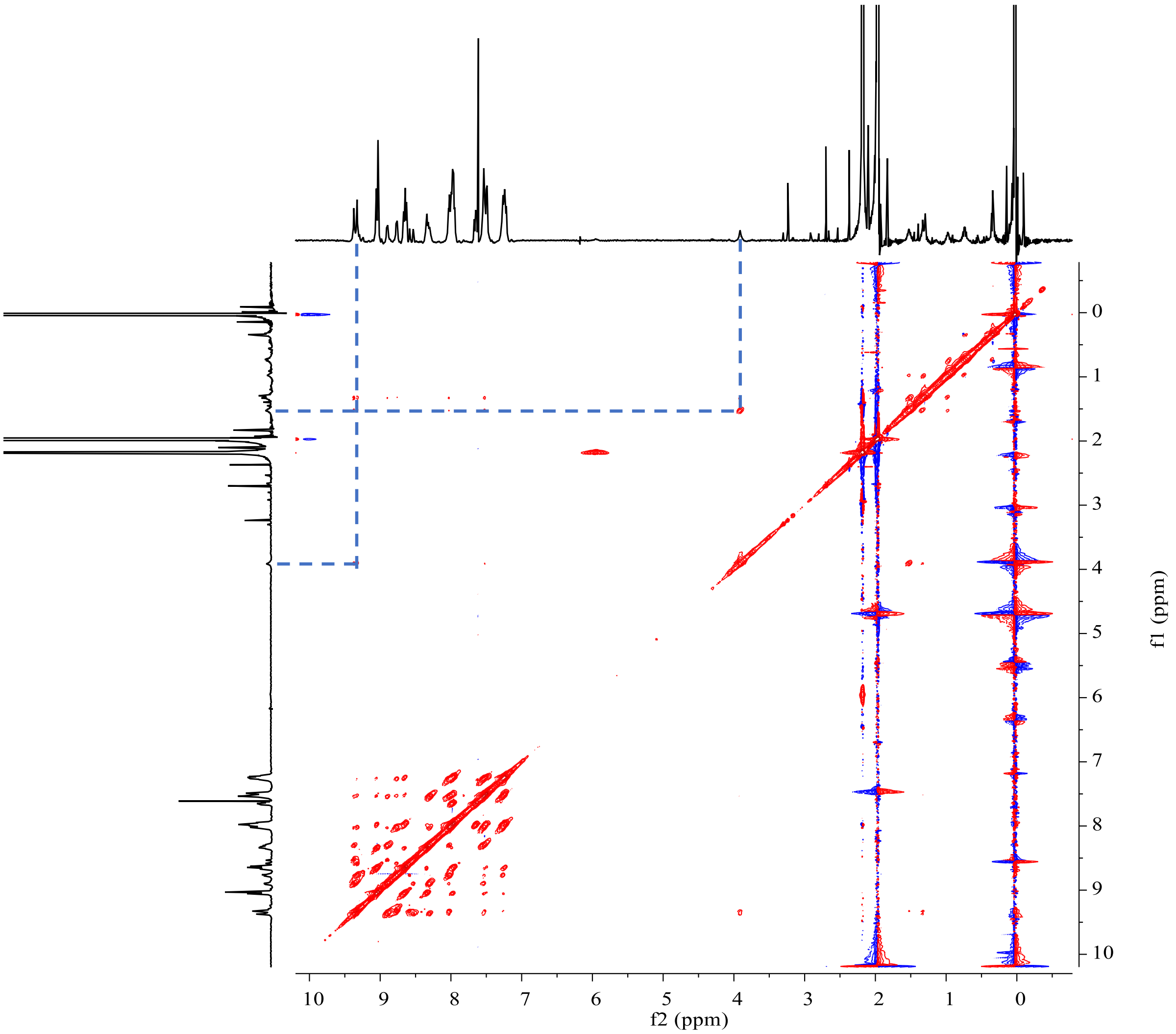


**Supplementary Figure 88.** 2D NOESY NMR spectrum (500 MHz, $CD_3CN$, 300K) for supramolecule **C3'**.

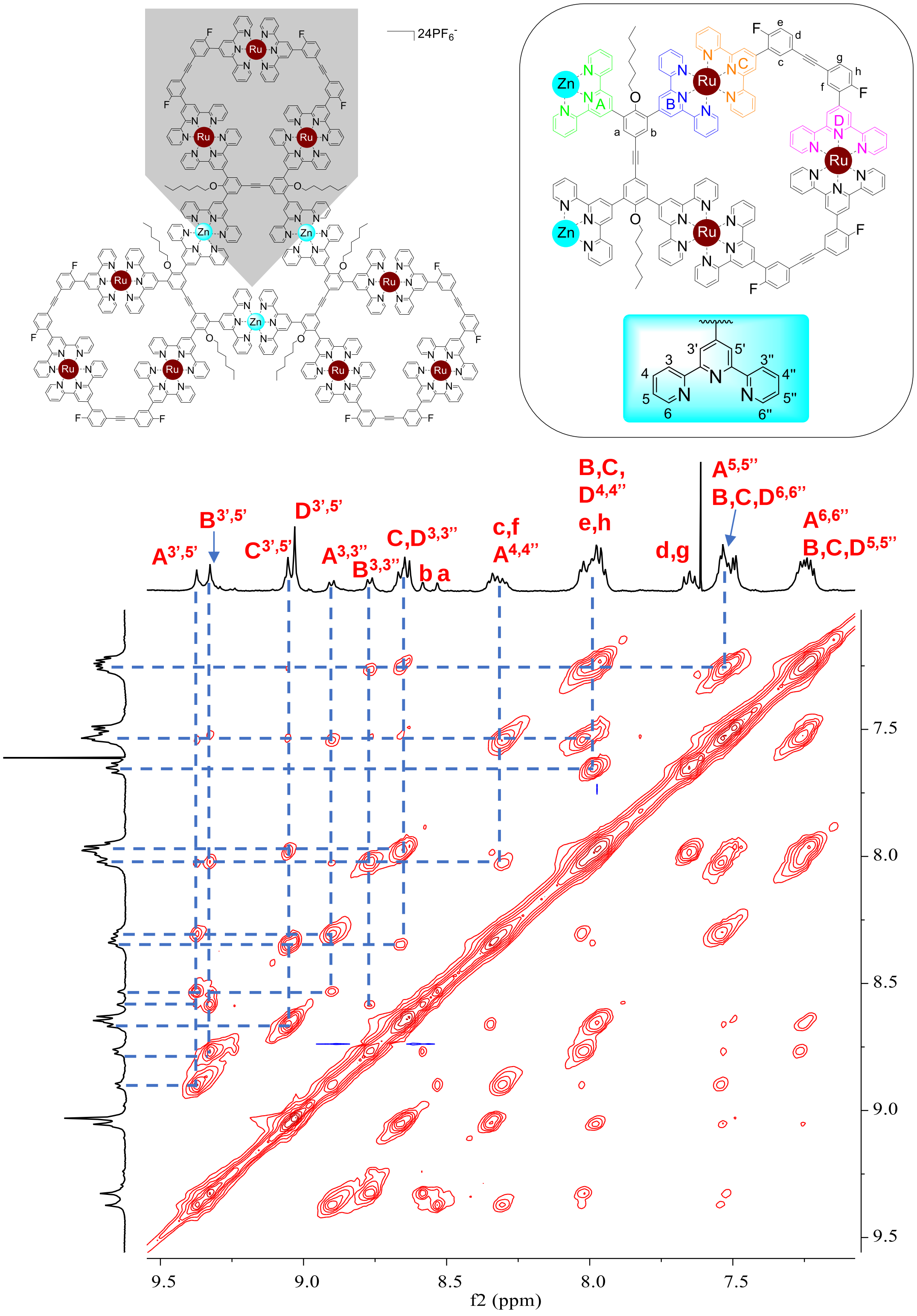

**Supplementary Figure 89.** 2D NOESY NMR spectrum (500 MHz, $CD_3CN$, 300K) for supramolecule **C3’** (aromatic region).

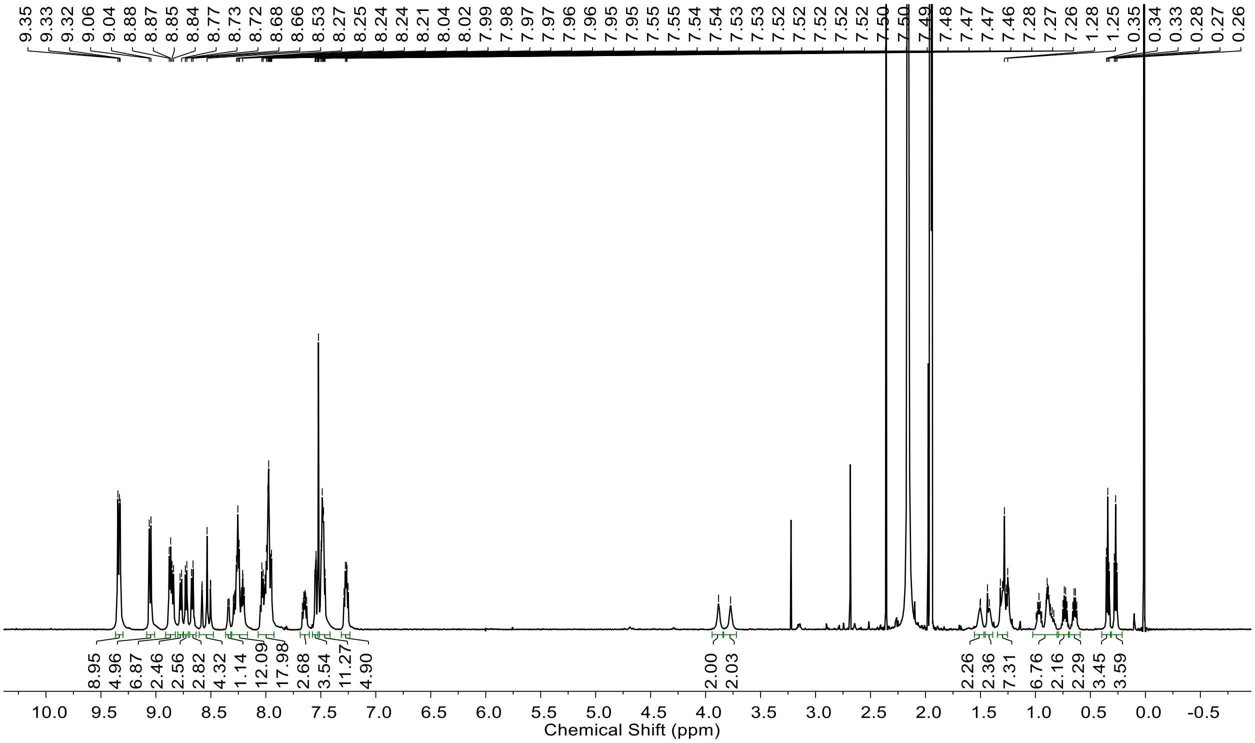


**Supplementary Figure 90.** $^{1}H$ NMR spectrum (600 MHz, $CD_3CN$, 300K) for supramolecule **C4**.

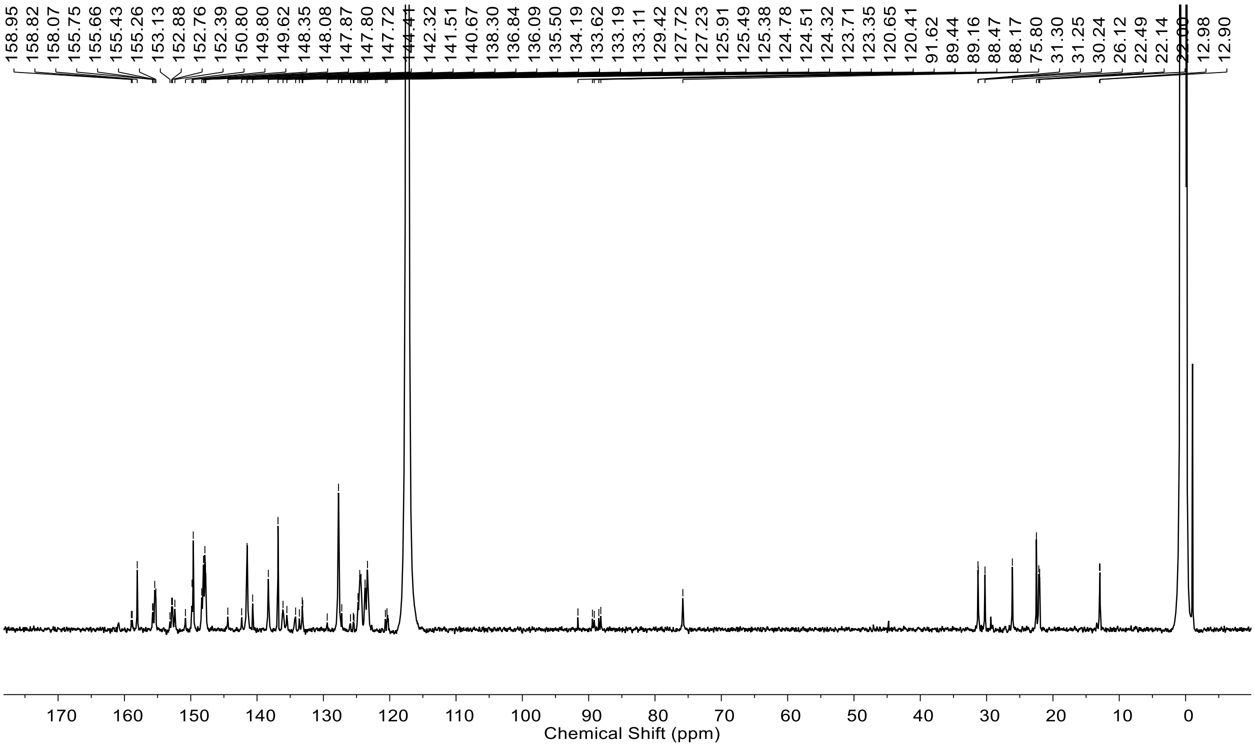


**Supplementary Figure 91.** $^{13}C$ NMR spectrum (125 MHz, $CD_3CN$, 300K) for supramolecule **C4**.

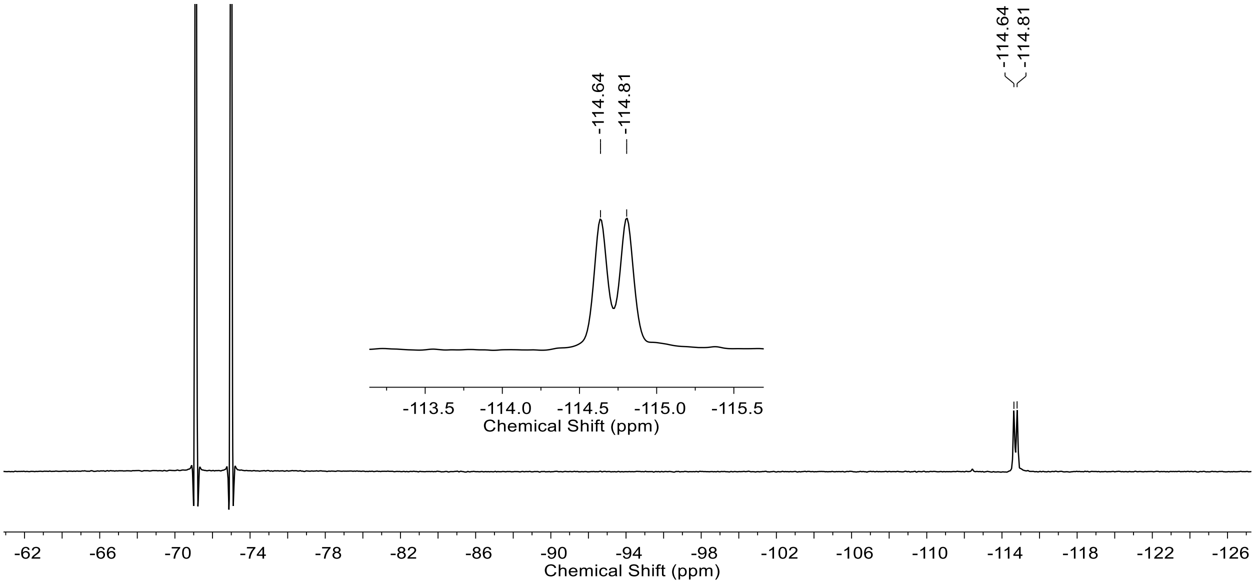


**Supplementary Figure 92.** $^{19}F$ NMR spectrum (376 MHz, $CD_3CN$, 300K) for supramolecule **C4**.

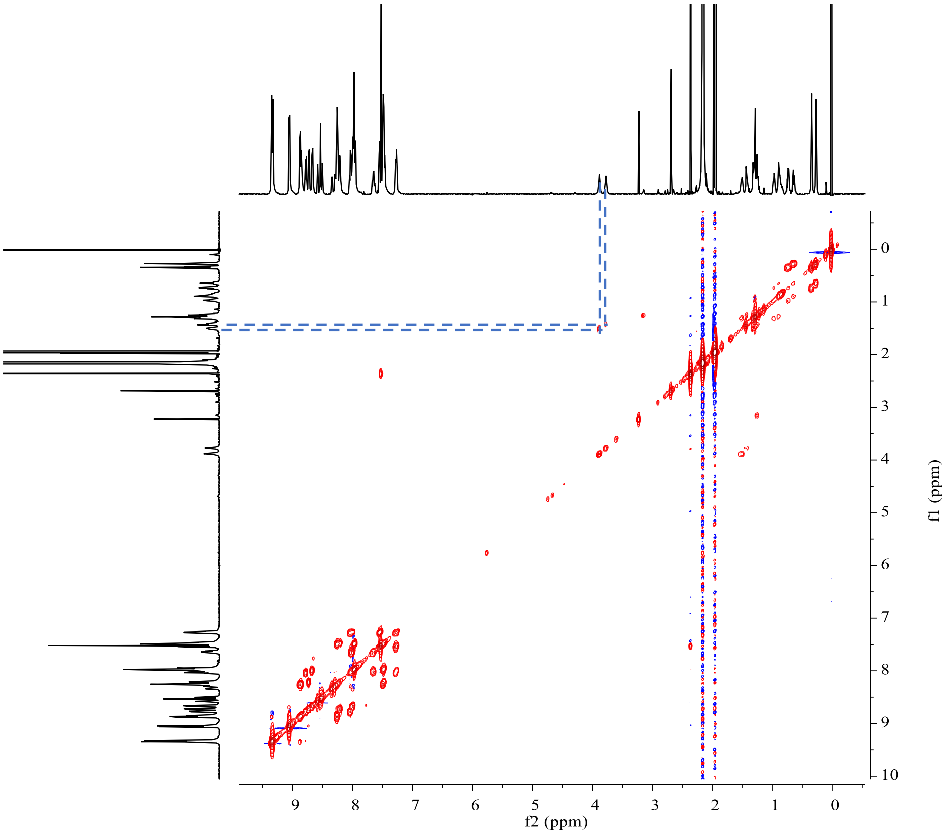


**Supplementary Figure 93.** 2D COSY NMR spectrum (600 MHz, $CD_3CN$, 300K) for supramolecule **C4**.

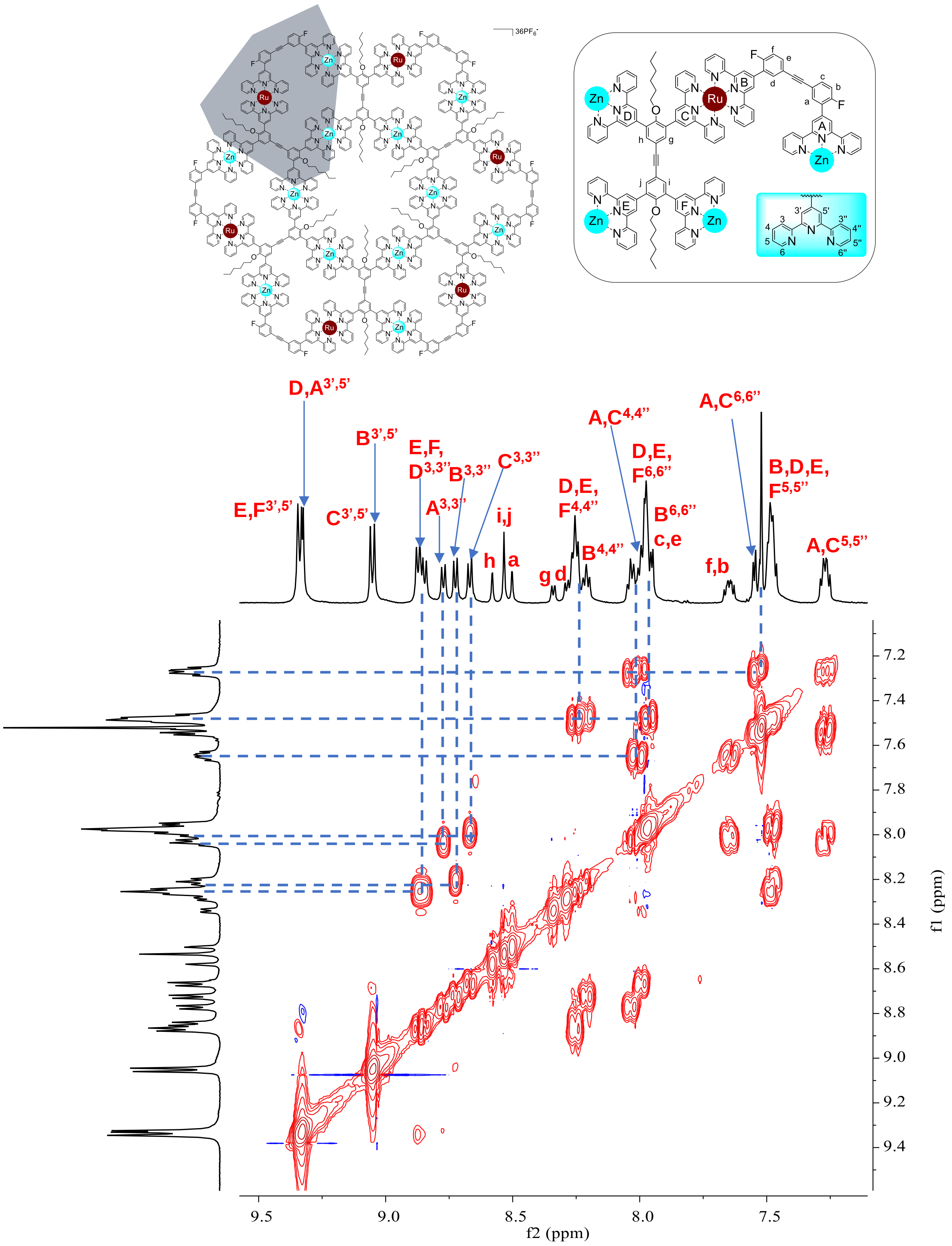


**Supplementary Figure 94.** 2D COSY NMR spectrum (600 MHz, $CD_3CN$, 300K) for supramolecule **C4** (aromatic region).

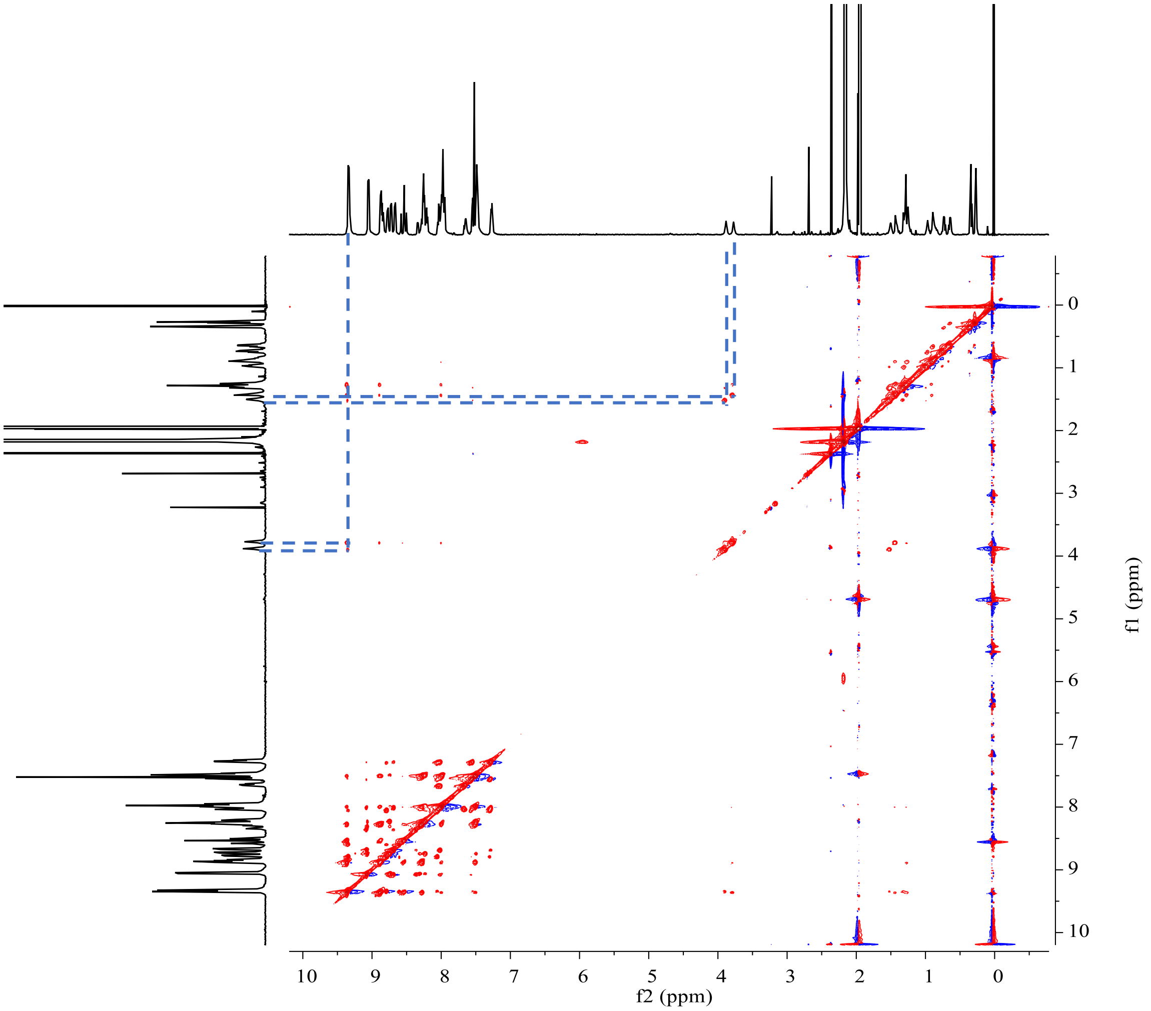


**Supplementary Figure 95.** 2D NOESY NMR spectrum (600 MHz, $CD_3CN$, 300K) for supramolecule **C4**.

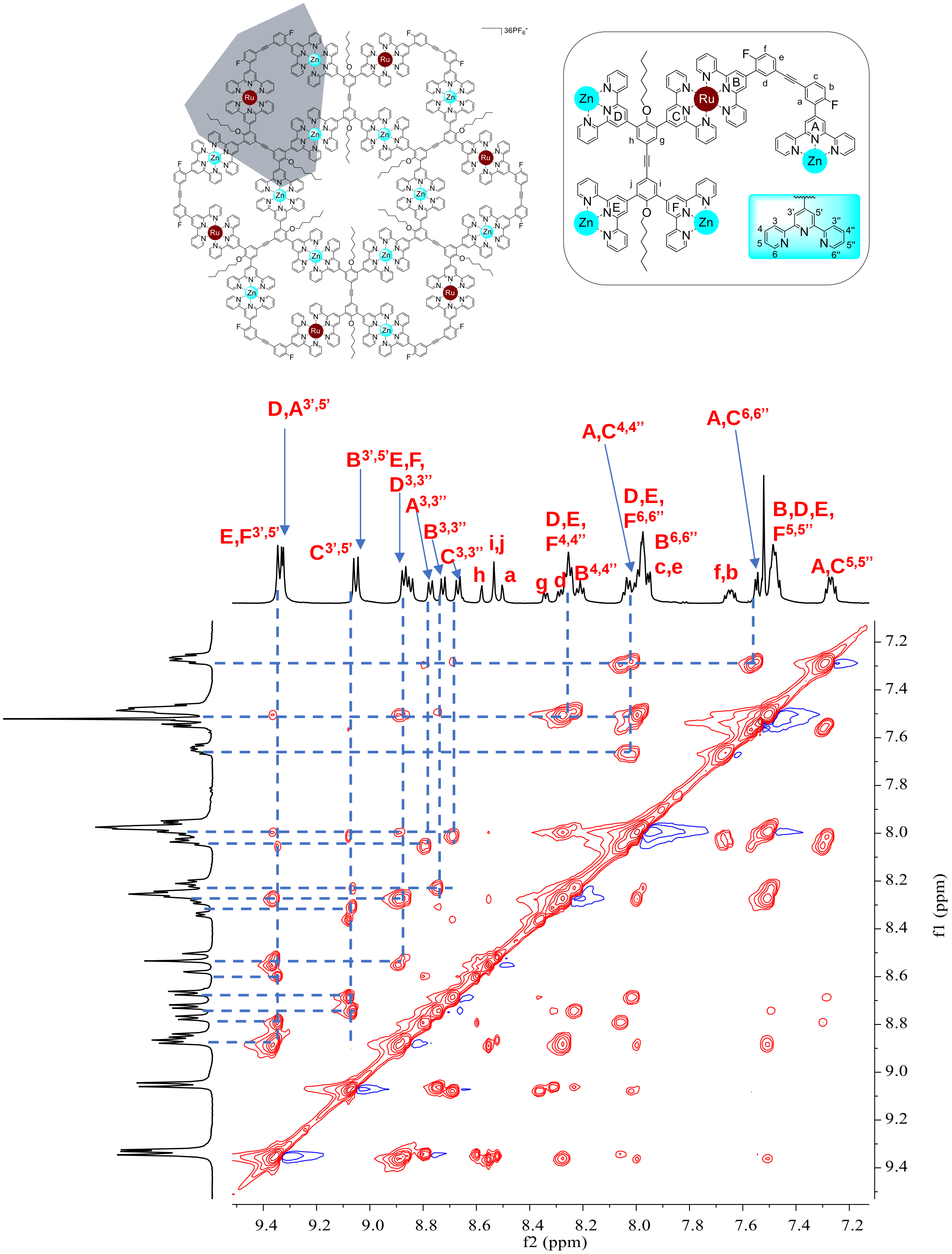


**Supplementary Figure 96.** 2D NOESY NMR spectrum (600 MHz, $CD_3CN$, 300K) for supramolecule **C4** (aromatic region).

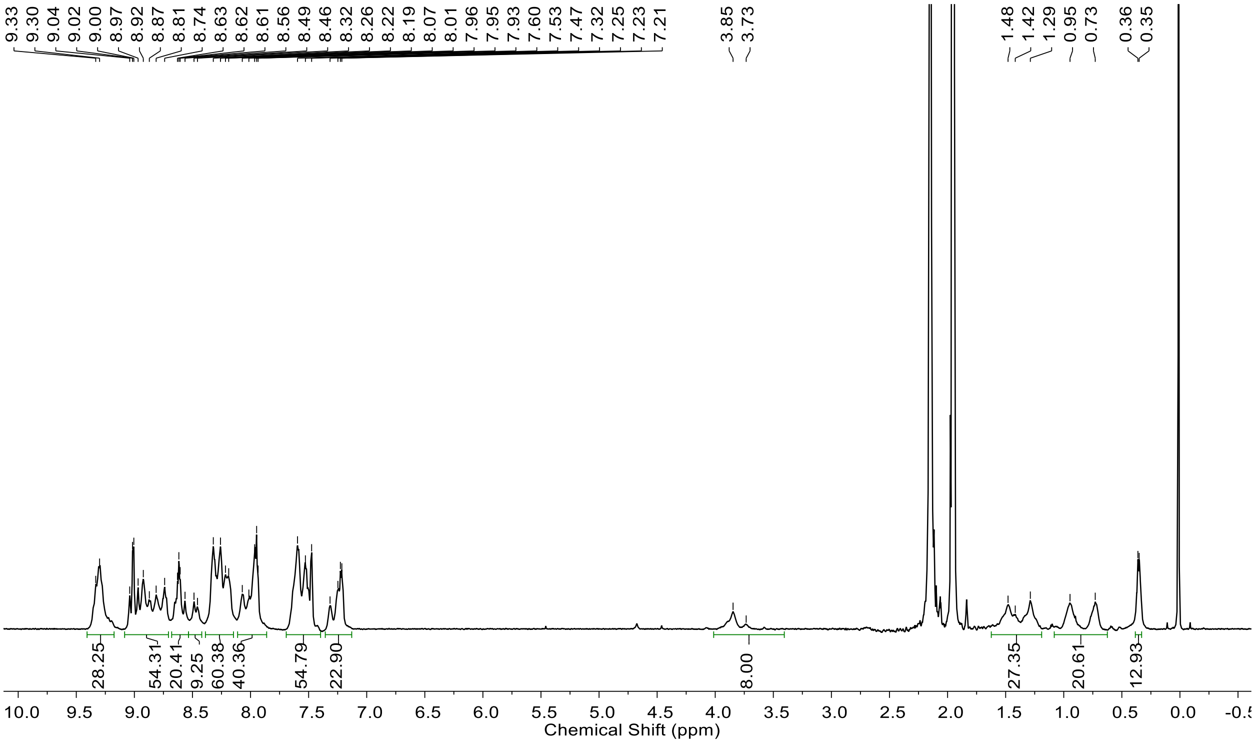


**Supplementary Figure 97.** $^{1}$H NMR spectrum (600 MHz, $CD_3CN$, 300K) for supramolecule **C5**.

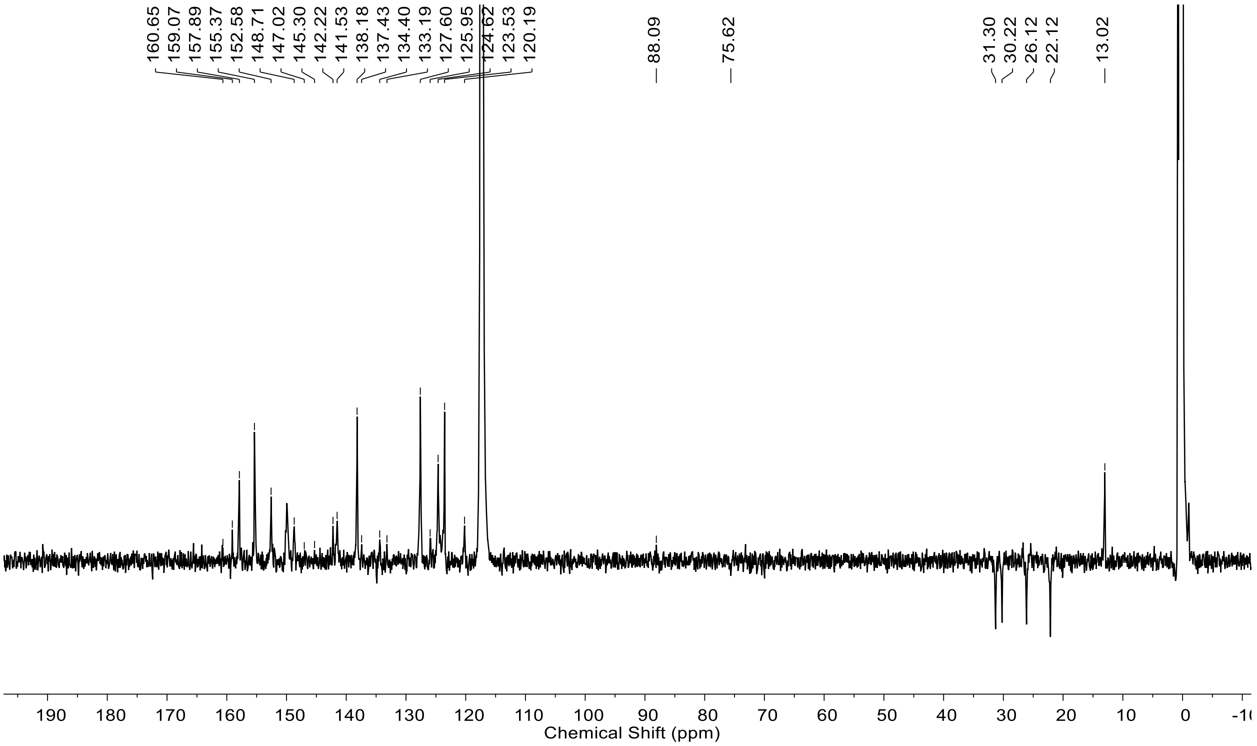


**Supplementary Figure 98.** DEPT Q NMR spectrum (150 MHz, $CD_3CN$, 300K) for supramolecule **C5**.

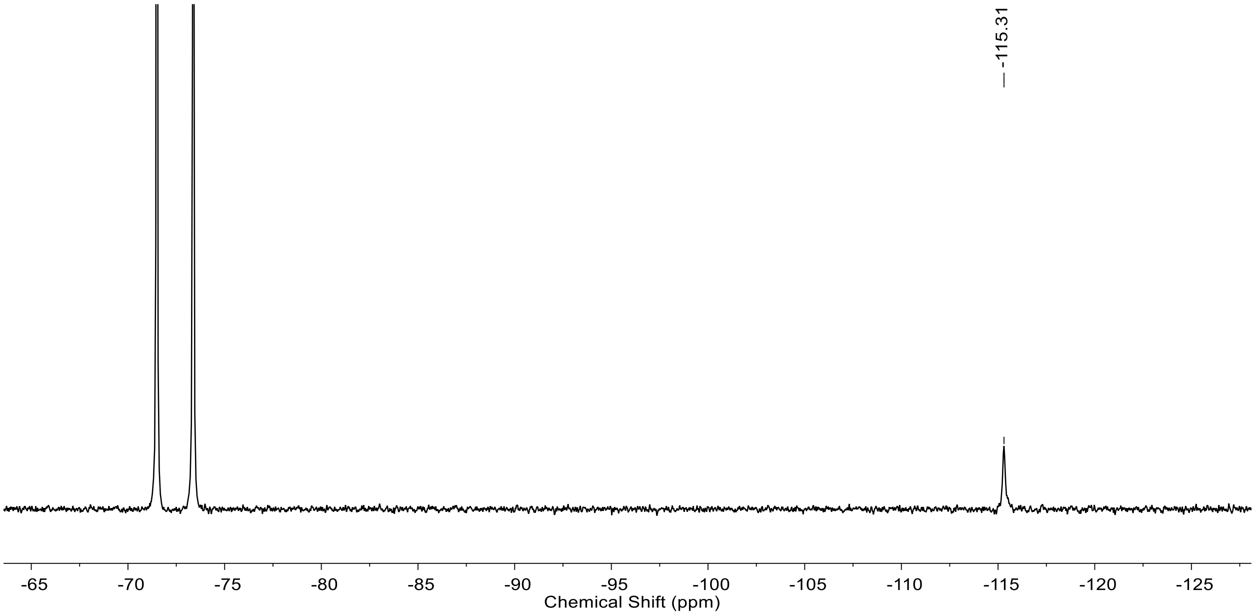


**Supplementary Figure 99.** $^{19}F$ NMR spectrum (376 MHz, $CD_3CN$, 300K) for supramolecule **C5**.

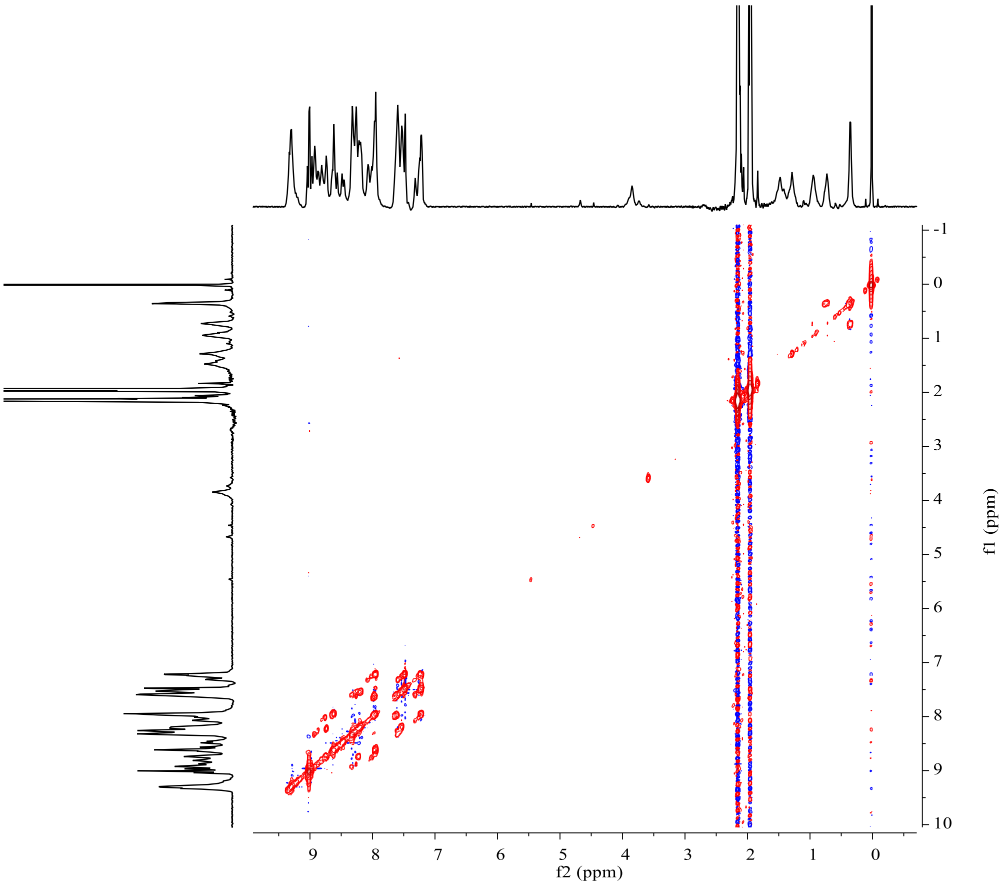


**Supplementary Figure 100.** 2D COSY NMR spectrum (600 MHz, $CD_3CN$, 300K) for supramolecule **C5**.

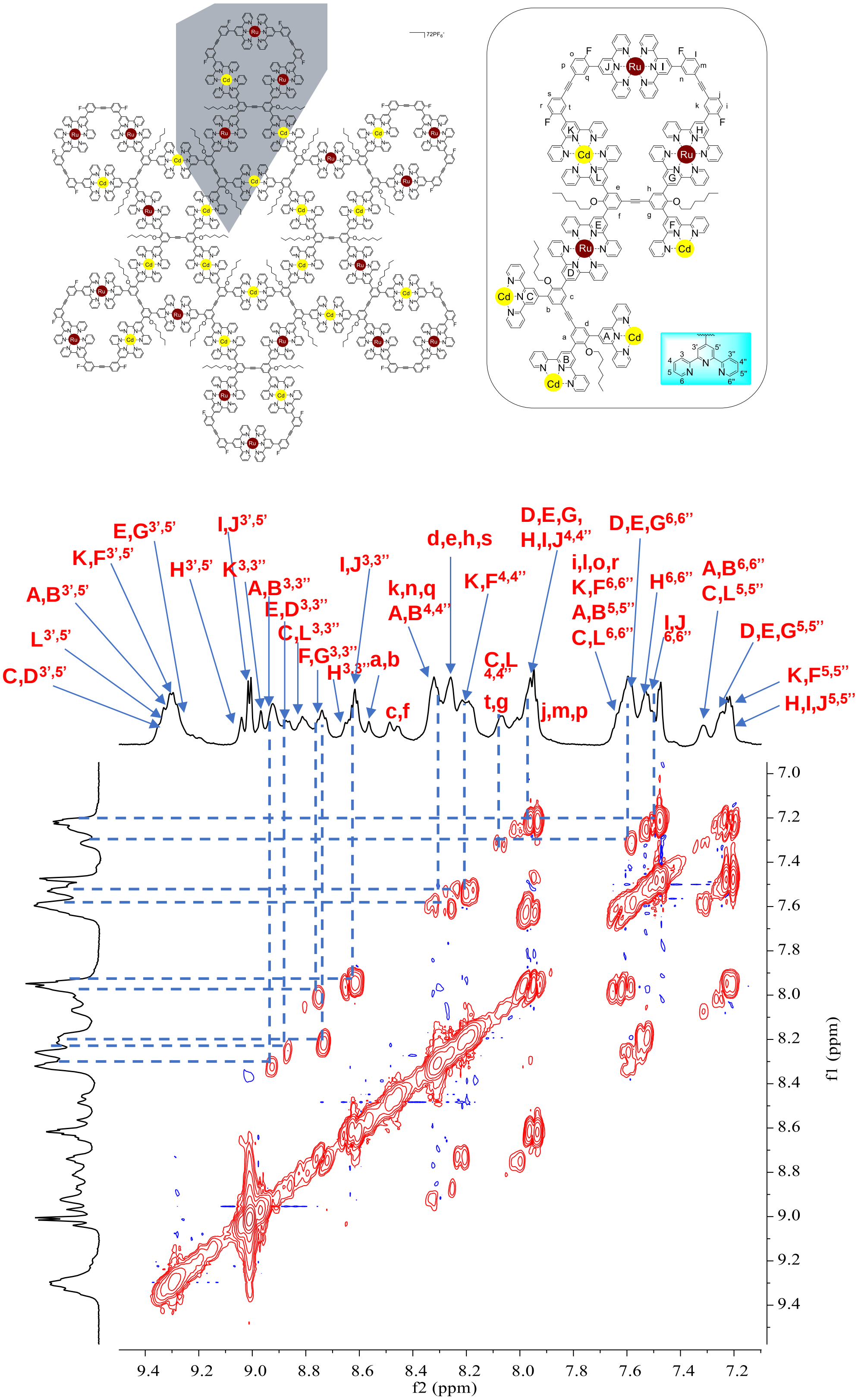


**Supplementary Figure 101.** 2D COSY NMR spectrum (600 MHz, $CD_3CN$, 300K) for supramolecule **C5** (aromatic region).

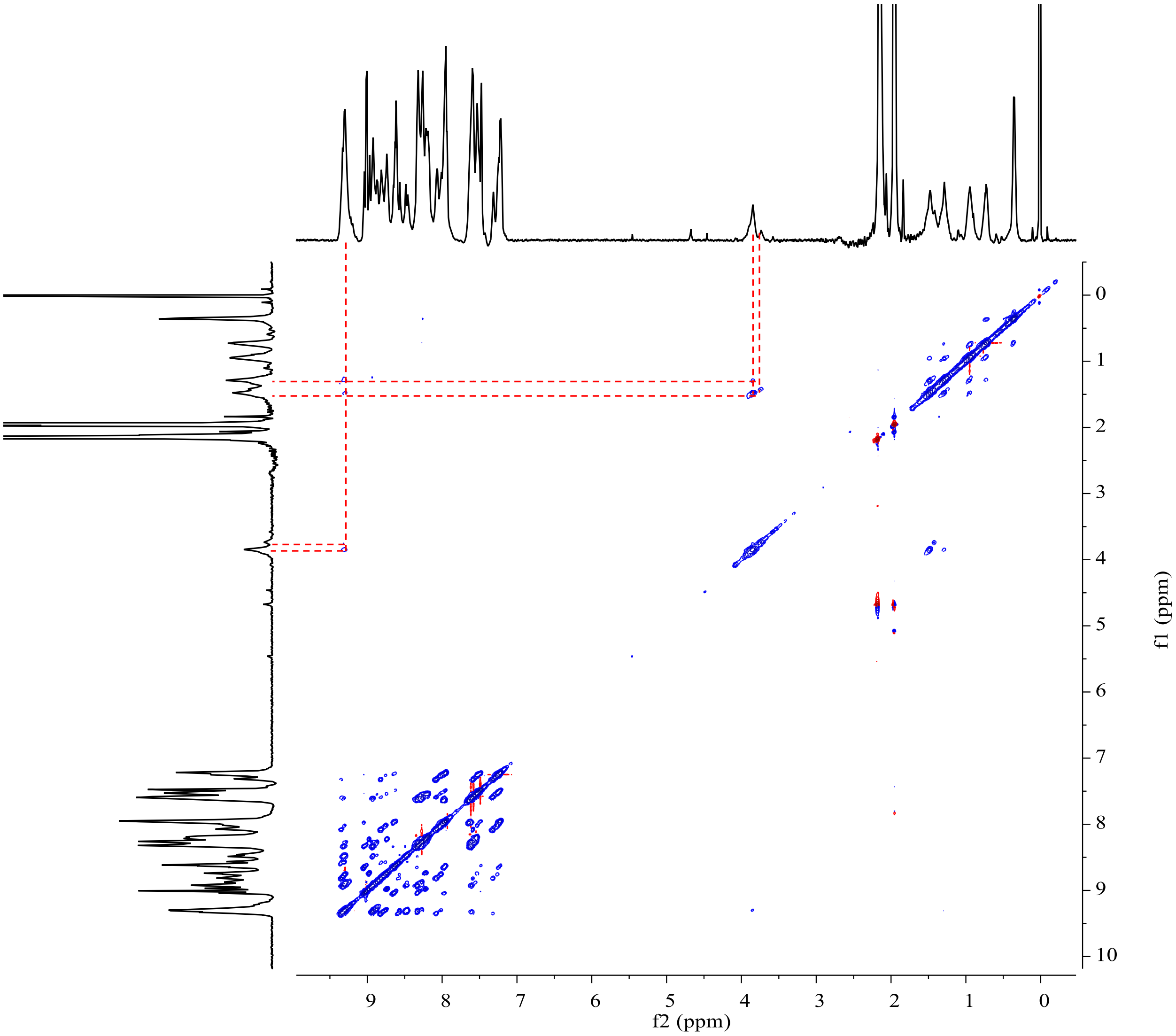


**Supplementary Figure 102.** 2D NOESY NMR spectrum (600 MHz, $CD_3CN$, 300K) for supramolecule **C5**.

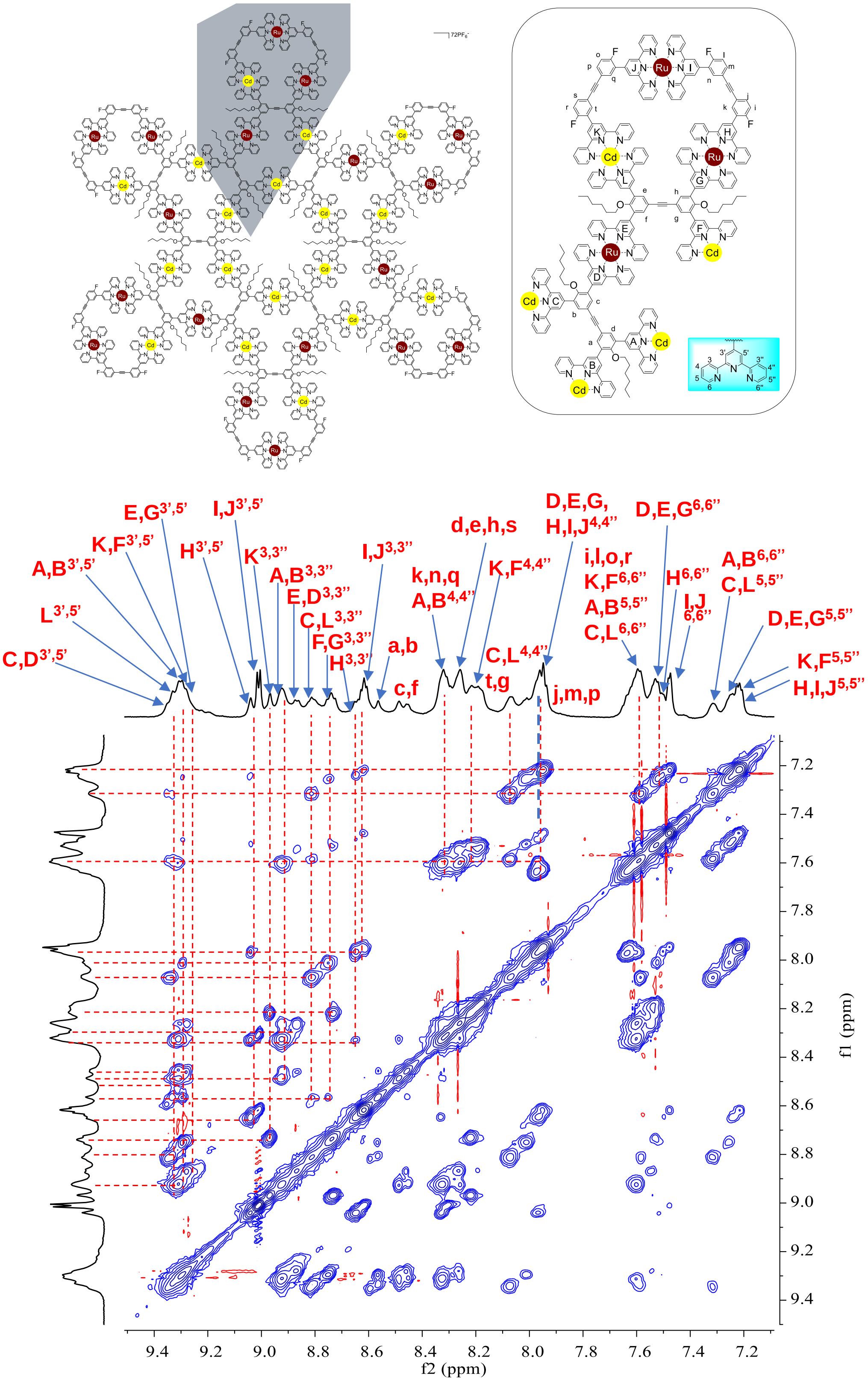

**Supplementary Figure 103.** 2D NOESY NMR spectrum (600 MHz, $CD_3CN$, 300K) for supramolecule **C5** (aromatic region).

## NMR spectra of isomers for compound 15, compound 21

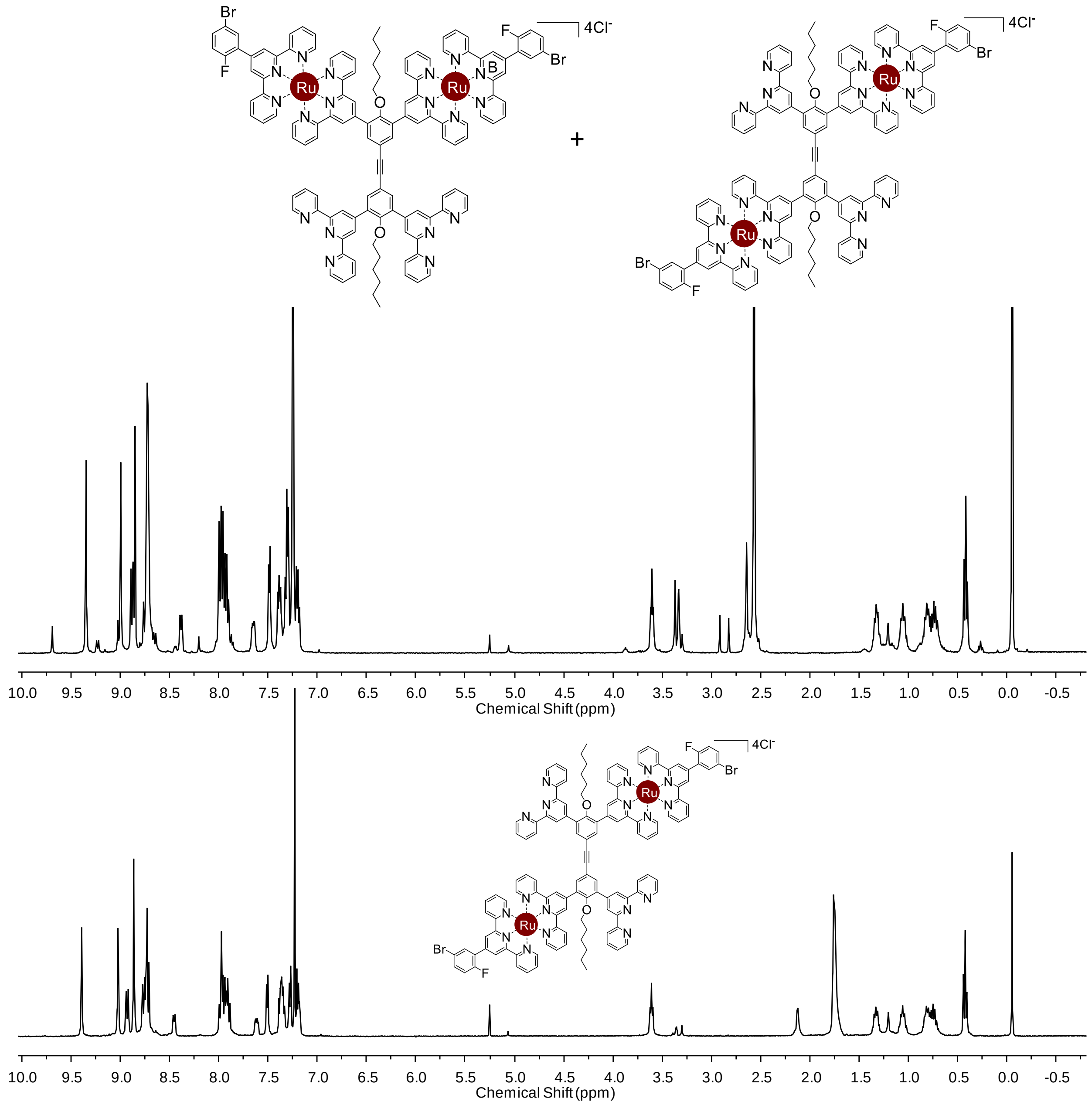


**Supplementary Figure 104.** $^{1}$H NMR (400 MHz, $CDCl_3$, 300K) for mixture of isomers (upper) and compound **15** as trans product after fine column separation (lower).

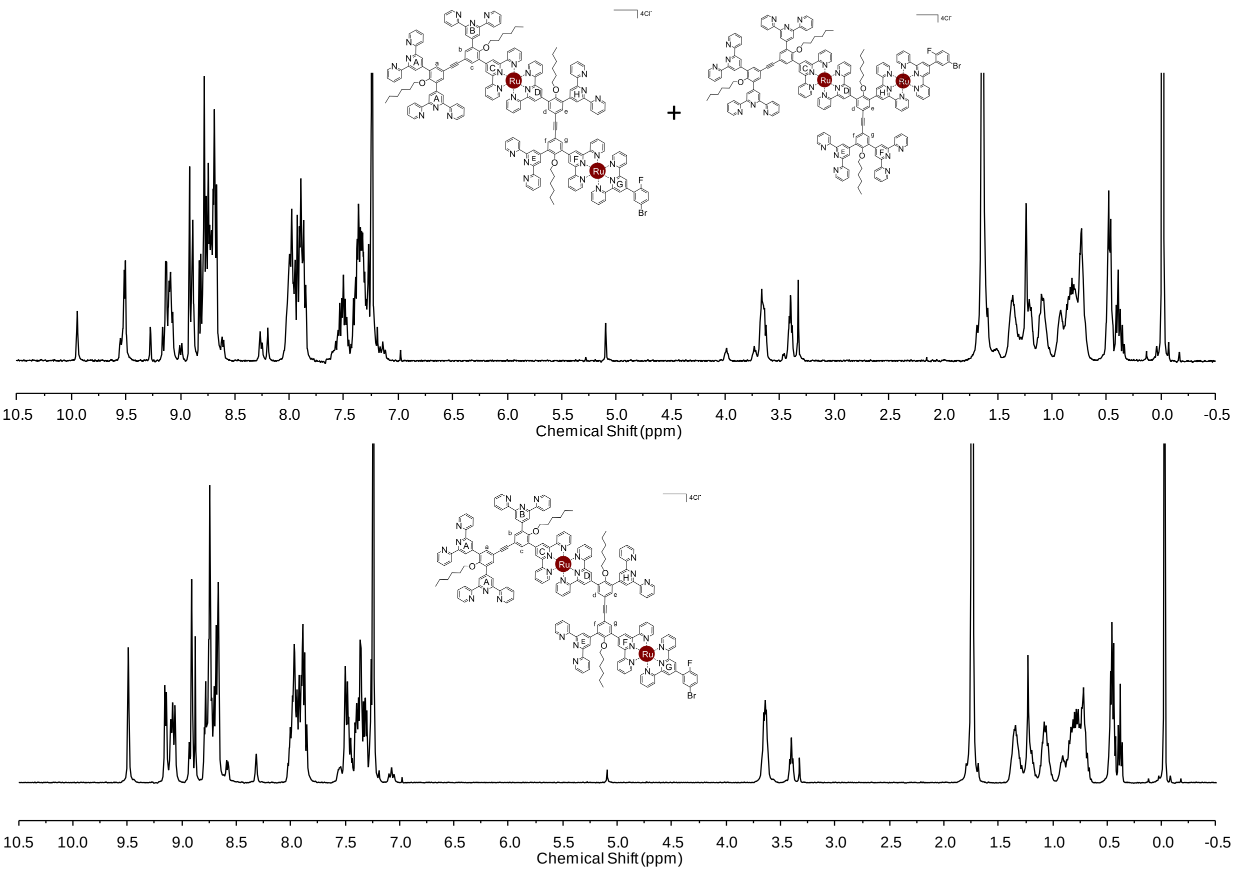


**Supplementary Figure 105.** $^{1}$H NMR (400 MHz, $CDCl_3$, 300K) for mixture of isomers (upper) and compound **21** as trans product after fine column separation (lower).

**$^1$H NMR comparison of ligand L1-L4 with corresponding supramolecules**

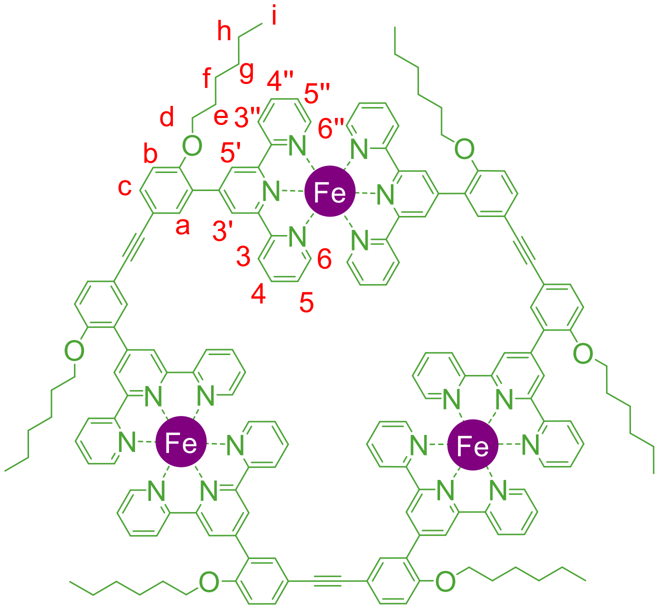


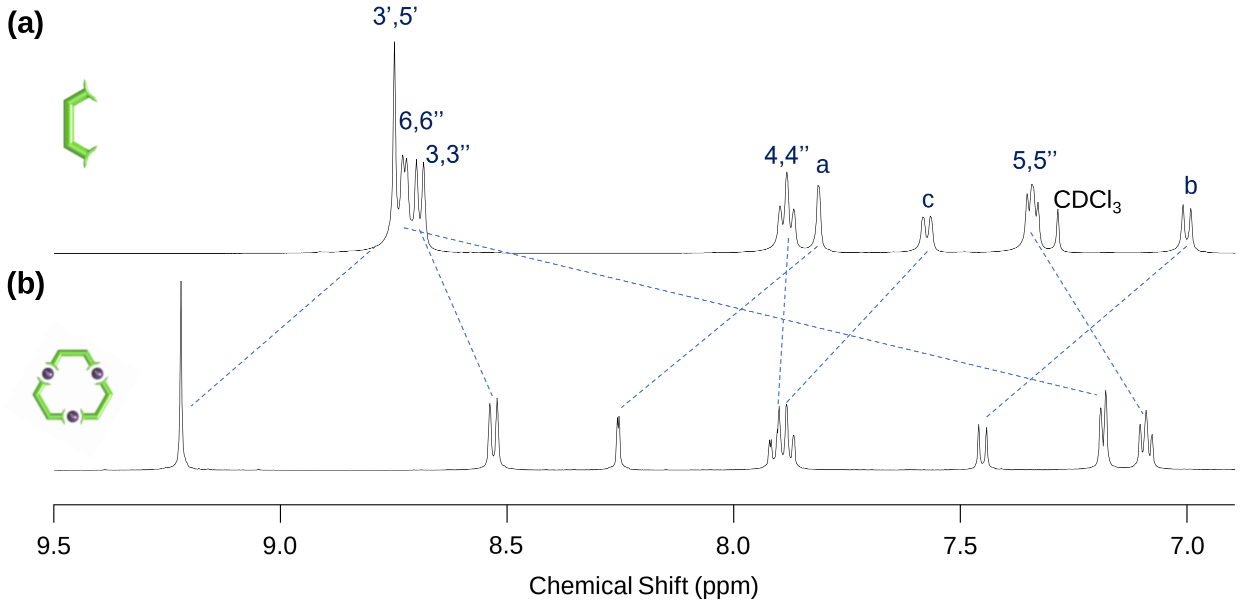


**Supplementary Figure 106.** (a) $^1$H NMR (500 MHz, $CDCl_3$, 300K) for **L1** (aromatic region). (b) $^1$H NMR (500 MHz, $CD_3CN$, 300K) for **C1** (aromatic region).

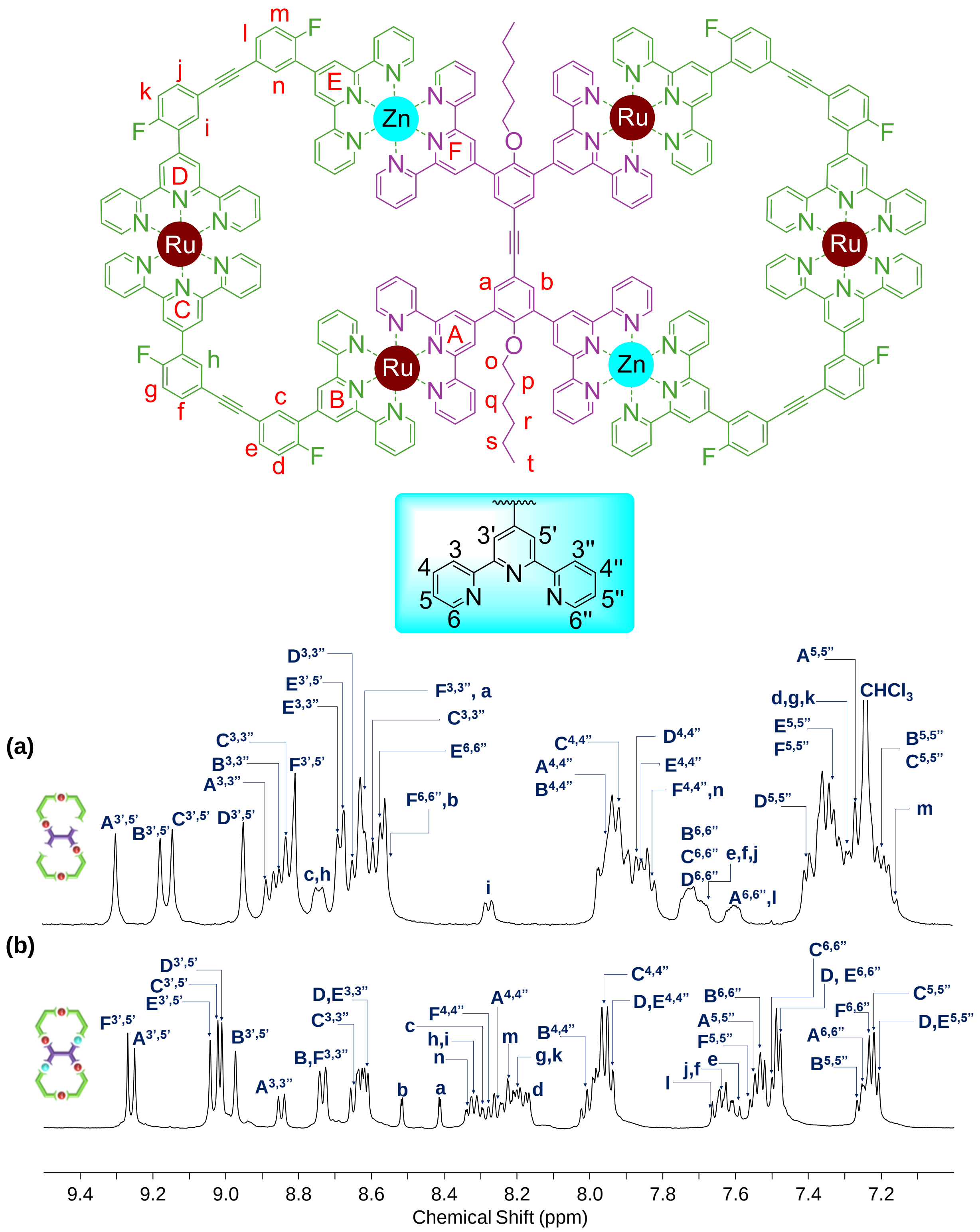


**Supplementary Figure 107.** (a) $^1$H NMR (400 MHz, $CDCl_3$, 300K) for **L2** (aromatic region)**.** (b) $^1$H NMR (600 MHz, $CD_3CN$, 300K) for **C2** (aromatic region)**.**

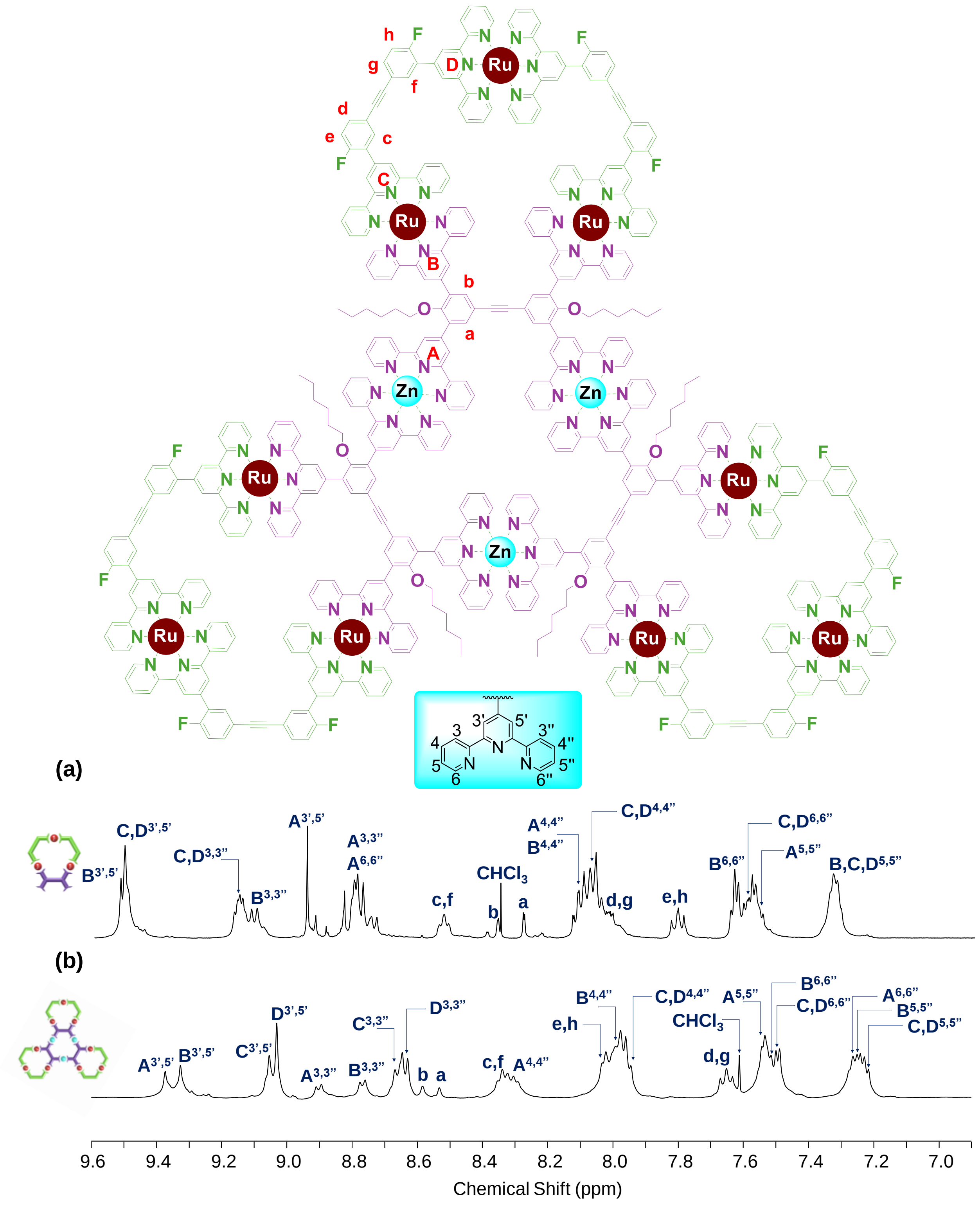


**Supplementary Figure 108.** (a) $^1$H NMR (500 MHz, DMSO-*d*$_6$, 300K) for **L3** (aromatic region)**.** (b) $^1$H NMR (500 MHz, $CD_3CN$, 300K) for **C3** (aromatic region)**.**

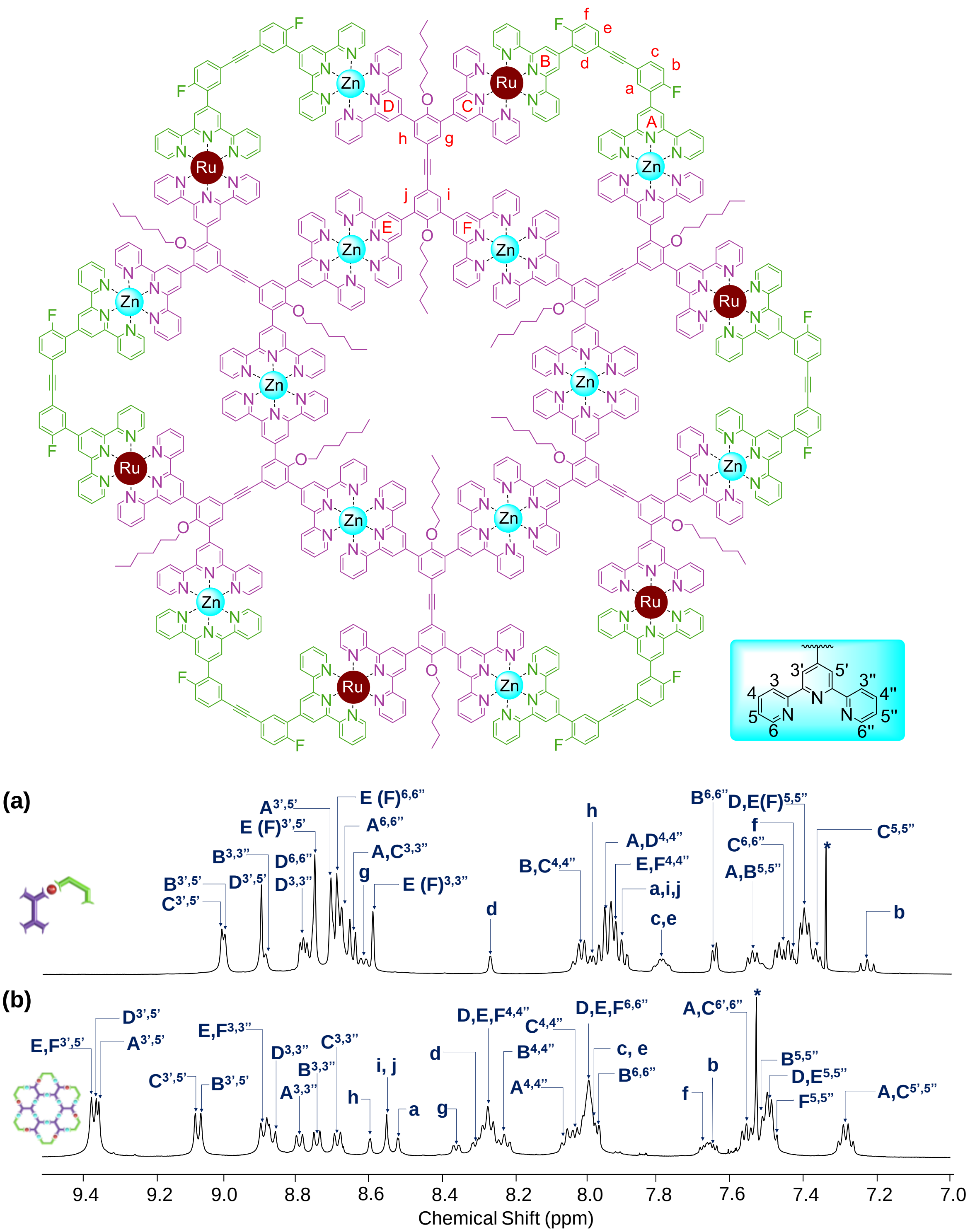


**Supplementary Figure 109.** (a) $^{1}$H NMR (500 MHz, $CDCl_3$, 300K) for **L4** (aromatic region)**.** (b) $^{1}$H NMR (600 MHz, $CD_3CN$, 300K) for **C4** (aromatic region)**.** * represent residue solvent $CHCl_3$.

**DOSY NMR comparison between C3 and C3'**

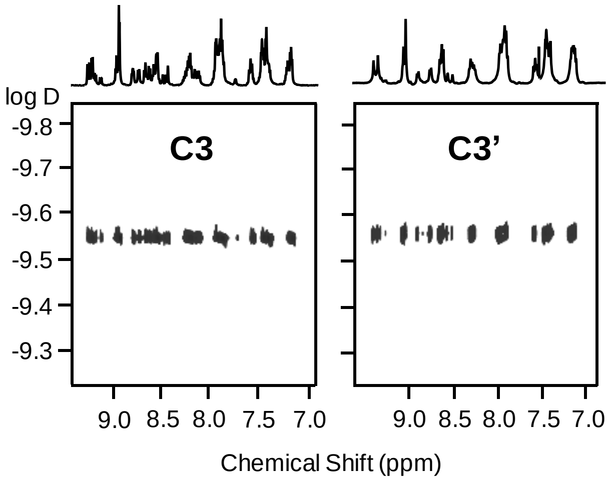


**Supplementary Figure 110.** DOSY NMR (600 MHz, $CD_3CN$, 300K) for **C3** and **C3'**.

**Synthesis of L5' using end-cap strategy and possible products**

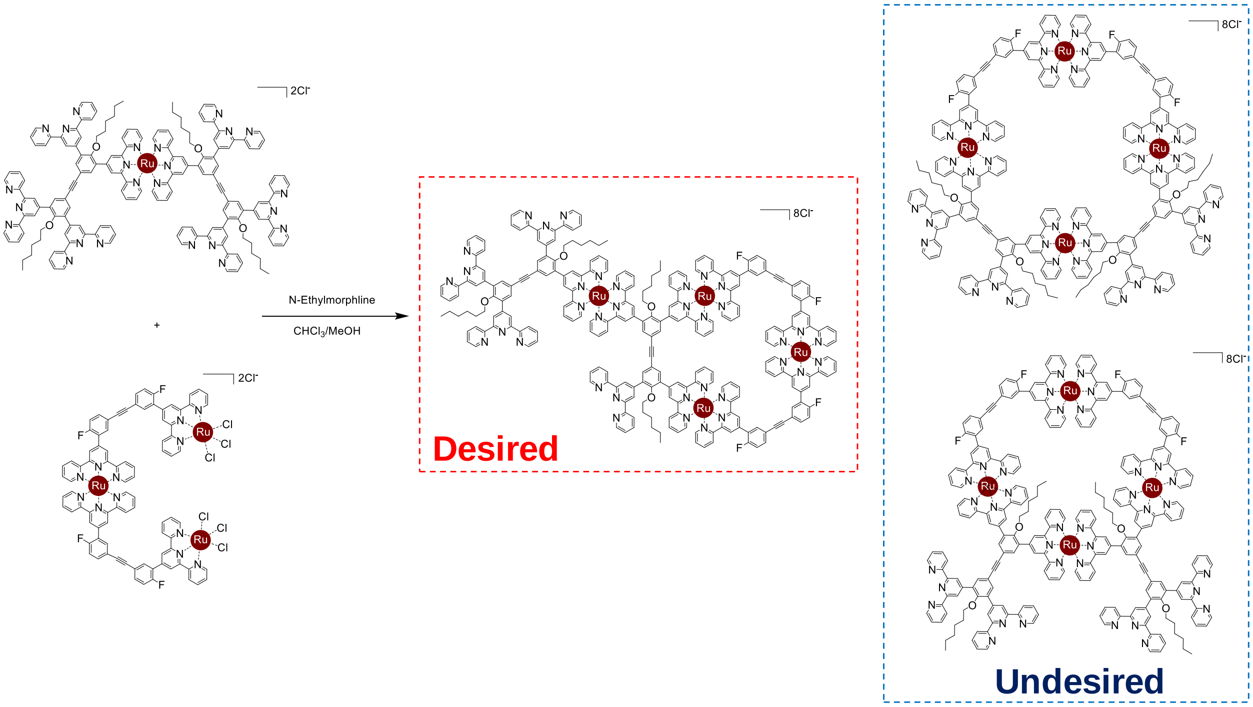


**Supplementary Figure 111.** Synthesis of **L5'** using end-cap strategy and possible products.

## Self-assembly of cis L2 and L5 for C2-like and C5-like supramolecules

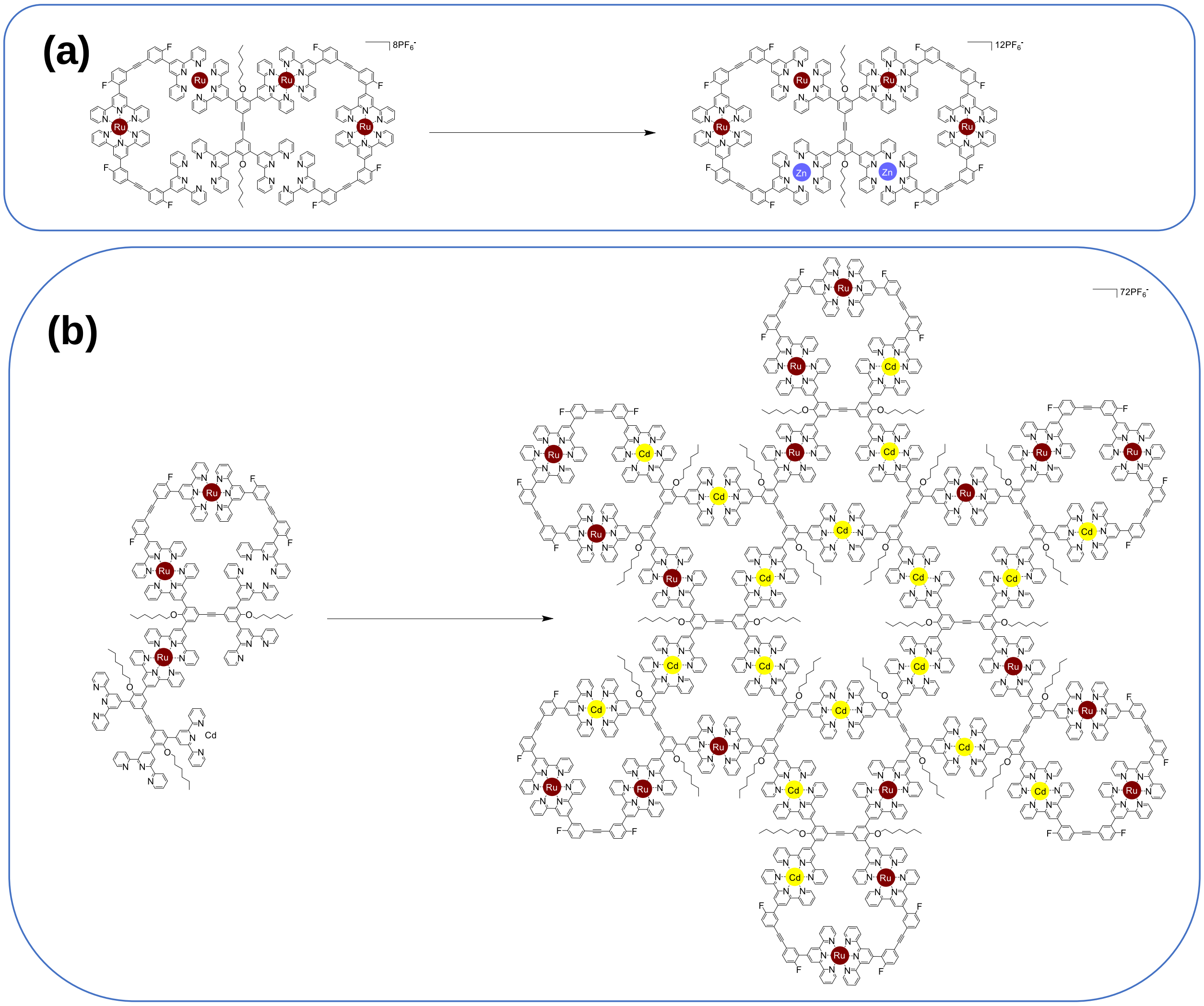


**Supplementary Figure 112.** Self-assembly of *cis*- (a) **L2** and (b) **L5** for **C2**-like and **C5**-like structures, respectively.

**Fitting procedures to derive size parameters from log D[16, 21, 22]**

In sphere model, Stocks-Einstein equation build a relationship between the diffusion coefficient and hydrodynamic radius:

$$D = \frac{k_B T}{6\pi\eta r_h(sp)}$$

Where $k_B$ is the *Boltzmann* constant, T is temperature in Kelvin, $\eta$ is viscosity of the solvent, and $r_h(sp)$ is the hydrodynamic radius.

For large 2D molecules, the sphere model is no longer suitable for direct calculation of hydrodynamic radius. Instead, the oblate spheroid model was used, and the equation can be modified as below:

$$D = \frac{k_B T}{c f_s \pi\eta r_h(ob)}$$

Where

$$c = \frac{6}{1 + 0.695(\frac{r_{vdW}}{r_h(ob)})^{2.234}}$$

$r_{vdW}$ is the van der Waals radius of the solvent, which is far less than the hydrodynamic radius of the supramolecules being investigated. Thus, $c \approx 6$.

As a result, $r_h(ob) = \frac{r_h(sp)}{f_s}$, where $f_s$ is the frictional coefficient, which is derived from $a$ and $b$ in oblate spheroid model as below:

$$f_s = \frac{\sqrt{(\frac{b}{a})^2 - 1}}{(\frac{b}{a})^{\frac{2}{3}} tan^{-1}\sqrt{(\frac{b}{a})^2 - 1}}$$

*a* and *b* are the semiminor and semimajor axis of the oblate spheroid.

The following figures shows the result for hydrodynamic radii of supramolecules **C1-C5**. $k_B$ = $1.38\times10^{-23}$ $m^2kg\ s^{-2}K^{-1}$; $\eta$ = 0.000367 Pa s; T = 300K. *r*(cal) is the radius of a sphere with an equivalent volume as the spheroid generated by a and b; since spheroid volume (V) has the relationship with a and b: $V = \frac{4}{3}\pi ab^2$, and $r(\text{cal}) = \sqrt[3]{\frac{3V}{4\pi}}$, *r*(cal) can be calculated as: $r(\text{cal}) = \sqrt[3]{ab^2}$ . $r_h$(ob)/*r*(cal) is the ratio of the hydrodynamic radius generated from the oblate model and the equivalent radius generated by the volume of the spheroid; this ratio was used to guide the adjustment of a and b until the volume generated by them matches the volume generated by $r_h$(ob) ($r_h$(ob)/*r*(cal) ≈ 1).

| | | | | | | | | | |
|---|---|---|---|---|---|---|---|---|---|
| **C1** | | | | | | | | | |
| Acetonitrile | | $k_B$ | 1.38E-23 | | | | | | |
| Oblate | b>a | p>1 | | | | | | | |
| | | | | | | | | | |
| | | | log D | | | | | | |
| D | 5.66E-10 | | -9.24718 | | | | | | |
| T (K) | 300 | | | | | | | | |
| η(Pa*s) | 0.000367 | | | | | | | | |
| rh(sp) = kbT/6πDη | | 1.06E-09 | | | | | | | |
| | b | a | rh(sp) | rh(ob) | fs | r(cal) | p=b/a | 1/p | r(cal)/rh(ob) |
| | 1.12E-09 | 1.1E-09 | 1.06E-09 | 1.06E-09 | 1.00003 | 1.11E-09 | 1.01818 | 0.98214 | 1.05 |

**Supplementary Figure 113.** Screenshot of Microsoft Excel spreadsheet used to fit **C1** in $CD_3CN$ using oblate spheroid model.

| C2 | | | | | | | | | |
|---|---|---|---|---|---|---|---|---|---|
| Acetonitrile | | $k_B$ | 1.38E-23 | | | | | | |
| Oblate | b>a | p>1 | | | | | | | |
| | | | | | | | | | |
| | | | log D | | | | | | |
| D | 4.01E-10 | | -9.39686 | | | | | | |
| T (K) | 300 | | | | | | | | |
| η(Pa*s) | 0.000367 | | | | | | | | |
| rh(sp) = kbT/6πDη | | 1.49E-09 | | | | | | | |
| | b | a | rh(sp) | rh(ob) | fs | r(cal) | p=b/a | 1/p | r(cal)/rh(ob) |
| | 1.75E-09 | 1.1E-09 | 1.49E-09 | 1.46E-09 | 1.01892 | 1.5E-09 | 1.59091 | 0.62857 | 1.03 |

**Supplementary Figure 114.** Screenshot of Microsoft Excel spreadsheet used to fit **C2** in $CD_3CN$ using oblate spheroid model.

| C3 | | | | | | | | | |
|---|---|---|---|---|---|---|---|---|---|
| Acetonitrile | | $k_B$ | 1.38E-23 | | | | | | |
| Oblate | b>a | p>1 | | | | | | | |
| | | | | | | | | | |
| | | | log D | | | | | | |
| D | 2.76E-10 | | -9.55909 | | | | | | |
| T (K) | 300 | | | | | | | | |
| η(Pa*s) | 0.000367 | | | | | | | | |
| rh(sp) = kbT/6πDη | | 2.17E-09 | | | | | | | |
| | b | a | rh(sp) | rh(ob) | fs | r(cal) | p=b/a | 1/p | r(cal)/rh(ob) |
| | 2.8E-09 | 1.1E-09 | 2.17E-09 | 2.02E-09 | 1.07587 | 2.05E-09 | 2.5455 | 0.39286 | 1.02 |

**Supplementary Figure 115.** Screenshot of Microsoft Excel spreadsheet used to fit **C3** in $CD_3CN$ using oblate spheroid model.

| C3’ | | | | | | | | | |
|---|---|---|---|---|---|---|---|---|---|
| Acetonitrile | | $k_B$ | 1.38E-23 | | | | | | |
| Oblate | b>a | p>1 | | | | | | | |
| | | | | | | | | | |
| | | | log D | | | | | | |
| D | 2.7E-10 | | -9.56864 | | | | | | |
| T (K) | 300 | | | | | | | | |
| η(Pa*s) | 0.000367 | | | | | | | | |
| rh(sp) = kbT/6πDη | | 2.22E-09 | | | | | | | |
| | b | a | rh(sp) | rh(ob) | fs | r(cal) | p=b/a | 1/p | r(cal)/rh(ob) |
| | 2.8E-09 | 1.1E-09 | 2.22E-09 | 2.06E-09 | 1.07587 | 2.05E-09 | 2.54545 | 0.39286 | 0.99 |

**Supplementary Figure 116.** Screenshot of Microsoft Excel spreadsheet used to fit **C3’** in $CD_3CN$ using oblate spheroid model.

| | | | | | | | | | | |
|---|---|---|---|---|---|---|---|---|---|---|
| **C4** | | | | | | | | | | |
| Acetonitrile | | $k_B$ | 1.38E-23 | | | | | | | |
| Oblate | b>a | p>1 | | | | | | | | |
| | | | | | | | | | | |
| | | | log D | | | | | | | |
| D | 2.32E-10 | | -9.63451202 | | | | | | | |
| T (K) | 300 | | | | | | | | | |
| η(Pa*s) | 0.000367 | | | | | | | | | |
| rh(sp) = kbT/6πDη | | 2.58E-09 | | | | | | | | |
| | b | a | rh(sp) | rh(ob) | fs | r(cal) | p=b/a | 1/p | r(cal)/rh(ob) | |
| | 3.4E-09 | 1.1E-09 | 2.58E-09 | 2.32E-09 | 1.11036 | 2.33E-09 | 3.09091 | 0.32353 | 1.005 | |

**Supplementary Figure 117.** Screenshot of Microsoft Excel spreadsheet used to fit **C4** in $CD_3CN$ using oblate spheroid model.

| | | | | | | | | | | |
|---|---|---|---|---|---|---|---|---|---|---|
| **C5** | | | | | | | | | | |
| Acetonitrile | | $k_B$ | 1.38E-23 | | | | | | | |
| Oblate | b>a | p>1 | | | | | | | | |
| | | | | | | | | | | |
| | | | log D | | | | | | | |
| D | 1.56E-10 | | -9.80688 | | | | | | | |
| T (K) | 300 | | | | | | | | | |
| η(Pa*s) | 0.000367 | | | | | | | | | |
| rh(sp) = kbT/6πDη | | 3.84E-09 | | | | | | | | |
| | b | a | rh(sp) | rh(ob) | fs | r(cal) | p=b/a | 1/p | r(cal)/rh(ob) | |
| | 5.4E-09 | 1.1E-09 | 3.84E-09 | 3.15E-09 | 1.2184 | 3.18E-09 | 4.90909 | 0.2037 | 1.008 | |

**Supplementary Figure 118.** Screenshot of Microsoft Excel spreadsheet used to fit **C5** in $CD_3CN$ using oblate spheroid model.

**Supplementary References**


1. Porras JA, Mills IN, Transue WJ, Bernhard S. Highly Fluorinated Ir(III)–2,2′:6′,2″-Terpyridine–Phenylpyridine–X Complexes via Selective C–F Activation: Robust Photocatalysts for Solar Fuel Generation and Photoredox Catalysis. *J. Am. Chem. Soc.* **138**, 9460-9472 (2016).

2. Thalassinos K, Grabenauer M, Slade SE, Hilton GR, Bowers MT, Scrivens JH. Characterization of Phosphorylated Peptides Using Traveling Wave-Based and Drift Cell Ion Mobility Mass Spectrometry. *Anal. Chem.* **81**, 248-254 (2009).

3. Shvartsburg AA, Liu B, Siu KWM, Ho K-M. Evaluation of Ionic Mobilities by Coupling the Scattering on Atoms and on Electron Density. *J. Phys. Chem. A* **104**, 6152-6157 (2000).

4. Newkome GR, Cho TJ, Moorefield CN, Baker GR, Cush R, Russo PS. Self- and Directed Assembly of Hexaruthenium Macrocycles. *Angew. Chem. Int. Ed.* **38**, 3717-3721 (1999).

5. Newkome GR, *et al.* Nanoassembly of a Fractal Polymer: A Molecular "Sierpinski Hexagonal Gasket". *Science* **312**, 1782-1785 (2006).

6. Schultz A, *et al.* Dondorff Rings: Synthesis, Isolation, and Properties of 60°-Directed Bisterpyridine-Based Folded Tetramers. *Chem. Eur. J.* **18**, 11569-11572 (2012).

7. El-Batal H, Guo K, Li X, Wesdemiotis C, Moorefield CN, Newkome GR. Perylene-Based Bis-, Tetrakis-, and Hexakis(terpyridine) Ligands and Their Ruthenium(II)–Bis(terpyridine) Complexes: Synthesis and Photophysical Properties. *Eur. J. Org. Chem.* **2013**, 3640-3644 (2013).

8. Li Y, *et al.* Facile Thermodynamic Conversion of a Linear Metallopolymer into a Self-Assembled Hexameric Metallomacrocycle. *Chem. Commun.* **51**, 5766-5769 (2015).

9. Chakraborty S, *et al.* Programmed Molecular Engineering: Stepwise, Multicomponent Assembly of a Dimetallic Metallotriangulane. *Eur. J. Org. Chem.* **2016**, 5091-5095 (2016).

10. Chakraborty S, *et al.* Terpyridine-Based, Flexible Tripods: From a Highly Symmetric Nanosphere to Temperature-Dependent, Irreversible, 3D Isomeric Macromolecular Nanocages. *J. Am. Chem. Soc.* **139**, 3012-3020 (2017).

11. Xie T-Z, *et al.* Supramolecular Arrays by the Self-Assembly of Terpyridine-Based Monomers with Transition Metal Ions. *Dalton. Trans* **47**, 7528-7533 (2018).

12. Li Y, *et al.* Giant, Hollow 2D Metalloarchitecture: Stepwise Self-Assembly of a Hexagonal Supramolecular Nut. *J. Am. Chem. Soc.* **138**, 10041-10046 (2016).

13. Liu D, *et al.* 3D Helical and 2D Rhomboidal Supramolecules: Stepwise Self-Assembly and Dynamic Transformation of Terpyridine-Based Metallo-Architectures. *Chem. Commun.* **52**, 9773-9776 (2016).

14. Jiang Z, *et al.* Self-Assembly of a Supramolecular Hexagram and a Supramolecular Pentagram. *Nat. Commun.* **8**, 15476 (2017).

15. Wu T, *et al.* Stepwise Self-Assembly of a Discrete Molecular Honeycomb using a Multitopic Metallo-Organic Ligand. *Chem. Commun.* **53**, 6732-6735 (2017).

16. Zhang Z, *et al.* Supersnowflakes: Stepwise Self-Assembly and Dynamic Exchange of Rhombus Star-Shaped Supramolecules. *J. Am. Chem. Soc.* **139**, 8174-8185 (2017).

17. Jiang Z, *et al.* Constructing High-Generation Sierpiński Triangles by Molecular Puzzling. *Angew. Chem. Int. Ed.* **56**, 11450-11455 (2017).

18. Sharghi H, Nasseri MA, Niknam K. Phenol-Containing Macrocyclic Diamides as New Catalysts in the Highly Regioselective Conversion of Epoxides to β-Hydroxy Thiocyanates. *J. Org. Chem.* **66**, 7287-7293 (2001).

19. Drewry JA, *et al.* Phosphopeptide Selective Coordination Complexes as Promising Src Homology 2 Domain Mimetics. *Inorg. Chem.* **51**, 8284-8291 (2012).

20. Wang M, *et al.* Self-Assembly of Concentric Hexagons and Hierarchical Self-Assembly of Supramolecular Metal–Organic Nanoribbons at the Solid/Liquid Interface. *J. Am. Chem. Soc.* **138**, 9258-9268 (2016).

21. Nicolas G, Schmitt J-L, Lionel A, Lehn J-M. DOSY NMR Experiments as a Tool for the Analysis of Constitutional and Motional Dynamic Processes: Implementation for the Driven Evolution of Dynamic Combinatorial Libraries of Helical Strands. *Angew. Chem. Int. Ed.* **47**, 2235-2239 (2008).

22. Schulze BM, Watkins DL, Zhang J, Ghiviriga I, Castellano RK. Estimating the shape and size of supramolecular assemblies by variable temperature diffusion ordered spectroscopy. *Org. Biomol. Chem.* **12**, 7932-7936 (2014).
